# Biomaterials and Bioactive Natural Products from Marine Invertebrates: From Basic Research to Innovative Applications

**DOI:** 10.3390/md20040219

**Published:** 2022-03-22

**Authors:** Giovanna Romano, Mariana Almeida, Ana Varela Coelho, Adele Cutignano, Luis G Gonçalves, Espen Hansen, Denis Khnykin, Tali Mass, Andreja Ramšak, Miguel S. Rocha, Tiago H. Silva, Michela Sugni, Loriano Ballarin, Anne-Marie Genevière

**Affiliations:** 1 Marine Biotechnology Department, Stazione Zoologica Anton Dohrn, Villa Comunale, 80121 Naples, Italy; acutignano@icb.cnr.it; 2 3B’s Research Group, I3B’s—Research Institute on Biomaterials, Biodegradables and Biomimetics of University of Minho, Headquarters of the European Institute of Excellence on Tissue Engineering and Regenerative Medicine, AvePark—Parque de Ciência e Tecnologia, Barco, 4805-017 Guimarães, Portugal; mariana.almeida@i3bs.uminho.pt (M.A.); miguel.rocha@i3bs.uminho.pt (M.S.R.); tiago.silva@i3bs.uminho.pt (T.H.S.); 3 ICVS/3B´s—PT Government Associate Laboratory, 4710-057 Braga, Portugal; 4 ITQB NOVA, Instituto de Tecnologia Química e Biológica António Xavier, Universidade Nova de Lisboa, Av. da República, 2780-157 Oeiras, Portugal; Varela@itqb.unl.pt (A.V.C.); lgafeira@itqb.unl.pt (L.G.G.); 5 CNR-Institute of Biomolecular Chemistry, Via Campi Flegrei 34, 80078 Pozzuoli, Italy; 6 Marbio, UiT-The Arctic University of Norway, 9037 Tromso, Norway; espen.hansen@uit.no; 7 Laboratory for Immunohistochemistry and Immunopathology (LIIPAT), Department of Pathology, Oslo University Hospital-Rikshospitalet, 0450 Oslo, Norway; denisdnr@gmail.com; 8 Faculty of Natural Science, Department of Marine Biology, Charney School of Marine Sciences, University of Haifa, Haifa 3498838, Israel; tmass@univ.haifa.ac.il; 9 National Institute of Biology, Marine Biology Station, Fornače 41, SI-6330 Piran, Slovenia; adreja.ramsak@nib.si2; 10 Department of Environmental Science and Policy, University of Milan, Via Celoria, 2, 20133 Milan, Italy; michela.sugni@unimi.it; 11 Department of Biology, University of Padova, Via U. Bassi 58/B, 35100 Padova, Italy; 12 Biologie Intégrative des Organismes Marins (BIOM), Observatoire Océanologique de Banyuls-sur-Mer, Sorbonne Université, CNRS, 1 Avenue Pierre Fabre, 66650 Banyuls-sur-Mer, France; anne-marie.geneviere@obs-banyuls.fr

**Keywords:** marine natural products, marine biomaterials, marine invertebrates, bioactivity, stem cells

## Abstract

Aquatic invertebrates are a major source of biomaterials and bioactive natural products that can find applications as pharmaceutics, nutraceutics, cosmetics, antibiotics, antifouling products and biomaterials. Symbiotic microorganisms are often the real producers of many secondary metabolites initially isolated from marine invertebrates; however, a certain number of them are actually synthesized by the macro-organisms. In this review, we analysed the literature of the years 2010–2019 on natural products (bioactive molecules and biomaterials) from the main phyla of marine invertebrates explored so far, including sponges, cnidarians, molluscs, echinoderms and ascidians, and present relevant examples of natural products of interest to public and private stakeholders. We also describe omics tools that have been more relevant in identifying and understanding mechanisms and processes underlying the biosynthesis of secondary metabolites in marine invertebrates. Since there is increasing attention on finding new solutions for a sustainable large-scale supply of bioactive compounds, we propose that a possible improvement in the biodiscovery pipeline might also come from the study and utilization of aquatic invertebrate stem cells.

## 1. Introduction

Historically, natural products have played a key role in drug discovery, especially for cancer and infectious diseases [1,2,3]. Although most drugs still derive from terrestrial sources, the marine environment represents a unique resource of natural bioactive products as many marine compounds have chemical characteristics not found in natural terrestrial products. Aquatic invertebrates, due to their high genetic richness, have been a major source of marine natural products (MNPs) of social value, as they produce molecules (enzymes, biopolymers, bioactive compounds, secondary metabolites) that can find applications in various fields as pharmaceutics, nutraceutics, cosmetics, antibiotics, antifouling products, biomaterials and more [4].

The relevance of bioactive compounds of marine origin in drug development is demonstrated by the fact that currently thirteen sea-derived drugs have been approved in the EU and/or USA, four of which received approval in the last three years [5]. These drugs were developed for the treatment of different diseases including carcinoma, chronic pain, and Alzheimer’s disease. In addition, the clinical pipeline in 2020 contained more than twenty drug candidates in different clinical trials in phase III, II, or I [6]. The number of natural products isolated from marine organisms has indeed grown rapidly and continues to provide significant chemical biodiversity that contributes to the development of new therapeutic agents.

The most studied marine invertebrates as sources of bioactive compounds include sponges, cnidarians, molluscs, echinoderms and ascidians [4], as also testified by the drugs currently available for therapeutic applications developed from marine natural products isolated from species belonging to these groups of animals [7,8]. The first compound developed for clinical use is a synthetic analogue of the C-nucleoside cytarabine (or Ara-C) isolated from the Caribbean sponge *Tethya crypta*. It was approved in 1969 and is still used to treat acute myelocytic leukaemia and non-Hodgkin’s lymphoma [9]. Another analogue of a nucleoside isolated from the same species is viradabine (Vira-A^®^), approved in 1976 as an antiviral against Herpes simplex. Thirty-two years later, Trabectedin (Yondelis^®^), an alkaloid isolated from the tunicate *Ecteinascidia turbinata* [10], was approved as an anticancer agent. Since then, other marine products were approved as anticancer agents, including eribulin mesylate (Halaven^®^), an analogue of halichondrin B from the sponge *Halichondria okadai,* plitidepsin (Aplidine^®^), a cyclic peptide from the ascidian *Aplidium albicans*, two derivatives of dolastatin 10 (brentuximab vedotin, Adcetris^®^), and polatuzumab vedotin (POLIVY^®^) from the mollusc *Dolabella auricularia* [7]. In addition, the potent analgesic ziconotide (Prialt^®^) produced by the gastropod mollusc *Conus magus* [11] is in clinical use for treating chronic pain. 

Although it has been demonstrated that symbiotic microorganisms produce many of the secondary metabolites initially isolated from macro-organisms, there is convincing evidence that a certain number of them are directly synthesized and released by cells of various tissues of the macro-organism, including immune cells deriving from the differentiation of stem cells [12]. Ideally, the possibility to produce bioactive compounds using aquatic invertebrate-derived immortalized cell lines would be of great importance in developing societal improvements and advances [12]. This could remove one of the major bottlenecks represented by a sustainable supply of sufficient amounts of bioactive compounds to support preclinical development and all phases of clinical trials required for a new drug to reach the market. Unfortunately, up to now, all the efforts to establish long-term cultures of aquatic invertebrate cells have failed to achieve significant results [13]. Therefore, the knowledge of the gene networks involved in biological processes underlying stem cell differentiation as well as in the biosynthetic pathways of useful bioactive metabolites is of great interest not only in medicine but also for industrial enterprises. In this sense, the continuous development of genomic, transcriptomic, proteomic and metabolomic tools is providing new opportunities to identify and understand those mechanisms and processes. 

There is also an increasing amount of scientific information available on marine biomaterials from marine invertebrates, among which the most promising are marine biominerals, collagen, chitin and adhesive proteins [14]. These biomaterials show high biocompatibility and may find several applications in the biomedical field for tissue engineering and regenerative medicine [15], drug delivery [16] and sutureless wound closure [17].

In this review, we analysed the literature of the years 2010–2019 on natural products (bioactive molecules and biomaterials) from the main phyla of marine invertebrates and present relevant examples with the aim of identifying natural products of interest to public and private stakeholders. We also discuss the potentialities of the main phyla of marine invertebrates in terms of the production of useful natural compounds and in light of advanced approaches and technologies that can provide a sustainable exploitation of valuable products from this source.

## 2. Sponges

Sponges (phylum Porifera) include nearly 9000 species of filter-feeding, benthic organisms living (mainly) in seawater and (about 220 species) freshwater environments. They are considered basal metazoan and include the clades Calcarea, Hexactinellidae, Demospongiae and Homoscleromorpha.

### 2.1. Biomaterials

In recent years, marine organisms have been emerging as an alternative and sustainable source of biomaterials and other compounds of biomedical interest, prompted by their safety regarding zoonosis and the lack of ethical constraints. Marine sponges present an interesting and promising biotechnological potential which is still vastly unexplored. Indeed, the number of Porifera species known to date, which is around 8500 species [18], is far higher compared to species studied for biomaterials development, that would include a few dozens of species. These ancient animals are a source of various compounds that have been proven to have applicability in biomedical research, such as biosilica, collagen/spongin and chitin (Table 1). These biomaterials have common specific features that make them ideal for tissue engineering approaches, such as low immunogenicity and cytotoxicity, and biodegradability. Additionally, the skeleton of some sponges is a suitable material for tissue engineering templates as it possesses a highly interconnected porous architecture, similar to bone structures, that allow culturing cells and guiding cell growth and differentiation, stimulating the regeneration of human tissues to recover lost functions [15]. Given the unique and particular characteristics of sponges’ components, most developed applications aim at bone tissue engineering, although they are currently being employed in innovative biomedical applications, helping to disclose the full potential of blue biotechnology.

Biosilica is an inorganic polymer produced by poriferan from monomeric silicate substrates to build up their inorganic skeleton. The main players in poriferan silicification are low molecular weight proteins, such as silicateins and cathepsins in demosponges, while glassin, collagen, and chitin are involved in the formation of glass sponge (Hexactinellida) exoskeleton [45]. Interestingly, the patterning of silica architecture is based, in both hexactinellids and demosponges, on axial filaments composed primarily of actin [45]. 

Biosilica is able to stimulate mineralizing activity and induce osteogenesis in vitro, supporting tissue mineralization [46]. Therefore, it has been used in various biomedical applications, mainly in hard tissue engineering. For example, biosilica extracted from the demosponge *Suberites domuncula* enhanced mineralizing activity of osteoblasts by favouring the formation of hydroxyapatite, making this material promising for studies of bone replacement [23]. Wang and colleagues have employed biosilica and polyphosphate to supplement biologically inert alginate [47]. The supplemented alginate promoted the growth and differentiation of human mesenchymal stromal cells (hMSCs), which could be advantageous for application in 3D tissue printing of hMSCs and for the delivery of hMSCs in fractures [47]. In another example, surface modification of biosilica obtained from the calcination of the *Petrosia ficidormis* body allowed osteoblasts to grow and colonize the highly porous and interconnected bioceramic structure, inducing hydroxyapatite formation [21]. In addition, microspheres prepared by encapsulation of β-tricalcium phosphate (β-TCP) supplemented with silica have presented morphogenetic activity on bone-forming cells in vivo, demonstrating that these biosilica-based scaffolds are promising biomaterials for bone repair/regeneration [25].

Silicate, a silica compound, has been shown to stimulate in vivo osteogenesis, demonstrating potential to ameliorate osteoporotic disorders [24]. Bioceramics obtained from *P. ficidormis*, *Chondrosia reniformis* and *Agelas oroides* have been considered suitable for biomedical applications, namely in tissue engineering, due to the coating of the surface resembling hydroxyapatite and its biocompatibility [19]. Furthermore, it has been shown that, due to their lack of cytotoxicity, these bioceramics can be used as substitutes for synthetic Bioglass^®^ [20].

Collagen is the most abundant structural protein present in animal extracellular matrices, and it is strikingly similar in both invertebrates and vertebrates. This allowed the development of various applications using collagen from invertebrates as a biomaterial. Spongin is a protein of collagenous nature identified in the exoskeleton of some demosponges. Although the spongins’ exact molecular composition is not elucidated yet due to its abundant diversity within this clade of marine sponges, the presence of a collagenous domain and of short non-fibrillar type-IV-related collagens were demonstrated [48,49]. Many authors consider spongin to be collagen, but only a detailed proteomic analysis could definitely clarify this issue. This review refers to spongin as sponge collagen. 

Various structures to support the regeneration of distinct tissues or drug delivery applications can be developed using sponge collagen as scaffolds, hydrogels, particles or membranes. Collagen from *Ircinia fusca* resulted in a promising biomaterial, as it successfully promoted osseous tissue formation [40]. In combination with chitosan and hydroxyapatite, it has been employed in the development of scaffolds mimicking the required properties of bone extracellular matrix (ECM) aiming at bone tissue engineering with encouraging results [43,44]. Marine sponge collagen has application also in skin care and regeneration. *C. reniformis* collagen has been isolated and incorporated in formulations for topical administration, assessing the effect on skin biophysical parameters [50]. Moreover, this marine sponge collagen has been used on the development of micro- and nanoparticles envisaging the dermal delivery of drugs [51,52]. It has been also used as biomaterial for the development of membranes capable of mimicking human basal lamina, thus supporting and promoting the migration, adhesion, and growth of epithelial cells for epithelial repair, regeneration or replacement [39]. Other membranes for tissue engineering and regenerative medicine approaches have been produced using this collagen, with the possibility of alternatively improving the mechanical properties or the antioxidant performances of the derived biomaterial by adapting the extraction procedure [41]. This versatility allows the tailoring of a membrane’s properties to the requirements of a specific application. Horny sponges (Order Dictyoceratida) are also a source of collagen with biomedical applicability as a bio-based dressing for topical drug delivery [16]. Due to its glycosaminoglycans content, this natural sponge skeletal scaffold is a bioactive and biocompatible carrier able to regulate the wound healing processes by releasing the drug while absorbing the excess of the wound exudate [16]. 

Chitin is a polysaccharide functionally comparable to keratin with many proven biotechnological and industrial applications. Chitin has been isolated from various demosponges, mostly from Verongiida, and in the biomedical field it has been mainly used as a scaffold for supporting various cell types, promoting their proliferation and differentiation due to its biocompatibility and mechanical properties [34,36,37,38]. Most sponges possessing chitin in their skeletal networks, mineralized with silica and calcium carbonates, are also partially brominated [53,54]. *Aplysina aerophoba* is a renewable source of unique 3D microporous chitinous scaffolds which are cyto-compatible and support attachment, growth and proliferation of hMSCs in vitro, being suitable for tissue engineering strategies [34]. The chitinous structure of this animal has also been studied as ready-to-use scaffolds for cultivation of cardiomyocytes, confirming the biocompatibility of the sponge biomaterial with this cell type [36]. Chitin-based scaffolds generated from verongid sponges promoted adhesion, proliferation and differentiation of adipose tissue-derived hMSCs into osteogenic and adipogenic lineages [38]. This finding enables the development of new biocompatible and functionally-active bioengineered structures. Naturally prefabricated 3D chitin scaffolds preserving their fibrous interconnected structure were also obtained from *Aplysina archeri*, raising interest for diverse technological and biomedical fields [37]. Chitin scaffolds isolated from *Ianthella basta* have also been suggested for stem cell-based tissue engineering applications due to their biocompatibility and capacity to maintain the cells’ ability to differentiate [35]. Furthermore, carriers of human mesenchymal stem cells (hMSCs) based on the chitinous skeleton of this species were developed as a stem cell cryopreservation method within 3D tissue engineered scaffolds, supporting cell adhesion, migration and proliferation, and maintaining cell viability after cryopreservation [29]. *Ianthella labyrinthus* is also a species with biotechnological potential, as it is a source of bandage-like 3D chitin scaffolds [27]. These scaffolds are naturally obtained by cleaning the sponge chitinous skeleton and are suitable for culture of cardiomyocytes differentiated from human induced pluripotent stem cells (iPSC-CMs), showing promise for the development of sponge chitin-based absorbable haemostats [27]. The biomedical potential of sponge-derived 3D chitinous scaffolds is extensive, as chondrocytes cultured in structures originated from various marine sponges are able to synthesize an ECM similar to that found in other cartilage tissue engineering constructs both in vitro and in vivo [55]. In a different approach, the natural 3D chitinous scaffold obtained from the skeleton of *Ianthella flabelliformis* was used as drug delivery biomaterial [28]. The prospective for the development of sponge chitin-based biomaterials for biomedical applications is auspicious, as recently many new and renewable sources of this polysaccharide have been discovered [30,31,32,33].

### 2.2. Bioactive Molecules

**Figure 1:**
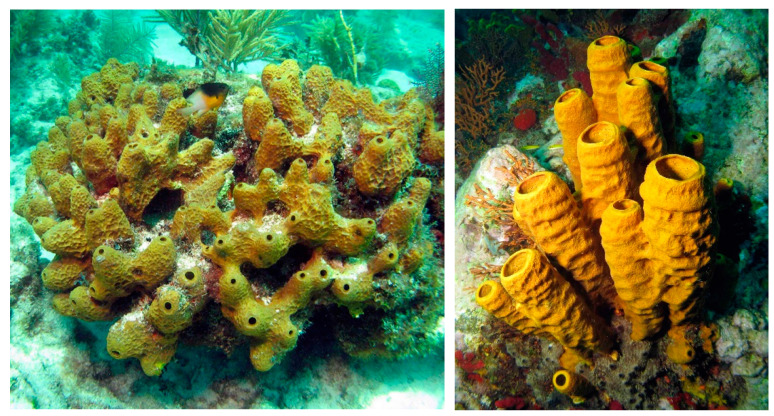
The demosponge *Smenospongia aurea* (**left**) and *Aplysina fistularis* (**right**). Photo by Joseph Pawlik (https://spongeguide.uncw.edu/, accessed on 13 February 2022).

**Figure 2:**
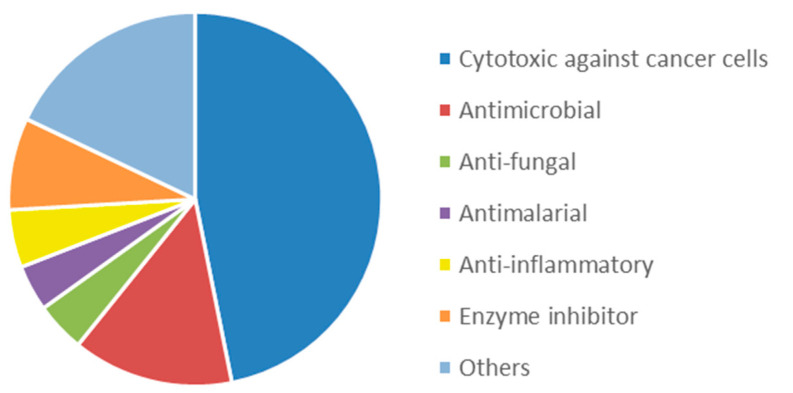
Proportion of different bioactivity associated to sponge-derived MNP, according to data in Table S1.

The interest in bioactive secondary metabolites from marine invertebrates started in the 1950s with the identification of modified nucleotides in extracts of the Caribbean sponge *Cryptotethya crypta* [56]. This discovery led to the development of cytarabine (Cytosar-U^®^) [57], the first drug on the market derived from a marine natural product. Since then, marine sponges (Figure 1) have remained a rich source of structurally diverse natural products, and 2639 new compounds were reported between 2010 and 2019 as reviewed in the ‘Marine Natural Products’ review articles published annually in *Natural Products Reports*, e.g., [58,59,60]. The majority of these compounds, more than 1700 or 65%, were initially described as bioactive. The dominating structural classes were terpenes (35%), alkaloids (29%) and lipids (22%), whereas peptides, polyketides and other molecules constituted 14% of the compounds (Table S1). The majority (about 50%) of the bioactive compounds showed cytotoxicity towards tumour cell lines (Figure 2). Compounds with antimicrobial/antibacterial bioactivity are the second most represented (14%). Interestingly, several MNPs from sponges were identified as possible inhibitors of protein phosphatase [61], proteasome [62], topoisomerase [63] and other key proteins and enzymes involved in cell cycle and apoptosis induction (Table S1).

Sponges are well known for containing substantial amounts of symbiotic microorganisms, including bacteria, fungi and microalgae. It has thus long been assumed that many of the structurally diverse and bioactive secondary metabolites originally isolated from sponge extracts are produced by such microorganisms. It is therefore fair to say that the sponge holobiome is the source of the rich chemical diversity. 

**Figure 3:**
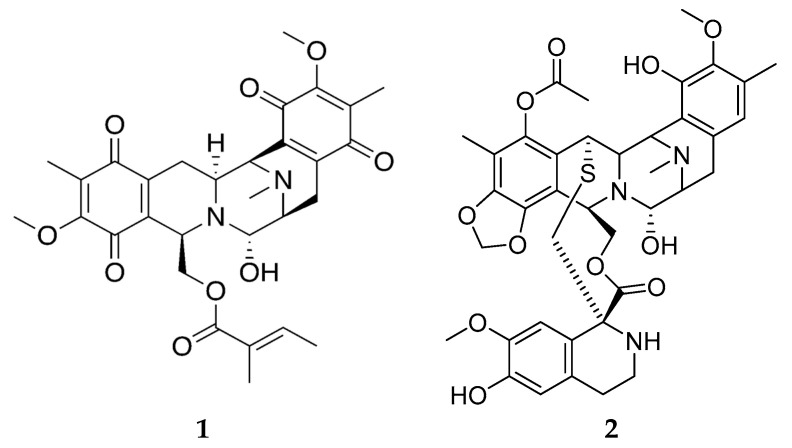
Renieramycin E (**1**) and ecteinascidin-743 (**2**).

Technical developments in molecular biology and analytical chemistry, especially within the field of omics, have made it possible to shed some light on the underlying mechanisms of production of sponge secondary metabolites. Renieramycins are a group of about 30 cytotoxic and antimicrobial tetrahydroisoquinoline quinones isolated from sponges belonging to the genera *Haliclona*, *Xestospongia* and *Neopetrosia*. Tianero et al. [64] demonstrated that renieramycin E (Figure 3) extracted from *Haliclona* sponges collected in Papua New Guinea was actually produced by the symbiont *Endohaliclona renieramycinifaciens*. Due to genome reduction, the symbiont has lost its capability for free living, and lives encapsulated in dedicated bacteriocytes. The term ‘chemobacteriocytes’ was introduced to describe such specialized sponge cells where symbiotic bacteria are living within the sponge, receive nutrients and are sheltered from bacterial competitors while they produce defence molecules that benefit the host sponge. Indeed, *E. renieramycinifaciens* live in the chemobacteriocytes where they produce renieramycins, which protects the *Haliclona* sponge from predators and pathogenic microorganisms. It is interesting to note that the distantly related bacteria *Endoecteinascidia frumentensis* is producing the chemically related defensive compounds ecteinascidins in a similar symbiosis with ascidians [65]. Thus, the renieramycins have the same chemical scaffold as ecteinascidin-743 (Figure 3 (**2**)), which is an approved drug for the treatment of ovarian cancer and advanced soft tissue carcinoma first isolated from the colonial ascidian *Ecteinascidia turbinata,* marketed under the commercial name Yondelis. This underscores the potential use of renieramycins as potential lead compounds for developing new anticancer drugs.

Another investigation used molecular networking to study the chemical diversity of extracts from *Smenospongia aurea* and the bloom-forming cyanobacterium *Trichodesmium* sp., revealing substantial overlap in the metabolomes of the two samples including the cytotoxic smenamides, smenothiazoles and conulothiazoles [66]. The presence of typical cyanobacterial structural motives in these bioactive secondary metabolites found in the sponge also lends support to their hypothesized cyanobacterial origin. 

**Figure 4:**
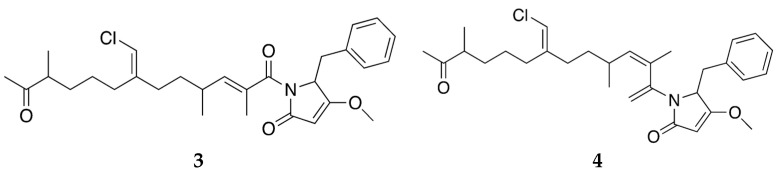
Smenamide A; (**3**) smenamide B (**4**).

Traditionally, chemical investigations of sponges have been based on the identification of major secondary metabolites in crude extracts, either based on bioassay-guided fractionation or chemical analysis as exemplified above. However, more information can be obtained by combining mass spectrometry imaging (MSI) with morphological studies. Using a combination of molecular networking based on HR-MS/MS data, MALDI-MSI and fluorescence microscopy, Cantrell et al. were able to demonstrate that smenamide A and B, (Figure 4, **3** and **4,** respectively) co-localized with cyanobacterial cells in the ectosome of a specimen of *S. aurea*. This observation lends further support to the hypothesis that smenamides are indeed produced by a cyanobacteria [67]. A number of other unrelated secondary metabolites were identified and investigated in the same study. 

**Figure 5:**
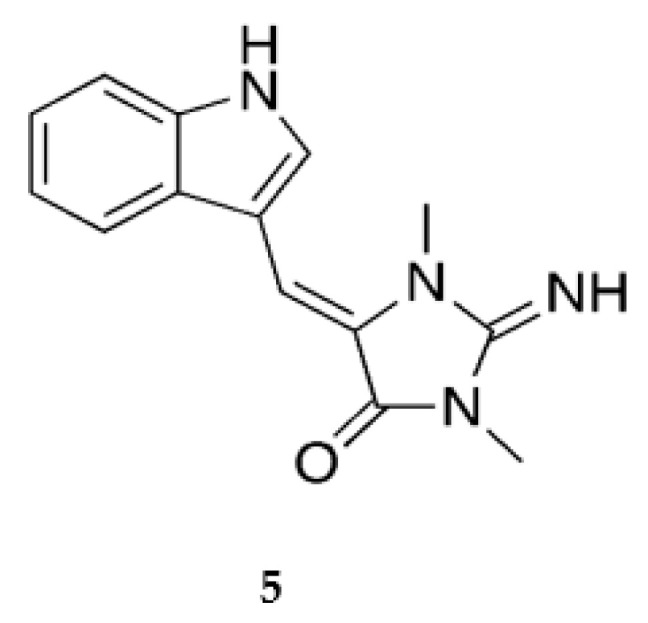
Aplysinopsin.

The tryptophan derivative aplysinopsin **5** (Figure 5) [68] was dereplicated through molecular networking, and MSI analysis revealed that this compound was more or less evenly distributed throughout the sponge, giving no clues about its biosynthetic origin. Aplysinopsin is interesting for its diverse biological activities, including neuro-modulating activities [69]; it would therefore be of interest to identify more analogues in order to investigate the structure–activity relationship between this molecular framework and relevant biological targets.

Considering that the true producers of bioactive natural products isolated from extracts of marine sponges, in many cases, seem to be uncultivable microorganisms, and the fact that is it difficult to activate biosynthetic gene clusters (BGC) for secondary metabolite production when microorganisms are grown in cultures, there is still a great interest in using sponges, collected at various different geographical sites in different seasons, for identifying novel natural products.

## 3. Cnidarians

The phylum Cnidaria includes over 11,000 species distributed among Anthozoa (sea anemones, corals, sea pens), Scyphozoa (jellyfish), Cubozoa (box jellies), Hydrozoa (hydroids) and Staurozoa. The phylum is named after cnidae—the stinging organ which contains toxins used for prey capture and defence.

### 3.1. Biomaterials

Corals are the main sources for biomaterials among cnidarians [15]. Their skeleton is composed of calcium carbonate mainly in the form of aragonite, which is a natural bioceramic. Stony coral skeleton can be used for biomedical applications as its mineral is stable with highly organized porous structure. One of the coral-derived materials is hydroxyapatite [70], which is structurally similar to human bone [71], biocompatible, non-toxic, biodegradable and of low immunogenicity [72]. Coral skeleton has been used both as hard scaffold for bone repair [73,74] as well as in collagen-based scaffold [75]. In addition, soft corals (Octocorallia), for which the mechanical properties of the skeleton depend on environmental conditions, such as the increased stiffness of deep-sea coral [72], also have high potential for biomimetic and biomedical applications [76]. The skeleton structure of black corals (Antipatharia) contains chitin that is also biocompatible and serves as a template for cell adhesion and differentiation [77]. 

Collagen from scyphomedusae is also an interesting resource [78]. Collagen extracted from *Rhizostoma pulmo* found application in tissue engineering, and several assays confirm that jelly-derived scaffolds have optimal adsorption and biocompatibility properties [79]. The Welsh company Jellagen is exploring not only the collagen derived from this jellyfish species but also derived biomaterials, as hydrogels and scaffolds, targeting regenerative medicine and biomedical research. In this regard, jellyfish collagen scaffolds have shown suitability for bone regeneration [80], and hydrogels produced by combination with other marine biopolymers are being proposed for tissue engineering using green processing approaches [81,82]. Other species of jellyfish have been also explored as sources of collagen for biomedicine, namely the Nomura’s jellyfish (formerly named *Stomolophus nomurai meleagris* but then reclassified as *Nemopilema nomurai*) used by Song and colleagues, who proposed collagen scaffolds prepared by freeze-drying capable to support the culture of fibroblast and eliciting an immune response comparable to bovine collagen, widely used in tissue engineering research [83]. The flame jellyfish, *Rhopilema esculentum*, was also used for the extraction of collagen, further re-fibrillized, and the scaffolds resulting from freeze-drying were used as matrix for the chondrogenic stimulation of human mesenchymal stem cells towards the engineering of cartilage tissue [84].

### 3.2. Bioactive Molecules

Cnidarians are known to produce bioactive polypeptides, which are stored in the cnidosomes. These polypeptides have a wide spectrum of activities, mainly as enzymes (phospholipase A2 and metalloproteases) catabolizing prey tissues, pore-forming toxins (cytolysins) causing cell death via osmotic lysis [85] and neurotoxins that rapidly change ion channel functioning. Other bioactive components are represented by vasodilatory biogenic amines such as serotonin, histamine, bunodosine and caissarone (see reviews [59,78,86,87]). 

**Figure 6:**
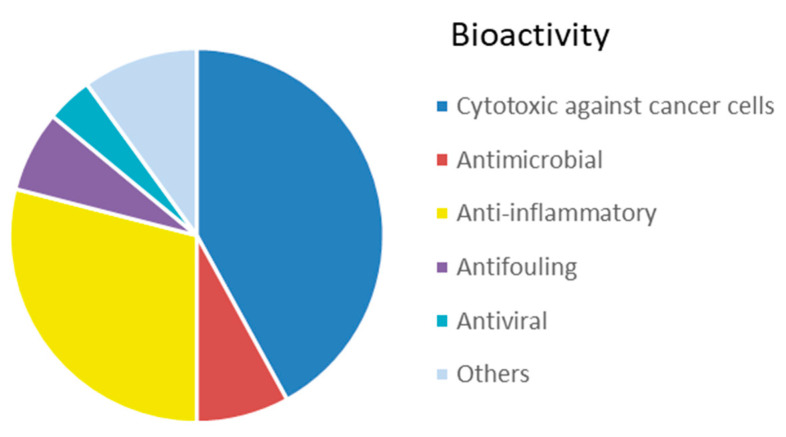
Proportion of different bioactivity associated to cnidarian-derived MNP, according to data in Table S2.

The simple, usually sessile, body of cnidarians is covered by a single-cell epithelium covered by a mucus layer representing a physical protective barrier and a niche for an array of commensal bacteria distinct from the microbiota of the surrounding environment [88]. In order to protect themselves from infections, fouling and predation, they produce many toxic substances. The immune system of cnidarians relies on an innate identification of the pathogens and provides an efficient protection through the synthesis of specific antimicrobial compounds. Currently, 5761 compounds have been described from cnidarians [59], which are the second most prolific source (after sponges) of MNPs among marine invertebrates. An overview of newly discovered MNPs in the period 2010–2019 revealed that the most common MNPs from cnidarians were terpenoids (67%), followed by steroids (21%), and, to a much lower extent, alkaloids (5%) and other compounds (6%) (Table S2). In addition, for this group, cytotoxicity against tumour cell lines is the most represented bioactivity (42%) reported in the literature from 2010 to 2019 (Figure 6), followed by anti-inflammatory activity (29%). A lower proportion of compounds with antimicrobial (8%), antifouling (7%) and antiviral (4%) activities has been identified.

The Asian territories close to tropical areas, particularly in Taiwan, Japan, and China, are the locations where most of the species were collected for extraction of MNPs. The groups from which the highest number of promising bioactive substances were isolated are soft corals and sea pens (Alcyonacea), sea anemones (Actinaria) and hard corals (Scleractinia) belonging to the clade Anthozoa. Anthozoa are a rich source of MNPs with anti-inflammatory, cytotoxic, and neuroprotective activities, as well as anti-nociceptive properties, stimulating bone and tooth regeneration with possible applications in rheumatoid arthritis treatment, Parkinson’s disease, alternative cancer therapy, pain medication and hard tissue growth [89]. Steroids (e.g., from genera *Lobophytum*, *Sinularia*, *Sarcophyton*, *Capnella*, and *Dendronephthya*), have been shown to display antiviral, cytotoxic, anti-inflammatory, anticancer, and antimicrobial properties, as well as cardiac and vascular activities [90]. Steroids of marine origin, and especially polyhydroxylated derivatives, display multiple biological activities, such as cytotoxic, antibacterial, anti-fungal or anti-inflammatory activities. Several steroid compounds were recently isolated from *Sinularia polydactyla*: they demonstrated cytotoxic activity towards human cancer and normal cells [91].

Zoanthid corals and hydroids are rich sources of alkaloids, the best known of which are zoanthoxanthins and zoanthamines [92]. 

Scleractinian corals, especially *Clavularia viridis*, *Briareum excavatum, Antillogorgia elisabethae,* and *Sarcophyton glucum*, produce small cysteine-rich peptides called SCRiPs [79,90,93].

**Figure 7:**
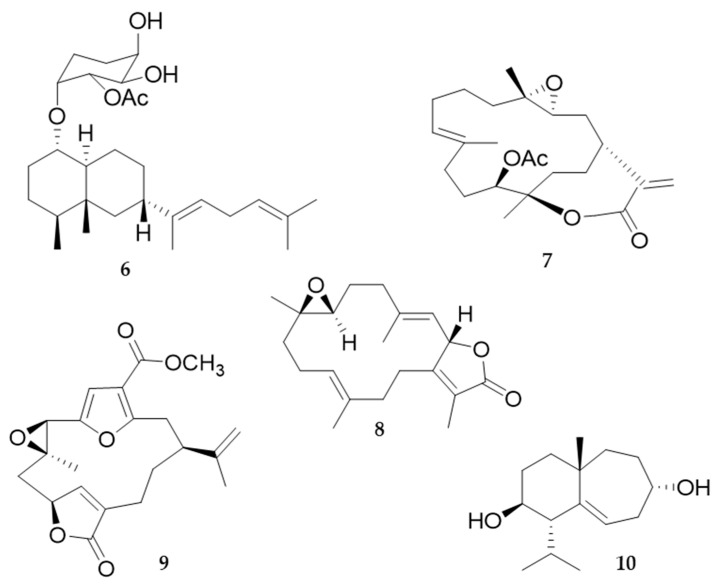
Fuscoside E (**6**); 11-epi-sinulariolide acetate (**7**); sarcophine (**8**); pukalide (**9**); cladidiol (**10**).

**Figure 8:**
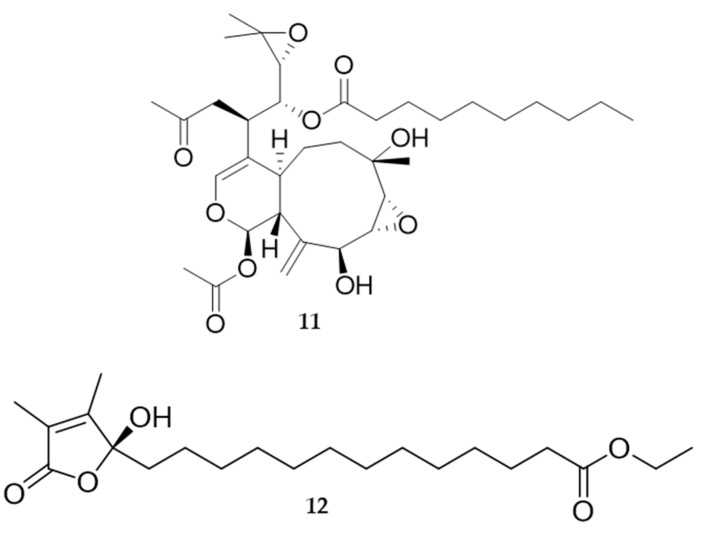
Protoxenicin A (**11**); sinularone I (**12**).

**Figure 9:**
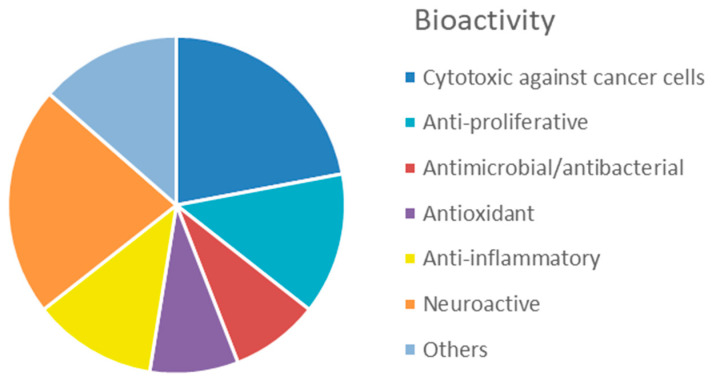
Proportion of different bioactivity associated to mollusc-derived MNP, according to data in Table S3.

**Figure 10:**
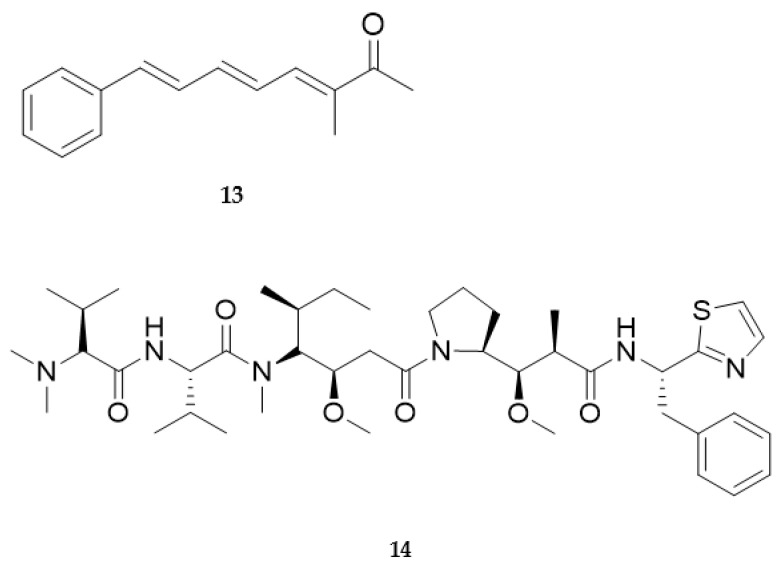
Lignarenone B (**13**) and dolastatin 10 (**14**).

Terpenoids with a wide range of biological activities have been found especially in Alcyonacea and Gorgonacea (Octocorallia), but their biosynthetic pathways are not studied in detail yet [94]. Diterpenes are a class of molecules with anti-inflammatory activity, among which fuscoside E (from *Eunicea fusca*) and sinularin (11-epi-sinulariolide acetate (Ya-s11) (Figure 7, **6** and **7**, respectively), a cembrane-type compound (from *Sinularia querciformis*), showed great potential as a cure for rheumatoid arthritis [89]. Sarcophine (cembranolide diterpene) (Figure 7) was first isolated from the soft coral *Sarcophyton glaucum* [95] and is toxic to fishes that do not prey on this species, as well as to mice, rats and guinea pigs, as demonstrated by in vitro experiments. Its ingestion induced a decrease in cardiac, pulmonary and motor functions, as well as in the control of the body temperature of the animals. Sarcophine (Figure 7, **8**) acts as a competitive inhibitor of cholinesterase [95]. Pukalide (Figure 7, **9**), a furanocembranolide diterpene originally isolated from the octocoral *Sinularia abrupta*, and its derivatives, are widespread among the Octocorallia [94]. Diterpenoids pukalide and 11β, 12β-epoxypukalide isolated from *Leptogorgia virgulata* are effective defence metabolites [96,97]. At non-toxic concentrations, pukalide can exhibit feeding deterrent, anti-attachment, anti-settlement, antifouling, antitumour, anti-inflammatory, narcotic, and cytotoxic properties [98]. Coll et al. [99] suggested that pukalide may also play a role in sperm chemotaxis, as chemical release for ovulation, or as a protective agent for the larval stages. Moreover, several terpenoids (characteristic 14-membered ring scaffold of the cembrane natural products) from corals have inhibitory activity against cholinesterase enzymes. Among them, cladidiol (Figure 7, **10**), is a sesquiterpene isolated from a soft coral of the genus *Cladiella* (from Andaman Island, India), with an IC50 value of 67 mM [100]. 

**Figure 11:**
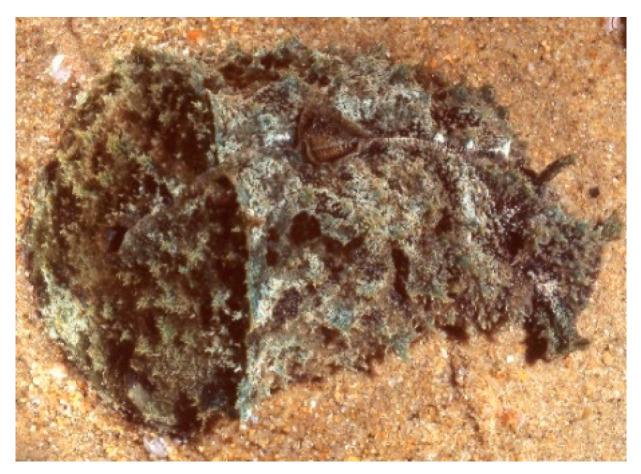
The sea hare *Dolabella auricularia.* (Photo by Dr. Ernesto Mollo.)

During the past decade many new 2,11-cyclized diterpenoids have been isolated from marine octocorals. Most of them have remarkable cytotoxic or anti-inflammatory properties, and also a pronounced antifouling activity [94]. Soft corals are prolific sources of cytotoxic substances against human tumour cell lines (e.g., members of the genus *Xenia* and their xenicane diterpenoids). Recently, two new diterpenoids protoxenicin A (Figure 8, **11**) and protoxenicin B were discovered in *Protodendron repens* with cytotoxic activity against A-549 (lung cancer), HT 29 (colon cancer) and MDA-MB-231 (breast cancer) cell lines [101]. *Sinularia crassa* produces sinularin and 5-episinuleptolide (5EPA), a non-cembranoidal diterpene with cytotoxicity against cell lines K562 and HL60 via HSP90 inhibition. *Sarcophyton crassocaule* is the source of 13-acetoxysarcocrassolide that exerts cytotoxic activity against bladder tumour cells [89].

Neuroactive peptides with affinity for ion channels represent new drugs for the treatment of neurological diseases arising from ion channel dysfunctions. Only a few neuropeptides derived from cnidarians reached the clinical trials, such as ShK-186, also named dalazatide [102].

Several peptides exert antimicrobial activity, such as aurelin from *Aurelia aurita* (Scyphozoa), arminin from *Hydra* (Hydrozoa), damicornin from scleractinian corals (Anthozoa) [90]. Aurelin is an endogenous antibacterial peptide active against Gram-positive and Gram-negative bacteria (IC50 of 7.7 µg/mL towards *Escherichia coli*) [90].

**Figure 12:**
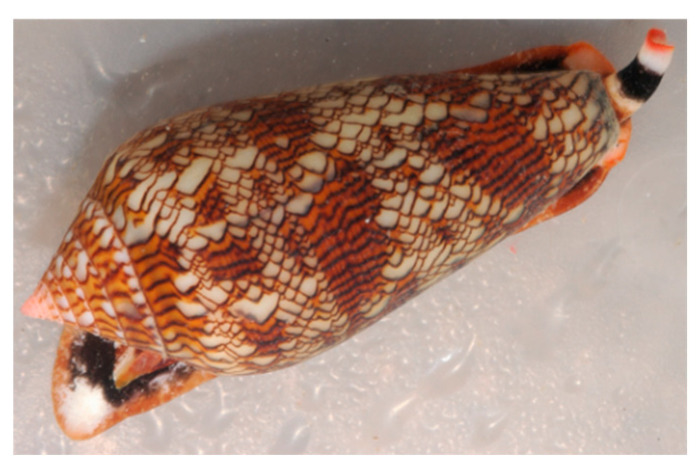
The gastropod *Conus textile.* (Courtesy of Dr. Ernesto Mollo.)

Soft corals also yield a large number of promising metabolites with antifouling activity, therefore 95% of potential antifouling natural compounds come from research on cnidarians, especially soft corals [103,104]. In these organisms, defence against fouling relies on MNPs and periodic sloughs of the external mucus so as to remove the accumulated fouling community. One of the most promising natural antifouling agents identified so far is an isogosterone isolated from an unspecified *Dendronephthya* species [105]. Several other sesquiterpene compounds from the gorgonian *Echinogorgia pseudossapo* were evaluated for their anti-settlement activity against the barnacle *Amphibalanus amphitrite* and the bryozoan *Bugula neritina* larvae. Results indicate that 3-methoxyguaian-10(14)-en-2-ol has significant anti-settlement activity towards *A. amphitrite* larvae with an EC50 value of 17.2 µg/mL (68.2 µM) and showed a 50% inhibition towards the settlement of *B. neritina* larvae at a concentration of 25 µg/mL. The gorgonian coral *Subergorgia suberosa* also produced two steroids with inhibitory activity on the settlement of *B. neritina* larvae with EC50 values of 6.25 and 7.8 µg/mL, respectively, and LD50 > 250 µg/mL [106]. Significant anti-adhesion activity against marine bacteria is shown by the indole alkaloids from the coral *Paramuricea clavata* [107,108]. Several additional compounds, such as sinularones G–I (Figure 8, **12**) from the soft coral *Sinularia* sp. and butenolides from the gorgonian *S. suberosa*, showed moderate antifouling activity against the barnacle *Balanus amphitrite* [107]. Avermectin derivatives are also interesting antifouling compounds; avermectins B1c and B1e, avermectin B2a and ivermectin A1a from the gorgonian *Anthogorgia caerulea* exhibited potent anti-settlement activity towards *B. amphitrite* larvae associated with low toxicity [107]. Inhibition of the biofilm maturation is an important aspect and several cembranoid compounds from the Caribbean gorgonian *Pseudoplexaura flagellosa* are capable of disrupting quorum sensing and inhibiting biofilm maturation of *Pseudomonas aeruginosa, Vibrio harveyi,* and *Staphylococcus aureus* [109].

## 4. Molluscs

Mollusca is a large phylum, constituting about 7% of living animals on Earth. The number of identified marine species is currently estimated around 80,000 [110]. This phylum shows great morphological, ecological, and chemical diversity and is distributed from tropical seas and temperate waters to polar regions, occupying a wide range of ecological niches [111].

### 4.1. Biomaterials

One of the innovative applications of the marine-derived biomaterials in medicine is a generation of surgical protein glues inspired by adhesion proteins from marine creatures. A major problem for a sutureless wound closure in the surgical procedures is a wet environment where most of the so-far developed glues are easily washed out, have a limited tissue adhesion or some toxicity. Many marine invertebrates have evolved their unique proteinaceous adhesives for strong wet adhesion, making them a good model to understand the biological mechanisms involved in attachment to wet surfaces [112,113,114]. Another issue in the wound healing process is a generation of excessive scars after deep dermal injuries including trauma, burn or surgery. Ideally, the surgical protein glue will not only be highly adhesive in wet conditions but will also efficiently prevent formation of scars.

Mussel adhesive proteins (MAPs) display unique adhesive and biocompatibility properties and are known for their great potential in many tissue engineering and biomedical applications [115,116,117,118,119,120]. The natural MAPs are extremely difficult to prepare and therefore have limited applications. However, MAPs may be successfully expressed in bacterial systems for mass production [121]. Yet, proteins produced in bacterial systems lack post-translation phenylalanine modifications (DOPA modifications) essential for MAP properties [122]. Therefore, various modified recombinant proteins or peptides based on natural MAPs but not requiring DOPA modification have been utilized in different applications, such as wound closure and guided bone regeneration [123,124]. They have proven to be flexible, biodegradable, biocompatible and strongly adhesive on various surfaces [121,125]. 

MAPs are tyrosine-rich (appr. 20 mol%) proteins. A photo-crosslinking strategy leading to formation of dityrosine bonds in recombinant MAPs has been utilized to develop a rapidly acting surgical protein glue (South Korea) [17]. Formation of dityrosine crosslinks is known to mediate mechanical and conformational stability and elasticity of protein chains (as example—resilin in dragonfly wing) [126]. Formation of dityrosine bonds may be accelerated via photo-oxidation reaction in a controlled and safe manner. This approach has been used for the generation of a light-activated mussel protein-based bioadhesive (LAMBA) [17]. LAMBA could rapidly close a bleeding and open wound on rat backs with effective adherence to the wound and with minimal inflammation. 

Scar-preventing therapeutics is another application of the protein glues based on MAPs. In impaired scar collagen, most abundant ECM protein is aligned in a single direction with loosely packed fibrils of uncontrolled diameter [127]. Therefore, eliciting normal collagen reorganization—basket weave-like pattern with normal fibril characteristics—in the process of wound healing would be a central strategy to reduce skin scarring. Collagen-modulators such as a decorin are known to regulate linear and lateral fibril growth for collagen packing and organization [128]. A recently published work reports a new protein glue which contains MAPs fused with collagen binding peptides derived from collagen I platelet receptors [123]. Co-treatment of wounds with collagen targeted fusion MAP and dermatan sulphate, the glycosaminoglycan side chain of decorin, stimulates re-epithelization, neovascularization and rapid collagen synthesis during the early stage and prevents pathologic scaring during the remodelling phase. This natural healing-inspired collagen targeting glue may be a promising therapeutic option for improving the healing rate and effective scar inhibition [123].

Molluscs are also gaining increasing attention for mineral-based biomaterial from shells as a source of biogenic calcium carbonates. Moreover, recent studies [129] reported the isolation of conchixes, a biocomposite material containing a complete set of biomacromolecules obtained by gentle demineralization of molluscan shells. This mineral-free biomaterial has been proposed for possible applications in pharmacy and cosmetics.

### 4.2. Bioactive Molecules

In the last decade, the number of new compounds from molluscs substantially increased, with 255 new compounds reported between 2010 and 2019 (‘Marine Natural Products’ series published annually in *Natural Products Reports*). A relatively small proportion of these compounds (about 26%) were described as bioactive. Cytotoxic and anti-proliferative activity against mammalian cell lines and anti-inflammatory activity account for 36% and 12% of the mollusc bioactivity, respectively (Figure 9, Table S3). Of great interest for drug development is the inhibitory activity on voltage gated ion channels displayed by conotoxins (22%).

Among the reported compounds, the dominating structural classes of the bioactive secondary metabolites isolated from molluscs in the selected period were peptides (31%), due to the significant contribution of conotoxins from conids. Terpenes, polyketides and sterols accounted for 24, 15 and 8%, respectively, whereas polyphenols and other compounds combined constituted 22% of the compounds.

In the ‘80s, slow-moving opisthobranch molluscs became an excellent model for investigations on secondary metabolites, playing defensive functions against natural predators. The majority of protective compounds are sequestered from algae, sponges, cnidarians, tunicates or bryozoans through the food chain and stored in external tissues, such as the mantle [130]. However, opisthobranchs are also able to de novo biosynthesize defensive compounds or biotransform compounds obtained from their diet. Often, these defensive molecules revealed an array of biological properties that were exploited, or turned out to be promising, for pharmacological applications, with a special interest for compounds as potential anticancer drugs [131]. 

**Figure 13:**
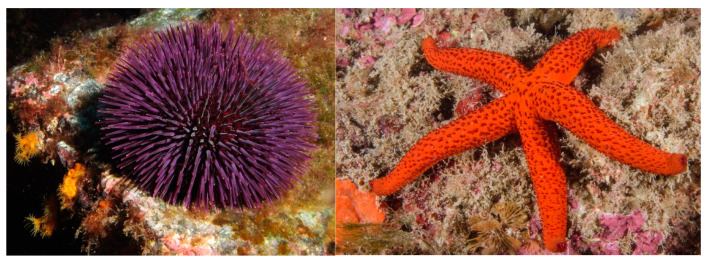
The sea urchin *Paracentrotus lividus* (**left**) and the sea star *Echinaster sepositus* (**right**) (photo by Federico Betti).

Originally isolated from the Mediterranean opistobranch *Scaphander lignarius* [132], lignarenones are a class of aryl octanoids synthesized by polyketide synthase-like enzymes [133,134,135]. Tissue analysis by fluorescence and electron microscopy has established that the synthesis of the aromatic polyketides takes place in the cytoplasm of Blochmann’s glands, specialized secretory cells that are located along the border of the mantle [134]. Lignarenone B (Figure 10, **13**) is an example of a signal molecule released by the mollusc when disturbed and revalued as a potential drug candidate after some years from its first characterization. Very recently, computational analysis predicted, and experimental studies confirmed, the capability of lignarenone B to inhibit the activity of glycogen synthase kinase 3 (GSK3), likely through an ATP competitive and non-competitive allosteric mechanism [136]. Furthermore, this molecule can increase neurite outgrowth in primary cortical neuron cultures, without neurotoxicity. Thus, it could be considered as a starting point to develop new future potential therapeutic agents for the treatment of neurodegenerative diseases such as Alzheimer’s disease. 

**Figure 14:**
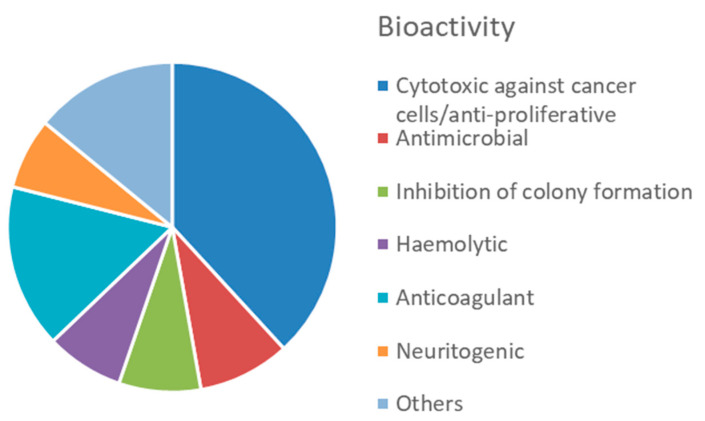
Proportion of different bioactivity associated to echinoderm-derived MNPs, according to data in Table S4.

Despite the de novo origin in molluscan tissues being proven in the above studies and a few other cases [137], the ultimate source of molluscan secondary metabolites is still a matter of debate. The lack of genetic studies leaves open the possibility that gastropod metabolites may derive from symbiotic bacteria or any other source [138]. This is the case of dolastatins (e.g., dolastatin 10, Figure 10, **14**), linear and cyclic cytotoxic peptides originally isolated from the sea hare *Dolabella auricularia* (Figure 11). 

The successive isolation of several dolastatin analogues from cyanobacterial sources strongly supported the microbial origin for several peptidic cytotoxins isolated from the sea hare [139]. 

Dolastatins are mitotic inhibitors, interfering with tubulin formation and thereby disrupting cell division and inducing apoptosis in several tumour cell lines. Many dolastatins were considered promising anticancer drugs with potency against breast and liver cancers, solid tumours and some leukaemias, and are therefore under clinical evaluation. Synthetic analogues of dolastatins, auristatins (POLIVY^®^), in a form conjugated to antibodies, have been recently approved against several tumours, including relapsed/refractory multiple myeloma, metastatic urothelial cancer and non-Hodgkin’s lymphoma (https://www.marinepharmacology.org/approved, accessed on 13 February 2022).

Another interesting group of gastropod molluscs producing bioactive compounds is represented by the Conidae. This group evolved a highly diversified family of neurotoxic peptides, named conotoxins, used as chemical weapons to paralyse their prey. Unlike most bioactive secondary metabolites isolated so far, conotoxins are direct gene products, deriving from post-translational modification of large protein precursors. Conopeptide biosynthesis occurs in the highly specialized venom gland. The up-regulation, in this structure, of peptidylprolyl cis-trans isomerase, suggests a potential and specialized role for this enzyme in the in vivo folding of conopeptides. 

The research on conotoxins began in the early 1970s and the first venom component characterized was alpha-conotoxin GI, a small peptide that proved to be a competitive nicotinic antagonist [140]. Since then, the research has been growing exponentially, and currently it is estimated that more than 80,000 natural conotoxins exist in various cone snails (Figure 12) around the world and each *Conus* species may possess an average of 100–200 conotoxins [141,142]. 

Conotoxins generally consist of 10 to 40 amino acid residues derived from RNA-encoded precursor proteins. Their structure is consolidated by the presence of two to four or more disulphide bonds, which provides an efficient protection from proteases. The enormous variety of conotoxins is due to hypermutation of conotoxin-encoding sequences, fragment insertion/deletion, and mutation-induced premature termination [143]. Research on conotoxins has provided numerous scientific and societal benefits, including their use as diagnostic agents, drug leads, as well as research tools in neuroscience, pharmacology, biochemistry, structural biology, and molecular evolution. In the last few years, a number of reviews focusing on conotoxins have been published [144,145,146], where additional information can be found. The growing interest towards conotoxins for pharmaceutical applications is based on their capability to bind various types of ion channels, thus interfering with the transmission of the neuronal impulse. The synthetic analogue of omega-conotoxin MVIIA, known as ziconotide (Prialt^®^), is a potent analgesic, approved since 2004 for the management of severe chronic pain in humans.

## 5. Echinoderms

The phylum Echinodermata includes about 7000 species, making it the second-largest grouping of deuterostomes and the largest phylum that has no freshwater or terrestrial members. Echinoderms in the adult form live on ocean floors where they play an important role in benthic ecosystems. The echinoderms fall into five clades with overlapping, yet diverse, characteristics: Echinoidea (sea urchins) (Figure 13, left); Holothuroidea (sea cucumbers); Crinoidea (sea lilies, feather stars); Asteroidea (sea stars, starfish) (Figure 13, right); and Ophiuroidea (brittle stars). Echinoderms are known producers of bioactive glycosylated metabolites, dominated by steroidal and sulphated metabolites, saponins and glycolipids [147].

### 5.1. Biomaterials

Echinoderms are a valid source of biomaterials that can be used for various biomedical applications and can be grouped in two main categories: (1) the ‘structural’ materials, mainly used to develop scaffolds for regenerative medicine and tissue engineering; and (2) bioadhesives for different applied fields (Table 2). Among the former, exploitation of echinoderm ECM components, and particularly of collagen, are currently the most active research field. Indeed, marine collagens are a hot topic in biomaterial development [148] as they can represent a valid alternative to mammalian collagen in tissue engineering applications. Collagen has been successfully extracted from various echinoderm taxa, including sea urchins, sea cucumbers and starfish [149]. The collagenous ECM of echinoderms possesses structural features and mechanical properties that are similar to mammalian ECM. However, even more, these animals possess mutable collagenous tissues (MCTs) [150]. MCTs are peculiar echinoderm connective tissues with unique properties in the animal kingdom, being able to undergo drastic, rapid and reversible changes in their mechanical properties [151]. Echinoderm collagen is easily obtained in its native fibrillar form, still superficially decorated by glycosaminoglycans [149,152] and thus fully preserving its structural and functional features. This allows its use for the production of highly biomimetic and mechanically-resistant devices which can include thin membranes or 3D scaffolds for tissue regeneration [153]. Of note is that these devices improved skin regeneration when applied in an in vivo (sheep) model [154]. Recently, MCT-derived collagen has reached the market and is currently one of the top products of a high-tech company devoted to the discovery and development of marine natural compounds (https://mebioscience.com, accessed on 13 February 2022). Similarly, starfish body wall extracts, which mostly consist of ECM components, particularly collagen, are one of the main ingredients of a newly branded anti-age cream produced by a Korean company (https://www.koreanqueens.com/en/home/returning-starfish-cream.html, accessed on 13 February 2022). 

Therefore, echinoderms, and sea urchins particularly, are innovative and alternative collagen sources to produce efficient guided tissue regeneration membranes [149]. In addition to being the direct source of raw material, MCT has also been the source of inspiration for biomaterial development, particularly stimuli-responsive synthetic nanocomposites mimicking the unique mechanical properties of MCTs [155,156]. These echinoderm-inspired biomimetic materials (which are composed of cellulose whiskers and synthetic polymers) were developed to produce mechanically compliant brain microelectrodes [157].

Among the structural materials, echinoderm skeleton was used to obtain bioceramics. The skeletal elements of these animals are made of calcite (CaCO_3_) with high magnesium content and are arranged in stereomes displaying a highly porous and trabecular structure [158]. This provides a high resistance of the skeletal pieces combined with a light weight. This particular architecture originally stimulated the development of biomaterials designed by replication of the skeletal microstructure [159]. In an attempt to find new solutions for bone regeneration, some research was addressed to directly use the porous calcitic architecture as a three-dimensional scaffold for seeding mammalian cells (e.g., osteoblasts), thus providing preliminary evidence of the biocompatibility of the calcite stereome [160,161]. Alternatively, the calcitic skeleton of some sea urchin species was converted to calcium phosphate salts (e.g., hydroxyapatite) via hydrothermal/chemical reaction in order to obtain novel materials for bone regeneration [162,163]. Similar to the ECM components, echinoderm skeleton was also used as source of inspiration for material design rather than directly used for its development. Particularly, the microstructural organization of sea urchin spines (highly ordered nanoparticles in a biomineral mesocrystal matrix) inspired the design of an elastic concrete material that might be used in future construction processes [164].

Lastly, bioadhesives from echinoderms are a recent field of investigation in material science. It is well known that starfish and sea urchins temporarily but firmly attach to the substrate thanks to a duo-gland adhesive system relying on both adhesive and de-adhesive secretions [165]. 

**Table 2:** Biomaterials from echinoderms. (Sorted alphabetically according to Class of Biomaterial.)

Holoturoidea	Sea cucumbers (and other echinoderms)	Proteins/neutral carbohydrates	Mutable collagenous tissue (MCT) components	Mutable collagenous tissue/ECM components	design of an MCT-inspired synthetic material	[166]
Holoturoidea	Sea cucumbers (and other echinoderms)	Proteins/neutral carbohydrates	Mutable collagenous tissue (MCT) components	dermis/ECM components	design of an MCT-inspired stimuli-responsive synthetic nanocomposite	[156,157]
Holoturoidea	Sea cucumbers	Proteins/neutral carbohydrates	Mutable collagenous tissue (MCT) components	dermis/ECM components	design of mechanically tunable synthetic biomaterials	[167]
Holoturoidea	Sea cucumbers	Proteins/neutral carbohydrates	Mutable collagenous tissue (MCT) components	dermis/ECM components	biomimetic design of artificial polymer nanocomposites	[168]
Holoturoidea	*Holothuria forskal, H. leucospilota,* *B. subrubra,* *P. graeffei*	Proteins/neutral carbohydrates	Proteins rich in small side amino acid	Cuvier tubule	bioadhesives	[165]
Holoturoidea	*Holothuria tubulosa*	Proteins	Collagen	dermis/ECM components	membranes for guided tissue regeneration	[149,152]
Asteroidea	*Pisaster giganteous*	Bioceramics	High-magnesium calcite	ossicles (skeletal microstructure)	scaffold for mammalian cell culture	[161]
Asteroidea	*Asterias rubens*	Proteins/glycosylated proteins	Glycosylated proteins	tube feet	bioadhesives	[169,170]
Asteroidea	*Echinaster sepositus*	Proteins	Collagen	dermis/ECM components	membranes for guided tissue regeneration	[149]
Asteroidea	*Asterias rubens*	Proteins	Sea star footprint protein 1 (Sfp1)	tube feet	bioadhesives	[165,171]
Echinoidea	Heart urchins	Bioceramics	High-magnesium calcite	ossicles	production of bioceramic nanopowder	[172]
Echinoidea	Sea urchins	Bioceramics	High-magnesium calcite	ossicles (skeletal microstructure)	production of structured hydroxyapatite material	[173]
Echinoidea	Sea urchin	Bioceramics	High-magnesium calcite	spine	bio-inspired design of super-resistant concrete materials	[164]
Echinoidea	*Tripneustes gratilla*	Bioceramics	High-magnesium calcite	ossicles (skeletal microstructure)	production of magnesium substituted β-tricalcium phosphate for bone graft materials	[162]
Echinoidea	*Paracentrotus lividus*	ECM components	Collagen	peristomial membrane/ECM components	membranes/scaffolds for tissue regeneration	[149,152]
Echinoidea	*Paracentrotus lividus*	ECM components	Mutable collagenous tissue (MCT) components	peristomial membrane/ECM components	decellularized membranes for invertebrate cell culture	[174]
Echinoidea	*Paracentrotus lividus*	Proteins		tube feet	bioadhesives	[175]
Ophiuroidea	Brittle stars	Bioceramics	High-magnesium calcite	dorsal arm plates (microstructure)	brittle-star-inspired micro-lens	[176]

Some of the key proteins of the attachment glue have been identified, and their molecular structure was characterized in an attempt to produce recombinant protein for the development of synthetic adhesive. Indeed, understanding the mechanisms and the key actors behind this very fast adhesion–de-adhesion process can be instrumental for the design of water-resistant adhesives. Recently, fractions of the adhesive protein Sfp1 identified in the adhesive of *Asterias rubens* were obtained by recombinant production and proposed as a material to develop coatings for various biomedical applications [177] and development of new antifouling strategies [165].

### 5.2. Bioactive Molecules

Echinoidea include 950 species distributed across all the oceans, from tropical to polar climates, and inhabiting marine benthic zones from the intertidal to 5000 m. Sea urchins contain several edible species for which effective culture methods have been developed. However, sea urchins have also generated interest for their peculiar defence system which protects them against microbial infections and fouling [178].

Accordingly, antimicrobial activities were identified in sea urchin coelomocytes or coelomic fluid [179], and several antimicrobial compounds were isolated. Among them, antimicrobial peptides, short cationic peptides containing positively-charged amino acid residues, were identified in various sea urchin species. Among them were strongylocins [180,181], centrocin 1 and 2 [182,183] or their analogues [184,185], and paracentrin 1 [186,187]. Sea urchin pigments, which are found in test spines but also in coelomocytes or gonads, gave rise to powerful antioxidant compounds [188]. 

Sea cucumbers belong to the clade Holothuroidea, globally found in deep seas and benthic areas. The body wall of these marine invertebrates contains most of their active constituents, mainly polysaccharides and collagen, which exhibit numerous biological activities, including anticancer, anti-hypertensive, anti-angiogenic, anti-inflammatory, anti-diabetic, anticoagulation, antimicrobial, antioxidant, and anti-osteoclastogenic properties [189]. It also contains sea cucumber saponins, cerebrosides and gangliosides [190,191].

Starfish (class Asteroidea, around 1800 species) are benthic animals that inhabit diversified ocean ecosystems from rocky beaches to the deep sea. Most bioactive compounds, isolated from whole body extracts, are steroids and their glycoside derivatives, including asterosaponins and polyhydroxy steroid glycosides. They are frequently sulphated in the steroid and/or glycoside portions. Starfish-derived compounds have been shown to be promising anticancer and anti-inflammatory agents, but also present neuritogenic, antimicrobial and antifouling properties. Their in vitro cytotoxic effects against various cancer cell lines have been studied through the evaluation of IC50 data, inhibition rates and colony formation [147]. 

Overall, this phylum displays a great variety of bioactive compounds, most of them (38%) with cytotoxic or anti-proliferative activities (Figure 14, Table S4). Products displaying anticoagulant activity (16%), i.e., fucoidans and other sulphated polysaccharides, seem characteristics of echinoderms, compared to other invertebrates. Another characteristic bioactivity associated to MNP from echinoderms is the neuritogenic effect displayed by gangliosides isolated, for example, from the starfish *Asterias amurensis* [192]. Antimicrobial and antioxidant activities represent a lower proportion, accounting for 9% and 7% of the total bioactive compounds, respectively.

**Figure 15:**
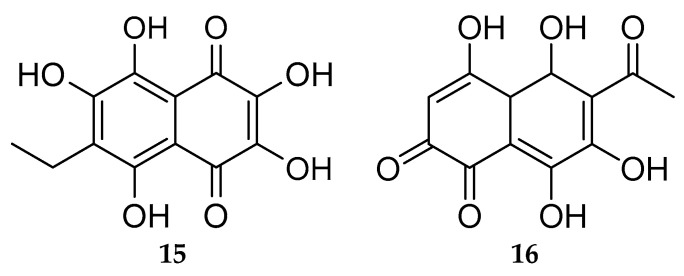
Echinochrome A (**15**); spinochrome E (**16**).

Echinoids are an important source of natural polyhydroxynaphthoquinones (PHNQ), of the 1,4-naphthoquinones group [193]. Investigation of the red-dark pigments derived from the spines and shells of sea urchins led to the identification of a sub-family of PHNQ called spinochromes and echinochrome A **15** (Figure 15); spinochromes A–E (e.g., spinochromes E **16**, Figure 15) are the best known and the more accessible molecule of this class [194]. Found in many sea urchin species, spinochromes are also expressed in coelomocytes, eggs, ovaries and larvae [195]. Their chemical structures were progressively uncovered so that the molecules and various derivatives were synthesized in the laboratory [196,197,198,199]. A distinctive feature of these compounds is their ability to effectively intercept free radicals and bind the Fe^2+^ ions responsible for the formation of reactive oxygen species (ROS) [200,201,202].

The spinochromes A–E show antioxidant activity but with distinct efficiencies and cytotoxicities on human cells [194]. Spinochrome D attenuates doxorubicin-induced mitochondrial damage in human cardiomyocyte cell line AC16 and human breast cancer cell line MCF-7 [203].

Spinochrome E and echinochrome A (see below) were produced by cultured coelomocytes [204], paving the way to the generation of sea urchin cell cultures producing complex bioactive compounds with therapeutic potential.

Echinochrome A, first isolated from the spines of sea urchin *Stomopneustes variolaris* [205], acts as a cardioprotective agent and was further used, in a water-soluble form, as the active substance in the drug Histochrome introduced in Russia for preventing ischemia/reperfusion injury and ophthalmopathic complications [206,207,208]. The antioxidant and anti-inflammatory capabilities of echinochrome A are responsible for the cardioprotective effect [201,209]. Indeed, echinochrome A attenuates the oxidative stress caused by ROS. More recent reports suggest that it could be a candidate molecule to promote cardiac regeneration [210,211,212]. Echinochrome A was also recently investigated in the framework of stem cell therapy, as a potential drug for enhancing in vitro cardiomyocyte differentiation from mouse embryonic stem cells (mESCs) [213] and for promoting ex vivo expansion and stemness maintenances of hematopoietic stem and progenitor cells [214,215]. Additionally, echinochrome A could exert a wide range of biological effects, including anti-fibrosis, anti-diabetic, anti-allergic, anti-acetylcholinesterase, mitochondria-protective and gastro-protective effects, as occurred in experimental models [216,217,218,219,220,221,222,223,224]. A di-glutathionyl functional analogue was synthesized with higher solubility and stability in aqueous solutions, and reduced toxicity while maintaining the anti-ischemic effect [225]. Echinochrome A also showed bactericidal activity [194,226,227] and antiviral ability in particular on herpes simplex virus type 1 [228,229]. 

Many polysaccharides of various biological origins have been isolated and used as a source of therapeutic agents. The most promising activities of these biopolymers are their immunomodulatory and anticancer effects [230]. A neutral, water-soluble polysaccharide, named SEP, was isolated from the eggs of the sea urchin *Strongylocentrotus nudus* [231]. SEP was found to be an α-(1→4)-d-glucan and was reported to display antitumour activity by stimulating immune cells, including NK and T cells, via TLR2 and TLR4 receptors [232]. Combining SEP with cytotoxic drugs [233,234] or other immunoregulatory agents [235] resulted in a potent synergistic antitumour effect in mice.

Two types of acid polysaccharides, a prominent class of glycans, are among the most important components extracted from the body wall of sea cucumber: sulphated fucans (also named fucoidans) and fucosylated chondroitin sulphates (FCS) [236,237,238]. These polysaccharides are often endowed with high bioactivity related to their sulphate functional groups, which can interact with many positively-charged biological macromolecules, enzymes included. Sulphated fucans are complex fucose-rich polysaccharides, often extracted from brown seaweeds in which they were first discovered [239] and, to a lesser extent, from the body wall of sea cucumbers or the egg jelly coat of sea urchins (for review see [240,241,242]). Unlike marine algae, which express sulphated polysaccharides with complex, heterogeneous structures, marine invertebrates synthesize sulphated fucans and sulphated galactans with regular repetitive structures but with variation of the pattern of sulfation and the position of glycosidic linkage [243,244]. This simpler structure can help to elucidate structure–biofunction relationships. Recently, nanomedicine began to use fucoidans especially in the fields of cancer, regenerative medicine, and cardiovascular diseases [240]. In this framework, the specific biotechnological potential of the regular structure of echinoid fucoidans remains poorly investigated. However, it was shown that sulphated polysaccharides isolated from three sea urchin species have different anticoagulant and anti-selectin activities, which give them a potential activity in the attenuation of metastasis progression [245,246]. 

Fucosylated chondroitin sulphates (FCS) are structurally unique glycosaminoglycans found exclusively in marine invertebrates such as the body wall of sea cucumbers [247], crabs [248] and octopuses [249]. FCS are composed of the same chondroitin sulphate structure as vertebrates, although with additional branches of sulphated fucopyranose units. The fine structure of FCS is species-specific, and their biological activity is supposed to depend mainly on the degree of sulfation and position of sulphate groups, as well as on the distribution of branches along the backbone. Among the multiple potential biomedical applications of FCS, their potential anticoagulant and antithrombotic properties are the most interesting for their possible use as alternative drugs with low bleeding risk with respect to heparin anticoagulants. Therefore, FCS have received extensive attention and several recent reviews have been published [250,251,252,253,254]. More recently, synthetic low molecular weight FCS have been examined as an alternative to natural FCS for their limited side effects [255].

Saponins, classified as steroid or triterpenic glycosides, with their amphipathic properties, are good surfactants with the ability to increase the permeability of cell membranes [256]. In Animalia, saponins are biosynthesized only by sponges, starfish and sea cucumbers [257]. Those isolated from Holothuroidea and sponges are triterpenic, although asterosaponins, the characteristic compounds of Asteroidea, are glycosides of sulphated steroids. A review listing asterosaponin structures and their biological functions and bioactivities was recently published [258], so details will not be presented here. Extracts containing asterosaponins are highly ichthyotoxic and haemolytic; additionally, their high abundances in the mucus layer, body wall and stomach suggest their protective action against pathogens and predators [259,260]. Co-ARIS, an asterosaponin isolated from the starfish *Asterias amurensis* egg jelly, has a crucial role in the acrosome reaction (AR) required for oocyte penetration by spermatozoa. It was shown that Co-ARIS induces conformational changes in membrane microdomains, affecting the status of AR-signalling proteins [261]. Asterosaponins isolated from several starfish organs or the whole body have demonstrated anticancer, anti-inflammatory and antimicrobial reported bioactivities [259]. Interestingly, a synergism of anticancer action was identified when 2D and 3D cultures of human melanoma cells were pre-treated with thornasteroside A or asteropsiside A from starfish *Asteropsis carinifera* and then by fucoidan from the brown algae *Fucus evanescens*. This process relies on the regulation of cell cycle protein expression and of a chain of signalling proteins such as MEK1/2, ERK1/2, and MSK1. Produced signals regulate the cell cycle, cell proliferation and cell development, opening up the prospects for the development of effective combined chemotherapeutic methods for melanoma treatment [262]. Other encouraging results come from the enhanced efficacy of chemoradiation therapy by asterosaponin P1, isolated from *Patiria pectinifera*, in reducing the number and size of the colonies of colorectal cancer cells. The radiosensitizing activity of asterosaponin P1 occurred by apoptosis induction through the regulation of anti- and pro-apoptotic protein expression followed by caspase activation and DNA degradation [263]. Nevertheless, these promising bioactivities of asterosaponins are not yet explored, envisaging their commercialization.

## 6. Tunicates

**Figure 16:**
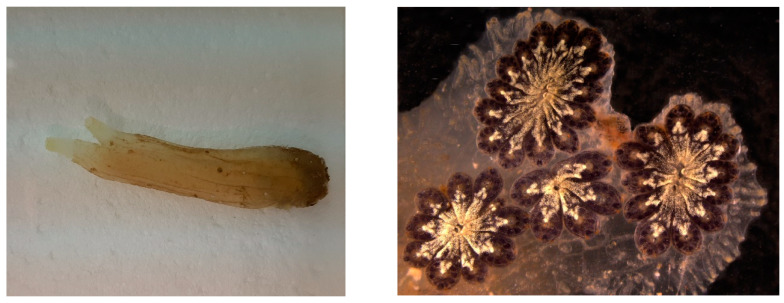
The ascidians *Ciona intestinalis* (**left**) and *Botryllus schlosseri* (**right**).

Tunicates or urochordates are marine invertebrate chordates considered the sister group of vertebrates. They owe their name to the tunic, the external layer that embeds the body. It is secreted by the epidermis and is composed by an ECM rich in collagen and tunicin (a form of cellulose) fibres, and hosts cells deriving from the delamination of the epidermis and from the circulation. Tunicates include sessile species, collectively grouped in the clade Ascidiacea (ascidians) (Figure 16), and pelagic species forming the clades Thaliacea and Larvacea or Appendicularia. About 3000 species of tunicates live in the seas and oceans of the world and 2300 of them are represented by ascidians, the largest and most studied tunicate group. Within tunicates, Ascidiacea is the most diverse clade and comprises benthic and sessile forms.

### 6.1. Biomaterials

Tunicates are also receiving attention from the biomedical field owing to their adhesiveness, regeneration capacity and tunic tissue features.

The tunic is mainly composed of tunicin, a highly crystalline cellulose nanofiber (tunicates are the only known animal source of cellulose), a unique composition among animals, which is found associated with proteins, lipids, sulphated glycans and mucopolysaccharides [264].

Polysaccharides are extensively used in regenerative medicine approaches due to their high biocompatibility and functional properties, among which chitosan and alginate are probably the most studied members. In addition, cellulose is a polysaccharide with low cytotoxicity and, compared to other polysaccharides, has excellent mechanical properties, which makes it also attractive for the development of medical applications, such as wound dressings, bone tissue replacements, drug delivery, vascular grafts and scaffolds for tissue engineering [265,266]. Cellulose nanocrystals (also often referred to as nanowhiskers) which consist of the nanoscale crystalline region of the cellulose polymer, have been isolated from plants and bacteria, and to a less extent from tunicates, and investigated for biomedical applications. Tunicate nanocellulose is exploited for commercial purposes, and significant steps towards the scalable isolation of cellulose from invasive tunicates are being developed, thus offering a potential solution to the numerous challenges which invasive tunicates pose to global aquaculture communities [264,267] and offering a valuable sustainable source to the biomedical field.

Membranes and liquid bandages manufactured from *Styela clava* tunics were prepared for healing skin wounds and showed positive results on skin regeneration [268,269]. Furthermore, a selenium-loaded cellulose film originated from this species accelerated cutaneous wounds during diabetic conditions [124]. Cellulose membranes were also prepared from *Styela clava*, demonstrating an osteoconductive effect in perforated rat frontal bone, which may be applied in injured bones [270]. In addition, aiming at bone tissue regeneration, cellulose nanocrystals isolated from tunicates demonstrated suitability as a scaffold for bone tissue engineering by promoting osteoblast growth and differentiation, and the recovery of damaged tissue [271]. This biomaterial isolated from *Ascidiella aspersa* induced guidance in skeletal muscle myoblasts, which could be used for skeletal muscle tissue engineering [272]. More recently, cellulose nanocrystals isolated from *Halocynthia roretzi* were employed in the development of hydrogels demonstrating highly ordered architectures and outstanding mechanical performances, having potential biomedical applications as artificial biomaterials, such as ligaments and tendons [273].

In addition to cellulose, the tunic also contains various glycosaminoglycans that can be extracted and find a potential use in the sanitary field [274,275].

In addition to containing tunicin, the tunic also contains proteins with 3,4-dihydroxyphenylalanine (DOPA) with a catechol moiety, and 3,4,5-trihydroxyphenylalanine (TOPA) with a pyrogallol moiety [276]. These compounds are associated with wound healing and are also present in the adhesive components of tunicates [277,278]. Though the exact mechanism of the adhesion in tunicates remains unclear, TOPA compounds were recently proposed in tunicate-inspired adhesives, envisaging biomedical applications. Gallol-functionalized copolymers exhibited stronger adhesive performances (typically seven times stronger in water) than the widely-used catechol groups, which could bring a new route to the development of new tunicate-inspired gallol polymers with potential as bioadhesives for the medical arena and for other applications [279]. In this regard, natural polymers such as chitin and its derivate chitosan are being conjugated with gallic acid, which has pyrogallol moieties, to create tunicate-mimetic hydrogels. These adhesives showed higher tissue adhesive properties than fibrin glue and mussel-mimetic adhesives [280] and improved haemostatic functions with potential application as sealants of internal tissues [281]. Similarly, a bio-inspired hydrogel conjugating pyrogallol to hyaluronic acid with two routes of pyrogallol oxidation revealed minimal cytotoxicity and immunogenicity in vitro and in vivo, demonstrating versatile applicability for tissue engineering (cell grafting) and drug delivery (therapeutic angiogenesis) [282].

In tunic proteins, the pyrogallol groups can form coordinative complexes with metal ions in the tunicate haemolymph to heal the tunic tissue. Based on metal–pyrogallol coordination of tunicate tissue, a gallic acid/metal ion complex-mediated coating was created to accelerate hydroxyapatite remineralization on human teeth, showing potential applicability in the treatment of dentin hypersensitivity [283]. In another example, inspired by the hemocompatibility of heparin, magnetic nanoparticles with heparin-mimetic coating were developed using TOPA as a biological adhesive, and showed great application potential in haemodialysis as recycling anticoagulants [284]. A list of biomaterials from tunicates is reported in Table 3.

### 6.2. Bioactive Molecules

Ascidians are the source of a great variety of bioactive molecules of potential sanitary applications, including cytotoxic, antimitotic, antiviral and antimicrobial compounds [285,286,287,288,289], as well as are molecules that are of interest to the biomedical field owing to their adhesiveness [290]. Ascidian bioactive compounds belong to a high variety of chemical categories [287,291]. Most of the metabolites synthesized by ascidians contribute to create the physicochemical barrier preventing the entrance of foreign organisms in the internal fluids or the tunic colonization by encrusting organisms. In addition, natural endosymbionts contribute to producing part of these compounds to prevent other microorganisms from entering the host [287,292,293]. 

**Figure 17:**
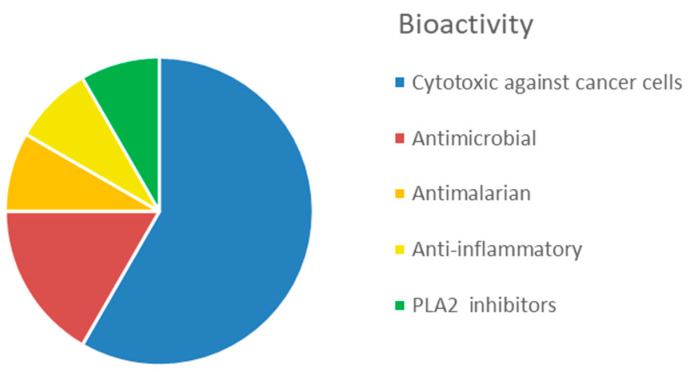
Proportion of different bioactivity associated to tunicate-derived MNP, according to data in Table S5.

The most represented chemical class among the bioactive secondary metabolites isolated from tunicates in the selected period was that of alkaloids, including 50% of the isolated compounds, followed by polyketides (37%) and peptides (13%) ([289]; Table S5). Cytotoxicity against mammalian cell lines and anti-proliferative activity were the most frequently assigned bioactivities, accounting for 58% of the total number of bioactive molecules (Figure 17). Compounds with cytotoxic and antineoplastic properties isolated from ascidians belong to disparate chemical classes, and three of them have been entered into clinical trials. 

**Figure 18:**
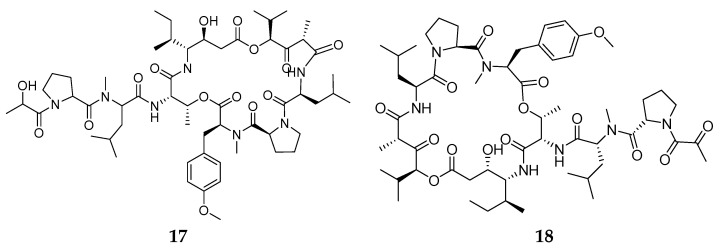
Didemnin B (**17**) and plitidepsin (**18**).

Didemnin B **17** (Figure 18, left), a cyclic dispeptide from the colonial ascidian *Trididemnum solidum* [294], was the first marine natural product to enter a clinical trial [295]. It displays strong antimitotic effects. Its collateral effects and the observation that dehydrodidemnin B, also known as plitidepsin **18** (Figure 18, right) (Aplidine^®^), was more potent than didemnin B [296] led to the end of the clinical development of didemnin B [297].

Plitidepsin, better known by the commercial name of Aplidine^®^ (marketed by PharmaMar, S.A), isolated from the Mediterranean colonial ascidian *Aplidium albicans* [298], exerts anticancer activity via the cell cycle arrest at G1-S, likely through the inhibition of protein tyrosine phosphatases, a class of enzymes frequently associated with tumour progression [299]. It can also inhibit protein synthesis by interacting with the elongation factor 1α and induce apoptosis in cultured cells through the overactivation of c-Jun N-terminal kinases [297]. Didemnin B (and, likely, also the closely-related Aplidine^®^) is synthesized by the symbiotic α-proteobacteria *Tistrella mobilis* and *Tristella bauzanensis* [295,300]. 

Trabectedin, formerly ecteinascidin-743 (ET-743) (Figure 2), is an alkaloid first isolated from the Caribbean colonial ascidian *Ecteinascidia turbinata* in 1990. It is the first anticancer drug from a marine source introduced to the market after encompassing all the clinical trials, and is particularly effective against solid tumours, especially soft tissue sarcomas and relapsed ovarian cancer [286,301]. Today, it is marketed by PharmaMar with the commercial name Yondelis^®^. It has a unique mechanism of action based on the interaction with the minor groove of deoxyribonucleic acid (DNA), which leads to strand breaks and consequent inhibition of DNA synthesis and gene transcription, as well as interruption of the cell cycle and induction of apoptosis [287,297]. When used at a micromolar concentration, trabectedin can inhibit a number of transcription factors [302]. In nature, trabectedin is produced by the bacteria endosymbiont *Candidatus Endoecteinascidia frumentensis* [302,303,304], but, due to its scarcity in the ascidian tissues, today it is obtained by chemical synthesis. 

Many other compounds exerting anticancer activity were isolated from ascidians, most of them produced by bacterial symbionts (see [286,288] for a review; Table S5), but none of them have entered the clinical trials up to now.

A huge variety of antimicrobials has been extracted from tunicates. They belong to disparate chemical classes, such as polysulfides, alkyl sulphates, terpenes, amino alcohols, spiroketals, alkaloids, furanones, peptides and others [291]. Some of them are synthesized by symbiotic organisms colonizing the tunic or the internal fluids [292,293].

Most of the known antimicrobial peptides are produced by ascidian circulating cells, mainly immunocytes (i.e., cells involved in immune responses) for defence purposes [305,306,307,308,309,310,311,312]. In *Halocynthia roretzi*, the tetrapeptides halocyamines A and B are produced by cytotoxic morula cells (MCs) [305], and their cytotoxic activity is likely related to their diphenol rings, representing suitable substrates for the enzyme phenoloxidase (PO), also stored inside MCs. The enzyme induces oxidative stress by oxidizing phenols to quinones with the consequent production of ROS [313]. The haemocytes of the congeneric species *H. aurantium* synthesize the α-helix peptide dicynthaurin [309] and the cationic peptide halocidin [308]. Clavanins A–D (histidine-rich, α-helix peptides) and clavaspirin are synthesized by *Styela clava* MCs [311,314] and the octapeptide plicatamide by haemocytes of *Styela plicata* [312]. In lysates of haemocytes of the same species, five cationic antimicrobial peptides, called styelins, were identified and isolated [310,311,315,316]. In *Ciona intestinalis*, cytotoxic haemocytes synthesize two types of α-helix antimicrobial peptides. The injection of foreign material in the body wall enhances the transcription of the corresponding genes [306,307,317,318].

## 7. Omics Approach to Discover New Marine Natural Products

Traditionally, the biodiscovery pipeline relied on bioassay-guided strategy to isolate bioactive natural products. Although this method had a high success rate in identifying these compounds, it also presented some drawbacks. The major issue of this approach is a high probability of re-isolation of already known compounds [319]. Alternative strategies based on the application of omic techniques, such as genomics, transcriptomics, and metabolomics, are currently driving many important advances in the field, increasing the possibility to find new bioactive metabolites [320]. Indeed, the improvement of sequencing technologies is providing a greater availability of genomic data, and the use of bioinformatic analysis allows to predict the biosynthetic potential of an organism, which made genome mining an increasingly widespread approach for biodiscovery [319,321,322,323,324]. Nevertheless, the genetic potential is not always fully expressed and biosynthetic gene clusters (BGCs) for natural product production may remain silent under laboratory culture conditions [324]. Transcriptome analyses can provide important information to distinguish between silent and expressed BGCs [320], and also proteomics can assist in screening for expressed BGCs, producing new natural products. The latter approach was also employed without the necessity for prior DNA sequence information [320]. However, a major contribution for the biodiscovery is provided by high-throughput techniques such as metabolomics. This approach has proven to be extremely useful in exploring chemical diversity at faster rates than classical bioassay-guided strategy [325]. In the following paragraphs we focus on the description of the metabolomics approach used for marine natural product discovery, as it currently represents one of the most widespread biodiscovery strategies.

### 7.1. Metabolomics

**Figure 19:**
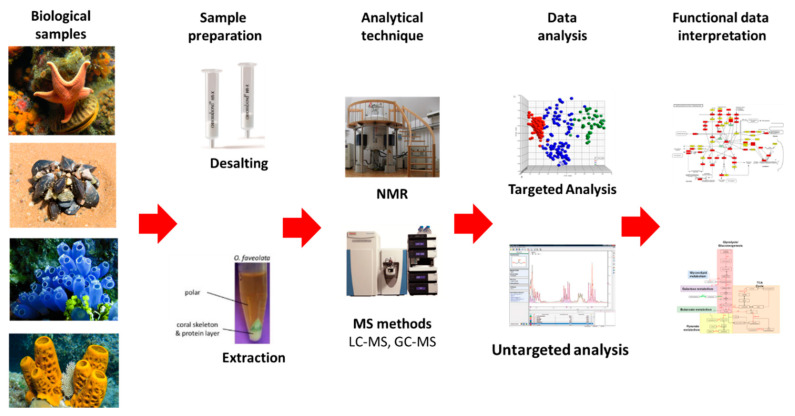
Metabolomics workflow for marine biodiscovery.

Metabolomics is directed towards comprehensive analysis of low molecular weight metabolites (<1500 Da), the products of many biological pathways. Among this type of molecule, the secondary metabolites are essential for competitiveness and defence of organisms in their habitats. Today, metabolomic approaches offer a great potential for the biodiscovery of new natural products with many advantages compared to the traditional (time-consuming) bioprospecting strategies. An exhaustive untargeted characterization of the metabolome, where all signals from either Nuclear Magnetic Resonance spectroscopy (NMR) or mass spectrometry (MS) are annotated, is challenging due to the high number of signals and to metabolites’ chemical diversity. Nonetheless, as an outcome, a multitude of new compounds can be detected, identified, characterized in their structure and quantified, allowing the establishment of isolation protocols to proceed to bioactivity assays. The metabolomic workflow for potential new drug identification is depicted in Figure 19.

The choice of the proper extraction solvent is one of the main challenges in untargeted metabolomics since it should ideally solubilize the entire metabolome to give a snapshot of the cellular content at the molecular level. This issue is quite difficult to accomplish given the co-occurrence of metabolites with different polarity. In a typical workflow, organic solvents with medium polarity such as methanol, ethanol or acetone are used with wet or lyophilized biological samples. Most recent protocols include a solid phase extraction (SPE) purification step aimed at fraction enrichment and avoiding salt interference [326,327,328,329]. 

#### 7.1.1. NMR and MS Approaches

**Figure 20:**
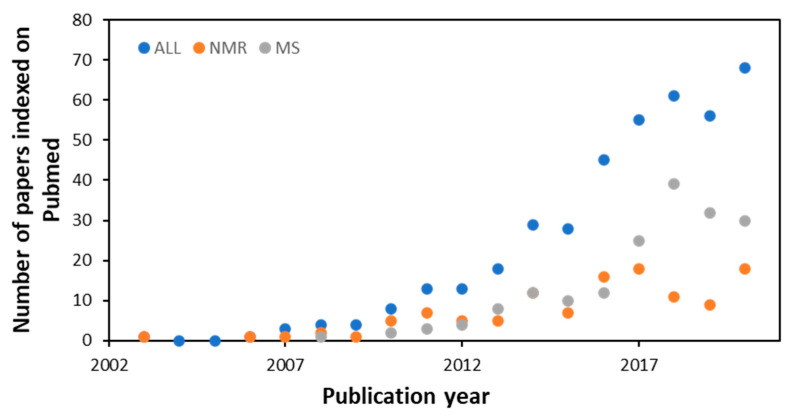
Number of papers using metabolomic approaches in marine invertebrates by year (search was performed on March 2021 in PubMed limited to original papers that mention (((metabolomic OR “metabolic profile”) AND (“marine invertebrate” OR echinoderm OR cnidarian OR mollusc OR sponge OR tunicate))) or (((metabolomic OR “metabolic profile”) AND (“NMR” OR “Nuclear Magnetic Resonance”) AND (“marine invertebrate” OR echinoderm OR cnidarian OR mollusc OR sponge OR tunicate))) or (((metabolomic OR “metabolic profile”) AND (“mass spectrometry” OR “LC-MS” OR “GC-MS” OR “MS”) AND (“marine invertebrate” OR echinoderm OR cnidarian OR mollusc OR sponge OR tunicate))) in the title, abstract or keyword).

NMR was the analytical methodology used in the first metabolomic studies involving marine invertebrates [330], but quickly MS was also employed, with a comparable number of publications using MS or NMR approaches until 2017, when the number of papers with an MS approach outperformed the studies using NMR (Figure 20).

MS, particularly when coupled to Ultra High Performance Liquid Chromatography-UHPLC-MS, is preferred in metabolomics studies due to its higher sensitivity and chromatographic resolution, smaller sample size required and relatively lower operational costs. Moreover, the recent MS instrumentation technology and the developments of new software tools and databases made MS analysis easier, generating high-resolution data leading to high mass accuracy determinations below 1–2 ppm [331]. Despite absolute quantification being possible, it requires the use of standardization with each individual analyte, due to the dependence of signal intensity from its ionization ability. Relative quantification is frequently used for comparison of metabolites amounts in diverse experimental conditions. Unlike MS, NMR detection does not require the derivatization of non-ionizable compounds and allows a direct determination of absolute concentrations by mono-dimensional ^1^H-NMR spectra. However, spectra of raw extracts may result in being extremely complex, requiring bi-dimensional experiments to resolve individual components. Nevertheless, NMR has the advantage of being a very reproducible technique, allowing spectra acquisition at different study times [332]. Finally, the sample is not consumed during the analysis, allowing eventual re-analyses.

#### 7.1.2. Compounds Databases

MNP databases are an essential support in dereplication of raw extracts. Compound identification requires the use of structural information, including molecular mass, structural formula, and ideally also MS patterns along with NMR spectral signals. An updated compilation of dedicated natural products databases is available in Wolfender et al., 2019 [333]. Its subset with applicability in MNP identification, open-access and searchable by biological source is resumed to seven databases from which only four include more than 50 MNPs entries. These data are presented in Table S6 together with the number of compound entries for the four phyla depicted in this publication. Few general metabolomics databases can be easily searched by biological source, such as the *Dictionary of Marine Natural Products* [334]. In the general database Metabolights, 130 compounds from sponges were sorted, although none were found for tunicates, molluscs and echinoderms, which contrasts with the number of entries found in the partial publicly available *Dictionary of Marine Natural Products*. Therein, 2891 TMSE (tunicate, mollusc, sponge and echinoderm) characterized compounds are reported. Moreover, compound identification is also dependent on a search tool compatible with available databases formats that, jointly with the limited coverage of MNPs, contributes to hamper this process. Some databases integrate experimental spectral information; however, others are chemical structure databases from which computational algorithms predict molecular masses, MS^2^ profiles and NMR spectra [335]. Helpfully, certain ones of those databases also incorporate compound bioactivities, organism source, molecular targets, and data on their clinical progress.

## 8. Concluding Remarks and Future Perspectives

In the past decades, more than 2000 MNPs have been identified and characterized. The most abundant chemical class is represented by terpenes from sponges (35.0%) and molluscs (23.0%). Steroids were the most relevant class from echinoderms, accounting for 42% of total bioactive compounds, whereas alkaloids were the most frequently described chemical class from tunicates (50.0%). For all marine invertebrate phyla considered for this review, the most frequently assigned bioactivities were cytotoxicity and anti-proliferative activity against tumour cell lines. This proportion may be related with the incidence of studies that are developed, which testifies the great interest of the scientific community and society in finding new drugs to fight cancer. 

Despite the great number of bioactive compounds described so far, those that have either been marketed or are under development are relatively few. In December 2020, the clinical marine pharmaceutical pipeline consisted of 13 marine-derived drugs approved by the United States Food and Drug Administration (FDA) and/or the European Medicines Agency (EMA), and 1 drug approved in Australia [5,7]. Bottlenecks before reaching the market by the high number of isolated natural products are still present, for example difficulties in harvesting organisms of extreme environments, low amount of natural product in producing organisms, problems in obtaining a sustainable supply of the compound, difficulties in isolation and purification procedures, ecological impact on natural populations, and insufficient investment by pharmaceutical companies [336]. Notwithstanding these difficulties, there is a general consensus that, in the near future, the marine environment will still be the most important source of novel bioactive compounds. In the last decades, there has been a ‘renaissance’ in marine drug discovery due to technological developments and the use of marine microbial genomics to provide biosynthetic pathways [337]. The technological advancements may help in exploring the great animal biodiversity in the sea, as most of the research on MNPs is concentrated on less than 1% of the invertebrate species, especially those living in shallow waters. The accessibility of deep waters, up to 200 m in depth, via remotely operated vehicles, is expanding the range of collection of new species that can be of interest for the discovery of new MNPs [337]. In addition, up to now the bulk of investigation was carried out along the well-studied coasts of Europe and North America, whereas the remaining coastal environments of the planet are still scantily known. This opens new opportunities for specific research programs to discover new marine drugs from animals living in poorly investigated marine regions, with particular reference to the biodiversity hotspots of tropical regions of Indo-Pacific and Oceania [338]. 

Since 2010, metabolomic investigations have been progressively applied to identify new MNPs from marine invertebrates. Metabolomics can furnish researchers with information on the metabolite composition of a biological extract and provide data on changes in metabolite composition under various physiological states. In addition, it can contribute to the identification of new chemical scaffolds, useful in designing new synthetic drugs [336]. A community-wide effort is currently under way to give larger access to the metabolome datasets to curate and dereplicate previously known MNPs in order to enable novel MNP discoveries [339]. Moreover, metabolomics has been particularly helpful to differentiate MNPs that have either been sequestered from symbionts, produced or promoted by the associated microbiome, or synthesized by the animal [340,341,342,343,344,345].

One of the problems in the production of bioactive compounds by extraction from natural populations of aquatic invertebrates is its low yield. Animals that cannot be collected in large amounts from the field, as repeated re-collection can lead to the depletion of the species, should be taken in consideration only for early screening assays. Therefore, the establishment of ex situ, inland culturing methods for species of interest can be a solution to ensure a constant provision of animals. This represents a first goal for further research advancements, such as the establishment of transgenic lines of animals expressing high levels of the molecules of interest. Although some edible species are widely cultivated (e.g., bivalves, holothurians, solitary ascidians), other species (e.g., sponges, cnidarians and some echinoids) may be difficult to keep under laboratory conditions for long periods of time so as to obtain the release of gametes and complete the life cycle in captivity.

Another possibility to have a continuous production of MNPs of interest is the use of cell culture, but up to now, we have no immortalized cell lines from marine invertebrates, although significant progress has been made in the establishment and maintenance of primary cultures of cells from sponges, bivalves [346] and echinoderms [347]. Such cell lines, if established, could be useful in providing a continuous supply of MNPs of interest produced by the cells themselves or by their endosymbionts. Attempts to maintain and grow cell lines from marine invertebrates were seriously hampered by the contamination by protists, mainly thraustochytrids, that grow and divide more rapidly than the cells of interest [347]. A possible way to overcome this problem is the establishment of immortal cell lines starting from the adult stem cells that are abundantly present in many marine taxa and provide them with the high regeneration potential of many species [12]. However, again, no serious advancement has been made in this direction. Some improvements have been achieved in sponge cell culture: establishment of primary cultures from dissociated and cryopreserved cells of several sponge species, optimization of nutrient media, stimulation of cell division with growth factors and mitogens, transient expression of immortalizing genes, establishment of methods for three-dimensional culture in hydrogels [348]. These advancements were further expanded by exploring the 3D culture in polymeric disks, hydrogel layers and gel microdroplets, with the latter being suggested for the in vitro production of bioactive compounds [349]. One of the problems in establishing immortalized cell lines from sponges is their high dynamism and plasticity, suggesting that sponge cells divide but also die at high rates, most likely to rapidly adapt to a changing micro-environment [350]. Thus, future studies should also focus on reducing cell apoptosis, in addition to focusing on stimulation of cell division. The production of bioactive molecules by cells in culture may also be hampered by the possible cytostatic or cytotoxic effects that compounds may exert on the producer cells. Müller obtained a successful in vitro production of Avarol from the primmorphs of the sponge *Dysidea avara* [351]. In that case, the successful production was explained according to Avarol’s cytostatic rather than cytotoxic effect, which does not imply the induction of apoptosis and consequent cell death of the sponge primmorphs. 

Genome and transcriptome mining can also represent useful strategies to identify new bioactive compounds. For instance, a computational “reverse search” approach, in which amino acid sequences were analysed for secondary structure prediction, has been successfully applied to discover new antimicrobial peptides with α-helix conformation in the ascidian *C. intestinalis* [307].

As for compounds produced by unicellular endosymbionts that cannot be cultured in vitro, metagenomics and genome mining can provide new knowledge on their biosynthetic pathways. In addition, once identified, the genes of interest can be cloned and inserted into organisms that are easy to culture, such as the laboratory strains of *Escherichia coli,* for heterologous expression and production of MNPs [352]. This approach can also be used for those microorganisms that can be cultured but do not adequately express the genes for natural product biosynthesis under laboratory conditions [336].

The technological and knowledge advancement may also provide support for the exploitation of marine biomaterials of interest for the biomedical field. These materials are receiving increasing attention since they present some important advantages with respect to their synthetic analogues, such as better biocompatibility and excellent biodegradability. In addition to their direct use, as illustrated in this review, marine biomaterials are also of interest for the growing field of bionics and biomimetics, for which these materials are taken as models for the creation of new composites inspired by nature [14]. In this regard, the mechanisms of their formation are particularly interesting, and fundamental science may play a key role in the advancement in this field, since further progress in biomimetic research and application relies on the understanding of mechanisms of biostructure formation as well as of their structural features at the molecular level.

In conclusion, marine invertebrates still represent a rich source of bioactive compounds, as testified by the high number of MNPs isolated in the last decade and the increasing interest for marine-derived biomaterials. To disclose the treasure offered by these organisms, a great effort is needed to integrate different disciplines and combine multiple approaches to go one step forward in marine drug discovery.

## Figures and Tables

**Table 1:** Biomaterials from sponges. (Sorted alphabetically according to Class of Biomaterial.)

Class	Producer Species	Class of Biomaterial	Biomaterial	Origin/Structural Component	Possible Applications	References
Demospongiae	*Petrosia ficidormis,*	Bioceramics	Silicate, Calcium carbonates Silicate/calcium salts	whole body	Support for tissue regeneration	[19]
Demospongiae	*Petrosia ficidormis, Chondrosia reniformis* and *Agelas oroides*	Bioceramics	Calcium phosphate (hydroxyapatite)	skeleton	Substitutes for synthetic Bioglass^®^	[20]
Demospongiae	*Petrosia ficidormis*	Inorganic polymer	Biosilica	whole body	3D support for osteoblast adhesion and growth	[21]
Demospongiae	*Spongia agaricina, Spongia officinalis, Spongia zimocca*	Inorganic polymer	Hydroxyapatite	whole body	Bone substitute material	[22]
Demospongiae	*Suberites domuncula*	Inorganic polymer	Biosilica	skeleton	Stimulates mineralizing activity	[23]
n.a.	n.a.	Inorganic polymer	Silicate	skeleton	Stimulates osteogenesis in vivo	[24]
n.a.	n.a.	Inorganic polymer	Silica/silicatein	skeleton	Regeneration of bone tissue	[25]
n.a.	n.a.	Inorganic polymer	Biosilica/polyphosphate	skeleton	Promotes growth and differentiation of hMSCs *; 3D tissue printing of hMSCs *; Delivery of hMSCs * in fractures	[25]
Demospongiae	*Spongia agaricina*	Inorganic polymer	Hydroxyapatite	whole body	Bone tissue engineering	[26]
Demospongiae	*Spongia agaricina*	Inorganic polymer	Hydroxyapatite	whole body	Tissue engineering (bone scaffolds)	[22]
Demospongiae	*Ianthella labyrinthus*	Polysaccharides	Chitin	skeleton	Scaffolds to culture cardiomyocytes differentiated from human-induced pluripotent stem cells (ipsc-cms); 3D tissue engineering	[27]
Demospongiae	*Ianthella flabelliformis*	Polysaccharides	Chitin	skeleton	Drug delivery biomaterial	[28]
Demospongiae	*Ianthella basta*	Polysaccharides	Chitin	skeleton	Tissue engineering and regenerative medicine	[29]
Demospongiae	*Pseudoceratina purpurea*	Polysaccharides	Chitin	skeleton	Biomedicine	[30]
Demospongiae	*Mycale euplectellioides*	Polysaccharides	Chitin	skeleton	Biomedicine	[31]
Demospongiae	*Acarnus wolffgangi and Echinoclathria gibbosa*	Polysaccharides	Chitin	skeleton	Biomedicine	[32]
Demospongiae	*Pseudoceratina arabica*	Polysaccharides	Chitin	skeleton	Biomedicine	[33]
Demospongiae	*Aplysina aerophoba*	Polysaccharides	Chitin	whole body	3D microporous chitinous scaffolds for hMSCs * in vitro	[34]
Demospongiae	*Ianthella basta*	Polysaccharides	Chitin	whole body	Scaffolds for human mesenchymal stromal cells	[35]
Demospongiae	*Aplysina cavernicola,* *Aplysina cauliformis,* *Aplysina fulva,* *Aiolochroia crassa,* *Plysina aerophoba*	Polysaccharides	Chitin	whole body	Chitin scaffolds for chondrocytes attachment	[34]
Demospongiae	*Aplysina aerophoba*	Polysaccharides	Chitin	whole body	Ready-to-use scaffolds for cultivation of cardiomyocytes	[36]
Demospongiae	*Spongia lamella,* *Spongia officinalis* *Hippospongia communis* *Sarcotragus spinosulus*	Polysaccharides/Proteins	Collagen/ proteoglycan	skeletons	Bio-based dressing for topical drug delivery	[16]
Demospongiae	*Aplysina archeri*	Polysaccharides	Chitin	skeletal fibres	Ready-to-use 3D chitin scaffolds	[37]
Demospongiae	*Aplysina fulva* *Aplysina aerophoba* *Ianthella basta*	Polysaccharides	Chitin	skeletons	Directed differentiation of human adipose tissue-derived hMSCs * within chitin-based skeletons	[38]
Demospongiae	*Chondrosia reniformis*	Proteins	Collagen	whole body	Support and promote the migration, adhesion, and growth of epithelial cells	[39]
Demospongiae	*Biemna fortis*	Proteins	Collagen	whole body	Bone repair and bone augmentation	[40]
Demospongiae	*Chondrosia reniformis*	Proteins	Collagen	whole body	Sponge collagenous membranes	[41]
Demospongiae	*Callyspongiidae*	Proteins	Collagen	skeletons	Scaffold for use in bone tissue engineering	[42]
Demospongiae	*Ircinia fusca*	Proteins	Collagen	whole body	Composite scaffolds (marine collagen + chitosan + hydroxyapatite) for matrix-based bone repair and bone augmentation	[43]
Demospongiae	*Aplysina fulva*	Proteins	Spongin	whole body	Spongin-enriched biosilicate scaffolds to support bone formation	[44]

* Human mesenchymal stem cells (hMSCs).

**Table 3:** Biomaterials from tunicates.

Class	Producer Species	Family/Class of Biomaterial	Biomaterial	Origin/Structural Component	Possible Applications	References
	*not identified*	Polysaccharides	Cellulose	tunic	Scaffold for bone tissue engineering	[271]
Ascidiacea	*Styela clava*	Polysaccharides	Cellulose	tunic	Biomaterial for treatment of bone defect	[270]
	*Ascidiella aspersa*	Polysaccharides	Cellulose	tunic	Biomaterial for skeletal muscle tissue engineering	[272]
	*Styela clava*	Polysaccharides	Cellulose	tunic	Membrane for wound healing	[124]
	*Styela clava*	Polysaccharides	Cellulose	tunic	Film for wound healing	[269]
	*Halocynthia roretzi*	Polysaccharides	Cellulose	tunic	Hydrogel for biomedical applications	[273]
	*Styela clava* *Broussonetia kazinoki*	Polysaccharides	Cellulose	tunic	Liquid bandage for wound healing	[268]
	*not identified*	Proteins	TOPA ^1^ proteins	tunic	Adhesive hydrogel for biomedical applications	[280]

^1^ TOPA proteins: DNA topoisomerase 1.

## Data Availability

The data presented in this study are available in [Supplementary Tables S1–S6].

## Supplementary Materials

The following are available online at https://www.mdpi.com/article/10.3390/md20040219/s1, Table S1: Sponge bioactive natural products isolated from 2010 to 2019; Table S2: Cnidarian bioactive natural products isolated from 2010 to 2019; Table S3: Mollusc bioactive natural products isolated from 2010 to 2019; Table S4: Echinoderm bioactive natural products isolated from 2010 to 2019; Table S5: Tunicate bioactive natural products isolated from 2010 to 2019; Table S6: Number of MNP entries for tunicates, molluscs, sponges and echinoderms in metabolite databases.
