# Supplementary material for: Biomaterials and Bioactive Natural Products from Marine Invertebrates: From Basic Research to Innovative Applications
Source: Mar Drugs. 2022 Mar 22;20(4):219. doi: 10.3390/md20040219 (PMC9027906; doi:10.3390/md20040219)
Supplement: Supplementary file 1 [file marinedrugs-20-00219-s001.zip › marinedrugs-1618376-Supplementary Material.pdf]

marine drugs

MDPI

Review

Bioactive natural products and biomaterials from marine invertebrates: from basic research to innovative applications.

Giovanna Romano [1,*], Mariana Almeida [2,3], Ana Varela Coelho [4], Adele Cutignano [1,5], Luis G Gonçalves [4], Espen Hansen [6], Denis Khnykin [7], Tali Mass [8], Andreja Ramšak [9], Miguel S. Rocha [2,3], Tiago H. Silva [2,3], Michela Sugni [10], Loriano Ballarin [11,*] and Anne-Marie Genevière [12]

[1] Marine Biotechnology Department, Stazione Zoologica Anton Dohrn, Villa Comunale, 80121 Naples, Italy; giovanna.romano@szn.it
[2] 3B's Research Group, I3B's—Research Institute on Biomaterials, Biodegradables and Biomimetics of University of Minho, Headquarters of the European Institute of Excellence on Tissue Engineering and Regenerative Medicine, AvePark—Parque de Ciência e Tecnologia, Barco, 4805-017 Guimarães, Portugal; mariana.almeida@i3bs.uminho.pt (M.A.); miguel.rocha@i3bs.uminho.pt (M.R.); tiago.silva@i3bs.uminho.pt (T.H.d.S.)
[3] ICVS/3B´s—PT Government Associate Laboratory, 4710-057 Braga, Guimarães, Portugal
[4] ITQB NOVA, Instituto de Tecnologia Química e Biológica António Xavier, Universidade Nova de Lisboa, Av. da República, 2780-157 Oeiras, Portugal; Varela@itqb.unl.pt (A.V.C.); lgafeira@itqb.unl.pt (L.G.G.)
[5] CNR-Institute of Biomolecular Chemistry, Via Campi Flegrei 34, 80078 Pozzuoli, (NA), Italy; acutignano@icb.cnr.it
[6] Marbio, UiT-The Arctic University of Norway, N-9037 Tromso, Norway; espen.hansen@uit.no
[7] Laboratory for Immunohistochemistry and Immunopathology (LIIPAT), Department of Pathology, Oslo University Hospital-Rikshospitalet, 0450 Oslo, Norway; denisdnr@gmail.com
[8] Faculty of Natural Science, Charney School of Marine Sciences, Department of Marine Biology, University of Haifa, 3498838 Haifa, Israel; tmass@univ.haifa.ac.il
[9] National Institute of Biology, Marine Biology Station, Fornaˇce 41, SI-6330 Piran, Slovenia; adreja.ramsak@nib.si2
[10] Department of Environmental Science and Policy, University of Milan, Via Celoria, 2, 20133, Milan, Italy. michela.sugni@unimi.it
[11] Department of Biology, University of Padova, Via U. Bassi 58/B 35100 Padova, Italy; loriano.ballarin@unipd.it
[12] Biologie Intégrative des Organismes Marins (BIOM), Observatoire Océanologique, Sorbonne Université, CNRS, F-66650 Banyuls-sur-Mer, France; anne-marie.geneviere@obs-banyuls.fr
* Correspondence: giovanna.romano@szn.it; loriano.ballarin@unipd.it

Supplementary Material

Supplementary Table S1. Sponges bioactive natural products isolated from 2010 to 2019. (Sorted alphabetically according to Class/Subclass and then to Chemical class of compounds)

Copyright: © 2022 by the authors. Licensee MDPI, Basel, Switzerland. This article is an open access article distributed under the terms and conditions of the Creative Commons Attribution (CC BY) license (https://creativecommons.org/licenses/by/4.0/).

Class/Subclass	Producer species	Chemical class of compounds	Compound(s)	Activity/toxicity	References
Calcarea/Calcinea	*Leucetta microraphis*	Alkaloids	Spironaadimine	Antimicrobial	[1]
Calcarea/Calcinea	*Leucetta sp.*	Alkaloids	Polyacetylene alkaloids (1)	Cytotoxic against tumour cell lines	[2]
Calcarea/Calcinea	*Latrunculia austini*	Alkaloids	Aleutianamine	Cytotoxic against cancer cell lines	[3]
Calcarea/Calcinea	*Latrunculia biformis*	Alkaloids	Tsitsikammamines	Cytotoxic against cancer cell lines	[4]
Calcarea/Calcinea	*Latrunculia biformis*	Alkaloids	Discorhabdin D-L (3)	Cytotoxic against cancer cell lines	[5]
Calcarea/Calcinea	*Leucetta chagosensis*	Alkaloids	Chagosendines A-C	Cytotoxic against cancer cell lines	[6]

Calcarea/Calcinea	*Leucetta chagosensis*	Alkaloids	Imidazole derivatives (5)	Cytotoxic against cancer cell lines	[7]
Calcarea/Calcinea	*Pericharax heterorapis*	Alkaloids	Naamidine J	Cytotoxic against cancer cell lines	[8]
Demospongiae	Verongida (Order) and Thorectidae (Family)	Polyketides	Isoascorbic acid derivative	Enzyme inhibition	[9]
Demospongiae/ Heteroscleromorpha	*Niphates sp*	Alcohol	Pellynols M–O	Cytotoxic against cancer cell lines	[10]
Demospongiae/ Heteroscleromorpha	*Ecionemia geodides*	Alkaloids	Pyridoacridines (2)	Cytotoxic against cancer cell lines	[11]
Demospongiae/ Heteroscleromorpha	*Halichondria sp.*	Alkaloids	Alkaloids (3)	Cytotoxic against cancer cell lines	[12]
Demospongiae/ Heteroscleromorpha	*Haliclona densaspicula*	Alkaloids	Denisanin A-B (2)	Anti-inflammatory	[13]
Demospongiae/ Heteroscleromorpha	*Haliclona sp.*	Alkaloids	Cyclostelettamines (10)	Cytotoxic against cancer cell lines	[14]
Demospongiae/ Heteroscleromorpha	*Haliclona tulearensis*	Alkaloids	Haliclorensins B-C (2)	Cytotoxic against brine shrimp	[15]
Demospongiae/ Heteroscleromorpha	*Haliclona tulearensis*	Alkaloids	Isohalitulin	Cytotoxic against brine shrimp	[15]
Demospongiae/ Heteroscleromorpha	*Iotrochota baculifera*	Alkaloids	Baculiferins A-O (15)	Anti-viral	[16]
Demospongiae/ Heteroscleromorpha	*Jaspis splendens*	Alkaloids	Jasplakinolides (4)	Cytotoxic against cancer cell lines	[17]
Demospongiae/ Heteroscleromorpha	*Latrunculia sp.*	Alkaloids	Alkaloids (2)	Anti-malarial	[18]

Demospongiae/ Heteroscleromorpha	*Pachastrissa nux*	Alkaloids	Kabiramide L	Anti-malarial	[19]
Demospongiae/ Heteroscleromorpha	*Penares sp.*	Alkaloids	Carbolines (2)	Cytotoxic against cancer cell lines	[20]
Demospongiae/ Heteroscleromorpha	*Guitarra fimbriata*	Alkaloids	Azaindoles (5)	Enzyme inhibition	[21]
Demospongiae/ Heteroscleromorpha	*Halichondria panicea*	Alkaloids	Halichondriamines A and B	Cytotoxic against cancer cell lines	[22]
Demospongiae/ Heteroscleromorpha	*Haliclona sp.*	Alkaloids	1-(1H-indol-3-yloxy) propan-2-ol	Cytotoxic against cancer cell lines	[23]
Demospongiae/ Heteroscleromorpha	*Haliclona sp.*	Alkaloids	Haliclocyclamines A–C	Anti-mycobacterial	[24]
Demospongiae/ Heteroscleromorpha	*Haliclona sp.*	Alkaloids	Halichondriamine C (2)	Antimicrobial	[25]
Demospongiae/ Heteroscleromorpha	*Haliclona sp.*	Alkaloids	Njaoamine I	Cytotoxic against cancer cell lines	[26]
Demospongiae/ Heteroscleromorpha	*Haplosclerida sp.*	Alkaloids	Conicamin (2)	Cytotoxic against cancer cell lines	[27]
Demospongiae/ Heteroscleromorpha	*Hemimycale aff arabica*	Alkaloids	Cyclic urea derivatives	Antiglycated agents	[28]
Demospongiae/ Heteroscleromorpha	*Hemimycale arabica*	Alkaloids	Hemimycalins (3)	Antimicrobial	[29]
Demospongiae/ Heteroscleromorpha	*Iotrochota sp.*	Alkaloids	Enisorine A-E	Antimicrobial	[30]
Demospongiae/ Heteroscleromorpha	*Jaspis splendis*	Alkaloids	Jasplakinolides (5)	Cytotoxic against cancer cell lines	[31]

Demospongiae/ Heteroscleromorpha	*Latrunculia biformis*	Alkaloids	Discorhabdin D-L (3)	Cytotoxic against cancer cell lines	[5]
Demospongiae/ Heteroscleromorpha	*Lipastrotethya sp.*	Alkaloids	Dragmacidins G and H	Cytotoxic against cancer cell lines	[32]
Demospongiae/ Heteroscleromorpha	*Lissodendoryx florida*	Alkaloids	Lissodendoric acids A (1) and B	Antioxidant	[33]
Demospongiae/ Heteroscleromorpha	*Neopetrosia sp.*	Alkaloids	Neopetrosides A and B	Upregulate mitochondrial functions	[34]
Demospongiae/ Heteroscleromorpha	*Oceanapia sp.*	Alkaloids	Adenoside derivative	Cytotoxic against cancer cell lines	[35]
Demospongiae/ Heteroscleromorpha	*Echinoclathria gibbosa*	Lipids	Diverse lipids (3)	Cytotoxic against cancer cell lines	[36]
Demospongiae/ Heteroscleromorpha	*Haliclona fulva*	Lipids	Fulvyne A-I (9)	Antimicrobial	[37]
Demospongiae/ Heteroscleromorpha	*Haliclona sp.*	Lipids	Brominated acetylenic hydricarbon	Cytotoxic against cancer cell lines	[38]
Demospongiae/ Heteroscleromorpha	*Jaspis duoaster*	Lipids	Stellettazole D	Cytotoxic against cancer cell lines	[39]
Demospongiae/ Heteroscleromorpha	*Lipastrotethya sp.*	Lipids	Pourosides F-I (12)	Cytotoxic against cancer cell lines	[40]
Demospongiae/ Heteroscleromorpha	*Lipastrotethya sp.*	Lipids	Sarasinosides N-R	Cytotoxic against cancer cell lines	[41]
Demospongiae/ Heteroscleromorpha	*Niphates digitalis*	Lipids	Niphatenones A-B	Cytotoxic against cancer cell lines	[42]
Demospongiae/ Heteroscleromorpha	*Niphates sp.*	Lipids	Nepheliosyne B	Cytotoxic against cancer cell lines	[43]

Demospongiae/ Heteroscleromorpha	*Pandaros acanthifolium*	Lipids	Pandarosides E-J (	Anti-parasitic	[44]
Demospongiae/ Heteroscleromorpha	*Pandaros acanthifolium*	Lipids	Pandaroside K-M (5)	Anti-parasitic	[45]
Demospongiae/ Heteroscleromorpha	*Pandaros acanthifolium*	Lipids	Acanthifoliosides A-F (6)	Anti-parasitic	[46]
Demospongiae/ Heteroscleromorpha	*Pandaros acanthifolium*	Lipids	Acanthifoliosides G-J	Antioxidant	[47]
Demospongiae/ Heteroscleromorpha	*Penares sp.*	Lipids	Penasins A-E (5)	Cytotoxic against cancer cell lines	[48]
Demospongiae/ Heteroscleromorpha	*Erylus cf. deficiens*	Lipids	Erylusamide A–D	Inhibit indoleamine 2,3-dioxygenase (IDO)	[49]
Demospongiae/ Heteroscleromorpha	*Iotrochota purpurea*	Peptides	Porpuniones A-J (10)	Antimicrobial	[50]
Demospongiae/ Heteroscleromorpha	*Iotrochota sp.*	Peptides	Iotrochamides A-B (2)	Anti-parasitic	[51]
Demospongiae/ Heteroscleromorpha	*Ecionemia acervus*	Peptides	Stelletolide a	Cytotoxic against cancer cell lines	[52]
Demospongiae/ Heteroscleromorpha	*Geodia barretti*	Peptides	Geobarretin A-C	Anti-inflammatory	[53]
Demospongiae/ Heteroscleromorpha	*Inflatella coelosphaeroides*	Peptides	Friomaramide	Antiparasitic	[54]
Demospongiae/ Heteroscleromorpha	*Geodia sp.*	Polyketides	Franklinolides A-C (3)	Cytotoxic against cancer cell lines	[55]
Demospongiae/ Heteroscleromorpha	*Lithoplocamica lithistoides*	Polyketides	Polyketides amines (2)	Cytotoxic against cancer cell lines	[56]

Demospongiae/ Heteroscleromorpha	*Echinochalina bargibanti*	Polyketides	Arsenicins B and C	Antimicrobial	[57]
Demospongiae/ Heteroscleromorpha	*Geodia macandrewii*	Polyketides	Geodiataurine	Cytotoxic against cancer cell lines	[58]
Demospongiae/ Heteroscleromorpha	*Halichondria cf. panicea*	Polyketides	Isopetrosynol	Inhibits PTP-1B	[59]
Demospongiae/ Heteroscleromorpha	*Leiodermatium sp.*	Polyketides	Leiodermatolides B and C	Cytotoxic against cancer cell lines	[60]
Demospongiae/ Heteroscleromorpha	*Haliclona crassiloba*	Steroids	Halicrasterols (4)	Antimicrobial	[61]
Demospongiae/ Heteroscleromorpha	*Lissodendryx fibrosa*	Steroids	Manadosterols A-B (2)	Enzyme inhibition	[62]
Demospongiae/ Heteroscleromorpha	*Haliclona simulans*	Steroids	Steroids (2)	Anti-parasitic	[63]
Demospongiae/ Heteroscleromorpha	*Halicondria vansoesti*	Steroids	Steroids (4)	Reduce glucose uptake	[64]
Demospongiae/ Heteroscleromorpha	*Inflatella sp.*	Steroids	Oxysterols	Anti-Parkonsons disease	[65]
Demospongiae/ Heteroscleromorpha	*Halichondria sp.*	Terpenoids	Halichonadin G-I (3)	Cytotoxic against cancer cell lines	[66]
Demospongiae/ Heteroscleromorpha	*Haliclona sp.*	Terpenoids	Meroterpenoid (1)	Antioxidant	[67]
Demospongiae/ Heteroscleromorpha	*Haliclona sp.*	Terpenoids	Haliclonic acid A-B (2)	Enzyme inhibition	[68]
Demospongiae/ Heteroscleromorpha	*Halicondria okadai*	Terpenoids	Halichonines A-C (3)	Cytotoxic against cancer cell lines	[69]

Demospongiae/ Heteroscleromorpha	*Hamigera tarangaensis*	Terpenoids	Hamigerans (11)	Cytotoxic against cancer cell lines	[70]
Demospongiae/ Heteroscleromorpha	*Jaspis stellifera*	Terpenoids	Jaspiferin A-B	Cytotoxic against cancer cell lines	[71]
Demospongiae/ Heteroscleromorpha	*Leiodermatium sp.*	Terpenoids	Leiodermatolide	Cytotoxic against cancer cell lines	[72]
Demospongiae/ Heteroscleromorpha	*Pachastrissa nux*	Terpenoids	Kabiramides J-K (2)	Anti-malarial	[73]
Demospongiae/ Heteroscleromorpha	*Erylus goffrilleri*	Terpenoids	Erylosides	Cytotoxic against cancer cell lines	[74]
Demospongiae/ Heteroscleromorpha	*Halichondria sp.*	Terpenoids	Halichons	Cytotoxic against cancer cell lines	[75]
Demospongiae/ Heteroscleromorpha	*Haliclona sp.*	Terpenoids	Sesquiterpenoids	Anti-inflammatory	[76]
Demospongiae/ Heteroscleromorpha	*Haliclona sp.*	Terpenoids	Halioxepine B-D	Cytotoxic against cancer cell lines	[77]
Demospongiae/ Heteroscleromorpha	*Hamigera tarangaensis*	Terpenoids	Hamigerans (9)	Cytotoxic against cancer cell lines	[78]
Demospongiae/ Heteroscleromorpha	*Hayttella sp.*	Terpenoids	Spongins	Cytotoxic against cancer cell lines	[79]
Demospongiae/ Heteroscleromorpha	*Hymerhabdia sp.*	Terpenoids	Hymerhabdrin A	Antifouling against Balanus amphitrite	[80]
Demospongiae/ Keratosa	*Smenospongia cerebriformis*	Alkaloids	Dictazole A	Enzyme inhibition	[81]
Demospongiae/ Keratosa	*Smenospongia sp.*	Alkaloids	Indole alkaloids (8)	Cytotoxic against cancer cell lines	[82]

Demospongiae/ Keratosa	*Spongia sp.*	Alkaloids	Guanines (4)	Neuronal signal transmission	[83]
Demospongiae/ Keratosa	*Smenospongia sp.*	Alkaloids	Smenamide F-G (2)	Cytotoxic against cancer cell lines	[84]
Demospongiae/ Keratosa	*Spongia sp.*	Alkaloids	Indoles (2)	Enzyme inhibition	[85]
Demospongiae/ Keratosa	*Spongia sp.*	Lipids	Heterofibrin A1-A3 and B (6)	Detergent	[86]
Demospongiae/ Keratosa	*Spongiidae sp.*	Lipids	Taurospongins B and C	Antifungal	[87]
Demospongiae/ Keratosa	*Phyllospongia sp.*	Lipids	Phyllactone H	Cytotoxic against cancer cell lines	[88]
Demospongiae/ Keratosa	*Smenospongia aurea*	Polyketides	Smenothiazole A and B	Cytotoxic against cancer cell lines	[89]
Demospongiae/ Keratosa	*Smenospongia cerebriformis*	Polyketides	Smenocerone B	Cytotoxic against cancer cell lines	[90]
Demospongiae/ Keratosa	*Spongia sp.*	Polyketides	Sarasinoside M2	Cytotoxic against cancer cell lines	[91]
Demospongiae/ Keratosa	*Spongia sp.*	Polyketides	Langcoquinone C	Antibacterial	[92]
Demospongiae/ Keratosa	*Scalarispongia aqabaensis*	Steroids	Callysterol	Anti-inflammatory	[93]
Demospongiae/ Keratosa	*Scalarispongia sp.*	Terpenoids	Scalarene derivatives (4)	Cytotoxic against cancer cell lines	[94]
Demospongiae/ Keratosa	*Psammocinia sp.*	Terpenoids	Sesterterpene (5)	Anti-inflammatory	[95]
Demospongiae/ Keratosa	*Sarcotragus sp.*	Terpenoids	Sarcotin P	Cytotoxic against cancer cell lines	[96]
Demospongiae/ Keratosa	*Sarcotragus spinosulsus*	Terpenoids	Quinone	Cytotoxic against cancer cell lines	[97]
Demospongiae/ Keratosa	*Spongia officinalis*	Terpenoids	Afficinic acid A-B (2)	Induction of biofilm formation	[98]

Demospongiae/ Keratosa	*Spongia sp.*	Terpenoids	Meroterpenoids (5)	Enzyme inhibition	[99]
Demospongiae/ Keratosa	*Spongia sp.*	Terpenoids	Diterpene (1)	Inhibit sea urchin development	[100]
Demospongiae/ Keratosa	*Thorecta reticulata*	Terpenoids	Metachromin U-W	Cytotoxic against cancer cell lines	[101]
Demospongiae/ Keratosa	*Phyllospongia lamellosa*	Terpenoids	Sesterterpene (5)	Cytotoxic against cancer cell lines	[102]
Demospongiae/ Keratosa	*Phyllospongia sp.*	Terpenoids	Deaacetylphylloketal	Anti-inflammatory	[103]
Demospongiae/ Keratosa	*Sarcotragus sp.*	Terpenoids	Sesterterpene	Cytotoxic against cancer cell lines	[104]
Demospongiae/ Keratosa	*Scalarispongia sp.*	Terpenoids	Scalarane sesterterpenoids (1)	Cytotoxic against cancer cell lines	[105]
Demospongiae/ Keratosa	*Smenospongia cerebriformis*	Terpenoids	Smenohaimiens A–E	Inhibits NO-production	[106]
Demospongiae/ Keratosa	*Smenospongia sp.*	Terpenoids	Sesquiterpenoidss	Cytotoxic against cancer cell lines	[107]
Demospongiae/ Keratosa	*Spongia ceylonensis*	Terpenoids	Ceylonamides A−F	Inhibit RANKL-Induced Osteoclastogenesis	[108]
Demospongiae/ Keratosa	*Spongia ceylonensis*	Terpenoids	Ceylonins A–F	inhibit the formation of multinuclear osteoclasts	[109]
Demospongiae/ Keratosa	*Spongia officinalis*	Terpenoids	Diterpenes (5)	Anti-inflammatory	[110]
Demospongiae/ Keratosa	*Spongia sp.*	Terpenoids	Langcoquinone A-B	Antibacterial	[111]
Demospongiae/ Keratosa	*Spongia sp.*	Terpenoids	Langconols A–C	Antibacterial	[92]
Demospongiae/ Keratosa	*Spongia sp.*	Terpenoids	Sesquiterpene aminoquinones (3)	Antimicrobial	[112]
Demospongiae/ Keratosa	*Spongia sp.*	Terpenoids	Sclaraolides (4)	Cytotoxic against cancer cell lines	[113]

Demospongiae/ Keratosa	*Spongia sp.*	Terpenoids	Spongiain A-C (3)	Promotes cell proliferation	[114]
Demospongiae/ Keratosa	*Spongia sp.*	Terpenoids	Ceylonamide G	Cytotoxic against cancer cell lines	[115]
Demospongiae/ Keratosa	*Spongionella sp.*	Terpenoids	Cracilines (4)	Protect mitochondria from oxidative stress	[116]
Demospongiae/ Verongimorpha	*Suberea ianthelliformis*	Alkaloids	Ianthelliformisamines A-C	Antimicrobial	[117]
Demospongiae/ Verongimorpha	*Suberea ianthelliformis*	Alkaloids	Araplysillins (2)	Anti-malarial	[118]
Demospongiae/ Verongimorpha	*Suberea sp.*	Alkaloids	Psammaplysins (3)	Cytotoxic against cancer cell lines	[119]
Demospongiae/ Verongimorpha	*Suberea ianthelliformis*	Alkaloids	Brominated tyrosines (4)	Cytotoxic against cancer cell lines	[120]
Demospongiae/ Verongimorpha	*Suberea sp.*	Alkaloids	Bromotyrosines (2)	Cytotoxic against cancer cell lines	[121]
Demospongiae/ Verongimorpha	*Suberea sp.*	Alkaloids	Maedamines C-D (2)	Cytotoxic against cancer cell lines	[122]
Demospongiae/ Verongimorpha	*Suberea mollis*	Peptides	Subereamines A-B (2)	Antioxidant	[123]
Demospongiae/ Verongimorpha	*Suberea sp.*	Terpenoids	Phenols	Enzyme inhibition	[124]
Demospongiae/Heterosclermorpha	*Aaptos sp.*	Alkaloids	Aaptamine	Antimicrobial	[125]
Demospongiae/Heterosclermorpha	*Aaptos aaptos*	Alkaloids	Aaptamine derivatives (5)	Antifungal	[126]

Demospongiae/Heteroscler omorpha	*Aaptos aaptos*	Alkaloids	Aaptamine derivatives (9)	Cytotoxic against cancer cell lines	[127]
Demospongiae/Heteroscler omorpha	*Phoriospongia sp.*	Alkaloids	Adenosine alkaloids	Nematocidal	[128]
Demospongiae/Heteroscler omorpha	*Agelas sp.*	Alkaloids	Agelamides A and B	Antimicrobial	[129]
Demospongiae/Heteroscler omorpha	*Agelas sp.*	Alkaloids	Agelamides C-E	Antimicrobial	[130]
Demospongiae/Heteroscler omorpha	*Aaptos sp.*	Alkaloids	Aaptamines (3)	Cytotoxic against cancer cell lines	[131]
Demospongiae/Heteroscler omorpha	*Aaptos suberitoides*	Alkaloids	Aamptamine derivatives (4)	Cytotoxic against cancer cell lines	[132]
Demospongiae/Heteroscler omorpha	*Agelas clathrodes*	Alkaloids	Bisuracil	Cytotoxic against cancer cell lines	[133]
Demospongiae/Heteroscler omorpha	*Agelas linnaei*	Alkaloids	Alkaloids (11)	Cytotoxic against cancer cell lines	[134]
Demospongiae/Heteroscler omorpha	*Agelas mauritania*	Alkaloids	Bromopyrrole (1)	Antimicrobial	[135]
Demospongiae/Heteroscler omorpha	*Agelas sp.*	Alkaloids	Nagelamides U-Z (5)	Antifungal	[136]
Demospongiae/Heteroscler omorpha	*Amphimedon sp.*	Alkaloids	Pyrinodemins E-F	Cytotoxic against cancer cell lines	[137]
Demospongiae/Heteroscler omorpha	*Amphimedon sp.*	Alkaloids	Alkaloids (2)	Antimicrobial	[138]
Demospongiae/Heteroscler omorpha	*Amphimedon sp.*	Alkaloids	Pyrinodenins G-I (3)	Cytotoxic against cancer cell lines	[139]

Demospongiae/Heteroscleromorpha	*Asteropus sp.*	Alkaloids	Catechol	Antimicrobial	[140]
Demospongiae/Heteroscleromorpha	*Biemna sp.*	Alkaloids	Amines (2)	Cytotoxic against cancer cell lines	[141]
Demospongiae/Heteroscleromorpha	*Clathrina clathrus*	Alkaloids	Alkaloids	Antimicrobial	[142]
Demospongiae/Heteroscleromorpha	*Crambe crambe*	Alkaloids	Crambescins (11)	Cytotoxic against cancer cell lines	[143]
Demospongiae/Heteroscleromorpha	*Monanchora pulchra*	Alkaloids	Guanidine alkaloids	Cytotoxic against cancer cell lines	[144]
Demospongiae/Heteroscleromorpha	*Monanchora pulchra*	Alkaloids	Monanchomycalins A-B (2)	Cytotoxic against cancer cell lines	[145]
Demospongiae/Heteroscleromorpha	*Monanchora pulchra*	Alkaloids	Pulchranins A-C (3)	Neuronal signal transmission	[146]
Demospongiae/Heteroscleromorpha	*Monanchora pulchra*	Alkaloids	Monanchomycalin C	Cytotoxic against cancer cell lines	[147]
Demospongiae/Heteroscleromorpha	*Neopetrosia proxima*	Alkaloids	Neopetrosiamine A	Antimicrobial	[148]
Demospongiae/Heteroscleromorpha	*Petrosia sp.*	Alkaloids	Amines (2)	Anti-parasitic	[149]
Demospongiae/Heteroscleromorpha	*Phorbas topsenti*	Alkaloids	Aminoimidazolines (3)	Antioxidant	[150]
Demospongiae/Heteroscleromorpha	*Sceptrella sp.*	Alkaloids	Alkaloids (2)	Enzyme inhibition	[151]
Demospongiae/Heteroscleromorpha	*Stelletta sp.*	Alkaloids	Diketipiperazine	Cytotoxic against cancer cell lines	[152]

Demospongiae/Heteroscleromorpha	*Stylissa sp.*	Alkaloids	Alkaloids (4)	Cytotoxic against cancer cell lines	[153]
Demospongiae/Heteroscleromorpha	*Suberites sp.*	Alkaloids	Nakijinamines C-E (3)	Antimicrobial	[154]
Demospongiae/Heteroscleromorpha	*Suberites sp.*	Alkaloids	Nakijinamines (6)	Antimicrobial	[155]
Demospongiae/Heteroscleromorpha	*Theonella swinhoei*	Alkaloids	Aurantoside J	Cytotoxic against cancer cell lines	[156]
Demospongiae/Heteroscleromorpha	*Theonella swinhoei*	Alkaloids	Solmonoamide A	Anti-inflammatory	[157]
Demospongiae/Heteroscleromorpha	*Xestospongia sp.*	Alkaloids	Norsalsonlinol (1)	Antioxidant	[158]
Demospongiae/Heteroscleromorpha	*Zyzzya sp.*	Alkaloids	Tsitsikammamine C	Anti-malarial	[159]
Demospongiae/Heteroscleromorpha	*Aaptos aaptos*	Alkaloids	Aaptamines (3)	Cytotoxic against cancer cell lines	[160]
Demospongiae/Heteroscleromorpha	*Aaptos aaptos*	Alkaloids	Aaptamine derivatives (2)	Cytotoxic against cancer cell lines	[161]
Demospongiae/Heteroscleromorpha	*Acanthodendrilla sp.*	Alkaloids	Acanthodendrilline	Cytotoxic against cancer cell lines	[162]
Demospongiae/Heteroscleromorpha	*Acanthostrongylophora ingens*	Alkaloids	Chloromethylhalicyclamine B	Protein Kinase CK1δ/ε Inhibitor	[163]
Demospongiae/Heteroscleromorpha	*Acanthostrongylophora ingens*	Alkaloids	Ingenines C and D	Cytotoxic against cancer cell lines	[164]
Demospongiae/Heteroscleromorpha	*Acanthostrongylophora ingens*	Alkaloids	Ingenine E	Cytotoxic against cancer cell lines	[165]

Demospongiae/Heteroscleromorpha	*Acanthostrongylophora ingens*	Alkaloids	Ingenine F	Cytotoxic against cancer cell lines	[166]
Demospongiae/Heteroscleromorpha	*Acanthostrongylophora ingens*	Alkaloids	Tetradehydrohalicyclamine B	Proteasome inhibitor	[167]
Demospongiae/Heteroscleromorpha	*Acanthostrongylophora sp.*	Alkaloids	Kepulauamine A-E	Weak cytotoxic and antibacterial	[168]
Demospongiae/Heteroscleromorpha	*Agelas citrina*	Alkaloids	Citrinamines A-D (5)	Antimicrobial	[169]
Demospongiae/Heteroscleromorpha	*Agelas nakamurai*	Alkaloids	Nakamurines B	Antifungal	[170]
Demospongiae/Heteroscleromorpha	*Agelas nakamurai*	Alkaloids	Isoagelasine C	Antifungal Antibacterial	[171]
Demospongiae/Heteroscleromorpha	*Agelas nakamurai*	Alkaloids	Isoagelasine B	Antifungal	[171]
Demospongiae/Heteroscleromorpha	*Agelas nemoechinata*	Alkaloids	Nemoechine G	Antibacterial	[172]
Demospongiae/Heteroscleromorpha	*Agelas nemoechinata*	Alkaloids	Nemoechine D	Cytotoxic against cancer cell lines	[173]
Demospongiae/Heteroscleromorpha	*Agelas nemoechinata*	Alkaloids	Dibromopyrrole alkaloids (3)	Cytotoxic against cancer cell lines	[174]
Demospongiae/Heteroscleromorpha	*Agelas nemoechinata*	Alkaloids	Agelanemoechine	Pro-angiogenic	[175]
Demospongiae/Heteroscleromorpha	*Agelas sp.*	Alkaloids	Longamides	Antifungal	[176]
Demospongiae/Heteroscleromorpha	*Agelas sp.*	Alkaloids	Hexazosceptrin	Antimicrobial	[177]

Demospongiae/Heteroscleromorpha	*Agleas kosrae*	Alkaloids	Sceptrin derivatives (2)	Cytotoxic against cancer cell lines	[178]
Demospongiae/Heteroscleromorpha	*Amphimedon sp.*	Alkaloids	Zamamidine D	Antibacterial Antifungal	[179]
Demospongiae/Heteroscleromorpha	*Biemna laboutei*	Alkaloids	Netamines O-S	Anti-malarial	[180]
Demospongiae/Heteroscleromorpha	*Callyspongia siphonella*	Alkaloids	Oxindole alkaloidss (2)	Antimicrobial	[181]
Demospongiae/Heteroscleromorpha	*Callyspongia sp.*	Alkaloids	Callyazepin	Cytotoxic against cancer cell lines	[182]
Demospongiae/Heteroscleromorpha	*Characella sp.*	Alkaloids	Poecillastrin H	Cytotoxic against cancer cell lines	[183]
Demospongiae/Heteroscleromorpha	*Cinachyrella sp.*	Alkaloids	Cinachylenic acid B-D	Cytotoxic against cancer cell lines	[184]
Demospongiae/Heteroscleromorpha	*Clathria bulbotaxa*	Alkaloids	Crambescidin-derivatives (3)	Cytotoxic against cancer cell lines	[185]
Demospongiae/Heteroscleromorpha	*Crambe crambe symbiosis*	Alkaloids	Crambescidin-derivatives (2)	Cytotoxic against cancer cell lines	[186]
Demospongiae/Heteroscleromorpha	*Dictyonella sp.*	Alkaloids	Bromopyrroles (3)	Yeast proteasome inhibition	[187]
Demospongiae/Heteroscleromorpha	*Dragmacidon sp.*	Alkaloids	Dragmacidins I and J	Enzyme inhibitors	[188]
Demospongiae/Heteroscleromorpha	*Monanchora arbuscula*	Alkaloids	Pyrimidines (6)	Anti-parasitic	[189]
Demospongiae/Heteroscleromorpha	*Monanchora pulchra*	Alkaloids	Monanchoxymycalin A-B	Cytotoxic against cancer cell lines	[190]

Demospongiae/Heteroscleromorpha	*Monanchora pulchra*	Alkaloids	Spongins	Cytotoxic against cancer cell lines	[191]
Demospongiae/Heteroscleromorpha	*Monanchora pulchra*	Alkaloids	Monanchoxymycalin C	Cytotoxic against cancer cell lines	[192]
Demospongiae/Heteroscleromorpha	*Monanchora sp.*	Alkaloids	Monachoradin a-c crambecidin	Cytotoxic against cancer cell lines	[193]
Demospongiae/Heteroscleromorpha	*Monanchora unguiculata*	Alkaloids	Ptilomycalin E and F	Antibacterial Anti-protozoal	[194]
Demospongiae/Heteroscleromorpha	*Mycale hentscheli*	Alkaloids	Peloruside E	Cytotoxic against cancer cell lines	[195]
Demospongiae/Heteroscleromorpha	*Mycale lissochela*	Alkaloids	Mycalenitrile 15	Inhibits PTP-1B	[196]
Demospongiae/Heteroscleromorpha	*Neopetrosia exigua*	Alkaloids	Neopetrocyclamines A and B	Cytotoxic against cancer cell lines	[197]
Demospongiae/Heteroscleromorpha	*Poecillastra sp.*	Alkaloids	Poecillastrin E, F, and G	Cytotoxic against cancer cell lines	[198]
Demospongiae/Heteroscleromorpha	*Pseudaxinella reticulata*	Alkaloids	Crambescin homologues (4)	Antifungal	[199]
Demospongiae/Heteroscleromorpha	*Spongosorites calcicola*	Alkaloids	Bisindole alkaloidss (2)	Cytotoxic against cancer cell lines	[200]
Demospongiae/Heteroscleromorpha	*Stylissa sp.*	Alkaloids	Dispaceamide E (2)	Enzyme inhibition	[201]
Demospongiae/Heteroscleromorpha	*Tedania brasilliensis*	Alkaloids	Pseudoceratidine (5)	Active against Leishmania	[202]
Demospongiae/Heteroscleromorpha	*Topsentia sp.*	Alkaloids	Tulongicin A	Antibacterial	[203]

Demospongiae/Heteroscleromorpha	*Topsentia sp.*	Alkaloids	Dihydrospongotine C	Antibacterial	[203]
Demospongiae/Heteroscleromorpha	*Tsitsikamma favus*	Alkaloids	Makaluvamine Q	Cytotoxic against cancer cell lines	[204]
Demospongiae/Heteroscleromorpha	*Xestospongia muta*	Alkaloids	Araguspongine C	Cytotoxic against cancer cell lines	[205]
Demospongiae/Heteroscleromorpha	*Xestospongia sp.*	Alkaloids	N-methylniphatyne a	Cytotoxic against cancer cell lines	[206]
Demospongiae/Heteroscleromorpha	*Agelas sp.*	Alkaloids	Bromopyrroles (5)	Antimicrobial	[207]
Demospongiae/Heteroscleromorpha	*Dragmacidon coccinea*	Alkaloids	Dragmacidoside	Anti-inflammatory	[208]
Demospongiae/Heteroscleromorpha	*Acanthella cavernosa*	Alkaloids	Guanidine alkaloids	Tumor suppressor	[209]
Demospongiae/Heteroscleromorpha	*Callyspongia sp.*	Alkaloids	Macrolide	Cytotoxic against cancer cell lines	[210]
Demospongiae/Heteroscleromorpha	*Acanthostrongylophora ingens*	Alkaloids	Manzamines (2)	Proteasome inhibition	[211]
Demospongiae/Heteroscleromorpha	*Biemna laboutei*	Alkaloids	Neatamines H-N	Anti-malarial	[212]
Demospongiae/Heteroscleromorpha	*Ancorina Geodides*	Alkaloids	Pyridoacridines (2)	Cytotoxic against cancer cell lines	[213]
Demospongiae/Heteroscleromorpha	*Topsentia sp.*	Alkaloids	Topsendines A-F	Voltage gated ion-channels	[214]
Demospongiae/Heteroscleromorpha	*Monanchora pulchra*	Alkaloids	Urupocidin A	Anti-inflammatory	[215]

Demospongiae/Heteroscleromorpha	*Xestospongia testudinaria*	Lipids	Brominated pufas (8)	Enzyme inhibition	[216]
Demospongiae/Heteroscleromorpha	*Siphonochalina siphonella*	Lipids	Callyspongenols (2)	Cytotoxic against cancer cell lines	[217]
Demospongiae/Heteroscleromorpha	*Cinachyrella sp.*	Lipids	Cinanthrenol A	Cytotoxic against cancer cell lines	[218]
Demospongiae/Heteroscleromorpha	*Axinyssa djiferi*	Lipids	Axidjiferoside A-C (3)	Anti-malarial	[219]
Demospongiae/Heteroscleromorpha	*Crella spinulata*	Lipids	Shishicrellastatin A-B (2)	Enzyme inhibition	[220]
Demospongiae/Heteroscleromorpha	*Dasychalina sp.*	Lipids	Desulfhaplosamate	Receptor activator	[221]
Demospongiae/Heteroscleromorpha	*Monanchora sp.*	Lipids	Phorboketals A-K	Cytotoxic against cancer cell lines	[222]
Demospongiae/Heteroscleromorpha	*Mycale acerata*	Lipids	Fatty acid	Cytotoxic against cancer cell lines	[223]
Demospongiae/Heteroscleromorpha	*Myrmekioderma dendyi*	Lipids	Myrmecoside E	Cytotoxic against cancer cell lines	[224]
Demospongiae/Heteroscleromorpha	*Perosia solida sp.*	Lipids	Petroacetylene	Cytotoxic against cancer cell lines	[225]
Demospongiae/Heteroscleromorpha	*Petrosia sp.*	Lipids	Polyacetylenes (6)	Cytotoxic against cancer cell lines	[226]
Demospongiae/Heteroscleromorpha	*Petrosia sp.*	Lipids	Miyakosyne A-F (6)	Cytotoxic against cancer cell lines	[227]
Demospongiae/Heteroscleromorpha	*Petrosia sp.*	Lipids	Petrosiacetylene E	Cytotoxic against cancer cell lines	[228]

Demospongiae/Heteroscleromorpha	*Petrosia sp.*	Lipids	Petrosynoic acid A-D (4)	Cytotoxic against cancer cell lines	[229]
Demospongiae/Heteroscleromorpha	*Petrosia strongylata*	Lipids	Petrosiol A-E (5)	Neuronal differentiation	[230]
Demospongiae/Heteroscleromorpha	*Placospongia sp.*	Lipids	Phosphate conatining alkynes (2)	Receptor activator	[231]
Demospongiae/Heteroscleromorpha	*Rhabdastrella globostellata*	Lipids	Globostellins K-L (2)	Enzyme inhibition	[232]
Demospongiae/Heteroscleromorpha	*Spirastrella abata*	Lipids	Sphingolipids (3)	Cytotoxic against cancer cell lines	[233]
Demospongiae/Heteroscleromorpha	*Spirastrella mollis*	Lipids	Mollenyne	Cytotoxic against cancer cell lines	[234]
Demospongiae/Heteroscleromorpha	*Suberites japonicus*	Lipids	Iodinated acetylenic acids (4)	Anti-inflammatory	[235]
Demospongiae/Heteroscleromorpha	*Terpios sp.*	Lipids	Terposides	Anti-inflammatory	[236]
Demospongiae/Heteroscleromorpha	*Theonella swinhoei*	Lipids	Bromotheonyic acid	Cytotoxic against cancer cell lines	[237]
Demospongiae/Heteroscleromorpha	*Topsentia sp.*	Lipids	Topsentinols (3)	Enzyme inhibition	[238]
Demospongiae/Heteroscleromorpha	*Xestospongia sp.*	Lipids	Glycolipids	Antimicrobial	[239]
Demospongiae/Heteroscleromorpha	*Xestospongia testudinaria*	Lipids	Mutafuran H	Enzyme inhibition	[240]
Demospongiae/Heteroscleromorpha	*Xestospongia testudinaria*	Lipids	Brominatet fatty acids (5)	Adipogenesis	[241]

Demospongiae/Heteroscleromorpha	*Placospongia sp.*	Lipids	Placotylene A-B	Receptor activator	[242]
Demospongiae/Heteroscleromorpha	*Callyspongia sp.*	Lipids	Polyacetylenes (2)	Cytotoxic against cancer cell lines	[243]
Demospongiae/Heteroscleromorpha	*Petrosia sp.*	Lipids	Polyacetylenes (5)	Cytotoxic against cancer cell lines	[244]
Demospongiae/Heteroscleromorpha	*Petrosia sp.*	Lipids	Sphingolipids (3)	Cytotoxic against cancer cell lines	[245]
Demospongiae/Heteroscleromorpha	*Asteropus niger*	Lipids	(2R,5Z,9Z)-2-methoxy-25-methyl-5,9-hexacosadienoic acid	Inhibitor of Topoisomerases IB	[246]
Demospongiae/Heteroscleromorpha	*Callyspongia cf. californica*	Lipids	Callyspongidic acids	Cytotoxic against cancer cell lines	[247]
Demospongiae/Heteroscleromorpha	*Desmapsamma anchorata*	Lipids	Ceramides (14)	Cytotoxic against cancer cell lines	[248]
Demospongiae/Heteroscleromorpha	*Dictyonella sp.*	Lipids	Dictyoneolone	Cytotoxic against cancer cell lines	[249]
Demospongiae/Heteroscleromorpha	*Discodermia kiiensis*	Lipids	Sulfolipodiscamides A–C	Cytotoxic against cancer cell lines	[250]
Demospongiae/Heteroscleromorpha	*Melonanchora kobjakovae*	Lipids	Melonoside A	Induce autophagy	[251]
Demospongiae/Heteroscleromorpha	*Melonanchora kobjakovae*	Lipids	Fatty acid amides	Transcription inhibitor	[252]
Demospongiae/Heteroscleromorpha	*Monanchora clathrata*	Lipids	Ceramides	Cytotoxic against cancer cell lines	[253]
Demospongiae/Heteroscleromorpha	*Mycale sp.*	Lipids	Albanitriles A-G (7)	Antiprotozoal	[254]

Demospongiae/Heteroscler omorpha	*Myxilla incrustans*	Lipids	Myxillin A and C	Anti-inflammatory	[255]
Demospongiae/Heteroscler omorpha	*Negombata sp.*	Lipids	Ceramides (5)	Antoconvulsant	[256]
Demospongiae/Heteroscler omorpha	*Pleroma sp.*	Lipids	Yakushynols (6)	Cytotoxic against cancer cell lines	[257]
Demospongiae/Heteroscler omorpha	*Poecillastra compressa*	Lipids	Poecillastrosides D and E	Antifungal	[258]
Demospongiae/Heteroscler omorpha	*Spheciospongia vagabunda*	Lipids	Ceramides (3)	Cytotoxic against cancer cell lines	[259]
Demospongiae/Heteroscler omorpha	*Spirastrella purpurea*	Lipids	Lysophospholipidss (4)	Antifungal	[260]
Demospongiae/Heteroscler omorpha	*Stelletta sp.*	Lipids	Glycosylated fatty acids (6)	Cytotoxic against cancer cell lines	[261]
Demospongiae/Heteroscler omorpha	*Theonella mirabilis*	Lipids	Mirabolides A and B	Cytotoxic against cancer cell lines	[262]
Demospongiae/Heteroscler omorpha	*Theonella swinhoei*	Lipids	Yakushinamides A and B	HDAC inhibitor	[263]
Demospongiae/Heteroscler omorpha	*Xestospongia sp.*	Lipids	Polyacetylene derivatives (2)	Antifungal	[264]
Demospongiae/Heteroscler omorpha	*Xestospongia testudinaria*	Lipids	Brominated Lipidss	Enzyme inhibition	[265]
Demospongiae/Heteroscler omorpha	*Xestospongia testudinaria*	Lipids	Xestonariene J	Cytotoxic against cancer cell lines	[266]
Demospongiae/Heteroscler omorpha	*Suberites waedoensis*	Peptides	Cyclic peptides (2)	Cytotoxic against cancer cell lines	[267]

Demospongiae/Heteroscleromorpha	*Discodermia kiiensis*	Peptides	Lipodesipeptides (3)	Cytotoxic against cancer cell lines	[268]
Demospongiae/Heteroscleromorpha	*Ancorina sp.*	Peptides	Peptides	Anti-malarial	[269]
Demospongiae/Heteroscleromorpha	*Callyspongia aerizusa*	Peptides	Callyaerins A-F (6)	Antifungal	[270]
Demospongiae/Heteroscleromorpha	*Ceratopsion sp.*	Peptides	Yakuamide A-B (2)	Cytotoxic against cancer cell lines	[271]
Demospongiae/Heteroscleromorpha	*Clathria araiosa*	Peptides	Araisoamine A-D	Cytotoxic against cancer cell lines	[272]
Demospongiae/Heteroscleromorpha	*Clathria gombawuiensis*	Peptides	Gombamide A	Cytotoxic against cancer cell lines	[273]
Demospongiae/Heteroscleromorpha	*Discodermia calyx*	Peptides	Calyxamides A-B (2)	Cytotoxic against cancer cell lines	[274]
Demospongiae/Heteroscleromorpha	*Melophlus*	Peptides	Papuamides E-F (2)	Cytotoxic against brine shrimp	[275]
Demospongiae/Heteroscleromorpha	*Neamphius huxleyi*	Peptides	Neamphamines (3)	Cytotoxic against cancer cell lines	[276]
Demospongiae/Heteroscleromorpha	*Neamphius sp.*	Peptides	Nemphamide B	Antimicrobial	[277]
Demospongiae/Heteroscleromorpha	*Phakellia fusca*	Peptides	Phakellistatins 15-18 (4)	Cytotoxic against cancer cell lines	[278]
Demospongiae/Heteroscleromorpha	*Pipestela candelabra*	Peptides	Pipestelides A-C	Cytotoxic against cancer cell lines	[279]
Demospongiae/Heteroscleromorpha	*Siliquariaspongia mirabilis*	Peptides	Namalide A	Enzyme inhibition	[280]

Demospongiae/Heteroscleromorpha	*Stelletta clavosa*	Peptides	Mirabamides E-H	Anti-viral	[281]
Demospongiae/Heteroscleromorpha	*Stylissa massa*	Peptides	Styllisin A	Anti-inflammatory	[282]
Demospongiae/Heteroscleromorpha	*Stylissa sp.*	Peptides	Stylissamide X	Cytotoxic against cancer cell lines	[283]
Demospongiae/Heteroscleromorpha	*Theonella swinhoei*	Peptides	Koshikamides F-H (3)	Anti-viral	[284]
Demospongiae/Heteroscleromorpha	*Theonella swinhoei*	Peptides	Perthamides G-K (5)	Anti-inflammatory	[285]
Demospongiae/Heteroscleromorpha	*Theonella swinhoei*	Peptides	Peptides (2)	Cytotoxic against cancer cell lines	[286]
Demospongiae/Heteroscleromorpha	*Pipestela candelabra*	Peptides	Peptides (4)	Cytotoxic against cancer cell lines	[287]
Demospongiae/Heteroscleromorpha	*Reniochalina*	Peptides	Reniochalistatins A-E	Cytotoxic against cancer cell lines	[288]
Demospongiae/Heteroscleromorpha	*Stylissa caribica*	Peptides	Stylissamides G and H	Cytotoxic against cancer cell lines	[289]
Demospongiae/Heteroscleromorpha	*Theonella swinhoei*	Peptides	Theonellamide G	Antifungal	[290]
Demospongiae/Heteroscleromorpha	*Asteropus sp.*	Peptides	Callipeltins N and O	Cytotoxic against cancer cell lines	[291]
Demospongiae/Heteroscleromorpha	*Callyspongia*	Peptides	Callyptide A	Cytotoxic against cancer cell lines	[292]
Demospongiae/Heteroscleromorpha	*Callyspongia aerizusa*	Peptides	Callyaerin I-M	Cytotoxic against cancer cell lines	[293]

Demospongiae/Heteroscler omorpha	*Callyspongia sp.*	Peptides	Callyspongiamides A and B	Enzyme inhibitors	[294]
Demospongiae/Heteroscler omorpha	*Characella pachastrelloides*	Peptides	Characellides A-D (4)	Anti-inflammatory	[295]
Demospongiae/Heteroscler omorpha	*Clathria basilana*	Peptides	Microcionamides C and D	Cytotoxic against cancer cell lines	[296]
Demospongiae/Heteroscler omorpha	*Cribrochalina sp.*	Peptides	Pembamide	Cytotoxic against cancer cell lines	[297]
Demospongiae/Heteroscler omorpha	*Daedalopelta sp.*	Peptides	Daedophamide	Cytotoxic against cancer cell lines	[298]
Demospongiae/Heteroscler omorpha	*Discodermia*	Peptides	Stellatolide H	Cytotoxic against cancer cell lines	[299]
Demospongiae/Heteroscler omorpha	*Petrosia sp.*	Peptides	Halicylindramide F	Farnesoid X Receptor Antagonist	[300]
Demospongiae/Heteroscler omorpha	*Phakellia fusca*	Peptides	Fuscasins A-D	Cytotoxic against cancer cell lines	[301]
Demospongiae/Heteroscler omorpha	*Stelletta sp.*	Peptides	Stellettapeptins A and B	HIV-inhibition	[302]
Demospongiae/Heteroscler omorpha	*Stylissa carteri*	Peptides	Carteritin A	Cytotoxic against cancer cell lines	[303]
Demospongiae/Heteroscler omorpha	*Stylissa flabelliformis*	Peptides	Cyclopeptidess (3)	Cytotoxic against cancer cell lines	[304]
Demospongiae/Heteroscler omorpha	*Stylissa massa*	Peptides	Stylissatins B–D	Cytotoxic against cancer cell lines	[305]
Demospongiae/Heteroscler omorpha	*Theonella swinhoei*	Peptides	Nazumazoles A-C	Cytotoxic against cancer cell lines	[306]

Demospongiae/Heteroscleromorpha	*Theonella swinhoei*	Peptides	Nazumazoles D–F	Inhibits chemotrypsin	[307]
Demospongiae/Heteroscleromorpha	*Theonella swinhoei*	Peptides	Cyclotheonellazoles A–C	Protease inhibitors	[308]
Demospongiae/Heteroscleromorpha	*Cinachyrella enigmata*	Polyketides	Enigmazoles (3)	Cytotoxic against cancer cell lines	[309]
Demospongiae/Heteroscleromorpha	*Mycale hentscheli*	Polyketides	Peloruside B	Cytotoxic against cancer cell lines	[310]
Demospongiae/Heteroscleromorpha	*Agelas nemoechinata*	Polyketides	Nemoechioxide A	Cytotoxic against cancer cell lines	[173]
Demospongiae/Heteroscleromorpha	*Agelas sp.*	Polyketides	Nemoechine D	Cytotoxic against cancer cell lines	[173]
Demospongiae/Heteroscleromorpha	*Callyspongia sp.*	Polyketides	Polyacetylenic alcohol	Cytotoxic against cancer cell lines	[311]
Demospongiae/Heteroscleromorpha	*Petrosia alfiani*	Polyketides	Petroquinones	Cytotoxic against cancer cell lines	[312]
Demospongiae/Heteroscleromorpha	*Petrosia sp.*	Polyketides	Phormidolides B and C	Cytotoxic against cancer cell lines	[313]
Demospongiae/Heteroscleromorpha	*Petrosia sp. and Halicondria*	Polyketides	Polyacetylenic alcohols (6)	Cytotoxic against cancer cell lines	[314]
Demospongiae/Heteroscleromorpha	*Theonella sp.*	Polyketides	Lanesoic acid	Cytotoxic against cancer cell lines	[315]
Demospongiae/Heteroscleromorpha	*Xestospongia sp.*	Polyketides	Renieramycin O	Cytotoxic against cancer cell lines	[316]
Demospongiae/Heteroscleromorpha	*Callyspongia fibrosa*	Steroids	Sterols (4)	Anti-parasitic	[317]

Demospongiae/Heteroscleromorpha	*Phorbas gukhulensis*	Steroids	Aminosterols (2)	Cytotoxic against cancer cell lines	[318]
Demospongiae/Heteroscleromorpha	*Theonella swinhoei*	Steroids	Solomonsterols A-B (2)	Receptor activator	[319]
Demospongiae/Heteroscleromorpha	*Theonella swinhoei*	Steroids	Theonellasterols B-H (7)	Receptor activator	[320]
Demospongiae/Heteroscleromorpha	*Theonella swinhoei*	Steroids	Malaitasterol A	Receptor activator	[321]
Demospongiae/Heteroscleromorpha	*Theonella swinhoei*	Steroids	Conicasterol E	Receptor activator	[322]
Demospongiae/Heteroscleromorpha	*Theonella swinhoei*	Steroids	Conicasterol F	Receptor activator	[323]
Demospongiae/Heteroscleromorpha	*Theonella swinhoei*	Steroids	Conicasterol G-K	Receptor activator	[324]
Demospongiae/Heteroscleromorpha	*Theonella swinhoei*	Steroids	Theonellasterol K	Cytotoxic against cancer cell lines	[325]
Demospongiae/Heteroscleromorpha	*Xestospongia testudinaria*	Steroids	Sterols (3)	Antifouling	[326]
Demospongiae/Heteroscleromorpha	*Theonella sp.*	Steroids	Steroids (4)	Receptor activator	[327]
Demospongiae/Heteroscleromorpha	*Theonella swinhoei*	Steroids	Swinhoeisterols A and B	Cytotoxic against cancer cell lines	[328]
Demospongiae/Heteroscleromorpha	*Biemna ehrenbergi*	Steroids	Ehrenasterol and biemnic acid	Antimicrobial	[329]
Demospongiae/Heteroscleromorpha	*Callyspongia impexa*	Steroids	Gelliusterol E and callimplexen A	Antimicrobial	[330]

Demospongiae/Heteroscleromorpha	*Clathria gombawuiensis*	Steroids	Gombasterols A–F	Antidiabetic	[331]
Demospongiae/Heteroscleromorpha	*Monanchora sp.*	Steroids	Sterols (3)	Cytotoxic against cancer cell lines	[332]
Demospongiae/Heteroscleromorpha	*Monanchora sp.*	Steroids	Monanchosterols A and B	Anti-inflammatory	[333]
Demospongiae/Heteroscleromorpha	*Petrosia sp.*	Steroids	Sterols	Cytotoxic against cancer cell lines	[334]
Demospongiae/Heteroscleromorpha	*Polymastia boletiformis*	Steroids	Steroids (2)	Antifungal	[335]
Demospongiae/Heteroscleromorpha	*Theonella swinhoei*	Steroids	Sterols (3)	Cytotoxic against cancer cell lines	[336]
Demospongiae/Heteroscleromorpha	*Theonella swinhoei*	Steroids	Swinhoeisterols C–F	Enzyme inhibitors	[337]
Demospongiae/Heteroscleromorpha	*Topsentia sp.*	Steroids	Topsensterols	Antimicrobial	[338]
Demospongiae/Heteroscleromorpha	*Topsentia sp.*	Steroids	Topsensterol (2)	Cytotoxic against cancer cell lines	[339]
Demospongiae/Heteroscleromorpha	*Xestospongia sp.*	Steroids	Aragusterol J	Cytotoxic against cancer cell lines	[340]
Demospongiae/Heteroscleromorpha	*Xestospongia testudinaria*	Steroids	Steroid	Enzyme inhibitors	[341]
Demospongiae/Heteroscleromorpha	*Xestospongia testudinaria*	Steroids	Xestosterol	Cytotoxic against cancer cell lines	[342]
Demospongiae/Heteroscleromorpha	*Asteropus sp.*	Steroids	Asteropsin A	Neuronal signal transmission	[343]

Demospongiae/Heteroscleromorpha	*Axinyssa sp.*	Terpenoids	Axinyssins A-L (12)	Antimicrobial	[344]
Demospongiae/Heteroscleromorpha	*Callyspongia sp.*	Terpenoids	Bromotyrosine drivatives (3)	Cardiovascular disease	[345]
Demospongiae/Heteroscleromorpha	*Diacarnus megaspinorhabosa*	Terpenoids	Dicarnaperoxides (5)	Anti-malarial	[346]
Demospongiae/Heteroscleromorpha	*Clathria gombawuiensis*	Terpenoids	Gombaspiroketals A-C	Antimicrobial	[347]
Demospongiae/Heteroscleromorpha	*Siphonochalina siphonella*	Terpenoids	Neviotine C	Cytotoxic against cancer cell lines	[348]
Demospongiae/Heteroscleromorpha	*Phorbas sp.*	Terpenoids	Phorbaketals L-N (3)	Cytotoxic against cancer cell lines	[349]
Demospongiae/Heteroscleromorpha	*Xestospongia sp.*	Terpenoids	Quinone	Proteasome inhibition	[350]
Demospongiae/Heteroscleromorpha	*Aka coralliphagum*	Terpenoids	Sesquiterpene hydroquinones (4)	Cytotoxic against cancer cell lines	[351]
Demospongiae/Heteroscleromorpha	*Topsentia sp.*	Terpenoids	Terpenes (4)	Antifungal	[352]
Demospongiae/Heteroscleromorpha	*Axinyssa sp.*	Terpenoids	Terpenes (5)	Cytotoxic against cancer cell lines	[353]
Demospongiae/Heteroscleromorpha	*Acanthella cavernosa*	Terpenoids	Terpenoids (7)	Cytotoxic against cancer cell lines	[354]
Demospongiae/Heteroscleromorpha	*Acanthella cavernosa*	Terpenoids	Kalihinols M-T (8)	Antifouling	[355]
Demospongiae/Heteroscleromorpha	*Agelas axifera*	Terpenoids	Axistatin 1-3 (3)	Antimicrobial	[356]

Demospongiae/Heterosclerorpha	*Agelas citrina*	Terpenoids	Agelasidines E-F (2)	Antifungal	[357]
Demospongiae/Heteroscleromorpha	*Agelas sp.*	Terpenoids	Agelasines O-U	Cytotoxic against cancer cell lines	[358]
Demospongiae/Heteroscleromorpha	*Aka coralliphagum*	Terpenoids	Sulfated compounds (2)	Antioxidant	[359]
Demospongiae/Heteroscleromorpha	*Auletta sp.*	Terpenoids	Jasplakinolides (2)	Cytotoxic against cancer cell lines	[360]
Demospongiae/Heteroscleromorpha	*Diacarnus erythraeanus*	Terpenoids	Cyclic peroxides (2)	Cytotoxic against cancer cell lines	[361]
Demospongiae/Heteroscleromorpha	*Diacarnus megaspinorhabosa*	Terpenoids	Dicarperoxide S	Cytotoxic against cancer cell lines	[362]
Demospongiae/Heteroscleromorpha	*Melophlus*	Terpenoids	Aurabtoside	Anti-parasitic	[363]
Demospongiae/Heteroscleromorpha	*Mycale hentscheli*	Terpenoids	Mycalamide E	Enzyme inhibition	[364]
Demospongiae/Heteroscleromorpha	*Myrmekioderma sp.*	Terpenoids	Oxobolenes (2)	Cytotoxic against cancer cell lines	[365]
Demospongiae/Heteroscleromorpha	*Negombata cortica*	Terpenoids	Terpenoids (7)	Cytotoxic against cancer cell lines	[366]
Demospongiae/Heteroscleromorpha	*Neopetrosia proxima*	Terpenoids	Neoptrosiquinone A-B (2)	Cytotoxic against cancer cell lines	[367]
Demospongiae/Heteroscleromorpha	*Petrosia alfiani*	Terpenoids	Quinones (3)	Enzyme inhibition	[368]
Demospongiae/Heteroscleromorpha	*Petrosia nigricans*	Terpenoids	Petronigrione	Cytotoxic against cancer cell lines	[369]

Demospongiae/Heteroscleromorpha	*Phorbas gukhulensis*	Terpenoids	Gukulenin C-F (4)	Cytotoxic against cancer cell lines	[370]
Demospongiae/Heteroscleromorpha	*Phorbas sp.*	Terpenoids	Phorbasones A-B (2)	Neuronal signal transmission	[371]
Demospongiae/Heteroscleromorpha	*Phorbas sp.*	Terpenoids	Sesterterpenoids (2)	Cytotoxic against cancer cell lines	[372]
Demospongiae/Heteroscleromorpha	*Phorbas sp.*	Terpenoids	Sesterterpene (5)	Enzyme inhibition	[373]
Demospongiae/Heteroscleromorpha	*Pseudoaxinella flava*	Terpenoids	Isonitrile	Cytotoxic against cancer cell lines	[374]
Demospongiae/Heteroscleromorpha	*Rhabdastrella globostellata*	Terpenoids	Globostelletin A-I (9)	Cytotoxic against cancer cell lines	[375]
Demospongiae/Heteroscleromorpha	*Rhabdastrella globostellata*	Terpenoids	Rhabdastin A-G (7)	Cytotoxic against cancer cell lines	[376]
Demospongiae/Heteroscleromorpha	*Rhabdastrella globostellata*	Terpenoids	Stelliferins (2)	Antimicrobial	[377]
Demospongiae/Heteroscleromorpha	*Stylissa massa*	Terpenoids	Diterpenes (3)	Anti-malarial	[378]
Demospongiae/Heteroscleromorpha	*Svenzea flava*	Terpenoids	Diterpenoids (2)	Antimicrobial	[379]
Demospongiae/Heteroscleromorpha	*Tedania ignis*	Terpenoids	Tedarenes A-B (2)	Anti-inflammatory	[380]
Demospongiae/Heteroscleromorpha	*Theonella swinhoei*	Terpenoids	Swinholide J	Cytotoxic against cancer cell lines	[381]
Demospongiae/Heteroscleromorpha	*Theonella swinhoei*	Terpenoids	Terpenoids (2)	Cytotoxic against cancer cell lines	[382]

Demospongiae/Heteroscleromorpha	*Xestospongia sp.*	Terpenoids	Xestosaprols (2)	Enzyme inhibition	[383]
Demospongiae/Heteroscleromorpha	*Xestospongia sp.*	Terpenoids	Xestasporols (2)	Antimicrobial	[384]
Demospongiae/Heteroscleromorpha	*Xestospongia sp.*	Terpenoids	Xestosparols (8)	Enzyme inhibition	[385]
Demospongiae/Heteroscleromorpha	*Xestospongia sp.*	Terpenoids	S-oxide (1)	Cytotoxic against cancer cell lines	[386]
Demospongiae/Heteroscleromorpha	*Agleas nakamurai*	Terpenoids	Agelasines (3)	Antimicrobial	[387]
Demospongiae/Heteroscleromorpha	*Arenosclera sp.*	Terpenoids	Brominated ether	Antimicrobial	[388]
Demospongiae/Heteroscleromorpha	*Clathria gombawuiensis*	Terpenoids	Sesterterpene (3)	Cytotoxic against cancer cell lines	[389]
Demospongiae/Heteroscleromorpha	*Diacarnus megaspinorhabdosa*	Terpenoids	Farnesyl derivatives (2)	Cytotoxic against cancer cell lines	[390]
Demospongiae/Heteroscleromorpha	*Diacarnus megaspinorhabdosa*	Terpenoids	Dicarnuperoxides	Anti-malarial	[391]
Demospongiae/Heteroscleromorpha	*Myrmekioderma sp.*	Terpenoids	Bisabolane derivatives (4)	Lipids-reducing activity	[392]
Demospongiae/Heteroscleromorpha	*Petrosia corticata*	Terpenoids	Stronglyphorine (2)	Antimicrobial	[393]
Demospongiae/Heteroscleromorpha	*Phorbas areolatus*	Terpenoids	Suberitane derivatives (3)	Cytotoxic against cancer cell lines	[394]
Demospongiae/Heteroscleromorpha	*Phorbas sp.*	Terpenoids	Alotaketals and ansellones	Antiviral	[395]

Demospongiae/Heteroscleromorpha	*Raspailia bouryesnaultae*	Terpenoids	Clerodane diterpenes (5)	Cytotoxic against cancer cell lines	[396]
Demospongiae/Heteroscleromorpha	*Rhabdastrella providentiae*	Terpenoids	Rhabdaprovidines A-C	Anti-inflammatory	[397]
Demospongiae/Heteroscleromorpha	*Rhabdastrella providentiae*	Terpenoids	Isomalabaricane analogues (1–5)	Cytotoxic against cancer cell lines	[398]
Demospongiae/Heteroscleromorpha	*Siphonochalina siphonella*	Terpenoids	Sipholenone	Enzyme inhibitors	[399]
Demospongiae/Heteroscleromorpha	*Stelletta sp.*	Terpenoids	Cyclobutastelettolide A-B	Pro-inflammatory	[400]
Demospongiae/Heteroscleromorpha	*Stelletta tenius*	Terpenoids	Stelletins (3)	Cytotoxic against cancer cell lines	[401]
Demospongiae/Heteroscleromorpha	*Strongylophora strongylata*	Terpenoids	Stronglyphorine	Enzyme inhibition	[402]
Demospongiae/Heteroscleromorpha	*Svenzea flava*	Terpenoids	Monamphilectines B-C	Anti-malarial	[403]
Demospongiae/Heteroscleromorpha	*Theonella swinhoei*	Terpenoids	P10 (a new blasticidin S analog)	Antibacterial	[404]
Demospongiae/Heteroscleromorpha	*Xestospongia sp.*	Terpenoids	Xestoadociaminals A–D (7)	Enzyme inhibition	[405]
Demospongiae/Heteroscleromorpha	*Xestospongia sp.*	Terpenoids	Fennebrecin C-D	Cytotoxic against cancer cell lines	[406]
Demospongiae/Keratosa	*Dysidea sp.*	Alkaloids	Dysideanins A-B (2)	Antimicrobial	[407]
Demospongiae/Keratosa	*Fascaplysimopsis sp.*	Alkaloids	Salarins D-J (7)	Cytotoxic against cancer cell lines	[408]
Demospongiae/Keratosa	*Hyattella sp.*	Alkaloids	Psammaplysin G	Anti-malarial	[409]

Demospongiae/Keratosa	*Hyrtios reticulatus*	Alkaloids	Hytriocarboline	Cytotoxic against cancer cell lines	[410]
Demospongiae/Keratosa	*Hyrtios reticulatus*	Alkaloids	Hyrtiorectulins A-E	Enzyme inhibition	[411]
Demospongiae/Keratosa	*Hyrtios sp.*	Alkaloids	Brominated indole	Antioxidant	[412]
Demospongiae/Keratosa	*Hyrtios sp.*	Alkaloids	Hyrtioerectines D-F (3)	Antimicrobial	[413]
Demospongiae/Keratosa	*Hyrtios sp.*	Alkaloids	Hyrtimomines A-C (3)	Cytotoxic against cancer cell lines	[414]
Demospongiae/Keratosa	*Hyrtios sp.*	Alkaloids	Hyrtimomines D-F (3)	Antifungal	[415]
Demospongiae/Keratosa	*Cacospongia mycofijiensis*	Alkaloids	Zampanolides B–E	Cytotoxic against cancer cell lines	[416]
Demospongiae/Keratosa	*Fascaplysinopsis reticulata*	Alkaloids	Spiroreticulatine (2)	Anti-inflammatory	[417]
Demospongiae/Keratosa	*Fascaplysinopsis reticulata*	Alkaloids	Subereamolline D	Cytotoxic against cancer cell lines	[418]
Demospongiae/Keratosa	*Fascaplysinopsis reticulata*	Alkaloids	Tryptophan alaloids (3)	Anti-malarial	[419]
Demospongiae/Keratosa	*Hexadella sp.*	Alkaloids	Anomians	Cytotoxic against cancer cell lines	[420]
Demospongiae/Keratosa	*Hyrtios sp.*	Alkaloids	Hyrtinadines C and D	Antimicrobial	[421]
Demospongiae/Keratosa	*Hyrtios sp.*	Alkaloids	Dihydrohyrtiosulawesine	Enzyme inhibitors	[422]
Demospongiae/Keratosa	*Hyrtios sp.*	Alkaloids	Ishigadine A	Cytotoxic against cancer cell lines	[423]
Demospongiae/Keratosa	*Hyrtios erecta*	Alkaloids	Hainanerectamines A-C	Enzyme inhibition	[424]
Demospongiae/Keratosa	*Hyrtios sp.*	Alkaloids	Hyrtimomines (6)	Antimicrobial	[425]
Demospongiae/Keratosa	*Hyrtios sp.*	Alkaloids	Tryptophan alkaloids	Cytotoxic against cancer cell lines	[426]
Demospongiae/Keratosa	*Luffariella variabilis*	Alkaloids	Variabines A-B	Proteasome inhibition	[427]

Demospongiae/Keratosa	*Dysidea sp.*	Lipids	Halogenated lipids (2)	Cytotoxic against cancer cell lines	[428]
Demospongiae/Keratosa	*Carteriospongia sp.*	Lipids	Scalaranes (5)	Cytotoxic against cancer cell lines	[429]
Demospongiae/Keratosa	*Hippospongia sp.*	Lipids	Hippospongide C	Cytotoxic against cancer cell lines	[430]
Demospongiae/Keratosa	*Coscinoderma sp.*	Lipids	Suavine analogs (5)	Cytotoxic against cancer cell lines	[431]
Demospongiae/Keratosa	*Carteriospongia sp.*	Lipids	Scalaraenes	Cytotoxic against cancer cell lines	[432]
Demospongiae/Keratosa	*Dysidea sp.*	Lipids	Dysiroid A and B	Antibacterial	[433]
Demospongiae/Keratosa	*Hyrtios erectus*	Lipids	Hyrtiolides	Cytotoxic against cancer cell lines	[434]
Demospongiae/Keratosa	*Hyrtios erectus*	Lipids	Petrosaspongia C	Cytotoxic against cancer cell lines	[435]
Demospongiae/Keratosa	*Hyrtios sp.*	Peptides	Hyrtiosergamines A-B	Antimicrobial	[436]
Demospongiae/Keratosa	*Dysidea herbacea*	Peptides	Dysiherbaine	Neurotoxicity	[437]
Demospongiae/Keratosa	*Hippospongia lachne*	Polyketides	Hippolachnin	Antifungal	[438]
Demospongiae/Keratosa	*Hyrtios erectus*	Polyketides	Erectuseneols A−F	Cytotoxic against cancer cell lines	[439]
Demospongiae/Keratosa	*Hyrtios erectus*	Polyketides	Alkyl benzoat	Cytotoxic against cancer cell lines	[440]
Demospongiae/Keratosa	*Petrosaspongia sp.*	Polyketides	Biakamides A-D	Antiproliferative against human cancer cells	[441]
Demospongiae/Keratosa	*Dysidea sp.*	Steroids	Dysideasterols F-H (3)	Cytotoxic against cancer cell lines	[442]

Demospongiae/Keratosa	*Ircinia sp.*	Steroids	Steroid	Antimicrobial	[443]
Demospongiae/Keratosa	*Dactylospongia elegans*	Steroids	Steroids (2)	Cytotoxic against cancer cell lines	[444]
Demospongiae/Keratosa	*Ircinia echinata*	Steroids	Epoxysterols (6)	Cytotoxic against cancer cell lines	[445]
Demospongiae/Keratosa	*Ircinia sp.*	Steroids	Sterol	Antimicrobial	[446]
Demospongiae/Keratosa	*Dendrilla nigra*	Terpenoids	Denigrins A-C	Antimicrobial	[447]
Demospongiae/Keratosa	*Dysidea fragilis*	Terpenoids	Dysidaminones A-M (13)	Anti-inflammatory	[448]
Demospongiae/Keratosa	*Dysidea avara*	Terpenoids	Dysideanones A-C	Cytotoxic against cancer cell lines	[449]
Demospongiae/Keratosa	*Dysidea sp.*	Terpenoids	Dysidinoid A	Antimicrobial	[450]
Demospongiae/Keratosa	*Hippospongia lachne*	Terpenoids	Hippolide derivatives (5)	Enzyme inhibition	[451]
Demospongiae/Keratosa	*Dactylospongia metachromia*	Terpenoids	Quinones (7)	Cytotoxic against cancer cell lines	[452]
Demospongiae/Keratosa	*Hyrtios sp.*	Terpenoids	Scalarene derivatives (5)	Enzyme inhibition	[453]
Demospongiae/Keratosa	*Cacospongia mycofijiensis*	Terpenoids	Cyclic terpenoids (2)	Anti-parasitic	[454]
Demospongiae/Keratosa	*Candidaspongia sp.*	Terpenoids	Precandidaspongolides A-B (2)	Cytotoxic against cancer cell lines	[455]
Demospongiae/Keratosa	*Carteriospongia sp.*	Terpenoids	Flabelliferins A-B (2)	Cytotoxic against cancer cell lines	[456]
Demospongiae/Keratosa	*Coscinoderma mathewsi*	Terpenoids	Terpenoids (3)	Antimicrobial	[457]
Demospongiae/Keratosa	*Coscinoderma sp.*	Terpenoids	Merosesterterpenoids (2)	Cytotoxic against cancer cell lines	[458]
Demospongiae/Keratosa	*Coscinoderma sp.*	Terpenoids	Sesterterpene (5)	Antimicrobial	[459]

Demospongiae/Keratosa	*Dactylospongia elegans*	Terpenoids	Nakijinol B and spongines (3)	Cytotoxic against cancer cell lines	[460]
Demospongiae/Keratosa	*Dactylospongia elegans*	Terpenoids	Quinones (5)	Cytotoxic against cancer cell lines	[461]
Demospongiae/Keratosa	*Dysidea avara*	Terpenoids	Dysideavarones A-D	Cytotoxic against cancer cell lines	[462]
Demospongiae/Keratosa	*Dysidea avara*	Terpenoids	Sesquiterpenoid	Cytotoxic against cancer cell lines	[463]
Demospongiae/Keratosa	*Dysidea sp.*	Terpenoids	Meroterpenoids (5)	Antioxidant	[464]
Demospongiae/Keratosa	*Dysidea sp.*	Terpenoids	Diplopuupehenone	Antioxidant	[465]
Demospongiae/Keratosa	*Fasciospongia sp.*	Terpenoids	Merodíterpenoids (6)	Antimicrobial	[466]
Demospongiae/Keratosa	*Hippospongia lachne*	Terpenoids	Hippolides A-H (8)	Enzyme inhibition	[467]
Demospongiae/Keratosa	*Hippospongia sp.*	Terpenoids	Hippospongides A-B	Cytotoxic against cancer cell lines	[468]
Demospongiae/Keratosa	*Hyrtios communis*	Terpenoids	Daeolides (6)	Enzyme inhibition	[469]
Demospongiae/Keratosa	*Ircinia sp.*	Terpenoids	Terpenes (3)	Cytotoxic against cancer cell lines	[470]
Demospongiae/Keratosa	*Cacospongia sp.*	Terpenoids	Trunculin X-Y	Cytotoxic against cancer cell lines	[471]
Demospongiae/Keratosa	*Carteriospongia foliascens*	Terpenoids	Sesterterpene (11)	Cytotoxic against cancer cell lines	[472]
Demospongiae/Keratosa	*Coscinoderma sp.*	Terpenoids	Suavine analogs (2)	Cytotoxic against cancer cell lines	[473]
Demospongiae/Keratosa	*Dactylospongia elegans*	Terpenoids	Dolabellane diterpene	Antibacterial	[474]
Demospongiae/Keratosa	*Dactylospongia elegans*	Terpenoids	Popolohuanones G - I	Anti-inflammatory	[475]

Demospongiae/Keratosa	*Dactylospongia elegans*	Terpenoids	Polyketides (1)	Cytotoxic against cancer cell lines	[476]
Demospongiae/Keratosa	*Dactylospongia elegans*	Terpenoids	Terpenoids (4)	Antimicrobial	[477]
Demospongiae/Keratosa	*Dactylospongia metachromia*	Terpenoids	Dactylocyanines A–H	Blue pigments	[478]
Demospongiae/Keratosa	*Dactylospongia sp.*	Terpenoids	Puupehenol	Antimicrobial	[479]
Demospongiae/Keratosa	*Dactylospongia sp.*	Terpenoids	Dactylospongins A–D	Anti-inflammatory	[480]
Demospongiae/Keratosa	*Dendrilla membranosa*	Terpenoids	Darwinolide	Inhibits Methicillin-Resistant Staphylococcus aureus Biofilm	[481]
Demospongiae/Keratosa	*Dendrilla rosea*	Terpenoids	Aplyresols (3)	Antimicrobial	[482]
Demospongiae/Keratosa	*Dictyoceratid sp.*	Terpenoids	Luakuliides (3)	Cytotoxic against cancer cell lines	[483]
Demospongiae/Keratosa	*Dysidea arenaria*	Terpenoids	Dysarenone	Anti-inflammatory	[484]
Demospongiae/Keratosa	*Dysidea avara*	Terpenoids	Quinones (8)	Cytotoxic against cancer cell lines	[485]
Demospongiae/Keratosa	*Dysidea cinerea*	Terpenoids	Cinerols (11)	Enzyme inhibition	[486]
Demospongiae/Keratosa	*Dysidea fragilis*	Terpenoids	Dysifragilones A-C	Anti-inflammatory	[487]
Demospongiae/Keratosa	*Dysidea frondosa*	Terpenoids	Frondoplysins A-B (2)	Enzyme inhibition	[488]
Demospongiae/Keratosa	*Dysidea sp.*	Terpenoids	Meroterpenoidss (6)	Antimicrobial	[489]
Demospongiae/Keratosa	*Dysidea sp.*	Terpenoids	Avapyran	Inhibits PTP-1B	[490]
Demospongiae/Keratosa	*Dysidea sp.*	Terpenoids	Dysiherbols A–C	NF-κB inhibitory and cytotoxic activity	[491]
Demospongiae/Keratosa	*Dysidea sp.*	Terpenoids	Dysiherbol A-C	Anti-inflammatory	[492]

Demospongiae/Keratosa	*Dysidea villosa*	Terpenoids	Dysivillosins A–D	Reduce release of pro-inflammatory cytokines	[493]
Demospongiae/Keratosa	*Euryspongia sp.*	Terpenoids	Euryspongins and eurydiene (3)	Enzyme inhibition	[494]
Demospongiae/Keratosa	*Hippospongia lachne*	Terpenoids	Hippolide JJ	Antifungal	[495]
Demospongiae/Keratosa	*Hippospongia sp.*	Terpenoids	Rhopaloic acid H	Cytotoxic against cancer cell lines	[496]
Demospongiae/Keratosa	*Hyattella sp.*	Terpenoids	Sesterterpene (2)	Enzyme inhibition	[497]
Demospongiae/Keratosa	*Hyrtios digitatus*	Terpenoids	19-methoxy-9,15-ene-puupehenol	Active against scavenger Receptor-Class B Type 1 HepG2 (SR-B1 HepG2) stable cell lines	[498]
Demospongiae/Keratosa	*Hyrtios erectus*	Terpenoids	Erectusolides B, C, D	Cytotoxic against cancer cell lines	[499]
Demospongiae/Keratosa	*Hyrtios erectus*	Terpenoids	Scalarane derivative	Cytotoxic against cancer cell lines	[500]
Demospongiae/Keratosa	*Hyrtios sp.*	Terpenoids	Nakijinol G	Inhibits PTP-1B	[501]
Demospongiae/Keratosa	*Ircinia felix*	Terpenoids	Sesterterpene (5)	Cytotoxic against cancer cell lines	[502]
Demospongiae/Keratosa	*Ircinia felix*	Terpenoids	Sesterterpene (2)	Cytotoxic against cancer cell lines	[503]
Demospongiae/Keratosa	*Lamellodysidea herbacea*	Terpenoids	Sesquiterpenes (2)	Enzyme inhibitors	[504]
Demospongiae/Keratosa	*Luffariella sp.*	Terpenoids	Sesterterpene (6)	Cytotoxic against cancer cell lines	[505]
Demospongiae/Keratosa	*Luffariella sp.*	Terpenoids	Terpenes	Cytotoxic against cancer cell lines	[79]
Demospongiae/Keratosa	*Ircinia sp.*	Terpenoids (5)	Terpenoids	Receptor activator	[506]

Demospongiae/Verongimorpha	*Aplysinella strongylata*	Alkaloids	Psammaplysin	Antimicrobial	[507]
Demospongiae/Verongimorpha	*Ianthella flabelliformis*	Alkaloids	Bastadins (2)	Receptor activator	[508]
Demospongiae/Verongimorpha	*Ianthella quadrangulata*	Alkaloids	Ianthellamide A	Enzyme inhibition	[509]
Demospongiae/Verongimorpha	*Ianthella reticulata*	Alkaloids	Bastadins (2)	Anti-parasitic	[510]
Demospongiae/Verongimorpha	*Ianthella sp.*	Alkaloids	Lamellarine O	Antimicrobial	[511]
Demospongiae/Verongimorpha	*Ianthella sp.*	Alkaloids	Dictyodendrins F-J (5)	Enzyme inhibition	[512]
Demospongiae/Verongimorpha	*Pseudoceratina arabica*	Alkaloids	Ceratinines A-E (5)	Inhibit cancer cell migration	[513]
Demospongiae/Verongimorpha	*Pseudoceratina sp.*	Alkaloids	Ceratinadins A-C (3)	Antifungal	[514]
Demospongiae/Verongimorpha	*Pseudoceratina sp.*	Alkaloids	Pseudoceramines A-D	Antimicrobial	[515]
Demospongiae/Verongimorpha	*Pseudoceratina verrucosa*	Alkaloids	Aplysamine 7	Cytotoxic against cancer cell lines	[516]
Demospongiae/Verongimorpha	*Aplysina lacunosa*	Alkaloids	Bromotyrosines (3)	Cytotoxic against cancer cell lines	[517]
Demospongiae/Verongimorpha	*Aplysinella sp.*	Alkaloids	Pupuramin M	Cytotoxic against cancer cell lines	[518]
Demospongiae/Verongimorpha	*Aplysinella sp.*	Alkaloids	Arapyllisins	Antibacterial	[518]

Demospongiae/Verongimorpha	*Aplysinella sp.*	Alkaloids	Psammaplysin analogues (2)	Cytotoxic against cancer cell lines	[519]
Demospongiae/Verongimorpha	*Narrabeena nigra*	Alkaloids	Brominated tryptamines (8)	Antioxidant	[200]
Demospongiae/Verongimorpha	*Pseudoceratina arabica*	Alkaloids	Ceratinines F-H	Cytotoxic against cancer cell lines	[520]
Demospongiae/Verongimorpha	*Pseudoceratina purpurea*	Alkaloids	Bromotyrosines (2)	Antimicrobial	[521]
Demospongiae/Verongimorpha	*Pseudoceratina sp.*	Alkaloids	Pseudoceratinamides	Antimicrobial	[522]
Demospongiae/Verongimorpha	*Pseudoceratina sp.*	Alkaloids	Ceratinadins E-F	Antimalarial	[523]
Demospongiae/Verongimorpha	*Pseudoceratina sp.*	Peptides	Pseudoceramines A-D	Antimicrobial	[524]
Demospongiae/Verongimorpha	*Aplysinella sp.*	Terpenoids	Bromotyrosine drivatives (4)	Cardiovascular disease	[525]
Demospongiae/Verongimorpha	*Ianthella basta*	Terpenoids	Sesquibastadin	Enzyme inhibition	[526]
Homoscleromorpha	*Plakortis lita*	Alkaloids	Thiaplakortones A-D (4)	Anti-malarial	[527]
Homoscleromorpha	*Plakortis sp.*	Alkaloids	Alkaloids (3)	Enzyme inhibition	[528]
Homoscleromorpha	*Plakortis sp.*	Alkaloids	Indoles (2)	Cytotoxic against cancer cell lines	[529]
Homoscleromorpha	*Corticium sp.*	Lipids	Plakinamine N	Antitubercular	[530]
Homoscleromorpha	*Plakinastrella clathrata*	Lipids	Fatty acid	Anti-inflammatory	[531]
Homoscleromorpha	*Plakortis angulospiculatus*	Lipids	Plakortisnic acid (2)	Antifungal	[532]
Homoscleromorpha	*Plakortis halichondrioides*	Lipids	Lipids (2)	Anti-inflammatory	[533]

Homoscleromorpha	*Plakortis lita*	Lipids	Lactones (3)	Anti-malarial	[534]
Homoscleromorpha	*Plakortis simplex*	Lipids	Manadoperoxides A-D (4)	Anti-malarial	[535]
Homoscleromorpha	*Plakortis simplex*	Lipids	Methylated peroxidic acids (6)	Cytotoxic against cancer cell lines	[536]
Homoscleromorpha	*Plakortis sp.*	Lipids	Manzamenone O	Antimicrobial	[537]
Homoscleromorpha	*Corticium niger*	Lipids	Plakinamines N and O	Cytotoxic against cancer cell lines	[538]
Homoscleromorpha	*Plakina sp.*	Lipids	Plakinamine P	Antimicrobial	[539]
Homoscleromorpha	*Oscarella stillans*	Peptides	Oscarellin	Inhibitor of Inflammatory Cytokines in Macrophages	[540]
Homoscleromorpha	*Plakinastrella mamillaris*	Polyketides	Plakortides R-U (4)	Anti-malarial	[541]
Homoscleromorpha	*Plakortis simplex*	Polyketides	Simplextones A-B (2)	Cytotoxic against cancer cell lines	[542]
Homoscleromorpha	*Plakortis simplex*	Polyketides	Simplextone C	Enzyme inhibition	[543]
Homoscleromorpha	*Plakortis sp.*	Polyketides	Manzamenones L-N (3)	Antimicrobial	[544]
Homoscleromorpha	*Plakortis simplex*	Polyketides	Polyketides endoperoxides (5)	Anti-malarial	[545]
Homoscleromorpha	*Plakortis bergquistae*	Polyketides	Manadidioxans A-E	Antimicrobial	[546]
Homoscleromorpha	*Plakortis halichondrioides*	Polyketides	Plakortinic acid A and B	Cytotoxic against cancer cell lines	[547]
Homoscleromorpha	*Plakortis halichondrioides*	Polyketides	Plakinidone B-C	Antimicrobial	[548]
Homoscleromorpha	*Plakortis simplex*	Polyketides	Plakdiepoxide	PPAR modulation	[549]
Homoscleromorpha	*Plakortis sp.*	Steroids	Incisterol A5 and A6	Receptor activator	[550]
Homoscleromorpha	*Plakinastrella mamillaris*	Terpenoids	Placktilactones A-F	Receptor activator	[551]
Homoscleromorpha	*Plakinastrella mamillaris*	Terpenoids	Gracilioethers E-J (6)	Anti-malarial	[552]

Homoscleromorpha	*Plakortis lita*	Terpenoids	Several compounds (9)	Anti-parasitic	[553]
Homoscleromorpha	*Plakortis simplex*	Terpenoids	Plakortide derivatives (2)	Anti-parasitic	[554]
Homoscleromorpha	*Plakortis simplex*	Terpenoids	Woodylides A-C	Antifungal	[555]
Homoscleromorpha	*Plakortis simplex*	Terpenoids	Simplexolides A-E (6)	Antifungal	[556]
Homoscleromorpha	*Plakortis sp.*	Terpenoids	Lehualide E-K	Antifungal	[557]
Homoscleromorpha	*Plakortis angulospiculatus*	Terpenoids	Plakortides (3)	Cytotoxic against cancer cell lines	[558]
Homoscleromorpha	*Plakortis sp.*	Terpenoids	Cyclic peroxide acids (3)	Cytotoxic against cancer cell lines	[559]
n.d.	*n.d..*	Lipids	Phenyl alkene	Cytotoxic against cancer cell lines	[560]
n.d.	*n.d.*	Terpenoids	Diterpene (2)	Cytotoxic against cancer cell lines	[561]

Supplementary Table S2. Cnidarian bioactive natural products isolated from 2010 to 2019. (Sorted alphabetically according to Class/Subclass and then to Chemical class of compounds)

**Class/Subclass**	**Producer species**	**Chemical Class of compounds**	**Compound(s)**	**Activity/toxicity**	**References**
Anthozoa/Hexacorallia	*Zoanthus sp.*	Alkaloids	Zoaramine and zoarenone	Anti-osteoporotic	[562]

Anthozoa/Hexacorallia	*Zoanthus kuroshio*	Alkaloids	Kuroshines A, C–G	Cytotoxic towards cancer cells	[563]
Anthozoa/Hexacorallia	*Zoanthus kuroshio*	Alkaloids	5α-iodozoanthenamine (1) and 11β-chloro-11-deoxykuroshine A (2)	Anti-inflammatory	[564]
Anthozoa/Hexacorallia	*Palythoa tuberculosa*	Alkaloids	Tuberazine A	anti-lymphangiogenesis activity	[565]
Anthozoa/Hexacorallia	*Zoanthus* cf. *pulchellus*	Alkaloids	Zoanthamine: 3-acetoxynorzoanthamine	neuroinflammatory activity	[566]
Anthozoa/Hexacorallia	*Zoanthus vietnamensis*	Alkaloids	Kuroshines H−J	Anti-lymphangiogenic	[567]
Anthozoa/Hexacorallia	*Zoanthus vietnamensis*	Alkaloids	Kuroshine K	Anti-lymphangiogenic	[567]
Anthozoa/Hexacorallia	*Zoanthus vietnamensis*	Alkaloids	27-hydroxykuroshine A	Anti-lymphangiogenic	[567]
Anthozoa/Hexacorallia	*Zoanthus vietnamensis*	Alkaloids	27-methyl glycinate zoanthenamine	Anti-lymphangiogenic	[567]
Anthozoa/Hexacorallia	*Zoanthus vietnamensis*	Alkaloids	27-hydroxyzoanthenamine	Anti-lymphangiogenic	[567]
Anthozoa/Hexacorallia	*Zoanthus vietnamensis*	Alkaloids	27-methyl glycinate kuroshine A	Anti-lymphangiogenic	[567]
Anthozoa/Hexacorallia	*Zoanthus vietnamensis*	Alkaloids	3β-hydroxy-28-deoxyzoanthenamine	Anti-lymphangiogenic	[567]
Anthozoa/Hexacorallia	*Zoanthus vietnamensis*	Alkaloids	14α-hydroxy-28-deoxyzoanthenamine	Anti-lymphangiogenic	[567]
Anthozoa/Hexacorallia	*Zoanthus vietnamensis*	Alkaloids	27-hydroxy-28-deoxyzoanthenamine	Anti-lymphangiogenic	[567]
Anthozoa/Hexacorallia	*Antipathozoanthus hickmani*	Dipeptides	Valdiviamides B	Cytotoxic against human cancer cells	[568]
Anthozoa/Hexacorallia	*Antipathes dichotoma*	Lipids/Sphingolipids	Sphingolipid (1)	Antibacterial	[569]

Anthozoa/Hexacorallia	*Urticina crassicornis*	Peptides	Crassicorin-iand ii	Antimicrobial	[570]
Anthozoa/Hexacorallia	*Anthopleura elegantissima*	Peptides	Apetx4.	KV10.1 inhibitor	[571]
Anthozoa/Hexacorallia	*Heteractis crispa*	Peptides	Rhcgs1.19, rhcgs1.36	Anti-inflammatory	[572]
Anthozoa/Hexacorallia	*Metridium senile*	Peptides	Anmtx Ms 9a-1	Analgesic	[573]
Anthozoa/Hexacorallia	*Heteractis crispa*	Peptides	Π-anmtx Hcr 1b-2, -3, and -4	neurotoxin, antihyperalgesic activity	[574]
Anthozoa/Hexacorallia	*Anthopleura anjunae*	Peptides	Aap-h	cytotoxicity to cancer cell lines	[575]
Anthozoa/Hexacorallia	*Actinia tenebrosa*	Peptides	K-actitoxin-Ate1a	neurotoxin,	[576]
Anthozoa/Hexacorallia	*Heteractis magnifica*	Peptides	Magnificamide	alfa-amylase inhibitor	[577]
Anthozoa/Hexacorallia	*Montipora digitata*	Polyacetylenes	Montiporic acid D (**1**)	Antibacterial	[578]
Anthozoa/Hexacorallia	*Stichodactyla helianthus*	Proteins	Sticholysin II	Pore forming toxin	[579]
Anthozoa/Hexacorallia	*Stichodactyla helianthus*	Proteins	Sticholysins I and II	actinoporin	[580]
Anthozoa/Hexacorallia	*Stylophora pistillata*	Proteins	Δ-pocilopotoxin-spi1	actinoporin	[581]
Anthozoa/Hexacorallia	*Anthopleura midori*	Steroids	Epoxyergosterols **1** and **2**	Cytotoxic towards cancer cells	[582]
Anthozoa/Hexacorallia	*Palythoa tuberculosa*	Steroids	Palysterols F	Apoptosis induction	[583]
Anthozoa/Hexacorallia	*Zoanthus spp.*	Steroids	Zoanthone A	Antiviral	[584]

Anthozoa/Hexacorallia	*Palythoa mutuki*	Steroids	Palythone A	Antiviral	[585]
Anthozoa/Hexacorallia	*Palythoa caribaeorum, Palythoa variabilis*	Steroids	6β-carboxyl-24(R)-(8→6)-abeo-ergostan-3β,5β-diol	Cytotoxic towards cancer cells	[586]
Anthozoa/Octocorallia	*Cespitularia sp.*		Alcyonolide congeners (1-4)	Cytotoxic towards cancer cells	[587]
Anthozoa/Octocorallia	*Sarcophyton ehrenbergi*		(S,E)-2-methyloctyl 4-(3-methoxyphenyl)propenoate	Cytotoxic towards cancer cells	[588]
Anthozoa/Octocorallia	*Echinogorgia pseudossapo*	Alkaloids	Malonganenones L (1) and Q (6)	PDE4D* inhibitor	[589]
Anthozoa/Octocorallia	*Abietinaria abietina*	Alkaloids	6-bromogramine (1) and bis-6-bromogramine (2)	Activate NF-kb-dependent transcriptional activity in JB6 Cl 41 NF-κb cells	[590]
Anthozoa/Octocorallia	*Muriceides collaris*	Alkaloids	Guaiazulene alkaloids muriceidines A–C (1–3)	Cytotoxic towards cancer cells, antifouling	[591]
Anthozoa/Octocorallia	*Echinogorgia pseudossapo*	Alkaloids	Pseudozoanthoxanthins III (1)	Antiviral activity	[592]
Anthozoa/Octocorallia	*Paramuricea clavata*	Alkaloids	2-bromo-N-methyltryptamine (1)	Antifouling activity, Antibacterial	[593]
Anthozoa/Octocorallia	*Paramuricea clavata*	Alkaloids	3-bromo-N-methyltyramine (2)	Antifouling activity, antibacterial	[593]
Anthozoa/Octocorallia	*Euplexaura robusta*	Alkaloids	Malonganenones I–K (**1–3)**	Cytotoxic towards cancer cells	[594]
Anthozoa/Octocorallia	*Leptogorgia gilchristi*	Alkaloids	Malonganenones A–C (**1**–**3**)	Cytotoxic towards cancer cells	[595]
Anthozoa/Octocorallia	*Sinularia sp.*	Alkaloids	Sinulasulfoxide (**1**) and sinulasulfone (**2**)	Anti-inflammatory activity	[596]

Anthozoa/Octocorallia	*Subergorgia suberosa*	butenolide	Butenolide (5R)-5-(1-ethoxypropyl)-5-hydroxy-3, 4-dimethylfuran-2(5H)-one (1)	Antifouling	[597]
Anthozoa/Octocorallia	*Menella kanisa*	Cyclic dipeptides	Menazepine A (1)	Antifouling	[598]
Anthozoa/Octocorallia	*Sinularia sp*	Cyclopentenone, butenolide-type analogues	Sinularones A–I (**1**–**9**)	Antifouling	[599]
Anthozoa/Octocorallia	*Cladiella hirsuta*	Glycosides	Cladophenol glycosides A (3) and B (4)	Cytotoxic activities	[600]
Anthozoa/Octocorallia	*Dichotella fragilis*	Glycosides	Fragilioside A (1),fragilioside B (2)	Antifouling activity	[601]
Anthozoa/Octocorallia	*Sarcophyton ehrenbergi.*	Lipids/Fatty acids and derivatives	Sarcoehrendins A–J (**1**–**10**)	PDE4D* inhibitor	[602]
Anthozoa/Octocorallia	*Clavularia viridis*	Lipids/Fatty acids and derivatives	Claviridins A–D (1–4)	Cytotoxic towards cancer cells	[603]
Anthozoa/Octocorallia	*Sinularia sp*	Lipids/Glicerolipids	Sinularioside (2)	Anti-inflammatory	[604]
Anthozoa/Octocorallia	*Anthogorgia caerulea*	Macrolides	Avermectins B1c and B1e (1 and 2)	Antifouling	[605]
Anthozoa/Octocorallia	*Stragulum bicolor*	Macrolides	Amphidinolide P, T1, C4, B8, and B9	Cytotoxic towards cancer cells	[606]
Anthozoa/Octocorallia	*Stragulum bicolor*	Macrolides	Stragulin	Cytotoxic against human cancer cells	[607]
Anthozoa/Octocorallia	*Clavularia viridis*	Polyacetylenes	Polyacetylene	cytotoxicity to cancer cell lines	[608]
Anthozoa/Octocorallia	*Sinularia flexibilis*	Quinones	Flexibilisquinone (1)	Anti-inflammatory	[609]
Anthozoa/Octocorallia	*Sinularia capillosa*	Quinones	Capilloquinol (1)	Cytotoxic towards cancer cells,	[610]

Anthozoa/Octocorallia	*Astrogorgia dumbea*	Steroidal saponins	Astrogorgiosides A (1) and B (2) bearing acetamido-glucose moieties, and astrogorgioside C (3)	Cytotoxic towards cancer cells	[611]
Anthozoa/Octocorallia	*Carijoa sp*	Steroids	Pregnane steroid (1)	Cytotoxic towards cancer cells, antibacterial	[612]
Anthozoa/Octocorallia	*Menella kanisa.*	Steroids	Polyoxygenated steroids (**6, 9, 14–18, 20–23, 25–27**)	Cytotoxic towards cancer cells	[613]
Anthozoa/Octocorallia	*Muriceopsis flavida*	Steroids	Muriflasteroids A–C (**1–3**)	Cytotoxic towards cancer cells	[614]
Anthozoa/Octocorallia	*Nephthea chabrolii*	Steroids	Nebrosteroid Q (**1**) and two new cytotoxic 19-norergosterols, nebrosteroids R and S (**2** and **3**)	Cytotoxic towards cancer cells	[615]
Anthozoa/Octocorallia	*Nephthea sp.*	Steroids	Steroids (1-5)	Cytotoxic towards cancer cells	[616]
Anthozoa/Octocorallia	*Nephthea sp.*	Steroids	Nephthoacetal (1)	Cytotoxic towards cancer cells, anti-fouling	[617]
Anthozoa/Octocorallia	*Paraminabea acronocephala*	Steroids	Paraminabic acids A-C (1-3)	Cytotoxic towards cancer cells, anti-inflammatory	[618]
Anthozoa/Octocorallia	*Sarcophyton sp*	Steroids	Compounds (**1–3**)	Cytotoxic towards cancer cells	[619]
Anthozoa/Octocorallia	*Sarcophyton sp.*	Steroids	Polyoxygenated steroids (**1–7**)	Antibacterial, antifungal	[620]
Anthozoa/Octocorallia	*Sarcophyton sp.*	Steroids	18-oxygenated polyhydroxy steroid, (24*S*)-ergostan-3β,5α,6β,18,25-pentaol 18,25-diacetate (**1**),	Cytotoxic towards cancer cells	[621]
Anthozoa/Octocorallia	*Sarcophyton trocheliophorum.*	Steroids	Zahramycins A (1) and B (2),	Antibacterial, antifungal	[622]
Anthozoa/Octocorallia	*Scleronephthya gracillimum*	Steroids	Sclerosteroids J–N (**1–5**)	Cytotoxic towards cancer cells, anti-inflammatory	[623]
Anthozoa/Octocorallia	*Sinularia brassica*	Steroids	Sinubrasolides A–G (1–7),	Cytotoxic towards cancer cells	[624]

Anthozoa/Octocorallia	*Sinularia candidula*	Steroids	3β-25-dihydroxy-4-methyl-5α,8α-epidioxy-2-ketoergost-9-ene (1) along with three new ceramides, N-[(2S,3R,E)-1,3-dihydroxyhexacos-4-en-2-yl]icosanamide (2), N-[(2S,3S,4R)-1,3,4-trihydroxyhexacosan-2-yl]icosanamide (3), and (R)-2′-hydroxy-N-[(2S,3S,4R)-1,3,4-trihydroxypentacosan-2-yl] nonadecanamide (4)	Antiviral	[625]
Anthozoa/Octocorallia	*Sinularia nanolobata.*	Steroids	9,11-secosteroids, 22α-acetoxy-24-methylene-3β,6α,11-trihydroxy-9, 11-seco-cholest-7-en-9-one (1) and 11-acetoxy-24-methylene-1β,3β,6α-trihydroxy-9, 11-seco-cholest-7-en-9-one (2)	Antiviral	[626]
Anthozoa/Octocorallia	*Subergorgia suberosa.*	Steroids	Subergorgols A–J (**1**–**10**)	Cytotoxic towards cancer cells	[627]
Anthozoa/Octocorallia	*Subergorgia suberosa*	Steroids	Pentacyclic steroid 16,22-epoxy-20β,23S-dihydroxycholest-1-ene-3-one (1) and 20β,23S-dihydroxycholest-1-ene-3,22-dione (2)	Antifouling	[628]
Anthozoa/Octocorallia	*Echinogorgia rebekka*	Steroids	Echrebsteroids A–D (1–4)	Antiviral	[629]
Anthozoa/Octocorallia	*Echinogorgia sassapo*	Steroids	Sassapols A (**1**)	Anti-inflammatory	[630]
Anthozoa/Octocorallia	*Leptogorgia punicea*	Steroids	Punicinols A–E (**1**–**5**)	Cytotoxic towards cancer cells	[631]
Anthozoa/Octocorallia	*Sarcophyton pauciplicatum*	Steroids	Sarcopanol A (**1**)	Anti-inflammatory	[632]
Anthozoa/Octocorallia	*Sarcophyton trocheliophorum, Sinularia flexibilis*	Steroids	Polyhydroxylated steroids, 7α-hydroxy-crassarosterol A (2)	Cytotoxic towards cancer cells	[633]

Anthozoa/Octocorallia	*Subergorgia suberosa*	Steroids	9,11-secosteroids 4	Cytotoxic towards cancer cells	[634]
Anthozoa/Octocorallia	*Verrucella umbraculum*	Steroids	Verumbsteroids A and B (**1** and **2)**	Cytotoxic towards cancer cells	[635]
Anthozoa/Octocorallia	*Cladiella hirsuta*	Steroids	Hirsutosterosides A (1) and B(2)	Cytotoxic towards cancer cells	[600]
Anthozoa/Octocorallia	*Dichotella gemmacea*	Steroids	25-acetate-nebrosteroid K	Antifouling activity towards A. Salina	[636]
Anthozoa/Octocorallia	*Klyxum flaccidum*	Steroids	23,24-dimethylated steroids (1 and 2), gorgosteroids(3–5), 9,11-secogorgosteroid (6)	Cytotoxic towards cancer cells, anti-inflammatory	[637]
Anthozoa/Octocorallia	*Menella woodin*	Steroids	Sterol derivatives (1–4)	Anti-inflammatory	[638]
Anthozoa/Octocorallia	*Sinularia acuta*	Steroids	Cyclopentenone 9	Cytotoxic towards cancer cells	[639]
Anthozoa/Octocorallia	*Subergorgia rubra*	Steroids	Subergosterones B and C	Antibacterial	[640]
Anthozoa/Octocorallia	*Clavularia sp.*	Steroids	9,11-secosteroid, haebaruol,	Cytotoxic towards cancer cells.	[641]
Anthozoa/Octocorallia	*Gorgonia sp.*	Steroids	Oxysterols	Anti- leishmanial	[642]
Anthozoa/Octocorallia	*Klyxum flaccidum*	Steroids	Klyflaccisteroids G–J	Cytotoxic towards cancer cells, anti-inflammatory	[643]
Anthozoa/Octocorallia	*Litophyton mollis*	Steroids	4α-methylated steroids	Cytotoxic towards cancer cells	[644]
Anthozoa/Octocorallia	*Nephthea columnaris*	Steroids	Columnaristerol A	Cytotoxic towards cancer cells	[645]
Anthozoa/Octocorallia	*Nephthea erecta*	Steroids	Nephtheasteroid A and B	Cytotoxic towards cancer cells	[646]
Anthozoa/Octocorallia	*Nephthea sp.*	Steroids	Steroid	Cytotoxic towards cancer cells	[647]

Anthozoa/Octocorallia	*Pacifigorgia senta*	Steroids	Cholesta-5,24-diene-3β,7β,19-triol	Cytotoxic towards cancer cells	[648]
Anthozoa/Octocorallia	*Pinnigorgia sp*	Steroids	Pinnigorgiols A-C	Anti-Inflammatory, anti-oxidant	[649]
Anthozoa/Octocorallia	*Pinnigorgia sp*	Steroids	Pinnisterols A–C (1–3),	Cytotoxic towards cancer cells, antioxidant	[649]
Anthozoa/Octocorallia	*Pinnigorgia sp*	Steroids	Pinnigorgiols D and E	Anti-inflammatory	[650]
Anthozoa/Octocorallia	*Pinnigorgia sp.*	Steroids	11-acetoxy-24S-methyl-3β,5α,6α-trihydroxy-9,11-secocholest-7-en-9-one	Pro-inflammatory	[651]
Anthozoa/Octocorallia	*Pinnigorgia sp.*	Steroids	5β,6β-epoxy-(22E,24R)-ergosta-8,22-diene-3β,7β-diol	Pro-inflammatory	[651]
Anthozoa/Octocorallia	*Sinularia microspiculata*	Steroids	7-oxogorgosterol	Cytotoxic towards cancer cells	[652]
Anthozoa/Octocorallia	*Sinularia microspiculata*	Steroids	16α-hydroxysarcosterol	Cytotoxic towards cancer cells	[652]
Anthozoa/Octocorallia	*Sinularia nanolobata*	Steroids	24(S),28-epoxyergost-5-ene-3β,4α-diol (2)	Cytotoxic towards cancer cells	[653]
Anthozoa/Octocorallia	*Sinularia sp.*	Steroids	(22E)-24-methylenecholestane-22-ene-3β,5α,6β-triol	Cytotoxic towards cancer cells	[654]
Anthozoa/Octocorallia	*Subergorgia suberosa*	Steroids	Subergorgols T–X	Antiviral	[655]
Anthozoa/Octocorallia	*Umbellulifera petasites*	Steroids	Petasitosterones A and B	Cytotoxic towards cancer cells	[656]
Anthozoa/Octocorallia	*Umbellulifera petasites*	Steroids	Spirosteroid petasitosterone C	Anti-inflammatory	[656]
Anthozoa/Octocorallia	*Lobophytum sp.*	Steroids	Lobophytene (1)	Cytotoxic towards cancer cells	[657]
Anthozoa/Octocorallia	*Scleronephthya sp.*	Steroids	Scleronine	Inhibition of cell migration	[658]

Anthozoa/Octocorallia	*Pinnigorgia sp.*	Steroids	11-acetoxy-9,11-secosterols, pinnisterols D–J (1–7)	Cytotoxic towards cancer cells; anti-inflammatory	[659]
Anthozoa/Octocorallia	*Carijoa riisei*	Steroids	15β-hydroxypregna-4,20-dien-3-one	Cytotoxic towards cancer cells	[660]
Anthozoa/Octocorallia	*Carijoa riisei*	Steroids	18-acetoxypregna-1,4,20-trien-3-one	Cytotoxic towards cancer cells	[660]
Anthozoa/Octocorallia	*Carijoa riisei*	Steroids	15β-acetoxypregna-1,4,20-trien-3-one	Cytotoxic towards cancer cells	[660]
Anthozoa/Octocorallia	*Carijoa riisei*	Steroids	20R-acetoxypregna-1,4-dien-3-one	Cytotoxic towards cancer cells	[660]
Anthozoa/Octocorallia	*Klyxum flaccidum*	Steroids	Klyflaccisteroids K–M (1–3),	Cytotoxic towards cancer cells	[661]
Anthozoa/Octocorallia	*Lobophytum crassum*	Steroids	24-methylenecholest-5-ene-1α,3β,11α-triol 1-acetate	Cytotoxic towards cancer cells	[662]
Anthozoa/Octocorallia	*Nephthea columnaris*	Steroids	Columnaristerols B and C	Anti-inflammatory	[663]
Anthozoa/Octocorallia	*Nephthea erecta*	Steroids	24-methyl-cholesta-5,24(28)-diene-3 ,19-diol-7 -monoacetate (mecdda)	Cytotoxic towards cancer cells	[664]
Anthozoa/Octocorallia	*Pinnigorgia sp.*	Steroids	5 ,6 -epoxy-(22E,24R)-3 ,11-dihydroxy-9,11-secoergosta-7-en-9-one	Anti-inflammatory	[665]
Anthozoa/Octocorallia	*Pinnigorgia sp.*	Steroids	(22R)-acetoxy-(24x)-ergosta-5-en-3 ,25-diol	Anti-inflammatory	[665]
Anthozoa/Octocorallia	*Pseudopterogorgia americana*	Steroids	Ameristerenol A and B	Cytotoxic towards cancer cells	[666]
Anthozoa/Octocorallia	*Sinularia brassica*	Steroids	Sinubrassione	Cytotoxic towards cancer cells	[667]
Anthozoa/Octocorallia	*Sinularia brassica*	Steroids	Sinubrasones A–D	B-c: Cytotoxic towards cancer cells;<br>C-d: anti-inflammatory	[668]

Anthozoa/Octocorallia	*Sinularia conferta.*	Steroids	Ergosta-24(28)-ene-3β,5α,6β-triol-6-acetate	Cytotoxic towards cancer cells	[669]
Anthozoa/Octocorallia	*Sinularia leptoclados*	Steroids	Leptosteroid; 5,6β-epoxygorgosterol	Cytotoxic towards cancer cells	[670]
Anthozoa/Octocorallia	*Xenia umbellata*	Steroids	3β-,5α-,6β-,11α-,20β-pentahydroxygorgosterol	Antimicrobial	[671]
Anthozoa/Octocorallia	*Sinularia brassica*	Steroids	Sinubrasolides H–L	Anti-inflammatory	[672]
Anthozoa/Octocorallia	*Verrucella corona*	Steroids	Verrucorosterone	Cytotoxic towards cancer cells	[673].
Anthozoa/Octocorallia	*Sinularia* sp	Steroids	Ximaosteroid E, F	cytotoxicity to cancer cell lines	[674]
Anthozoa/Octocorallia	*Lobophytum michaelae*	Steroids	Michosterols A-C	cytotoxicity to cancer cell lines and anti-inflammatory activity	[675]
Anthozoa/Octocorallia	*Dendronephthya gigantea*	Steroids	7-dehydroerectasteroid F	antioxidant	[676]
Anthozoa/Octocorallia	*Sinularia sp*	Steroids	2 ergostane-type sterols	Cytotoxic against human cancer cells	[677]
Anthozoa/Octocorallia	*Carijoa sp.*	Steroids	Carijoside A (1)	Cytotoxic towards cancer cells	[678]
Anthozoa/Octocorallia	*Astrogorgia sp.*	Steroids	Astrogorgols A–N (1–14)	Protein kinases inhibition	[679]
Anthozoa/Octocorallia	*Carijoa multiflora*	Steroids	Carijodienone 1	Antimicrobial	[680]
Anthozoa/Octocorallia	*Dendronephthya mucronata*	Steroids	5-α-pregn-20-en-3,6-dione	Anti-Inflammatory	[681]
Anthozoa/Octocorallia	*Dendronephthya sp.*	Steroids	Dendronesterones D and E	Anti-Inflammatory	[682]
Anthozoa/Octocorallia	*Lobophytum sp.*	Steroids	Lobophysterols D	Cytotoxic against human cancer cells	[683]

Anthozoa/Octocorallia	*Cladiella hirsuta*	Steroids	Hirsutosterols A–G (1–7)	Cytotoxic towards cancer cells	[684]
Anthozoa/Octocorallia	*Isis hippuris*	Steroids	Polyoxygenated steroids (1-6)	Cytotoxic towards cancer cells, Antiviral	[685]
Anthozoa/Octocorallia	*Isis hippuris*	Steroids	Polyoxygenated steroids (1–4)	Cytotoxic towards cancer cells	[686]
Anthozoa/Octocorallia	*Isis hippuris*	Steroids	Hipposterone M–O (1–3)	Antiviral	[687]
Anthozoa/Octocorallia	*Lobophytum laevigatum*	Steroids	Lobophytosterol (1)	Cytotoxic towards cancer cells	[688]
Anthozoa/Octocorallia	*Muricella flexuosa*	Steroids	Muricellasteroids A– E (1 – 5)	Cytotoxic towards cancer cells	[689]
Anthozoa/Octocorallia	*Paraminabea sp.*	Steroids	Methyl-3-oxochola-1,4-dien-24-oate (1)	Cytotoxic towards cancer cells	[690]
Anthozoa/Octocorallia	*Isis minorbrachyblasta*	Steroids	(1a,3b,7a,11a,12b)-gorgost-5-ene-1,3,7,11,12-pentol 12-acetate (1), 11-O-acetyl-22-epihippuristanol (2)	Cytotoxic towards cancer cells	[691]
Anthozoa/Octocorallia	*Lobophytum patulum*	Steroids	Patusterol A and B (2, 3)	Antifouling activity	[692]
Anthozoa/Octocorallia	*Lobophytum sarcophytoides*	Steroids	Sarcophytosterol (1)	Anti-inflammatory	[693]
Anthozoa/Octocorallia	*Menella sp.*	Steroids	Menellin A (1), menellsteroid C (2)	Anti-inflammatory	[694]
Anthozoa/Octocorallia	*Paraminabea acronocephala*	Steroids	Paraminabeolides A-F (1-6)	Cytotoxic towards cancer cells	[695]
Anthozoa/Octocorallia	*Scleronephthya flexilis*	Steroids	3β-methoxy-5,20-pregnadiene (1)	Cytotoxic towards cancer cells	[696]

Anthozoa/Octocorallia	*Scleronephthya gracillimum*	Steroids	Clerosteroids aei (1, 5, 6, 8e13)	Cytotoxic towards cancer cells, anti-inflammatory	[697]
Anthozoa/Octocorallia	*Sinularia crassa*	Steroids	Crassarosterol A (1),	Cytotoxic towards cancer cells	[698]
Anthozoa/Octocorallia	*Sinularia crassa*	Steroids	Crassarosterosides A–D (2–5)	Inti-inflamatory	[698]
Anthozoa/Octocorallia	*Sinularia granosa*	Steroids	New 9,11-secosteroid, 8ah-3b,11-dihydroxy-5a,6a-expoxy-24-methylene-9,11-secocholestan-9-one (1)	Anti-inflammatory	[699]
Anthozoa/Octocorallia	*Sinularia humilis*	Steroids	Sinularosides A and B (1, 2)	Antifungal, antibacterial	[700]
Anthozoa/Octocorallia	*Subergorgia suberosa.*	Steroids	(3b,12b,16b,23E)-cholesta-5,23-diene-3,12,16,20,25-pentaol (1) and (3b,12b,16b)-cholesta-5,25(26)-diene-3,12,16,20,24-pentaol (2)	Cytotoxic towards cancer cells	[701]
Anthozoa/Octocorallia	*Briareum excavatum*	Terpenoids	Excavatoids L—N (1—3)	Anti-inflammatory	[702]
Anthozoa/Octocorallia	*Anthogorgia sp*	Terpenoids	Anthogorgienes A–O (**1**–**15**)	Antifouling	[703]
Anthozoa/Octocorallia	*Anthogorgia sp.*	Terpenoids	Anthogorgiene P (1), anthogorgiene Q (2)	Antifouling	[704]
Anthozoa/Octocorallia	*Astrogorgia*	Terpenoids	Astrogorgins B−M (1−12)	Antifouling	[705]
Anthozoa/Octocorallia	*Briareum asbestinum*	Terpenoids	Seco-briarellinone (1) and briarellin S (2),	Anti-inflammatory	[706]
Anthozoa/Octocorallia	*Briareum asbestinum*	Terpenoids	Riareolate ester K (2)	Antiproliferative	[707]
Anthozoa/Octocorallia	*Briareum excavatum*	Terpenoids	Briacavatolides A–C (1–3)	Anti viral	[708]

Anthozoa/Octocorallia	*Briareum sp.*	Terpenoids	Briarenolides, F (1) and G (2)	Anti inflamatory	[709]
Anthozoa/Octocorallia	*Briareum sp.*	Terpenoids	Briarenolides H (**1**) and I (**2**)	Anti inflamatory	[710]
Anthozoa/Octocorallia	*Briareum sp.*	Terpenoids	Briarenolide E (1),	Anti inflamatory	[711]
Anthozoa/Octocorallia	*Cespitularia sp.*	Terpenoids	Diterpenoids 1–5	Cytotoxic towards cancer cells	[587]
Anthozoa/Octocorallia	*Briareum excavatum*	Terpenoids	Excavatoids O (1) and P (2)	Anti-inflammatory	[712]
Anthozoa/Octocorallia	*Briareum excavatum (accepted Briareum stechei)*	Terpenoids	Excavatoids GK	Anti-inflammatory	[712]**.**
Anthozoa/Octocorallia	*Cespitularia taeniata*	Terpenoids	Verticillanes, cespitulins E–G (1–3)	Anti-inflammatory	[713]
Anthozoa/Octocorallia	*Cladiella sp.*	Terpenoids	Cladieunicellin H (1)	Anti-inflammatory	[714]
Anthozoa/Octocorallia	*Cladiella sp.*	Terpenoids	Cladieunicellin G (1) and 6-epi-cladieunicellin F (2)	Anti-inflammatory	[715]
Anthozoa/Octocorallia	*Dichotella gemmacea*	Terpenoids	Gemmacolides T–Y (1–6)	Cytotoxic towards cancer cells	[716]
Anthozoa/Octocorallia	*Echinomuricea sp*	Terpenoids	Echinoclerodane A (1)	Cytotoxic towards cancer cells, anti-inflammatory	[717]
Anthozoa/Octocorallia	*Echinomuricea sp.*	Terpenoids	Echinohalimane A (1),	Cytotoxic towards cancer cells, anti-inflammatory	[718]
Anthozoa/Octocorallia	*Echinomuricea sp.*	Terpenoids	Echinolabdane A (1)	Cytotoxic towards cancer cells, anti-inflammatory	[719]
Anthozoa/Octocorallia	*Eunicea fusca*	Terpenoids	Eunicidiol (1),	Anti-inflammatory	[720]

Anthozoa/Octocorallia	*Eunicea knighti*	Terpenoids	Knightine (**1**), 11(*R*)-hydroxy-12(20)-en-knightal (**2**), and 11(*R*)-hydroxy-12(20)-en-knightol acetate (**3**)	Antibacterial	[721]
Anthozoa/Octocorallia	*Eunicea sp.*	Terpenoids	Diterpenoids, 1 and 2	Antibacterial	[722]
Anthozoa/Octocorallia	*Briareum polyanthes (accepted as Briareum asbestinum)*	Terpenoids	Briarellins 1-9 and polyanthellin A (10)	Antiparasite	[723]
Anthozoa/Octocorallia	*Junceella juncea*	Terpenoids	Juncenolides M–O (1–3) (briarenes)	Anti-inflamatory	[724]
Anthozoa/Octocorallia	*Klyxum simplex*	Terpenoids	Simplexins P–S (1–4)	Cytotoxic towards cancer cells	[725]
Anthozoa/Octocorallia	*Lobophytum michaelae*	Terpenoids	Michaolides L–Q (**1–6**)	Cytotoxic towards cancer cells	[726]
Anthozoa/Octocorallia	*Lobophytum pauciflorum*	Terpenoids	Cyclolobatriene (1)	Cytotoxic towards cancer cells	[727]
Anthozoa/Octocorallia	*Melitodes squamata*	Terpenoids	(−)-4β-N-methenetauryl-10β-methoxy-1β,5β,6α,7α-aromadendrane (7),	Antibacterial	[728]
Anthozoa/Octocorallia	*Menella sp. and Lobophytum crassum*	Terpenoids	Menelloide E (**1**) and lobocrassin F (**2**)	Anti-inflammatory	[729]
Anthozoa/Octocorallia	*Sarcophyton crassocaule*	Terpenoids	Sarcocrassocolides M–O (1–3)	Cytotoxic towards cancer cells, anti-inflammatory	[730]
Anthozoa/Octocorallia	*Sarcophyton ehrenbergi*	Terpenoids	(+)-12-ethoxycarbonyl-11Z-sarcophine (**1**), ehrenbergol A and B (**2** and **3**).	Cytotoxic towards cancer cells, antiviral	[731]
Anthozoa/Octocorallia	*Sarcophyton glaucum*	Terpenoids	11(S) hydroperoxylsarcoph-12(20)-ene (1), 12(S)-hydroperoxylsarcoph-10-ene (2), 8-epi-sarcophinone (3)	Inhibitors of cytochrome P450, inducers of glutathione S-transferases (GST), quinone	[732]

				reductase (QR), and epoxide hydrolase (meh)	
Anthozoa/Octocorallia	*Sarcophyton sp.*	Terpenoids	Dihydrofuranocembranoids **1** and **2**	Cytotoxic towards cancer cells	[733]
Anthozoa/Octocorallia	*Briareum sp.*	Terpenoids	Briarenolide D (1)	Cytotoxic towards cancer cells	[734]
Anthozoa/Octocorallia	*Sarcophyton trocheliophorum*	Terpenoids	Yalongenes A (**1**) and B (**2**)	Cytoprotective	[735]
Anthozoa/Octocorallia	*Sinularia flexibilis*	Terpenoids	Flexibilisolides C–G (**1–5**), flexibilisin C (**6**), 11,12-secoflexibillin (**7**)	Cytotoxic towards cancer cells, anti-inflammatory	[736]
Anthozoa/Octocorallia	*Sinularia lochmodes.*	Terpenoids	Lochmolins A–G (**1–7**)	Anti-inflamatory	[737]
Anthozoa/Octocorallia	*Sinularia pavida*	Terpenoids	Pavidolides C and D (3,4))	Antifouling	[738]
Anthozoa/Octocorallia	*Sinularia pavida*	Terpenoids	Pavidolides B and C (2, 3)	Cytotoxic towards cancer cells	[738]
Anthozoa/Octocorallia	*Sinularia scabra*	Terpenoids	Scabralins A (**1**) and B (**2**)	Cytotoxic towards cancer cells, anti-inflammatory	[739]
Anthozoa/Octocorallia	*Sinularia sp.*	Terpenoids	Diterpene (1), lobane (2)	Cytotoxic towards cancer cells	[740]
Anthozoa/Octocorallia	*Sinularia sp.*	Terpenoids	5-episinuleptolide acetate (**1**)	Cytotoxic towards cancer cells	[741]
Anthozoa/Octocorallia	*Cladiella hirsuta*	Terpenoids	Hirsutalins A-H (1-8)	Anti-inflammatory	[742]
Anthozoa/Octocorallia	*Cladiella sp.*	Terpenoids	Cladielloides A (1) and B (2)	Cytotoxic towards cancer cells	[742]
Anthozoa/Octocorallia	*Cladiella pachyclados*	Terpenoids	Pachycladins A-E (1-5)	Cytotoxic towards cancer cells	[743]
Anthozoa/Octocorallia	*Sinularia candidula*	Terpenoids	N-[(2S,3R,E)-1,3-dihydroxyhexacos-4-en-2-yl]icosanamide (2), N-[(2S,3S,4R)-1,3,4-	Antiviral	[625]

			trihydroxyhexacosan-2-yl]icosanamide (3), and (R)-2′-hydroxy-N-[(2S,3S,4R)-1,3,4-trihydroxypentacosan-2-yl] nonadecanamide (4)		
Anthozoa/Octocorallia	*Cladiella hirsuta*	Terpenoids	Hirsutalins I–M (**1**–**5**)	Anti-inflammatory	[744]
Anthozoa/Octocorallia	*Cladiella krempfi*	Terpenoids	Krempfielins J–M (**1**–**4**)	Anti-inflammatory	[745]
Anthozoa/Octocorallia	*Cladiella krempfi*	Terpenoids	Krempfielins E–I (1–5)	Cytotoxic towards cancer cells	[746]
Anthozoa/Octocorallia	*Cladiella sp*	Terpenoids	Cladieunicellin I (**1**)	Cytotoxic towards cancer cells	[747]
Anthozoa/Octocorallia	*Cladiella sp*	Terpenoids	Eunicellin-based diterpenoids, cladieunicellins K (1) and L (2)	Cytotoxic towards cancer cells	[748]
Anthozoa/Octocorallia	*Cladiella sp.*	Terpenoids	Australins E–H (1–4)	Activate the inositol 5-phosphatase SHIP1	[749]
Anthozoa/Octocorallia	*Dichotella gemmacea*	Terpenoids	Gemmacolides AA–AR (**1**–**18**) ((11,20-epoxy-3Z,5E-dien briaranes))	Cytotoxic towards cancer cells	[750]
Anthozoa/Octocorallia	*Dichotella gemmacea*	Terpenoids	Dichotellides F–U (**1**–**16**)	Antifouling	[751]
Anthozoa/Octocorallia	*Junceella fragilis*	Terpenoids	Frajunolides P–S (**1**–**4**)	Anti-inflammatory	[752]
Anthozoa/Octocorallia	*Klyxum molle*	Terpenoids	Klymollins I–S (**1**–**11**)	Anti-inflammatory	[753]
Anthozoa/Octocorallia	*Lemnalia philippinensis*	Terpenoids	Philippinlins A and B (**1** and **2**)	Cytotoxic towards cancer cells	[754]
Anthozoa/Octocorallia	*Lobophytum pauciflorum*	Terpenoids	Lobophytones A–G (**1**–**7**	Anti-inflammatory	[755]
Anthozoa/Octocorallia	*Lobophytum sp.*	Terpenoids	7,8-epoxycembranoid (1-4)	Anti-inflammatory	[756]

Anthozoa/Octocorallia	*Lobophytum sp.*	Terpenoids	Cembranoid (1-3)	Cytotoxic towards cancer cells, antibacterial	[757]
Anthozoa/Octocorallia	*Muricella sibogae*	Terpenoids	Sibogin A and B (**1** and **2**)	Cytotoxic towards cancer cells	[758]
Anthozoa/Octocorallia	*Muricella sibogae*	Terpenoids	9,10-secosteroids sibogol A–C (**6**–**8**),	Cytotoxic towards cancer cells	[758]
Anthozoa/Octocorallia	*Paralemnalia thyrsoides*	Terpenoids	Parathyrsoidins A–D (1–4)	Cytotoxic towards cancer cells	[759]
Anthozoa/Octocorallia	*Convexella magelhaenica*	Terpenoids	Dolabellane diterpenoids (1 and 2)	Cytotoxic towards cancer cells	[760]
Anthozoa/Octocorallia	*Rumphella antipathies*	Terpenoids	2β-acetoxyclovan-9α-ol (1), 9α-acetoxyclovan-2β-ol (2)	Anti-inflammatory	[761]
Anthozoa/Octocorallia	*Sarcophyton ehrenbergi*	Terpenoids	Ehrenbergol C and acetyl ehrenberoxide B (1 and 2)	Antiviral	[762]
Anthozoa/Octocorallia	*Sarcophyton elegans*	Terpenoids	Sarcophyolides B–E (**1**–**4**)	Cytotoxic towards cancer cells	[763]
Anthozoa/Octocorallia	*Sarcophyton glaucum*	Terpenoids	Sarglaucol(1)	Cytotoxic towards cancer cells	[764]
Anthozoa/Octocorallia	*Sarcophyton glaucum*	Terpenoids	Diterpene (1S,2E,4R,6E,8S,11R,12S)-8,11-epoxy-4,12-epoxy-2,6-cembradiene (2), diterpene (1S,2E,4R,6E,8R,11S,12R)-8,12-epoxy-2,6-cembradiene-4,11-diol (3) and (1S,4R,13S)-cembra-2E,7E,11E-trien-4,13-diol (6)	Cytotoxic towards cancer cells, antifungal	[765]
Anthozoa/Octocorallia	*Sarcophyton pauciplicatum*	Terpenoids	Sarcophytolides M and N (**1** and **2**)	Cytotoxic towards cancer cells	[766]
Anthozoa/Octocorallia	*Sarcophyton trocheliophorum*	Terpenoids	Arcotroates A and B (**3** and **4**)	Inhibition of protein tyrosine phosphatase 1B (PTP1B)	[767]

Anthozoa/Octocorallia	*Sinularia capillosa*	Terpenoids	Capillosananes A–N (**1–14**)	Antifouling	[768]
Anthozoa/Octocorallia	*Sinularia capillosa*	Terpenoids	Capillosananes B and I and (−)-sinularone A	Anti-inflammatory	[768]
Anthozoa/Octocorallia	*Sinularia flexibilis*	Terpenoids	Flexibilin D (2)	Anti-inflammatory	[769]
Anthozoa/Octocorallia	*Sinularia flexibilis*	Terpenoids	11-acetylsinuflexolide (1)	Cytotoxic towards cancer cells	[770]
Anthozoa/Octocorallia	*Klyxum simplex*	Terpenoids	Klysimplexin sulfoxides A–C (1–3)	Anti-inflammatory activity	[771]**.**
Anthozoa/Octocorallia	*Sinularia gaweli*	Terpenoids	Flexibilin D (2)	Anti-inflammatory	[772]
Anthozoa/Octocorallia	*Sinularia leptoclados*	Terpenoids	Leptoclalin A (**1**)	Cytotoxic towards cancer cells	[773]
Anthozoa/Octocorallia	*Lobophytum crassum*	Terpenoids	Lobocrasols A and B (1 and 2)	Anti-inflammatory	[774]
Anthozoa/Octocorallia	*Sinularia maxima*	Terpenoids	12-hydroxy-scabrolide A (**2)** and 13-*epi*-scabrolide C (**6**)	Anti-inflammatory	[774]
Anthozoa/Octocorallia	*Sinularia rigida*	Terpenoids	Sinulariols T–Z_5 (7)	Antifouling	[775]
Anthozoa/Octocorallia	*Sinularia sp.*	Terpenoids	Sinularcasbanes A and E (2 and 5)	Anti-inflammatory	[776]
Anthozoa/Octocorallia	*Sinularia sp.*	Terpenoids	Sinularianins C–F (**3–6**)	Anti-inflammatory	[777]
Anthozoa/Octocorallia	*Lobophytum crassum*	Terpenoids	Diterpenes (1—4)	Anti-inflammatory	[778]
Anthozoa/Octocorallia	*Lobophytum pauciflorum*	Terpenoids	Lobophytones H—N (1—7)	Anti-inflammatory	[779]
Anthozoa/Octocorallia	*Lobophytum pauciflorum*	Terpenoids	Lobophytones O–S (1–5)	Anti-inflammatory, antibacterial	[779]

Anthozoa/Octocorallia	*Anthogorgia caerulea*	Terpenoids	Anthogonoid A and antsimplexin A (1–2),	Antifouling	[780]
Anthozoa/Octocorallia	*Briareum sp.*	Terpenoids	Briarenolide J (1)	Anti-inflammatory	[781]
Anthozoa/Octocorallia	*Briareum violacea*	Terpenoids	Briaviolides A–J (5 and 9)	Anti-inflammatory	[782]
Anthozoa/Octocorallia	*Cespitularia taeniata*	Terpenoids	Cespitulones A (**1**)	Cytotoxic towards cancer cells	[783]
Anthozoa/Octocorallia	*Cladiella*	Terpenoids	Cladieunicellin J (1)	Cytotoxic towards cancer cells	[784]
Anthozoa/Octocorallia	*Cladiella hirsuta*	Terpenoids	Eunicellin-type hirsutalins N–R (1–5)	Anti-inflammatory	[785]
Anthozoa/Octocorallia	*Cladiella krempfi*	Terpenoids	Krempfielins Q and R (1 and 2)	Anti-inflammatory	[786]
Anthozoa/Octocorallia	*Cladiella krempfi*	Terpenoids	Krempfielins N–P (1–3)	Anti-inflammatory	[787]
Anthozoa/Octocorallia	*Cladiella sp*	Terpenoids	Cladieunicellins M–Q (1–5)	Cytotoxic towards cancer cells	[788]
Anthozoa/Octocorallia	*Lobophytum schoedei*	Terpenoids	Lobophynins A (1), B (2), C (3)	Antifouling activity	[789]
Anthozoa/Octocorallia	*Dendronephthya sp.*	Terpenoids	Dendronephthol A (7,13-dihydroxy-3,4-dihydro-a-ylang-5-one) (1), dendronephthol C (6,7,13-trihydroxy-a-ylang-5-one) (3)(6,7,13-trihydroxy-a-ylang-5-one) (3)	Cytotoxic towards cancer cells	[790]
Anthozoa/Octocorallia	*Dichotella gemmacea*	Terpenoids	Gemmacolides AS-AY (1–7)	Antiproliferative	[791]
Anthozoa/Octocorallia	*Eunicea pinta*	Terpenoids	Pintoxolanes A–C (1 – 3)	Cytotoxic towards cancer cells	[792]
Anthozoa/Octocorallia	*Junceella fragilis*	Terpenoids	Fragilisinins A–L (5,6,10)	Antifouling	[793]

Anthozoa/Octocorallia	*Klyxum molle*	Terpenoids	Klymollins T–X (1–5)	Cytotoxic towards cancer cells, anti-inflammatory	[794]
Anthozoa/Octocorallia	*Klyxum molle*	Terpenoids	Klymollins I–S (1−11),	Anti-inflammatory	[753]
Anthozoa/Octocorallia	*Lobophytum crassum*	Terpenoids	Secocrassumol	Antiviral	[795]
Anthozoa/Octocorallia	*Menella kanisa*	Terpenoids	Menecubebane A (1)	Antifouling	[796]
Anthozoa/Octocorallia	*Muricella sibogae*	Terpenoids	Sibogins B (2)	Antifouling	[797]
Anthozoa/Octocorallia	*Nephthea erecta*	Terpenoids	Kelsoenethiol (1)	Cytotoxic towards cancer cells	[798]
Anthozoa/Octocorallia	*Lobophytum sp.*	Terpenoids	Crassumolide E (3)	Ache Inhibitor	[799]
Anthozoa/Octocorallia	*Pennatula aculeata*	Terpenoids	2-acetoxyverecynarmin C	Anti-inflammatory	[800]
Anthozoa/Octocorallia	*Rumphella antipathies*	Terpenoids	Rumphellols A and B (1 and 2)	Anti-inflammatory	[801]
Anthozoa/Octocorallia	*Rumphella antipathies*	Terpenoids	Rumphellaoic acid A (1)	Anti-inflammatory	[802]
Anthozoa/Octocorallia	*Rumphella antipathies*	Terpenoids	Rumphellaones B (1) and C (2)	Anti-inflammatory	[803]
Anthozoa/Octocorallia	*Sarcophyton auritum*	Terpenoids	2-epi-sarcophine (2) and (1R,2E,4S,6E,8R,11R,12R)-2, 6-cembradiene-4,8,11,12-tetrol (4)	Cytotoxic towards cancer cells	[804]
Anthozoa/Octocorallia	*Sarcophyton crassocaule*	Terpenoids	Sarcocrassocolides P–R (1–3)	Anti-inflammatory	[805]
Anthozoa/Octocorallia	*Sarcophyton ehrenbergi*	Terpenoids	7-keto-8α-hydroxy-deepoxysarcophine, 7β-chloro-8α-hydroxy-12-acetoxy-deepoxysarcophine,(E)-	Cytotoxic towards cancer cells	[806]

			methyl-3-(5-butyl-1-hydroxy-2,3-dimethyl-4-oxocyclopent-2-enyl) acrylate		
Anthozoa/Octocorallia	*Sarcophyton glaucum*	Terpenoids	Sarcophytolol (1), sarcophytolide B (2), and sarcophytolide C (3)	Cytotoxic towards cancer cells	[807]
Anthozoa/Octocorallia	*Sarcophyton sp.*	Terpenoids	Sarcophyton A, sarcophyton B, sarcophyton C, sarcophyton D	Cytotoxic towards cancer cells	[808]
Anthozoa/Octocorallia	*Sarcophyton tortuosum*	Terpenoids	Tortuosenes A and B (1 and 2)	Anti-inflammatory	[809]
Anthozoa/Octocorallia	*Paralemnalia thyrsoides*	Terpenoids	Paralemnolins J—O (1—6)	Cytotoxic towards cancer cells	[810]
Anthozoa/Octocorallia	*Sarcophyton trocheliophorum*	Terpenoids	Sarsolilides B (**5**)	Inhibitory activity towards protein tyrosine phosphatases 1B (PTP1B)	[811]
Anthozoa/Octocorallia	*Sarcophyton trocheliophorum*	Terpenoids	Sarsolilides B (5)	Inhibitory towards protein tyrosine phosphatases 1B (PTP1B)	[811]
Anthozoa/Octocorallia	*Sinularia arborea*	Terpenoids	Sinularbols A (1) and B (2)	Anti-inflammatory	[812]
Anthozoa/Octocorallia	*Sinularia gyrosa*	Terpenoids	Sinugyrosanolide A	Cytotoxic towards cancer cells	[813]
Anthozoa/Octocorallia	*Sinularia sp.*	Terpenoids	Sinulariaoid A (1), sinulariaoid B (2), sinulariaoid C (3), sinulariaoid D (4)	Cytotoxic towards cancer cells	[814]
Anthozoa/Octocorallia	*Xenia elongata*	Terpenoids	New diterpenes (**2**, **3**)	Apoptosis induction	[815]
Anthozoa/Octocorallia	*Capnella sp.*	Terpenoids	Capgermacrenes A (1)	Anti-inflamatory	[816]
Anthozoa/Octocorallia	*Paralemnalia thyrsoides*	Terpenoids	(1S,2S,4R,6S,7R,8S)-4a-formyloxy-b -ylangene (1)	Anti-inflammatory	[817]

Anthozoa/Octocorallia	*Briareum sp*	Terpenoids	Briarenolides K (1) and L (2)	Anti-inflammatory	[818]
Anthozoa/Octocorallia	*Briareum sp.*	Terpenoids	Briarenolides U–Y (1–5)	Anti-inflammatory	[819]
Anthozoa/Octocorallia	*Cespitularia taeniata*	Terpenoids	Cespilamide E (7)	Cytotoxic towards cancer cells.	[820]
Anthozoa/Octocorallia	*Cespitularia taeniata*	Terpenoids	Cespitaenins A (6)	Cytotoxic towards cancer cells	[820]
Anthozoa/Octocorallia	*Litophyton arboreum*	Terpenoids	3a,6a-epidioxyhimachal-1-ene (1), 22-norerogostane derivative, 13,14-seco-22-norergosta-4,24(28)-dien-19-hydroperoxide-3-one (2)	Antiproliferative	[821]
Anthozoa/Octocorallia	*Litophyton arboreum*	Terpenoids	Di-acetyl cembranoid derivative (3)	Antiproliferative	[821]
Anthozoa/Octocorallia	*Litophyton arboreum*	Terpenoids	(3a,6a-epidioxyhimachal-1-ene (1)	Antiproliferative	[821]
Anthozoa/Octocorallia	*Nephthea columnaris*	Terpenoids	Columnariols A (1) and B (2)	Cytotoxic towards cancer cells	[822]
Anthozoa/Octocorallia	*Plumigorgia terminosclera*	Terpenoids	Plumisclerin A (1)	Cytotoxic towards cancer cells	[823]
Anthozoa/Octocorallia	*Sarcophyton auritum*	Terpenoids	N-((2S,3R,4E,6E)-1,3-dihydroxyhenicosa-4,6-dien-2-yl)tridecanamide (1)	Agonistic activity on GABA-A receptors	[824]
Anthozoa/Octocorallia	*Sarcophyton ehrenbergi*	Terpenoids	(+)-2-epi-12-methoxycarbonyl-11E-sarcophine (2)	Antiviral	[825]
Anthozoa/Octocorallia	*Sarcophyton ehrenbergi*	Terpenoids	3,4-epoxyehrenberoxide A (3), ehrenbergol D (4) and ehrenbergol E (5)	Cytotoxic towards cancer cells	[825]
Anthozoa/Octocorallia	*Sarcophyton glaucum*	Terpenoids	A,β-unsaturated ε-lactone, Glaucumolides A (1) and B (2)	Cytotoxic towards cancer cells, anti-inflammatory	[826]

Anthozoa/Octocorallia	*Sarcophyton glaucum*	Terpenoids	6-oxo-germacra-4(15),8,11-triene	Cytotoxic towards cancer cells	[827]
Anthozoa/Octocorallia	*Sarcophyton pauciplicatum*	Terpenoids	Sarcophytolides M	Cytotoxic towards cancer cells	[828]
Anthozoa/Octocorallia	*Sarcophyton trocheliophorum*	Terpenoids	Sarcotrocheliol (2)	Antibacterial	[829]
Anthozoa/Octocorallia	*Sinularia flexibilis*	Terpenoids	Flexibilide	Cytotoxic towards cancer cells	[830]
Anthozoa/Octocorallia	*Sinularia gaweli*	Terpenoids	Sinulacembranolide A (4)	Anti-inflamatory	[831]
Anthozoa/Octocorallia	*Sinularia sandensis*	Terpenoids	Sandensone A	Cytotoxic towards cancer cells	[832]
Anthozoa/Octocorallia	*Sinularia numerosa*	Terpenoids	Sinumerolide A (1), 7E-sinumerolide A (2)	Anti inflammatory	[833]
Anthozoa/Octocorallia	*Pseudopterogorgia acerosa*	Terpenoids	15-chlorodeoxypseudopterolide (**1**)	Cytotoxic towards cancer cells	[834]
Anthozoa/Octocorallia	*Sinularia sandensis and Sinularia f lexibilis*	Terpenoids	Isosinulaflexiolide K (9)	Anti-inflammatory	[835]
Anthozoa/Octocorallia	*Subergorgia suberosa*	Terpenoids	Epoxysubergorgic acid	Antimicrobial	[836]
Anthozoa/Octocorallia	*Xenia sp.*	Terpenoids	Xenicanes	Cytotoxic towards cancer cells, anti-inflammatory	[837]
Anthozoa/Octocorallia	*Cladiella hirsuta*	Terpenoids	Hirsutocospiro A (1)	Anti-inflammatory activity	[838]
Anthozoa/Octocorallia	*Sarcophyton crassocaule*	Terpenoids	Sarcocrassocolides A–E (1–5), cembranoids (6–8)	Cytotoxic activity, Anti-inflammatory	[839]

Anthozoa/Octocorallia	*Sarcophyton ehrenbergi*	Terpenoids	Cembranoids (1), lobophynin C (2), ehrenberoxides A-C (4-6)	Antiviral activity towards human cytomegalovirus	[840]
Anthozoa/Octocorallia	*Briareum sp*	Terpenoids	Briarenolides ZI–ZVI	Pro-inflammatory	[841]
Anthozoa/Octocorallia	*Briareum sp.*	Terpenoids	Briarenolides M-T	Pro-inflammatory	[842]
Anthozoa/Octocorallia	*Cladiella tuberculosa*	Terpenoids	Cladieunicellins R and S,	Cytotoxic towards cancer cells	[843]
Anthozoa/Octocorallia	*Sarcophyton infundibuliforme*	Terpenoids	Sarcolactone A (1)	Antifouling activity	[844]
Anthozoa/Octocorallia	*Dichotella gemmacea*	Terpenoids	Gemmacolides AZ–BF	Cytotoxic towards cancer cells, antibacterial	[591]
Anthozoa/Octocorallia	*Eunicea succinea*	Terpenoids	Uprolide N, O, P	Anti-inflammatory	[845]
Anthozoa/Octocorallia	*Gersemia fruticosa*	Terpenoids	Gersemiols A−C	Antibacterial	[846]
Anthozoa/Octocorallia	*Gersemia fruticosa*	Terpenoids	Eunicellol A	Antibacterial	[846]
Anthozoa/Octocorallia	*Heteroxenia ghardaqensis*	Terpenoids	2S,3R-4E,8E-2-(hexadecanoylamino)-docosa-4,8-diene-1,3-diol	Cytotoxic towards cancer cells	[847]
Anthozoa/Octocorallia	*Klyxum molle*	Terpenoids	Klymollins Y and Z	Cytotoxic towards cancer cells, anti-inflammatory	[848]
Anthozoa/Octocorallia	*Klyxum molle*	Terpenoids	Klyxumollins A-D	Cytotoxic towards cancer cells, anti-inflammatory	[848]
Anthozoa/Octocorallia	*Lobophytum crassum*	Terpenoids	Locrassumins A–G; (–)-laevigatol B; (–)-isosarcophine; (–)-7R,8S-dihydroxydeepoxysarcophytoxide	Anti-inflammatory	[849]

Anthozoa/Octocorallia	*Lobophytum sp*	Terpenoids	Casbane-type diterpenoid 1, diterpenoids 2, 3	Cytotoxic towards cancer cells, antibacterial, anti-inflammatory	[850]
Anthozoa/Octocorallia	*Menella sp.*	Terpenoids	Menecubebane B	Cytotoxic towards cancer cells	[851]
Anthozoa/Octocorallia	*Nephthea sp.*	Terpenoids	10-hydroxy-nephthenol acetate	Cytotoxic towards cancer cells, antibacterial	[852]
Anthozoa/Octocorallia	*Nephthea sp.*	Terpenoids	7,8-epoxy-10-hydroxy-nephthenol acetate	Cytotoxic towards cancer cells, antibacterial	[852]
Anthozoa/Octocorallia	*Nephthea sp.*	Terpenoids	6-acetoxy-7,8-epoxy-10-hydroxy-nephthenol acetate	Cytotoxic towards cancer cells, antibacterial	[852]
Anthozoa/Octocorallia	*Pseudopterogogia rigida*	Terpenoids	Bisabolanes (1, 2, 9-11, 13 and 14),	Inhibitory activity towards CDC25 phosphatases,	[853]
Anthozoa/Octocorallia	*Pseudopterogogia rigida*	Terpenoids	Cadinanes (15-20)	Inhibitory activity towards CDC25 phosphatases	[853]
Anthozoa/Octocorallia	*Sinularia capillosa*	Terpenoids	Capilloquinone (1), capillobenzopyranol (2) and capillobenzofuranol (3),apillofuranocarboxylate (4)	Cytotoxic towards cancer cells	[854]
Anthozoa/Octocorallia	*Sarcophyton sp*	Terpenoids	16-hydroxycembra-1,3,7,11-tetraene	Antibacterial	[855]
Anthozoa/Octocorallia	*Sarcophyton trocheliophorum*	Terpenoids	Sarcotrocheldiol A - B	Antimicrobial	[856]
Anthozoa/Octocorallia	*Sarcophyton trocheliophorum*	Terpenoids	Trocheliane	Antimicrobial	[856]
Anthozoa/Octocorallia	*Sinularia erecta.*	Terpenoids	Cembranoid sinulerectadione	Antiproliferative	[857]
Anthozoa/Octocorallia	*Sinularia erecta.*	Terpenoids	Norcembranoids sinulerectols A and B	Anti-inflammatory	[857]
Anthozoa/Octocorallia	*Sinularia erecta.*	Terpenoids	Cembranoid sinulerectol C	Antiproliferative	[857]

Anthozoa/Octocorallia	*Sinularia inelegans*	Terpenoids	Pambanolides A-C; 4,5-secosinulochmodin C	Cytotoxic towards cancer cells	[858]
Anthozoa/Octocorallia	*Sinularia mollis*	Terpenoids	Mollisolactones A and B	Cytotoxic towards cancer cells, antiviral	[859]
Anthozoa/Octocorallia	*Sinularia sp*	Terpenoids	Endoperoxide (1) and hydroperoxides (2, 3)	Antibacterial	[860]
Anthozoa/Octocorallia	*Sinularia sp*	Terpenoids	Prenyl-α-elemenone and ent-prenyl-β-elemene	Antibacterial	[861]
Anthozoa/Octocorallia	*Sinularia sp.*	Terpenoids	Peroxy sesquiterpenoids	Cytotoxic towards cancer cells	[862]
Anthozoa/Octocorallia	*Subergorgia suberosa*	Terpenoids	Suberosoid	Cytotoxic towards cancer cells	[863]
Anthozoa/Octocorallia	*Sinularia depressa*	Terpenoids	Depressin (5-13)	Cytotoxic towards cancer cells	[864]
Anthozoa/Octocorallia	*Clavularia viridis*	Terpenoids	Clavuridins A and B	Cytotoxic towards cancer cells	[865]
Anthozoa/Octocorallia	*Sinularia flexibilis*	Terpenoids	Thioflexibilolide A (1)	Anti-inflammatory, Neuroprotective	[866]
Anthozoa/Octocorallia	*Sinularia gyrosa*	Terpenoids	Gyrosanols A-C (1-3)	Cytotoxic towards cancer cells	[867]
Anthozoa/Octocorallia	*Xenia umbellata*	Terpenoids	Xeniumbellal	Cytotoxic towards cancer cells, antimicrobial	[671]
Anthozoa/Octocorallia	*Briareum excavatum*	Terpenoids	Briarenol B	Pro-inflammatory	[868]
Anthozoa/Octocorallia	*Sarcophyton elegans*	Terpenoids	Sarelengans A and B (1 and 2),	Inhibitory effects on the NO production in LPS-induced macrophages	[868]
Anthozoa/Octocorallia	*Briareum excavatum*	Terpenoids	Briarenols C–E	Pro-inflammatory	[869]

Anthozoa/Octocorallia	*Capnella fungiformis*	Terpenoids	Ethyl 5-[(1E,5Z)-2,6-dimethylocta-1,5,7-trienyl]furan-3-carboxylate (6)	Anti-parasite	[870]
Anthozoa/Octocorallia	*Capnella fungiformis*	Terpenoids	Oxyfungiformin (9a)	Anti-parasite	[870]
Anthozoa/Octocorallia	*Sarcophyton stellatum*	Terpenoids	(1E,3E,11E)-7,8-epoxycembra-1,3,11,15-tetraene (2)	Anti-parasite	[870]
Anthozoa/Octocorallia	*Cespitularia stolonifera*	Terpenoids	2S, 3R-4E, 8E-2-(heptadecanoylamino)-heptadeca-4, 8-diene-1, 3-diol (1)	Cytotoxic towards cancer cells	[871]
Anthozoa/Octocorallia	*Euplexaura sp.*	Terpenoids	Euplexaurenes A–C (1–3)	Cytotoxic towards cancer cells	[872]
Anthozoa/Octocorallia	*Junceella fragilis*	Terpenoids	Briarane diterpenoids fragilolides B-Q (2-17)	Antiviral	[873]
Anthozoa/Octocorallia	*Junceella fragilis*	Terpenoids	Frajunolide H	Cytotoxic towards cancer cells	[873]
Anthozoa/Octocorallia	*Junceella fragilis*	Terpenoids		Anti-inflammatory	[874]
Anthozoa/Octocorallia	*Klyxum flaccidum*	Terpenoids	Klyflaccicembranols A–I (1-9)	Cytotoxic towards cancer cells, anti-inflammatory	[875]
Anthozoa/Octocorallia	*Sinularia gyrosa*	Terpenoids	Gyrosanolides A–F (1–6), gyrosanin A (7)	Anti-inflammatory	[876]
Anthozoa/Octocorallia	*Lobophytum crassum*	Terpenoids	Lobophyolide A (1) and B (2),	Anti-inflammatory	[877]
Anthozoa/Octocorallia	*Lobophytum varium*	Terpenoids	Diterpenoids (6–12)	Anti-inflammatory	[878]
Anthozoa/Octocorallia	*Lobophytum varium*	Terpenoids	Lobovarols A–D (1–4)	Anti-inflammatory	[878]
Anthozoa/Octocorallia	*Lobophytum varium*	Terpenoids	Lobovarol E (5)	Anti-inflammatory	[878]
Anthozoa/Octocorallia	*Muriceides collaris*	Terpenoids	Bis-sesquiterpene muriceidone A (4)	Cytotoxic towards cancer cells	[879]

Anthozoa/Octocorallia	*Nephthea chabroli Paralemnalia thyrsoides*	Terpenoids	Parathyrsoidins E-G (2-4)	Cytotoxic towards cancer cells	[880]
Anthozoa/Octocorallia	*Nephthea chabroli Paralemnalia thyrsoides*	Terpenoids	Chabrolin A	Cytotoxic towards cancer cells	[880]
Anthozoa/Octocorallia	*Sarcophyton ehrenbergi*	Terpenoids	Sarcoehrenbergilid A–C (1–3),	Anti-proliferation	[881]
Anthozoa/Octocorallia	*Sarcophyton glaucum*	Terpenoids	10(14)-aromadendrene (1), sarcophinediol (2), ent-deoxysarcophine (3) and sarcotrocheliol acetate (4).	Cytotoxic towards cancer cells	[882]
Anthozoa/Octocorallia	*Sarcophyton glaucum*	Terpenoids	3,4,8,16-tetra-epi-lobocrasol, 1,15 -epoxy-deoxysarcophine, 3,4-dihydro-4 ,7 ,8 -trihydroxy-D2-sarcophine, ent-sarcophyolide E, 16-deacetylhalicrasterol B	Cytotoxic towards cancer cells	[883]
Anthozoa/Octocorallia	*Sinularia flexibilis*	Terpenoids	Epoxycembrane A (1), sinularin (2), sinulariolide (3),(1R,13S,12S,9S,8R,5S,4R)-9-acetoxy-5,8:12,13-diepoxycembr-15(17)-en-16,4-olide (4), 11-dehydrosinulariolide (5), ( )14-deoxycrassin (6) and dihydrosinularin (7).	Antifouling	[884]
Anthozoa/Octocorallia	*Sinularia polydactyla*	Terpenoids	Xishacorenes A–C	Cona-induced T lymphocyte proliferation	[885]
Anthozoa/Octocorallia	*Sinularia leptoclados*	Terpenoids	Leptocladolins A and B (1 and 2)	Anti-inflammatory	[886]
Anthozoa/Octocorallia	*Protodendron repens*	Terpenoids	Protoxenicins A -B	Cytotoxic towards cancer cells	[887]

Anthozoa/Octocorallia	*Junceella fragilis*	Terpenoids	Briaranes : fragilides K, L	anti-inflammatory activity	[888]
Anthozoa/Octocorallia	*leptogorgia sp.*	Terpenoids	Two chloro-furanocembranolides (**1**, **2**), two 1,4-diketo cembranolides (**3**, **4**) and a seco-furanocembranolide (5)	anti-diabete	[889]
Anthozoa/Octocorallia	*Sarcophyton infundibuliforme*	Terpenoids	Nitrogenous diterpenoids: sarinfacetamides A (1) and B (2)	induce lymphocyte proliferation	[890]
Anthozoa/Octocorallia	*Sarcophyton glaucom*	Terpenoids	Sarcophinone (**2a**), 8-*epi*-sarcophinone (**2b**), (+)-7*α*,8*β*-dihydroxydeepoxysarcophine (**3**), sinumaximol G (**4**), (+)-sarcophine (**5**), sesquiterpene; prostantherol (**6**), sterol	potent–moderate cytotoxicity	[891]
Anthozoa/Octocorallia	*Sarcophyton cherbonnieri*	Terpenoids	Cherbonolides A-E and bischerbolide peroxide	anti-inflammatory activity	[892]
Anthozoa/Octocorallia	*Sarcophyton mililatensis*	Terpenoids	Sarcomililatins A	potential anti-inflammatory and anti-cancer activity	[893]
Anthozoa/Octocorallia	*Cladiella krempfi*	Terpenoids	8-n-butyryl-litophynol A, 6-keto-litophynol B and 6-epi-litophynol B	moderate anti-inflammatory activity	[894]
Anthozoa/Octocorallia	*Sinularia* sp	Terpenoids	Sinuketal, sinulins A, B,	sinbuketal: mild anti malarial activity	[895]
Anthozoa/Octocorallia	*Sinularia* sp	Terpenoids	Sinulins C,D	sinulin D: mild target inhibitory activity against PTP1B	[895]
Anthozoa/Octocorallia	*Echinogorgia pseudossapo*	Terpenoids	3β-methoxyguaian-10(14)-en-2β-ol (**7**)	Antifouling	[592]
Anthozoa/Octocorallia	*Sinularia* sp	Terpenoids	Sinularolide	potential anti-inflammatory activity	[896]

Anthozoa/Octocorallia	*Sinularia flexibilis*	Terpenoids	Flexibilisins D and E, secoflexibilisolides A and B and flexibilisolide H	Anti-inflammatory	[897]
Anthozoa/Octocorallia	*Sinularia erecta*	Terpenoids	3β,5α-dihydroxyeudesma-4(15),11-diene (1)	cytotoxicity	[898]
Anthozoa/Octocorallia	*Sinularia* cf. *molesta*	Terpenoids	Molestin E	cytotoxic activity	[899]
Anthozoa/Octocorallia	*Sinularia* cf. *molesta*	Terpenoids	Molestin C, D	inhibitory activity against protein tyrosine phosphatase 1B (PTP1B)	[899]
Anthozoa/Octocorallia	*Sinularia polydactyla*	Terpenoids	Sinupol	inhibitory activity against protein tyrosine phosphatase 1B (PTP1B)	[900]
Anthozoa/Octocorallia	*Sinularia polydactyla*	Terpenoids	Sinulacetate	inhibitory activity against protein tyrosine phosphatase 1B (PTP1B)	[900]
Anthozoa/Octocorallia	*Briareum violaceum*	Terpenoids	Briaviolides K-N	potential* anti-inflammatory	[901]
Anthozoa/Octocorallia	*Clavularia viridis*	Terpenoids	Dolabellane diterpene	cytotoxicity to cancer cell lines	[608]
Anthozoa/Octocorallia	*Litophyton arboreum*	Terpenoids	Litopharbol	antibacterial activity	[902]
Anthozoa/Octocorallia	*Dendronephthya gigantea*	Terpenoids	Stigmast-5-en-3-ol	antiproliferative effects on HL-60 (leukemia) and MCF-7 (breast cancer)	[903]
Anthozoa/Octocorallia	*Clavularia viridis*	Terpenoids	Isobromolaureniso	NF-κB inhibition	[904]
Anthozoa/Octocorallia	*Clavularia viridis*	Terpenoids	Clalaurenol A	NF-κB inhibition	[904]
Anthozoa/Octocorallia	*Clavularia viridis/Lemnalia flava*	Terpenoids	Clalaurenol B	NF-κB inhibition	[904]
Anthozoa/Octocorallia	*Clavularia koellikeri*	Terpenoids	Clavukoellians A–F	Anti-angiogenic	[905]

Anthozoa/Octocorallia	*Clavularia koellikeri*	Terpenoids	4-O-deacetylparalemnolin D	Anti-angiogenic	[905]
Anthozoa/Octocorallia	*Briareum violaceum*	Terpenoids	Briaviodiols B, D, E	Anti-inflammatory	[906]
Anthozoa/Octocorallia	*Briareum violaceum*	Terpenoids	Briaviotriols A-B	Anti-inflammatory	[907]
Anthozoa/Octocorallia	*Klyxum flaccidum*	Terpenoids	Flaccidenols A-B	Cytotoxic against human cancer cells	[908]
Anthozoa/Octocorallia	*Lobophytum sp.*	Terpenoids	Cembranolides	Cytotoxic against human cancer cells	[677]
Anthozoa/Octocorallia	*Nephthea sp.*	Terpenoids	Nephthecrassocolides A	Anti-fungal	[909]
Anthozoa/Octocorallia	*Pseudoplexaura flagellosa*	Terpenoids	Asperdiol stereoisomer	Cytotoxic against human cancer cells	[910]
Anthozoa/Octocorallia	*Sarcophyton sp.*	Terpenoids	Sarcophytonolide V	Anti-fungal	[911]
Anthozoa/Octocorallia	*Sarcophyton sp.*	Terpenoids	Sarchophine-like	Cytotoxic against human cancer cells	[912]
Anthozoa/Octocorallia	*Sarcophyton ehrenbergi*	Terpenoids	Sarcoehrenolide A,B,D	Anti-Inflammatory	[913]
Anthozoa/Octocorallia	*Sarcophyton mililatensis*	Terpenoids	Sarcomililate A	Immunosuppressive	[914]
Anthozoa/Octocorallia	*Sinularia sp.*	Terpenoids	2 cembranoid-type diterpenoids	Anti-Aβ aggregation	[915]
Anthozoa/Octocorallia	*Sinularia flexibilis*	Terpenoids	Xidaosinularides A	Anti-Inflammatory	[916]
Anthozoa/Octocorallia	*Sinularia flexibilis*	Terpenoids	Sinulaflexiolide P	Anti-fungal	[917]

Anthozoa/Octocorallia	*Sinularia scabra*	Terpenoids	Xiguscabrates B	Immunosuppressive	[918]
Anthozoa/Octocorallia	*Sinularia scabra*	Terpenoids	Xiguscabrols A and B	Immunosuppressive	[918]
Anthozoa/Octocorallia	*Sinularia scabra*	Terpenoids	8-epi-xiguscabrol B	Immunosuppressive	[918]
Anthozoa/Octocorallia	*Sarcophyton trocheliophorum*	Terpenoids	Bistrochelides B and C	Immunomodulatory	[919]
Anthozoa/Octocorallia	*Anthoptilum grandifloru*	Terpenoids	Bathyptilone A	Cytotoxic against human cancer cells	[920]
Anthozoa/Octocorallia	*Briareum violaceum*	Terpenoids	Briaviolide S	Anti-Inflammatory	[921]
Anthozoa/Octocorallia	*Briareum violaceum*	Terpenoids	Briaviolide V	Anti-Inflammatory	[921]
Anthozoa/Octocorallia	*Junceella fragilis*	Terpenoids	Fragilide S	Anti-Inflammatory	[922]
Anthozoa/Octocorallia	*Junceella fragilis*	Terpenoids	Fragilide W	Anti-Inflammatory	[923]
Anthozoa/Octocorallia	*Cladiella sp.*	Terpenoids	Cladieunicellin U	Anti-Inflammatory	[924]
Anthozoa/Octocorallia	*Cladiella sp.*	Terpenoids	Cladieunicellin X	Anti-Inflammatory	[925]
Anthozoa/Octocorallia	*Klyxum flaccidum*	Terpenoids	Klyflaccilide A	Anti-Inflammatory	[926]
Anthozoa/Octocorallia	*Xenia sp.*	Terpenoids	12-epi-9-deacetoxyxenicin	Cytotoxic against human cancer cells	[927]
Anthozoa/Octocorallia	*Subergorgia suberosa*	Terpenoids	Subergorgic acid	Antifouling	[928]
Anthozoa/Octocorallia	*Sinularia gibberosa*	Terpenoids	Flaccidoxide-13-acetate	Cytotoxic against human cancer cells	[929]

Anthozoa/Octocorallia	*Asterospicularia laurae*	Terpenoids	Asterolaurins K–M(1–3)	Cytotoxic towards cancer cells	[930]
Anthozoa/Octocorallia	*Briareum asbestinum*	Terpenoids	Briareolate esters L- N (1-3)	Antiproliferative	[931]
Anthozoa/Octocorallia	*Briareum sp.*	Terpenoids	Brialalepolides A (1), B (2), and C (3)	Cytotoxic towards cancer cells	[932]
Anthozoa/Octocorallia	*Cladiella krempfi*	Terpenoids	Krempfielins A–D (1–4)	Anti-inflammatory	[933]
Anthozoa/Octocorallia	*Cladiella sp.*	Terpenoids	Cladielloides C (**1**) and D (**2**)	Cytotoxic towards cancer cells, Anti-inflammatory	[934]
Anthozoa/Octocorallia	*Cladiella sp.*	Terpenoids	Cladieunicellin F (1), (–)-solenopodin C (2)	Anti-inflammatory	[935]
Anthozoa/Octocorallia	*Cladiella sp.*	Terpenoids	Cladieunicellins A—E (1—5)	Cytotoxic towards cancer cells, anti-inflammatory	[936]
Anthozoa/Octocorallia	*Dichotella gemmacea*	Terpenoids	Gemmacolides N–S (1–6)	Cytotoxic towards cancer cells, antimicrobial	[937]
Anthozoa/Octocorallia	*Dichotella gemmacea*	Terpenoids	Gemmacolides G-M (1- 7)	Cytotoxic towards cancer cells, antimicrobial	[938]
Anthozoa/Octocorallia	*Cladiella krempfi*	Terpenoids	Oxylitophynol (**5**), litophynol A acetate (**6**), litophynol C (**8**)	Cytotoxic towards cancer cells	[939]
Anthozoa/Octocorallia	*Dichotella gemmacea*	Terpenoids	Dichotellides A - E (1-5)	Cytotoxic towards cancer cells	[939]
Anthozoa/Octocorallia	*Dichotella gemmacea*	Terpenoids	Dichotellates A (**1**)	Antifouling	[940]
Anthozoa/Octocorallia	*Dichotella gemmacea*	Terpenoids	Dichotellates B (2)	Anti-fouling	[940]
Anthozoa/Octocorallia	*Eunicea fusca*	Terpenoids	Fuscoside E	Anti-inflammatory and antifouling activity	[941]

Anthozoa/Octocorallia	*Eunicea sp.*	Terpenoids	Cembradiene diterpenoid 1	Antiplasmodial	[942]
Anthozoa/Octocorallia	*Junceella fragilis*	Terpenoids	Frajunolides L–O (1–4)	Anti-inflammatory	[943]
Anthozoa/Octocorallia	*Junceella juncea*	Terpenoids	8-hydroxy briarane diterpenoid (compound 1)	Antifungal activity	[944]
Anthozoa/Octocorallia	*Klyxum molle*	Terpenoids	Klymollins A−H (1−8)	Anti-inflammatory	[945]
Anthozoa/Octocorallia	*Klyxum simplex*	Terpenoids	Klysimplexins I–T (1–12)	Anti-inflammatory	[946]
Anthozoa/Octocorallia	*Klyxum simplex.*	Terpenoids	Simplexins J–O (**1**–**6**)	Anti-inflammatory	[947]
Anthozoa/Octocorallia	*Lemnalia flava*	Terpenoids	Flavalins A-D (1-4)	Anti-inflammatory, neuroprotective	[948]
Anthozoa/Octocorallia	*Lobophytum compactum*	Terpenoids	Lobocompactols A (1) and B (2)	Cytotoxic towards cancer cells	[949]
Anthozoa/Octocorallia	*Lobophytum crassum*	Terpenoids	Lobocrassins A–E (1–5),	Cytotoxic towards cancer cells, anti-inflammatory	[950]
Anthozoa/Octocorallia	*Lobophytum crassum*	Terpenoids	13-acetoxysarcophytoxide (4)	Cytotoxic towards cancer cells, anti-inflammatory	[951]
Anthozoa/Octocorallia	*Lobophytum crassum*	Terpenoids	Culobophylins A–C (1–3)	Cytotoxic towards cancer cells	[952]
Anthozoa/Octocorallia	*Lobophytum cristatum Tixier-Durivault*	Terpenoids	Lobophytumins C and D (3 and 4)	Cytotoxic towards cancer cells	[953]
Anthozoa/Octocorallia	*Lobophytum durum*	Terpenoids	Durumolides M–Q (1–5)	Cytotoxic towards cancer cells	[954]
Anthozoa/Octocorallia	*Lobophytum durum*	Terpenoids	Durumolides M–Q (1–5)	Antiviral	[954]

Anthozoa/Octocorallia	*Lobophytum laevigatum*	Terpenoids	Laevigatol A–D (1–4)	Anti-inflammatory	[955]
Anthozoa/Octocorallia	*Lobophytum pauciflorum*	Terpenoids	Lobophytones U–Z_1 (1–7)	Anti-inflammatory	[956]
Anthozoa/Octocorallia	*Lobophytum pauciflorum*	Terpenoids	Lobophytones U–Z_1 (1–7)	Antimicrobial	[956]
Anthozoa/Octocorallia	*Menella sp.*	Terpenoids	Menelloide D (2)	Anti-inflammatory	[957]
Anthozoa/Octocorallia	*Menella sp.*	Terpenoids	Menelloide C (1)	Antiinflamatory	[957]
Anthozoa/Octocorallia	*Menella sp.*	Terpenoids	Menelloide B (2)	Anti-inflammatory	[958]
Anthozoa/Octocorallia	*Menella sp.*	Terpenoids	Menelloide A (1)	Antiinflamatory	[958]
Anthozoa/Octocorallia	*Menella sp.*	Terpenoids	(þ)-chloranthalactone B (3)	Anti-oxidative	[958]
Anthozoa/Octocorallia	*Menella sp. (Plexauridae)*	Terpenoids	(-)-Hydroxylindestrenolide (1)	Anti-inflammatory	[959]
Anthozoa/Octocorallia	*Paralemnalia thyrsoides*	Terpenoids	Paralemnolins Q–U (1–5)	Neuroprotective	[960]
Anthozoa/Octocorallia	*Pseudoplexaura flagellosa*	Terpenoids	Cembrenoid 2 , cembrenoid 4,cembrenoid 6	Antifouling, antibacterial	[961]
Anthozoa/Octocorallia	*Rumphella antipathies*	Terpenoids	Rumphellclovane B (**1**)	Anti-inflammatory	[962]
Anthozoa/Octocorallia	*Sarcophyton crassocaule*	Terpenoids	Crassocolide N (1), crassocolide O (2), crassocolide P (3)	Cytotoxic towards cancer cells	[963]
Anthozoa/Octocorallia	*Sinularia crassa*	Terpenoids	Crassarines A–H (1–8)	Anti-inflammatory	[964]

Anthozoa/Octocorallia	*Sinularia rigida*	Terpenoids	Sinulariols A - S (1 - 19)	Cytotoxic towards cancer cells	[965]
Anthozoa/Octocorallia	*Sinularia rigida*	Terpenoids	Sinulariols A - S (1 - 19)	Antimicrobial	[965]
Anthozoa/Octocorallia	*Sinularia sp. - Indonesian soft coral*	Terpenoids	Chloroscabrolides B (4)	Anti-inflammatory	[966]
Anthozoa/Octocorallia	*Sinularia sp. - Indonesian soft coral*	Terpenoids	Prescabrolide (5)	Anti-inflammatory	[966]
Anthozoa/Octocorallia	*Sinularia sp. - Indonesian soft coral*	Terpenoids	Chloroscabrolides A (3)	Anti-inflammatory	[966]
Anthozoa/Octocorallia	*Sinularia triangula*	Terpenoids	Sinutriangulin A (1)	Cytotoxic towards cancer cells	[967]
Anthozoa/Octocorallia	*Subergorgia reticulata*	Terpenoids	(þ)-(7R, 10S)-2,5-dimethoxy calamenene (2)	Antifouling	[968]
Anthozoa/Octocorallia	*Subergorgia reticulata*	Terpenoids	(þ)-(7R, 10S)-2-methoxy-5-acetoxy calamenene (3)	Antifouling	[968]
Anthozoa/Octocorallia	*Acanthoprimnoa cristata*	Terpenoids	Cristaxenicin A (1)	Antiprotozoal	[969]
Anthozoa/Octocorallia	*Lobophytum sarcophytoides*	Terpenoids and Lipid derivatives	Lobophytins A and B<br>4 new prostaglandin B2 derivatives	Anti-Inflammatory	[970]
Hydrozoa/Hydroidolina	*Macrorhynchia philippina*	Alkaloids	Macrophilones B−G	cytotoxicity to cancer cell lines	[971].
Hydrozoa/Hydroidolina	*Thuiaria breitfussi*	Alkaloids	Breitfussin C-D	Cytotoxic against human cancer cells	[972]
Hydrozoa/Hydroidolina	*Macrorhynchia philippina*	Quinones	Macrophilone A	Cytotoxic towards cancer cells	[973]
	n.i. soft coral	Terpenoids	Shagenes A (1) and B (2)	Antiparasite	[974]

Supplementary Table S3 Molluscs bioactive natural products isolated from 2010 to 2019 (Sorted alphabetically according to Class/Subclass and then to Chemical class of compounds)

Class/Subclass	Producer species	Chemical Class of compounds	Compound(s)	Activity/toxicity	References
Bivalvia/ Autobranchia	*Mytilus galloprovincialis*	Carotenoids	Carotenoids	Antioxidant	[975]
Bivalvia/ Autobranchia	*Crassostrea gigas*	Peptides	GnRH-related peptides	Neuro-modulatory	[976]
Bivalvia/ Autobranchia	*Meretrix meretrix*	Peptides	Peptides	Antioxidant	[977]
Bivalvia/ Autobranchia	*Mytilus coruscus*	Peptides	Mytilin B	Antibacterial, antiviral	[978,979]
Bivalvia/ Autobranchia	*Mytilus galloprovincialis*	Peptides	Myticalins	Antibacterial	[980]
Bivalvia/ Autobranchia	*Mytilus edulis*	Polyketides	Azaspiracid 7-10	Cytotoxic against cancer cell lines	[981]
Bivalvia/ Autobranchia	*Paphia malabarica*	Polyketides	Aryl polyketides	Anti-inflammatory	[982]
Bivalvia/ Autobranchia	*Crassostrea gigas*	Polyphenols	3,5-dihydroxy-4-methoxybenzyl alcohol	Antioxidant	[983]
Bivalvia/ Autobranchia	*Bathymodiolus thermophilus*	Sphingolipids	Bathymodiolamides A	Antiproliferative	[984]

Bivalvia/ Autobranchia	*Mytilidae*	Steroids	Brassicasterol; 24-Methylenecholesterol	Anti-inflammatory	[985]
Bivalvia/ Autobranchia	*Villorita cyprinoides*	Steroids	several sterols	Anti-inflammatory	[986]
Bivalvia/ Autobranchia	*Paphia malabarica*	Terpenoids	18 (4 → 14), 19 (4 → 8)-bis-abeo C_{19} norditerpenoid	Antioxidant, anti-inflammatory	[987]
Bivalvia/ Autobranchia	*Villorita cyprinoides*	Terpenoids	Isochromenyl meroterpenoids; furano meroterpenoid	Antioxidant, anti-inflammatory	[988]
Cephalopoda/ Coleoidea	*Amphioctopus neglectus*	Polyketides	Macrocyclic lactones	ACE inhibitor	[989]
Cephalopoda/ Coleoidea	*Sepiella inermis*	Terpenoids	1-(3,4,4a,5,8,8a-Hexahydro-8-methoxy-4-m e t hy l -1H-isochromen-4-yloxy)-11-hydroxyethyl pentanoate; ethyl 9-(4,4a,5,8-tetrahydro-3-oxo-3H-isochromen-5-yl)hexanoate	Antihyperglycemic	[990]
Gastropoda/ Caenogastropoda	*Conus genuanus and C. imperialis*	Alkaloids	Genuanine	Neuro-modulatory	[991]
Gastropoda/ Caenogastropoda	*Conus araneosus*	Peptides	14 conotoxins	Neuro-modulatory	[992]
Gastropoda/ Caenogastropoda	*Conus australis*	Peptides	a5/5 conotoxin AusIA	nAChR receptor inhibitor	[993]
Gastropoda/ Caenogastropoda	*Conus bandanus*	Peptides	a4/7-conotoxin, a- BnIA ; BnIIID	Antispasmodic	[994]
Gastropoda/ Caenogastropoda	*Conus consors*	Peptides	m-conotoxin CnIIIC	Analgesic	[995]

Gastropoda/ Caenogastropoda	*Conus figulinus*	Peptides	M-superfamily Fi3a, Fi3b, and Fi3c; T superfamily Fi5a peptides and contryphans fib, fic, and fid	Neuro-modulatory	[996]
Gastropoda/ Caenogastropoda	*Conus imperialis*	Peptides	im23a; im23b	Neuro-modulatory	[997]
Gastropoda/ Caenogastropoda	*Conus parius*	Peptides	M-superfamily and O-superfamily peptides	Neuro-modulatory	[998]
Gastropoda/ Caenogastropoda	*Conus regius*	Peptides	4/7-conotoxin, RegIIA	Neuro-modulatory	[999]
Gastropoda/ Caenogastropoda	*Conus spurius*	Peptides	Conorfamide-Sr2	Neuro-modulatory	[1000]
Gastropoda/ Caenogastropoda	*Conus textile*	Peptides	TxVC	Neuro-modulatory	[1001]
Gastropoda/ Caenogastropoda	*Conus textile*	Peptides	a-TxIA	Neuro-modulatory	[1002]
Gastropoda/ Caenogastropoda	*Conus virgo*	Peptides	Conotoxin Vi804	Neuro-modulatory	[1003]
Gastropoda/ Caenogastropoda	*Rapana venosa*	Proteins	Emocyanin	Antimicrobial	[1004]
Gastropoda/ Caenogastropoda	*Conus pulicarius*	Steroidal Glycosides	Conusaponin A-C	Cytotoxic against cancer cell lines	[1005]
Gastropoda/ Heterobranchia	*Phidiana militaris*	Alkaloids	Phidianidine A and B	Cytotoxic against cancer cell lines, Immunosuppressor, Antifoulant	[1006–1009]
Gastropoda/ Heterobranchia	*Pleurobranchus forskalii*	Alkaloids	Ergosinine	Neuromodulator	[1010]

Gastropoda/ Heterobranchia	*Tambja ceutae*	Alkaloids	Tambjamine	Antiproliferative (aspecific)	[1011]
Gastropoda/ Heterobranchia	*Aplysia californica*	Aminoacids/ mycosporine-like amino acids	Aplysiapalythine A–C	UV-absorbing	[1012]
Gastropoda/ Heterobranchia	*Elysia ornata*	Depsipeptides	Kahalalide Z1; Kahalalide Z2	Antifungal; antiproliferative	[1013]
Gastropoda/ Heterobranchia	*Actinocyclus papillatus*	Lipid derivatives	Actisonitrile	Cytotoxic against cancer cell lines (modest)	[1014]
Gastropoda/ Heterobranchia	*Stylocheilus longicauda*	Macrolides	Aplysiatoxin	Antiproliferative	[1015]
Gastropoda/ Heterobranchia	*Pleurobranchus forskalii*	Peptides	Cycloforskamide	Cytotoxic against cancer cell lines	[1010]
Gastropoda/ Heterobranchia	*Aldisa andersoni*	Polyketides	Phorboxazole	Antiproliferative	[1016]
Gastropoda/ Heterobranchia	*Aplysia kurodai*	Polyketides	Aplyronine congeners D–H	Cytotoxic against cancer cell lines	[1017,1018]
Gastropoda/ Heterobranchia	*Aplysia kurodai*	Polyketides	Aplyronine A and C	Cytotoxic against cancer cell lines	[1019]
Gastropoda/ Heterobranchia	*Onchidium sp.*	Polyketides	Ilikonapyrones, onchidione	Cytotoxic against cancer cell lines	[1020,1021]
Gastropoda/ Heterobranchia	*Scaphander lignarius*	Polyketides	Lignarenone	Anti-inflammatory/ Immunomodulatory	[1022,1023]
Gastropoda/ Heterobranchia	*Aplysia kurodai*	Steroids	Aplysiasecosterol A	Cytotoxic against cancer cell lines	[1024]

Gastropoda/ Heterobranchia	*Dolabrifera dolabrifera*	Steroids	5α,8α-Epidioxycholest-6-en-3β-ol	Anti-parasitic (anti-Leishmania)	[1025]
Gastropoda/ Heterobranchia	*Actinocyclus papillatus*	Terpenoids	Actinofide	Antiproliferative	[1026]
Gastropoda/ Heterobranchia	*Aplysia dactylomela*	Terpenoids	Dactylditerpenol acetate	anti-neuroinflammatory	[1027]
Gastropoda/ Heterobranchia	*Aplysia dactylomela*	Terpenoids	Aplysqualenol A	Cytotoxic against cancer cell lines	[1028]
Gastropoda/ Heterobranchia	*Aplysia oculifera*	Terpenoids	Oculiferane and Epi-obtusane	Cytotoxic against cancer cell lines	[1029]
Gastropoda/ Heterobranchia	*Austrodoris kerguelenensis*	Terpenoids	Palmadorins	Antiproliferative	[1030]
Gastropoda/ Heterobranchia	*Cadlina luteomarginata*	Terpenoids	Ansellone A	cAMP signaling acivation	[1031]
Gastropoda/ Heterobranchia	*Goniobranchus splendidus*	Terpenoids	Gracilins O, P and Q;	Cytotoxic against cancer cell lines	[1032]
Gastropoda/ Heterobranchia	*Hypselodoris jacksoni*	Terpenoids	Dendrolasin-5-acetate	Antibacterial	[1033]
Gastropoda/ Heterobranchia	*Phyllidia coelestis*	Terpenoids	1-formamido-10(1→2)-abeopupukeanane	Antiproliferative	[1034]
Gastropoda/ Heterobranchia	*Phyllidia ocellata*	Terpenoids	2-Isocyanoclovene; 2-Isocyanoclovane; 1-Isothiocyanatoepicaryolane; 4,5-Epi-10-isocyanoisodauc-6-ene;13-Isocyanocubebane	Antiparasite (anti-Plasmodium falciparum)	[1035]

Gastropoda/ Heterobranchia	*Phyllidiella pustulosa*	Terpenoids	Pustulosaisonitrile-1	Antiparasite (anti-Plasmodium falciparum)	[1036]
Gastropoda/ Patellogastropoda	*Cellana grata and C. toreuma*	Steroids	Unsaturated 4-methyl and 4,4-dimethyl sterols	Anti-inflammatory	[1037]
Gastropoda/ Vetigastropoda	*Monodonta labio*	Polyketides	Monodontins A and B	Cytotoxic against cancer cell lines	[1038]
Gastropoda/ Vetigastropoda	*Scutus antipodes*	Polyols	Scutinin A and B	Antibacterial;, antifungal	[1039]

Supplementary Table S4. Echinoderm bioactive natural products isolated from 2010 to 2019. (Sorted alphabetically according to Class/Subclass and then to Chemical class of compounds)

**Class/ Subclass**	**Producer species**	**Chemical Class of compounds**	**Compound(s)**	**Bioactivity**	**References**
Asteroidea/ Ambuloasteroidea	*Asterias amurensis*	Lipids/ Glycolipid	gangliosides LLG-3	Apoptosis induction, antiproliferative	[1040]
Asteroidea/ Ambuloasteroidea	*Linchia laevigata*	Lipids/Glycolipid	gangliosides LLG-3	neuritogenic activity	[1041]
Asteroidea/ Ambuloasteroidea	*Narcissia canariensis*	Lipids/Glycolipid	peracetylated derivatives	cytotoxic towards cancer cells	[1042]
Asteroidea/ Ambuloasteroidea	*Patiria pectinifera*	Peptides	cysteine-rich antimicrobial peptide (PpCrAMP)	Antimicrobial	[1043]
Asteroidea/ Ambuloasteroidea	*Craspidaster hesperus*	Polyhydroxy steroidal glycosides	novaeguinoside A	cytotoxic towards cancer cells	[1044]
Asteroidea/ Ambuloasteroidea	*Culcita novaeguineae*	Polyhydroxy steroidal glycosides	culcinosides A–D, echinasteroside C, linckoside F and linckoside L3	cytotoxic towards cancer cells	[1045]

Asteroidea/ Ambuloasteroidea	*Anthenea aspera*	Polyhydroxylated steroid	Anthenosides A1 and A2	cytotoxic towards cancer cells, inhibition of cell migration	[1046]
Asteroidea/ Ambuloasteroidea	*Asterina pectinifera*	Polyhydroxylated steroid	(25S)-5alpha-cholestane-3beta,6alpha,7alpha,8,15alpha,16beta-hexahydroxyl-26-O-14'Z-eicosenoate together with seven known steroid derivatives	cytotoxic towards cancer cells, antiviral	[1047]
Asteroidea/ Ambuloasteroidea	*Asteropsis carinifera*	Polyhydroxylated steroid	cariniferosides A–F	inhibition of colony formation of cancer cells.	[1048]
Asteroidea/ Ambuloasteroidea	*Astropecten polyacanthus*	Polyhydroxylated steroid	(20*R*,24*S*)-3β,6α,8,15β,24-pentahydroxy-5α-cholestane	cytotoxic toward cancer cells.	[1049]
Asteroidea/ Ambuloasteroidea	*Ceramaster patagonicus*	Polyhydroxylated steroid	esters of polyhydroxysteroids with long-chain fatty acids	Inhibition of colony formation and migration of cancer cells	[1050]
Asteroidea/ Ambuloasteroidea	*Choriaster granulatus*	Polyhydroxylated steroid	granulatosides D	immunomodulatory activity	[1051]
Asteroidea/ Ambuloasteroidea	*Choriaster granulatus*	Polyhydroxylated steroid	granulatosides D, echinasterosides B and F and laeviuscoloside D	cytotoxic toward cancer cells	[1051]
Asteroidea/ Ambuloasteroidea	*Culcita novaeguineae*	Polyhydroxylated steroid		cytotoxic toward cancer cells	[1052]
Asteroidea/ Ambuloasteroidea	*Echinaster luzonicus*	Polyhydroxylated steroid	luzonicoside	cytotoxic toward cancer cells	[1053]
Asteroidea/ Ambuloasteroidea	*Echinaster luzonicus*	Polyhydroxylated steroid	luzonicosides B–E	lysosomal activity stimulation, intracellular ROS level elevation, and NO synthesis up-regulation in murine macrophages	[1054]
Asteroidea/ Ambuloasteroidea	*Leptasterias hylodes reticulata and*	Polyhydroxylated steroid		antimicrobial	[1055]

	Culcita novaeguineae				
Asteroidea/ Ambuloasteroidea	*Pentaceraster gracilis*	Polyhydroxylated steroid	maculatoside	cytotoxic toward cancer cells	[1056]
Asteroidea/ Ambuloasteroidea	*Pentaceraster regulus*	Polyhydroxylated steroid	regulusoside D, (24*S*)-cholestane-3*β*,5,6*β*,8,15*α*,24-hexol, granulatoside A, 5*α*-cholestane-3*β*,6*β*,7*α*,15*α*,16*β*,26-hexol, 5*α*-cholestane-3*β*,6*β*,7*α*,8*β*,15*α*,16*β*,26-heptol and 5*α*-cholestane-3*β*,6*α*,8,15*α*,16*β*,26-hexol	Immunomodulatory	[1057]
Asteroidea/ Ambuloasteroidea	*Acanthaster planci*	Polyhydroxylated steroid	plancisides A	antiproliferative	[1058]
Asteroidea/ Ambuloasteroidea	*Acanthaster planci*	Polyhydroxylated steroid	plancisides B, C	cytotoxic towards cancer cells	[1058]
Asteroidea/ Ambuloasteroidea	*Acanthaster planci*	Polyhydroxylated steroid	ethanol extract	cytotoxic towards cancer cells, inhibition of colony formation and cell migration	[1059]
Asteroidea/ Ambuloasteroidea	*Craspidaster hesperus*	Polyhydroxysteroidal glycosides	hesperuside A, B, C	cytotoxic toward cancer cells	[1044]
Asteroidea/ Ambuloasteroidea	*Acanthaster planci*	Proteins	plancitoxin I	cytotoxic toward cancer cells, antiproliferative	[1060,1061]
Asteroidea/ Ambuloasteroidea	*Astropecten polyacanthus*	Steroid derivatives	polyacanthoside A	cytotoxic toward cancer cells	[1049]
Asteroidea/ Ambuloasteroidea	*Leptasterias hylodes reticulata and*	Steroidal glycosides-Asterosaponins	hylodoside A and novaeguinoside Y	hemolytic activity	[1055]

	Culcita novaeguineae				
Asteroidea/ Ambuloasteroidea	*Leptasterias ochotensis*	Steroidal glycosides-Asterosaponins	leptasteriosides A-F	cytotoxic toward cancer cells	[1062]
Asteroidea/ Ambuloasteroidea	*Aphelasterias japonica*	Steroidal glycosides-Asterosaponins	Aphelasteroside F	Antiproliferative, inhibition of colony formation of cancer cells	[1063]
Asteroidea/ Ambuloasteroidea	*Archaster typicus*	Steroidal glycosides-Asterosaponins	archasterosides A and B and regularoside A	cytotoxic toward cancer cells	[1064]
Asteroidea/ Ambuloasteroidea	*Asterias microdiscus*	Steroidal glycosides-Asterosaponins	thornasteroside A and versicoside A	cytotoxic toward cancer cells	[1065]
Asteroidea/ Ambuloasteroidea	*Asterina pectinifera*	Steroidal glycosides-Asterosaponins	Asterosaponins P1	cytotoxic toward cancer cells	[1066]
Asteroidea/ Ambuloasteroidea	*Asteropsis carinifera*	Steroidal glycosides-Asterosaponins	asteropsiside A, regularoside A and thornasteroside A	Antiproliferative	[1067]
Asteroidea/ Ambuloasteroidea	*Asteropsis carinifera*	Steroidal glycosides-Asterosaponins	thornasteroside A	cytotoxic toward cancer cells	[1068]
Asteroidea/ Ambuloasteroidea	*Astropecten monacanthus*	Steroidal glycosides-Asterosaponins	astrosterioside D	cytotoxic toward cancer cells	[1069]
Asteroidea/ Ambuloasteroidea	*Astropecten monacanthus*	Steroidal glycosides-Asterosaponins	astrosterioside A and D, marthasteroside B	anti-inflammatory	[1070]
Asteroidea/ Ambuloasteroidea	*Culcita novaeguineae*	Steroidal glycosides-Asterosaponins	novaeguinosides I and II	cytotoxic toward cancer cells	[1071]
Asteroidea/ Ambuloasteroidea	*Culcita novaeguineae*	Steroidal glycosides-Asterosaponins	CN-3	cytotoxic toward cancer cells, antiproliferative	[1072]

Asteroidea/ Ambuloasteroidea	*Diplasterias brucei*	Steroidal glycosides-Asterosaponins	diplasteriosides A and B	cytotoxic toward cancer cells	[1073]
Asteroidea/ Ambuloasteroidea	*Lethasterias fusca*	Steroidal glycosides-Asterosaponins	lethasteriosides A	inhibition of colony formation of cancer cells	[1074]
Asteroidea/ Ambuloasteroidea	*Pentaceraster regulus*	Steroidal glycosides-Asterosaponins	pentaregulosides A	cytotoxic toward cancer cells	[1075]
Asteroidea/ Ambuloasteroidea	*Pentaceraster regulus*	Steroidal glycosides-Asterosaponins	pentaregulosides C-E	immunomodulatory activity	[1075]
Asteroidea/ Ambuloasteroidea	*Acanthaster planci*	Steroidal glycosides-Asterosaponins	acanthaglycoside G	inhibition of cell migration and colony formation of cancer cells	[1059]
Asteroidea/ Ambuloasteroidea	*Acanthaster planci*	Steroidal glycosides-Asterosaponins	acanthaglycoside A and maculatoside (or luidiaglycoside B)	inhibition of cell migration and colony formation of cancer cells	[1059]
Asteroidea/ Ambuloasteroidea	*Ctenodiscus crispatus*	Steroids	(25S)-5α5α-cholestane-3β3β,5,6β6β,15α15α,16β16β,26-hexaol	cytotoxic toward cancer cells	[1076]
Asteroidea/ Ambuloasteroidea	*Leptasterias ochotensis*	Steroids	asterogenin	cytotoxic toward cancer cells	[1062]
Asteroidea/ Ambuloasteroidea	*Distolasterias nipon*	Steroids	polar steroids	neuritogenic and neuroprotective	[1077]
Asteroidea/ Ambuloasteroidea	*Patiria pectinifera*	Steroids	polar steroids	neuritogenic and neuroprotective activities	[1077]
Asteroidea/ Ambuloasteroidea	*Astropecten polyacanthus*	Sterols	5α-cholest-7-ene-3β,6α-diol (5),3) 5α-cholest8(14)-ene-3β,7α-diol (6),4) and 5α-cholest-7,9(11)-diene-3β-ol	Apoptosis induction in leukemia related cells	[1078]
Asteroidea/ Ambuloasteroidea	*Leptasterias ochotensis*	Sulfated steroid monoglycoside	leptaochotensoside A	inhibition of colony formation of cancer cells	[1079]

Asteroidea/ Ambuloas-teroidea	*Leptasterias ochotensis*	Sulfated steroid monoglycosides	leptaochotensoside A	inhibition of colony formation of cancer cells	[1080]
Asteroidea/ Ambuloas-teroidea	*Solaster pacificus*	Triterpene glycosides	cucumariosides C1, C2, and A10	cytotoxic activity	[1081]
Asteroidea/ Ambuloas-teroidea	*Solaster pacificus*	Triterpene glycosides	pacificusosides C, cucumariosides C2 and A10	inhibition of colony formation of cancer cells	[1081]
Crinoidea/ Articulata	*Comanthus Sp*	Anthraquinone derivative	1'-deoxyrhodoptilometrin (SE11) and (S)-(-)-rhodoptilometrin (SE16)	cytotoxic toward cancer cells, apoptosis induction	[1082]
Crinoidea/ Articulata	*Anneissia japonica*	Lectin	OXYL	Antibiofilm	[1083]
Echinoidea/Euechinoidea (sea urchins)	*Arbacia Lixula*	Carotenoid	Astaxanthin	Antioxidant, anti-neurodegenerative	[1084]
Echinoidea/Euechinoidea (sea urchins)	*Toxopneustes pileolus*	Lectins	rhamnose-binding lectin (cloned)	mitogenic, chemotactic, and cytotoxic activities	[1085]
Echinoidea/Euechinoidea (sea urchins)	*Strongylocentrotus purpuratus*	Lectins	rhamnose-binding lectin	Apoptosis induction in cancer cells	[1086]
Echinoidea/Euechinoidea (sea urchins)	*Strongylocentrotus droebachiensis*	Peptide	Centrocin	antimicrobial	[1087]
Echinoidea/Euechinoidea (sea urchins)	*Paracentrotus lividus*	Peptide	antimicrobial peptides	antimicrobial	[1088]

Echinoidea/Euechinoidea (sea urchins)	*Paracentrotus lividus*	Peptide	Beta-thymosin fragment	antimicrobial and antibiofilm activity	[1089]
Echinoidea/Euechinoidea (sea urchins)	*Paracentrotus lividus*	Peptide	Paracentrin 1	antimicrobial	[1090]
Echinoidea/Euechinoidea (sea cucumbers)	*Echinus euculentus*	Peptide	Centrocin	antimicrobial	[1091]
Echinoidea/Euechinoidea (sea cucumbers)	*Echinus euculentus*	Peptide	Strongylocin	antimicrobial	[1091]
Echinoidea/Euechinoidea (sea urchins)	*Paracentrotus lividus*	Peptide	Derivatives from paracentrin	antimicrobial, antibiofilm	[1092]
Echinoidea/Euechinoidea (sea urchins)	*Diadema savignyi*	Polyhydroxylated naphthoquinones	Spinochrome	antibacterial, antioxidant, inflammatory and cytotoxic	[1093]
Echinoidea/Euechinoidea (sea urchins)	*Echinometra mathaei*	Polyhydroxylated naphthoquinones	Spinochrome	antibacterial, antioxidant, inflammatory and cytotoxic	[1093]
Echinoidea/Euechinoidea (sea urchins)	*Toxopneustes pileolus*	Polyhydroxylated naphthoquinones	Spinochrome	antibacterial, antioxidant, inflammatory and cytotoxic	[1093]
Echinoidea/Euechinoidea	*Tripneustes gratilla*	Polyhydroxylated naphthoquinones	Spinochrome	antibacterial, antioxidant, inflammatory and cytotoxic	[1093]

(sea urchins)					
Echinoidea/Euechinoidea (sea urchins)	*Strongylocentrotus nudus*	Polysaccharide	SEP	cytotoxic toward cancer cells	[1094]
Echinoidea/Euechinoidea (sea urchins)	*Strongylocentrotus nudus*	Polysaccharide	SEP	cytotoxic toward cancer cells	[1095]
Echinoidea/Euechinoidea (sea urchins)	*Strongylocentrotus nudus*	Polysaccharide	SEP (combined with gemcitabine)	cytotoxic toward cancer cells	[1096]
Echinoidea/Euechinoidea	*Echinometra lucunter*	Polysaccharides	sulfated fucans and galactans	anticoagulant and antithrombotic	[1097]
Echinoidea/Euechinoidea	*Echinometra lucunter*	Polysaccharides	fucan sulfate	anticoagulant and antithrombotic	[1098]
Echinoidea/Euechinoidea (sea urchins)	*Lytechinus variegatus*	Polysaccharides	fucan sulfate	anticoagulant and antithrombotic	[1098]
Echinoidea/Euechinoidea (sea urchins)	*Paracentrotus lividus*	Thiol	ovothiol	Antioxidant	[1099]
Echinoidea/sea urchins	n.a.	Polyhydroxylated naphthoquinones	Echinochrome A	Antioxidant	[1100]
Echinoidea/sea urchins	n.a.	Polyhydroxylated naphthoquinones	Echinochrome A	antioxidant, antimicrobial, anti-inflammatory, and chelating effects	[1101]

Holoturoidea/ Actinopoda (sea cucumbers)	*Holothuria edulis*	Polysaccharides	fucan sulfate	anticoagulant	[1102]
Holoturoidea/ Actinopoda (sea cucumbers)	*Apostichopus japonicas*	Alkenes	(5Z)-dec-5-en-1-yl sulfate, (3E)-dec-3-en-1-yl sulfate, 2,6-dimethylheptyl sulfate, octyl sulfate and decyl sulfate	cytotoxic toward cancer cells, antibacterial	[1103]
Holoturoidea/ Actinopoda (sea cucumbers)	*Holothuria polii*	Glycoside	Bivittoside	cytotoxic toward cancer cells	[1104]
Holoturoidea/ Actinopoda (sea cucumbers)	*Apostichopus japonicas*	Lectin	fusion alkakine phosphatase-lectin MBL-Aj		[1105]
Holoturoidea/ Actinopoda (sea cucumbers)	*Apostichopus japonicas*	Lectin	AJCTL		[1106]
Holoturoidea/ Actinopoda (sea cucumbers)	*Holothuria grisea*	Lectin	HGL agglutinin	anti-inflammatory	[1107]
Holoturoidea/ Actinopoda (sea cucumbers)	*Holothuria grisea*	Lectin	HGL	hemagglutinating/hemolytic activity	[1108]
Holoturoidea/ Actinopoda (sea cucumbers)	*Cucumaria echinata*	Lectin	CEL-III	hemolytic activity	[1109]
Holoturoidea/ Actinopoda	*Apostichopus japonicas*	Lectin	Fucolectin AjFL-1	binding activities towards bacteria, agglutination *P. pastoris*	[1110]

(sea cucumbers)					
Holoturoidea/ Actinopoda (sea cucumbers)	*Holothuria tubulosa*	Low MW peptides	Holothuroidin	antimicrobial	[1111]
Holoturoidea/ Actinopoda (sea cucumbers)	*Holothuria albiventer*	Polysaccharides	fucan sulfate	anticoagulant	[1112]
Holoturoidea/ Actinopoda (sea cucumbers)	*Stichopus japonicus*	Polysaccharides	immunostimulating protein-sulfated fucan complex	anticoagulant	[1113]
Holoturoidea/ Actinopoda (sea cucumbers)	*Isostichopus badionotus*	Polysaccharides	fucan sulfate	Anticoagulant, antithrombotic	[1114]
Holoturoidea/ Actinopoda (sea cucumbers)	*Stichopus japonicus*	Polysaccharides	sulfated fucans	osteoclastogenesis	[1115]
Holoturoidea/ Actinopoda (sea cucumbers)	*Isostichopus badionotus*	Polysaccharides	fucosylated chondroitin sulfate and fucoidans	hypolipidemic	[1116]
Holoturoidea/ Actinopoda (sea cucumbers)	*Isostichopus badionotus*	Polysaccharides	fucan sulfate	hypolipidemic	[1116]
Holoturoidea/ Actinopoda (sea cucumbers)	*Pearsonothuria graeffei*	Polysaccharides	fucosylated chondroitin sulfate and fucoidans	hypolipidemic	(Li et al., 2017)

Holoturoidea/ Actinopoda (sea cucumbers)	*Pearsonothuria graeffei*	Polysaccharides	fucan sulfate	hypolipidemic	[1116]
Holoturoidea/ Actinopoda (sea cucumbers)	*Holothuria Mexicana*	Polysaccharides	fucosylated chondroitin sulfate	anticoagulant	[1117]
Holoturoidea/ Actinopoda (sea cucumbers)	*Cucumaria frondosa*	Polysaccharides	fucosylated chondroitin sulfate	anticoagulant	[1118]
Holoturoidea/ Actinopoda (sea cucumbers)	*Apostichopus japonicas*	Polysaccharides	fucan sulfate	anticoagulant	[1102]
Holoturoidea/ Actinopoda (sea cucumbers)	*Holothuria nobilis*	Polysaccharides	fucan sulfate	anticoagulant	[1102]
Holoturoidea/ Actinopoda (sea cucumbers)	*Holothuria forskali*	Polysaccharides	fucosylated chondroitin sulfate	anti-inflammation	[1119]
Holoturoidea/ Actinopoda (sea cucumbers)	*Holothuria fuscopunctata*	Polysaccharides	fucan sulfate	anticoagulant	[1120]
Holoturoidea/ Actinopoda (sea cucumbers)	*Stichopus horrens*	Polysaccharides	fucan sulfate	anticoagulant	[1120]

Holoturoidea/ Actinopoda (sea cucumbers)	*Thelenota ananas*	Polysaccharides	fucan sulfate	anticoagulant activities	[1120]
Holoturoidea/ Actinopoda (sea cucumbers)	*Stichopus japonicus*	Polysaccharides	sulfated fucans	cytotoxicity	[1121]
Holoturoidea/ Actinopoda (sea cucumbers)	*Stichopus variegatus*	Polysaccharides	fucosylated chondroitin sulfates	antitumor	[1122]
Holoturoidea/ Actinopoda (sea cucumbers)	*Stichopus horrens*	Polysaccharides	fucosylated chondroitin sulfate	anticoagulant	[1123]
Holoturoidea/ Actinopoda (sea cucumbers)	*Cucumaria djakonovi*	Polysaccharides	fucosylated chondroitin sulfate	Anti-inflammatory	[1124]
Holoturoidea/ Actinopoda (sea cucumbers)	*Apostichopus japonicas*	Polysaccharides	glycosyl aminoglycan	immunomodulatory	[1125]
Holoturoidea/ Actinopoda (sea cucumbers)	*Holothuria edulis*	Polysaccharides	fucosylated chondroitin sulfate	anticoagulant	[1126]
Holoturoidea/ Actinopoda (sea cucumbers)	*Ludwigothurea grisea*	Polysaccharides	fucosylated chondroitin sulfate	anticoagulant	[1126]
Holoturoidea/ Actinopoda	*Holothuria coluber*	Polysaccharides	fucan sulfate	anticoagulant	[1127]

(sea cucumbers)					
Holoturoidea/ Actinopoda (sea cucumbers)	*Holothuria scabra*	Polysaccharides	fucosylated chondroitin sulfate	anticoagulant	[1128]
Holoturoidea/ Actinopoda (sea cucumbers)	*Cucumaria japonica*	Saponins	Cumaside	cytotoxic toward cancer cells	[1129]
Holoturoidea/ Actinopoda (sea cucumbers)	*Holothuria lesson*	Saponins	triterpene glycoside/saponin	cytotoxic toward cancer cells, wound healing, hypolipidemia, pain relieving, anti-hypertension, anti-ageing.	[1130]
Holoturoidea/ Actinopoda (sea cucumbers)	*Stichopus horrens*	Saponins	stichorrenosides A-D	cytotoxic toward cancer cells	[1131]
Holoturoidea/ Actinopoda (sea cucumbers)	*Cucumaria japonica*	Saponins	cucumarioside A2-2	cytostatic	[1132]
Holoturoidea/ Actinopoda (sea cucumbers)	*Cucumaria frondosa*	Saponins	Frondoside A	cytotoxic toward cancer cells	[1133]
Holoturoidea/ Actinopoda (sea cucumbers)	*Stichopus horrens*	Saponins	stichorrenoside E	cytotoxic toward cancer cells	[1134]
Holoturoidea/ Actinopoda	*Holothuria moebii*	Saponins	sulfated and non-sulfated saponins	cytotoxic toward cancer cells	[1135]

(sea cucumbers)					
Holoturoidea/ Actinopoda (sea cucumbers)	*Stichopodiidae*	Saponins	stichoposides, triterpene glycosides	ceramide inducers	[1136]
Holoturoidea/sea cucumbers	n.a.	Glycan	glycosaminoglycan (hGAG),	inhibitor of P-selectin	[1137]

Supplementary Table S5

. Tunicates bioactive natural products isolated from 2010 to 2019 (Sorted alphabetically according to Class/Subclass and then to Chemical class of compounds).

Class/Order	Producer species	Chemical Class of compounds	Compound(s)	Activity/toxicity	References
Ascidiacea/Aplousobranchia	*Didemnidae*	Amino alchool	Siladenoserinols	Cytotoxic against cancer cells (moderate)	[1138]
Ascidiacea/Aplousobranchia	*Didemnum sp.*	Alkaloids	3-bromofascaplysin	Cytotoxic against cancer cells (moderate)	[1139,1140]
Ascidiacea/Aplousobranchia	*Didemnum ternerratum*	Alkaloids	Lamellarin Sulfates	Cytotoxic against cancer cells	[1141]
Ascidiacea/Aplousobranchia	*Eudistoma glaucus*	Alkaloids	Eudistomidin B, G	Cytotoxic against cancer cells	[1139,1142]
Ascidiacea/Aplousobranchia	*Pseudodistoma opacum*	Alkaloids	Opacaline B-C	Anti-parasitic (anti-P*lasmodium falciparum*)	[1139,1143]
Ascidiacea/Aplousobranchia	*Diazona formosa*	Alkaloids	Tanjungide A-B	Cytotoxic against cancer cells	[1144]
Ascidiacea/Aplousobranchia	*Didemnum* sp.	Alkaloids	Didemninide A-B	Inhibitor of PLA2	[1145]
Ascidiacea/Aplousobranchia	*Aplidium falklandicum, Aplidium meridianum*	Alkaloids	Meridianins	Anti-proliferative; Apoptosis inducer	[1146]
Ascidiacea/Aplousobranchia	*Aplidium* sp., *Synoicum* sp.	Alkaloids	Meridianins	Anti-proliferative; Apoptosis inducer	[1147,1148]
Ascidiacea/Aplousobranchia	*Synoicum adareanum*	Polyketides	Palmerolide A	Cytotoxic against cancer cells	[1139,1149]
Ascidiacea/Aplousobranchia	*Lissoclinum* sp.	Polyketides	Mandelalides	Cytotoxic against cancer cells; Antifungal	[1139,1150]
Ascidiacea/Aplousobranchia	*Polysyncraton sp.*	Polyketides	Mycalamide A	Cytotoxic against cancer cells	[1139,1151]
Ascidiacea/Aplousobranchia	*Aplidium elegans*	Polyketides	Phosphoeleganin	Enzyme inhibitor (Inhibition of tyrosine phosphatase 1B)	[1139,1152]

Ascidiacea/Aplousobranchia	*Synoicum* sp.	Polyketides	*Z*-rubrolide O, *E*-rubrolide O	Immunomodulatory	[1139,1153]
Ascidiacea/Aplousobranchia	*Aplidium* sp., *Synoicum* sp.	Quinones	Rossinone, Epoxy-rossinone	Cytotoxic against cancer cells	[1147]
Ascidiacea/Phlebobranchia	*Ciona intestinalis*	Peptides	Antimicrobial peptide	Antimicrobial	[1154–1157]
Ascidiacea/Phlebobranchia	*Ciona savignyi*	Peptides	CS5931	Cytotoxic against cancer cells	[1139,1158]
Ascidiacea/Phlebobranchia	*Ecteinascidia turbinata*	Polyketides	Arenimycin	Antimicrobia l	[1139,1159]
Ascidiacea/Phlebobranchia	*Ciona savignyi*	Proteins-glycoprotein	Chondromodulin-1	Anti-proliferative; Antioxidant	[1139,1160]
Ascidiacea/Stolidobranchia	*Herdmania momus*	Alkaloids	Herdmanines A-D	Anti-inflammatory	[1139,1161]

Supplementary Table S6: Number of MNPs entries for tunicates, molluscs, sponges and echinoderms in metabolite databases searchable by biological source.

Database	Tunicates	Molluscs	Sponges	Echinoderms	URL

**ChEBI**	10	2	242	0	https://www.ebi.ac.uk/chebi/
**Dictionary of Marine Natural Products***	212	179	2491	9	http://dmnp.chemnetbase.com/faces/chemical/ChemicalSearch.xhtml
**HMDB**	1	1	0	0	https://hmdb.ca/
**KNApSAcK**	3	1	2	0	http://www.knapsackfamily.com/KNApSAcK/
**MetaboLights**	0	0	130	0	https://www.ebi.ac.uk/metabolights/index
**Norine**	0	30	27	0	https://bioinfo.lifl.fr/
**NPCARE**	0	0	3	5	http://silver.sejong.ac.kr/npcancer/

*Specific information for each compound is not publicly available

References

1. Nagasawa, Y.; Kato, H.; Rotinsulu, H.; Mangindaan, R.E.P.; de Voogd, N.J.; Tsukamoto, S. Spironaamidine, a New Spiroquinone-Containing Alkaloid from the Marine Sponge *Leucetta microraphis*. *Tetrahedron Letters* **2011**, *52*, 5342–5344, doi:10.1016/j.tetlet.2011.08.026.
2. Hermawan, I.; De Voogd, N.J.; Tanaka, J. An Acetylenic Alkaloid from the Calcareous Sponge *Leucetta* sp. *Marine Drugs* **2011**, *9*, 382–386, doi:10.3390/md9030382.
3. Zou, Y.; Wang, X.; Sims, J.; Wang, B.; Pandey, P.; Welsh, C.L.; Stone, R.P.; Avery, M.A.; Doerksen, R.J.; Ferreira, D.; et al. Computationally Assisted Discovery and Assignment of a Highly Strained and PANC-1 Selective Alkaloid from Alaska's Deep Ocean. *J Am Chem Soc* **2019**, *141*, 4338–4344, doi:10.1021/jacs.8b11403.
4. Li, F.; Janussen, D.; Peifer, C.; Pérez-Victoria, I.; Tasdemir, D. Targeted Isolation of Tsitsikammamines from the Antarctic Deep-Sea Sponge *Latrunculia biformis* by Molecular Networking and Anticancer Activity. *Mar Drugs* **2018**, *16*, E268, doi:10.3390/md16080268.
5. Li, F.; Peifer, C.; Janussen, D.; Tasdemir, D. New Discorhabdin Alkaloids from the Antarctic Deep-Sea Sponge *Latrunculia biformis*. *Mar Drugs* **2019**, *17*, E439, doi:10.3390/md17080439.
6. An, B.; Yin, F.; de Voogd, N.J.; Chen, X.; Cheng, W.; Lin, W. Chagosendines A – C, New Metal Complexes of Imidazole Alkaloids from the Calcareous Sponge *Leucetta chagosensis*. *Chemistry & Biodiversity* **2018**, *15*, e1700481, doi:10.1002/cbdv.201700481.

7. Tang, W.-Z.; Yang, Z.-Z.; Wu, W.; Tang, J.; Sun, F.; Wang, S.-P.; Cheng, C.-W.; Yang, F.; Lin, H.-W. Imidazole Alkaloids and Their Zinc Complexes from the Calcareous Marine Sponge *Leucetta chagosensis*. *J Nat Prod* **2018**, *81*, 894–900, doi:10.1021/acs.jnatprod.7b01006.
8. Gong, K.-K.; Tang, X.-L.; Liu, Y.-S.; Li, P.-L.; Li, G.-Q. Imidazole Alkaloids from the South China Sea Sponge *Pericharax heteroraphis* and Their Cytotoxic and Antiviral Activities. *Molecules* **2016**, *21*, 150, doi:10.3390/molecules21020150.
9. Kulkarni, R.; Jo, A.; Kim, Y.H.; Na, M. Epi -Leptosphaerin: A New L-Isoascorbic Acid Derivative from Marine Sponges. *Natural Product Sciences* **2015**, *21*, 293, doi:10.20307/nps.2015.21.4.293.
10. Wang, J.; Liu, L.-Y.; Liu, L.; Zhan, K.-X.; Jiao, W.-H.; Lin, H.-W. Pellynols M−O, Cytotoxic Polyacetylenic Alcohols from a *Niphates* sp. Marine Sponge. *Tetrahedron* **2018**, *74*, 3701–3706, doi:10.1016/j.tet.2018.05.041.
11. Barnes, E.C.; Said, N.A.B.M.; Williams, E.D.; Hooper, J.N.A.; Davis, R.A. Ecionines A and B, Two New Cytotoxic Pyridoacridine Alkaloids from the Australian Marine Sponge, *Ecionemia geodides*. *Tetrahedron* **2010**, *66*, 283–287, doi:10.1016/j.tet.2009.10.109.
12. Mudianta, I.W.; Katavic, P.L.; Lambert, L.K.; Hayes, P.Y.; Banwell, M.G.; Munro, M.H.G.; Bernhardt, P.V.; Garson, M.J. Structure and Absolute Configuration of 3-Alkylpiperidine Alkaloids from an Indonesian Sponge of the Genus *Halichondria*. *Tetrahedron* **2010**, *66*, 2752–2760, doi:10.1016/j.tet.2010.01.068.
13. Hwang, B.S.; Oh, J.S.; Jeong, E.J.; Sim, C.J.; Rho, J.-R. Densanins A and B, New Macrocyclic Pyrrole Alkaloids Isolated from the Marine Sponge *Haliclona densaspicula*. *Org. Lett.* **2012**, *14*, 6154–6157, doi:10.1021/ol3028303.
14. Lee, Y.; Jang, K.H.; Jeon, J.; Yang, W.-Y.; Sim, C.J.; Oh, K.-B.; Shin, J. Cyclic Bis-1,3-Dialkylpyridiniums from the Sponge *Haliclona* sp. *Marine Drugs* **2012**, *10*, 2126–2137, doi:10.3390/md10092126.
15. Sorek, H.; Rudi, A.; Aknin, M.; Gaydou, E.M.; Kashman, Y. Isohalitulin and Haliclorensins B and C, Three Marine Alkaloids from *Haliclona tulearensis*. *J. Nat. Prod.* **2010**, *73*, 456–458, doi:10.1021/np900500c.
16. Fan, G.; Li, Z.; Shen, S.; Zeng, Y.; Yang, Y.; Xu, M.; Bruhn, T.; Bruhn, H.; Morschhäuser, J.; Bringmann, G.; et al. Baculiferins A–O, O-Sulfated Pyrrole Alkaloids with Anti-HIV-1 Activity, from the Chinese Marine Sponge *Iotrochota baculifera*. *Bioorganic & Medicinal Chemistry* **2010**, *18*, 5466–5474, doi:10.1016/j.bmc.2010.06.052.
17. Watts, K.R.; Morinaka, B.I.; Amagata, T.; Robinson, S.J.; Tenney, K.; Bray, W.M.; Gassner, N.C.; Lokey, R.S.; Media, J.; Valeriote, F.A.; et al. The Biostructural Features of Additional Jasplakinolide (Jaspamide) Analogues. *J Nat Prod* **2011**, *74*, 341–351, doi:10.1021/np100721g.
18. Na, M.; Ding, Y.; Wang, B.; Tekwani, B.L.; Schinazi, R.F.; Franzblau, S.; Kelly, M.; Stone, R.; Li, X.-C.; Ferreira, D.; et al. Anti-Infective Discorhabdins from a Deep-Water Alaskan Sponge of the Genus *Latrunculia*. *J. Nat. Prod.* **2010**, *73*, 383–387, doi:10.1021/np900281r.

19. Sirirak, T.; Brecker, L.; Plubrukarn, A. Kabiramide L, a New Antiplasmodial Trisoxazole Macrolide from the Sponge *Pachastrissa nux*. *Natural Product Research* **2013**, *27*, 1213–1219, doi:10.1080/14786419.2012.724410.
20. Lyakhova, E.G.; Kolesnikova, S.A.; Kalinovsky, A.I.; Afiyatullov, S.Sh.; Dyshlovoy, S.A.; Krasokhin, V.B.; Minh, C.V.; Stonik, V.A. Bromine-Containing Alkaloids from the Marine Sponge *Penares* sp. *Tetrahedron Letters* **2012**, *53*, 6119–6122, doi:10.1016/j.tetlet.2012.08.148.
21. Guzii, A.G.; Makarieva, T.N.; Denisenko, V.A.; Gerasimenko, A.V.; Udovenko, A.A.; Popov, R.S.; Dmitrenok, P.S.; Golotin, V.A.; Fedorov, S.N.; Grebnev, B.B.; et al. Guitarrins A-E and Aluminumguitarrin A: 5-Azaindoles from the Northwestern Pacific Marine Sponge *Guitarra fimbriata*. *J Nat Prod* **2019**, *82*, 1704–1709, doi:10.1021/acs.jnatprod.9b00334.
22. Abdjul, D.B.; Yamazaki, H.; Kanno, S.; Takahashi, O.; Kirikoshi, R.; Ukai, K.; Namikoshi, M. Haliclonadiamine Derivatives and 6-Epi-Monanchorin from the Marine Sponge *Halichondria panicea* Collected at Iriomote Island. *J. Nat. Prod.* **2016**, *79*, 1149–1154, doi:10.1021/acs.jnatprod.6b00095.
23. Al-Massarani, S.M.; El-Gamal, A.A.; Al-Said, M.S.; Abdel-Kader, M.S.; Ashour, A.E.; Kumar, A.; Abdel-Mageed, W.M.; Al-Rehaily, A.J.; Ghabbour, H.A.; Fun, H.-K. Studies on the Red Sea Sponge *Haliclona* sp. for Its Chemical and Cytotoxic Properties. *Pharmacognosy Magazine* **2016**, *12*, 114, doi:10.4103/0973-1296.177906.
24. Maarisit, W.; Abdjul, D.B.; Yamazaki, H.; Kato, H.; Rotinsulu, H.; Wewengkang, D.S.; Sumilat, D.A.; Kapojos, M.M.; Ukai, K.; Namikoshi, M. Anti-Mycobacterial Alkaloids, Cyclic 3-Alkyl Pyridinium Dimers, from the Indonesian Marine Sponge *Haliclona* sp. *Bioorganic & Medicinal Chemistry Letters* **2017**, *27*, 3503–3506, doi:10.1016/j.bmcl.2017.05.067.
25. Abdjul, D.B.; Yagi, A.; Yamazaki, H.; Kirikoshi, R.; Takahashi, O.; Namikoshi, M.; Uchida, R. Anti-Mycobacterial Haliclonadiamine Alkaloids from the Okinawan Marine Sponge *Haliclona* sp. Collected at Iriomote Island. *Phytochemistry Letters* **2018**, *26*, 130–133, doi:10.1016/j.phytol.2018.05.028.
26. Urda, C.; Pérez, M.; Rodríguez, J.; Fernández, R.; Jiménez, C.; Cuevas, C. Njaoamine I, a Cytotoxic Polycyclic Alkaloid from the Haplosclerida Sponge *Haliclona* (*reniera*) sp. *Tetrahedron Letters* **2018**, *59*, 2577–2580, doi:10.1016/j.tetlet.2018.05.059.
27. Lorig-Roach, N.; Hamkins-Indik, F.; Johnson, T.A.; Tenney, K.; Valeriote, F.A.; Crews, P. The Potential of Achiral Sponge-Derived and Synthetic Bromoindoles as Selective Cytotoxins against PANC-1 Tumor Cells. *Tetrahedron* **2018**, *74*, 217–223, doi:10.1016/j.tet.2017.11.029.
28. Shaaban, M.; Hassan, A.Z.; Soltan, M.M.; Abdelwahab, A.B. Naturally Bioactive Compounds from *Hemimycale* aff *arabica*: Antimicrobial, Antiglycation, Cytotoxicity, and Molecular Docking Studies. *Med Chem Res* **2018**, *27*, 2079–2088, doi:10.1007/s00044-018-2217-6.
29. Youssef, D.T.A.; Shaala, L.A.; Alshali, K.Z. Bioactive Hydantoin Alkaloids from the Red Sea Marine Sponge *Hemimycale arabica*. *Marine Drugs* **2015**, *13*, 6609–6619, doi:10.3390/md13116609.

30. McCauley, E.P.; Lam, H.; Lorig-Roach, N.; Luu, J.; Lloyd, C.; Tenney, K.; Pietraszkiewicz, H.; Diaz, C.; Valeriote, F.A.; Auerbuch, V.; et al. Investigation of the Physical and Bioactive Properties of Bromo- and Iodo-Containing Sponge-Derived Compounds Possessing an Oxyphenylethanamine Core. *J. Nat. Prod.* **2017**, *80*, 3255–3266, doi:10.1021/acs.jnatprod.7b00694.
31. Ebada, S.S.; Müller, W.E.G.; Lin, W.; Proksch, P. New Acyclic Cytotoxic Jasplakinolide Derivative from the Marine Sponge *Jaspis splendens*. *Marine Drugs* **2019**, *17*, 100, doi:10.3390/md17020100.
32. Hitora, Y.; Takada, K.; Ise, Y.; Okada, S.; Matsunaga, S. Dragmacidins G and H, Bisindole Alkaloids Tethered by a Guanidino Ethylthiopyrazine Moiety, from a *Lipastrotethya* sp. Marine Sponge. *J. Nat. Prod.* **2016**, *79*, 2973–2976, doi:10.1021/acs.jnatprod.6b00710.
33. Lyakhova, E.G.; Kolesnikova, S.A.; Kalinovsky, A.I.; Berdyshev, D.V.; Pislyagin, E.A.; Kuzmich, A.S.; Popov, R.S.; Dmitrenok, P.S.; Makarieva, T.N.; Stonik, V.A. Lissodendoric Acids A and B, Manzamine-Related Alkaloids from the Far Eastern Sponge *Lissodendoryx florida*. *Org. Lett.* **2017**, *19*, 5320–5323, doi:10.1021/acs.orglett.7b02608.
34. Shubina, L.K.; Makarieva, T.N.; Yashunsky, D.V.; Nifantiev, N.E.; Denisenko, V.A.; Dmitrenok, P.S.; Dyshlovoy, S.A.; Fedorov, S.N.; Krasokhin, V.B.; Jeong, S.H.; et al. Pyridine Nucleosides Neopetrosides A and B from a Marine *Neopetrosia* sp. Sponge. Synthesis of Neopetroside A and Its β-Riboside Analogue. *J. Nat. Prod.* **2015**, *78*, 1383–1389, doi:10.1021/acs.jnatprod.5b00256.
35. Nakamukai, S.; Ise, Y.; Ohtsuka, S.; Okada, S.; Matsunaga, S. Isolation and Identification of N6-Isopentenyladenosine as the Cytotoxic Constituent of a Marine Sponge *Oceanapia* sp. *Biosci Biotechnol Biochem* **2019**, *83*, 1985–1988, doi:10.1080/09168451.2019.1630258.
36. Mohamed, G.A.; Abd-Elrazek, A.E.E.; Hassanean, H.A.; Youssef, D.T.A.; Soest, R. van New Compounds from the Red Sea Marine Sponge *Echinoclathria gibbosa*. *Phytochemistry Letters* **2014**, *Complete*, 51–58, doi:10.1016/j.phytol.2014.04.008.
37. Nuzzo, G.; Ciavatta, M.L.; Villani, G.; Manzo, E.; Zanfardino, A.; Varcamonti, M.; Gavagnin, M. Fulvynes, Antimicrobial Polyoxygenated Acetylenes from the Mediterranean Sponge *Haliclona fulva*. *Tetrahedron* **2012**, *68*, 754–760, doi:10.1016/j.tet.2011.10.068.
38. Alarif, W.M.; Abdel-Lateff, A.; Al-Lihaibi, S.S.; Ayyad, S.-E.N.; Badria, F.A. A New Cytotoxic Brominated Acetylenic Hydrocarbon from the Marine Sponge *Haliclona* sp. with a Selective Effect against Human Breast Cancer. *Z. Naturforsch., C: J. Biosci.* **2013**, *68C*, 70–75.
39. Sato, S.; Iwata, F.; Takeo, J.; Kawahara, H.; Kuramoto, M.; Uno, H. Stellettazole D, a Cytotoxic Imidazole Alkaloid from the Marine Sponge *Jaspis duoaster*. *Chem. Lett.* **2011**, *40*, 186–187, doi:10.1246/cl.2011.186.
40. Lee, J.-H.; Jang, K.H.; Lee, Y.-J.; Lee, H.-S.; Sim, C.J.; Oh, K.-B.; Shin, J. Triterpene Galactosides of the Pouoside Class and Corresponding Aglycones from the Sponge *Lipastrotethya* sp. *J Nat Prod* **2011**, *74*, 2563–2570, doi:10.1021/np200748g.

41. Lee, J.-H.; Jeon, J.; Lee, Y.-J.; Lee, H.-S.; Sim, C.J.; Oh, K.-B.; Shin, J. Nortriterpene Glycosides of the Sarasinoside Class from the Sponge *Lipastrotethya* sp. *J. Nat. Prod.* **2012**, *75*, 1365–1372, doi:10.1021/np300297d.
42. Meimetis, L.G.; Williams, D.E.; Mawji, N.R.; Banuelos, C.A.; Lal, A.A.; Park, J.J.; Tien, A.H.; Fernandez, J.G.; de Voogd, N.J.; Sadar, M.D.; et al. Niphatenones, Glycerol Ethers from the Sponge *Niphates digitalis* Block Androgen Receptor Transcriptional Activity in Prostate Cancer Cells: Structure Elucidation, Synthesis, and Biological Activity. *J. Med. Chem.* **2012**, *55*, 503–514, doi:10.1021/jm2014056.
43. Legrave, N.; Hamrouni-Buonomo, S.; Dufies, M.; Guérineau, V.; Vacelet, J.; Auberger, P.; Amade, P.; Mehiri, M. Nepheliosyne B, a New Polyacetylenic Acid from the New Caledonian Marine Sponge *Niphates* sp. *Marine Drugs* **2013**, *11*, 2282–2292, doi:10.3390/md11072282.
44. Regalado, E.L.; Tasdemir, D.; Kaiser, M.; Cachet, N.; Amade, P.; Thomas, O.P. Antiprotozoal Steroidal Saponins from the Marine Sponge *Pandaros acanthifolium*. *J. Nat. Prod.* **2010**, *73*, 1404–1410, doi:10.1021/np100348x.
45. Regalado, E.L.; Turk, T.; Tasdemir, D.; Gorjanc, M.; Kaiser, M.; Thomas, O.P.; Fernández, R.; Amade, P. Cytotoxic and Haemolytic Steroidal Glycosides from the Caribbean Sponge *Pandaros acanthifolium*. *Steroids* **2011**, *76*, 1389–1396, doi:10.1016/j.steroids.2011.07.010.
46. Regalado, E.L.; Jiménez-Romero, C.; Genta-Jouve, G.; Tasdemir, D.; Amade, P.; Nogueiras, C.; Thomas, O.P. Acanthifoliosides, Minor Steroidal Saponins from the Caribbean Sponge *Pandaros acanthifolium*. *Tetrahedron* **2011**, *67*, 1011–1018, doi:10.1016/j.tet.2010.11.103.
47. Berrué, F.; McCulloch, M.W.B.; Boland, P.; Hart, S.; Harper, M.K.; Johnston, J.; Kerr, R. Isolation of Steroidal Glycosides from the Caribbean Sponge *Pandaros acanthifolium*. *J. Nat. Prod.* **2012**, *75*, 2094–2100, doi:10.1021/np300520w.
48. Ando, H.; Ueoka, R.; Okada, S.; Fujita, T.; Iwashita, T.; Imai, T.; Yokoyama, T.; Matsumoto, Y.; van Soest, R.W.M.; Matsunaga, S. Penasins A−E, Long-Chain Cytotoxic Sphingoid Bases, from a Marine Sponge *Penares* sp. *J. Nat. Prod.* **2010**, *73*, 1947–1950, doi:10.1021/np1003565.
49. Gaspar, H.; Cutignano, A.; Grauso, L.; Neng, N.; Cachatra, V.; Fontana, A.; Xavier, J.; Cerejo, M.; Vieira, H.; Santos, S. Erylusamides: Novel Atypical Glycolipids from *Erylus* cf. *deficiens*. *Marine Drugs* **2016**, *14*, 179, doi:10.3390/md14100179.
50. Shen, S.; Liu, D.; Wei, C.; Proksch, P.; Lin, W. Purpuroines A–J, Halogenated Alkaloids from the Sponge *Iotrochota purpurea* with Antibiotic Activity and Regulation of Tyrosine Kinases. *Bioorganic & Medicinal Chemistry* **2012**, *20*, 6924–6928, doi:10.1016/j.bmc.2012.10.014.
51. Feng, Y.; Davis, R.A.; Sykes, M.L.; Avery, V.M.; Quinn, R.J. Iotrochamides A and B, Antitrypanosomal Compounds from the Australian Marine Sponge *Iotrochota* sp. *Bioorganic & Medicinal Chemistry Letters* **2012**, *22*, 4873–4876, doi:10.1016/j.bmcl.2012.05.029.
52. Martín, M.J.; Rodríguez-Acebes, R.; García-Ramos, Y.; Martínez, V.; Murcia, C.; Digón, I.; Marco, I.; Pelay-Gimeno, M.; Fernández, R.; Reyes, F.; et al. Stellatolides, a New Cyclodepsipeptide Family from the Sponge *Ecionemia acervus*: Isolation, Solid-Phase Total Synthesis, and Full Structural Assignment of Stellatolide A. *J. Am. Chem. Soc.* **2014**, *136*, 6754–6762, doi:10.1021/ja502744a.

53. Di, X.; Rouger, C.; Hardardottir, I.; Freysdottir, J.; Molinski, T.F.; Tasdemir, D.; Omarsdottir, S. 6-Bromoindole Derivatives from the Icelandic Marine Sponge *Geodia barretti*: Isolation and Anti-Inflammatory Activity. *Mar Drugs* **2018**, *16*, E437, doi:10.3390/md16110437.
54. Knestrick, M.A.; Wilson, N.G.; Roth, A.; Adams, J.H.; Baker, B.J. Friomaramide, a Highly Modified Linear Hexapeptide from an Antarctic Sponge, Inhibits Plasmodium Falciparum Liver-Stage Development. *J Nat Prod* **2019**, *82*, 2354–2358, doi:10.1021/acs.jnatprod.9b00362.
55. Zhang, H.; Conte, M.M.; Capon, R.J. Franklinolides A–C from an Australian Marine Sponge Complex: Phosphodiesters Strongly Enhance Polyketide Cytotoxicity. *Angewandte Chemie International Edition* **2010**, *49*, 9904–9906, doi:10.1002/anie.201005883.
56. Martín, M.J.; Coello, L.; Fernández, R.; Reyes, F.; Rodríguez, A.; Murcia, C.; Garranzo, M.; Mateo, C.; Sánchez-Sancho, F.; Bueno, S.; et al. Isolation and First Total Synthesis of PM050489 and PM060184, Two New Marine Anticancer Compounds. *J. Am. Chem. Soc.* **2013**, *135*, 10164–10171, doi:10.1021/ja404578u.
57. Tähtinen, P.; Guella, G.; Saielli, G.; Debitus, C.; Hnawia, E.; Mancini, I. New Sulfur-Containing Polyarsenicals from the New Caledonian Sponge *Echinochalina bargibanti*. *Mar Drugs* **2018**, *16*, E382, doi:10.3390/md16100382.
58. Ota, K.; Hamamoto, Y.; Eda, W.; Tamura, K.; Sawada, A.; Hoshino, A.; Mitome, H.; Kamaike, K.; Miyaoka, H. Amitorines A and B, Nitrogenous Diterpene Metabolites of *Theonella swinhoei*: Isolation, Structure Elucidation, and Asymmetric Synthesis. *J. Nat. Prod.* **2016**, *79*, 996–1004, doi:10.1021/acs.jnatprod.5b01069.
59. Abdjul, D.B.; Yamazaki, H.; Takahashi, O.; Kirikoshi, R.; Ukai, K.; Namikoshi, M. Isopetrosynol, a New Protein Tyrosine Phosphatase 1B Inhibitor, from the Marine Sponge *Halichondria* cf. *panicea* Collected at Iriomote Island. *Chemical and Pharmaceutical Bulletin* **2016**, *64*, 733–736, doi:10.1248/cpb.c16-00061.
60. Wright, A.E.; Roberts, J.C.; Guzmán, E.A.; Pitts, T.P.; Pomponi, S.A.; Reed, J.K. Analogues of the Potent Antitumor Compound Leiodermatolide from a Deep-Water Sponge of the Genus *Leiodermatium*. *J. Nat. Prod.* **2017**, *80*, 735–739, doi:10.1021/acs.jnatprod.6b01140.
61. Cheng, Z.-B.; Xiao, H.; Fan, C.-Q.; Lu, Y.-N.; Zhang, G.; Yin, S. Bioactive Polyhydroxylated Sterols from the Marine Sponge *Haliclona crassiloba*. *Steroids* **2013**, *78*, 1353–1358, doi:10.1016/j.steroids.2013.10.004.
62. Ushiyama, S.; Umaoka, H.; Kato, H.; Suwa, Y.; Morioka, H.; Rotinsulu, H.; Losung, F.; Mangindaan, R.E.P.; de Voogd, N.J.; Yokosawa, H.; et al. Manadosterols A and B, Sulfonated Sterol Dimers Inhibiting the Ubc13–Uev1A Interaction, Isolated from the Marine Sponge *Lissodendryx fibrosa*. *J. Nat. Prod.* **2012**, *75*, 1495–1499, doi:10.1021/np300352u.
63. Viegelmann, C.; Parker, J.; Ooi, T.; Clements, C.; Abbott, G.; Young, L.; Kennedy, J.; Dobson, A.D.W.; Edrada-Ebel, R. Isolation and Identification of Antitrypanosomal and Antimycobacterial Active Steroids from the Sponge *Haliclona simulans*. *Marine Drugs* **2014**, *12*, 2937–2952, doi:10.3390/md12052937.

64. Tabakmakher, K.M.; Makarieva, T.N.; Denisenko, V.A.; Popov, R.S.; Dmitrenok, P.S.; Dyshlovoy, S.A.; Grebnev, B.B.; Bokemeyer, C.; von Amsberg, G.; Cuong, N.X. New Trisulfated Steroids from the Vietnamese Marine Sponge *Halichondria vansoesti* and Their PSA Expression and Glucose Uptake Inhibitory Activities. *Mar Drugs* **2019**, *17*, E445, doi:10.3390/md17080445.
65. Kolesnikova, S.A.; Lyakhova, E.G.; Kalinovsky, A.I.; Popov, R.S.; Yurchenko, E.A.; Stonik, V.A. Oxysterols from a Marine Sponge *Inflatella* sp. and Their Action in 6-Hydroxydopamine-Induced Cell Model of Parkinson's Disease. *Mar Drugs* **2018**, *16*, E458, doi:10.3390/md16110458.
66. Suto, S.; Tanaka, N.; Fromont, J.; Kobayashi, J. Halichonadins G–J, New Sesquiterpenoids from a Sponge *Halichondria* sp. *Tetrahedron Letters* **2011**, *52*, 3470–3473, doi:10.1016/j.tetlet.2011.04.105.
67. Trianto, A.; Hermawan, I.; Voogd, N.J. de; Tanaka, J. Halioxepine, a New Meroditerpene from an Indonesian Sponge *Haliclona* sp. *Chemical and Pharmaceutical Bulletin* **2011**, *59*, 1311–1313, doi:10.1248/cpb.59.1311.
68. Williams, D.E.; Steinø, A.; de Voogd, N.J.; Mauk, A.G.; Andersen, R.J. Halicloic Acids A and B Isolated from the Marine Sponge *Haliclona* sp. Collected in the Philippines Inhibit Indoleamine 2,3-Dioxygenase. *J. Nat. Prod.* **2012**, *75*, 1451–1458, doi:10.1021/np300345j.
69. Ohno, O.; Chiba, T.; Todoroki, S.; Yoshimura, H.; Maru, N.; Maekawa, K.; Imagawa, H.; Yamada, K.; Wakamiya, A.; Suenaga, K.; et al. Halichonines A, B, and C, Novel Sesquiterpene Alkaloids from the Marine Sponge *Halichondria okadai* Kadota. *Chem. Commun.* **2011**, *47*, 12453–12455, doi:10.1039/C1CC15557A.
70. Singh, A.J.; Dattelbaum, J.D.; Field, J.J.; Smart, Z.; Woolly, E.F.; Barber, J.M.; Heathcott, R.; Miller, J.H.; Northcote, P.T. Structurally Diverse Hamigerans from the New Zealand Marine Sponge *Hamigera tarangaensis*: NMR-Directed Isolation, Structure Elucidation and Antifungal Activity. *Org. Biomol. Chem.* **2013**, *11*, 8041–8051, doi:10.1039/C3OB41305E.
71. Tang, S.; Xu, R.; Lin, W.; Duan, H. Jaspiferin A and B: Two New Secondary Metabolites from the South China Sea Sponge *Jaspis stellifera*. *Rec. Nat. Prod.* **2012**, 4.
72. Paterson, I.; Dalby, S.M.; Roberts, J.C.; Naylor, G.J.; Guzmán, E.A.; Isbrucker, R.; Pitts, T.P.; Linley, P.; Divlianska, D.; Reed, J.K.; et al. Leiodermatolide, a Potent Antimitotic Macrolide from the Marine Sponge *Leiodermatium* sp. *Angewandte Chemie International Edition* **2011**, *50*, 3219–3223, doi:10.1002/anie.201007719.
73. Sirirak, T.; Kittiwisut, S.; Janma, C.; Yuenyongsawad, S.; Suwanborirux, K.; Plubrukarn, A. Kabiramides J and K, Trisoxazole Macrolides from the Sponge *Pachastrissa nux*. *J. Nat. Prod.* **2011**, *74*, 1288–1292, doi:10.1021/np100886y.
74. Antonov, A.S.; Kalinovsky, A.I.; Afiyatullov, S.S.; Leshchenko, E.V.; Dmitrenok, P.S.; Yurchenko, E.A.; Kalinin, V.I.; Stonik, V.A. Erylosides F8, V1–V3, and W–W2 – New Triterpene Oligoglycosides from the Carribean Sponge *Erylus goffrilleri*. *Carbohydrate Research* **2017**, *449*, 153–159, doi:10.1016/j.carres.2017.08.001.

75. Prawat, H.; Mahidol, C.; Kaweetripob, W.; Prachyawarakorn, V.; Tuntiwachwuttikul, P.; Ruchirawat, S. Sesquiterpene Isocyanides, Isothiocyanates, Thiocyanates, and Formamides from the Thai Sponge *Halichondria* sp. *Tetrahedron* **2016**, *72*, 4222–4229, doi:10.1016/j.tet.2016.05.060.
76. Lee, K.; Rho, J.-R. A New Sesterterpenoid Showing Anti-Inflammatory Effect from the Marine Sponge *Haliclona* species. *Journal of the Korean Magnetic Resonance Society* **2015**, *19*, 23–28, doi:10.6564/JKMRS.2015.19.1.023.
77. Tarazona, G.; Benedit, G.; Fernández, R.; Pérez, M.; Rodríguez, J.; Jiménez, C.; Cuevas, C. Can Stereoclusters Separated by Two Methylene Groups Be Related by DFT Studies? The Case of the Cytotoxic Meroditerpenes *Halioxepines*. *J Nat Prod* **2018**, *81*, 343–348, doi:10.1021/acs.jnatprod.7b00807.
78. Dattelbaum, J.D.; Singh, A.J.; Field, J.J.; Miller, J.H.; Northcote, P.T. The Nitrogenous Hamigerans: Unusual Amino Acid-Derivatized Aromatic Diterpenoid Metabolites from the New Zealand Marine Sponge *Hamigera tarangaensis*. *J. Org. Chem.* **2015**, *80*, 304–312, doi:10.1021/jo502370b.
79. Ahmadi, P.; Haruyama, T.; Kobayashi, N.; Voogd, N.J. de; Tanaka, J. Spongian Diterpenes from the Sponge *Hyattella* aff. *intestinalis*. *Chemical and Pharmaceutical Bulletin* **2017**, *65*, 874–877, doi:10.1248/cpb.c17-00297.
80. Fang, S.-T.; Yan, B.-F.; Yang, C.-Y.; Miao, F.-P.; Ji, N.-Y. Hymerhabdrin A, a Novel Diterpenoid with Antifouling Activity from the Intertidal Sponge *Hymerhabdia* sp. *J Antibiot* **2017**, *70*, 1043–1046, doi:10.1038/ja.2017.109.
81. Dai, J.; Jiménez, J.I.; Kelly, M.; Williams, P.G. Dictazoles: Potential Vinyl Cyclobutane Biosynthetic Precursors to the Dictazolines. *J. Org. Chem.* **2010**, *75*, 2399–2402, doi:10.1021/jo902566n.
82. Prawat, H.; Mahidol, C.; Kaweetripob, W.; Wittayalai, S.; Ruchirawat, S. Iodo–Sesquiterpene Hydroquinone and Brominated Indole Alkaloids from the Thai Sponge *Smenospongia* sp. *Tetrahedron* **2012**, *68*, 6881–6886, doi:10.1016/j.tet.2012.06.018.
83. Sakurada, T.; Gill, M.B.; Frausto, S.; Copits, B.; Noguchi, K.; Shimamoto, K.; Swanson, G.T.; Sakai, R. Novel N-Methylated 8-Oxoisoguanines from Pacific Sponges with Diverse Neuroactivities. *J. Med. Chem.* **2010**, *53*, 6089–6099, doi:10.1021/jm100490m.
84. Caso, A.; Esposito, G.; Della Sala, G.; Pawlik, J.R.; Teta, R.; Mangoni, A.; Costantino, V. Fast Detection of Two Smenamide Family Members Using Molecular Networking. *Mar Drugs* **2019**, *17*, E618, doi:10.3390/md17110618.
85. Yang, I.; Choi, H.; Nam, S.-J.; Kang, H. Two Indole-Alkaloids from a Korean Marine Sponge *Spongia* sp. *Bulletin of the Korean Chemical Society* **2015**, *36*, 2120–2123, doi:10.1002/bkcs.10372.
86. Salim, A.A.; Rae, J.; Fontaine, F.; Conte, M.M.; Khalil, Z.; Martin, S.; Parton, R.G.; Capon, R.J. Heterofibrins: Inhibitors of Lipid Droplet Formation from a Deep-Water Southern Australian Marine Sponge, *Spongia* (*Heterofibria*) sp. *Org. Biomol. Chem.* **2010**, *8*, 3188–3194, doi:10.1039/C003840G.
87. Kubota, T.; Suzuki, H.; Takahashi-Nakaguchi, A.; Fromont, J.; Gonoi, T.; Kobayashi, J. Taurospongins B and C, New Acetylenic Fatty Acid Derivatives Possessing a Taurine Amide Residue from a Marine Sponge of the Family Spongiidae. *RSC Adv.* **2014**, *4*, 11073–11079, doi:10.1039/C3RA47796G.

88. Zhang, H.; Crews, P.; Tenney, K.; Valeriote, F.A. Cytotoxic Phyllactone Analogs from the Marine Sponge *Phyllospongia papyrecea*. *Medicinal Chemistry* **2017**, *13*, 295–300.
89. Esposito, G.; Teta, R.; Miceli, R.; Ceccarelli, L.S.; Della Sala, G.; Camerlingo, R.; Irollo, E.; Mangoni, A.; Pirozzi, G.; Costantino, V. Isolation and Assessment of the in Vitro Anti-Tumor Activity of Smenothiazole A and B, Chlorinated Thiazole-Containing Peptide/Polyketides from the Caribbean Sponge, *Smenospongia aurea*. *Mar Drugs* **2015**, *13*, 444–459, doi:10.3390/md13010444.
90. Huyen, L.T.; Hang, D.T.T.; Nhiem, N.X.; Yen, P.H.; Anh, H.L.T.; Quang, T.H.; Tai, B.H.; Dau, N.V.; Kiem, P.V. Naphtoquinones and Sesquiterpene Cyclopentenones from the Sponge *Smenospongia cerebriformis* with Their Cytotoxic Activity. *Chemical and Pharmaceutical Bulletin* **2017**, *65*, 589–592, doi:10.1248/cpb.c17-00123.
91. Puilingi, C.G.; Kudo, Y.; Cho, Y.; Konoki, K.; Yotsu-Yamashita, M. A New Sarasinoside Congener, Sarasinoside M2, from a Marine Sponge Collected in the Solomon Islands. *Bioscience, Biotechnology, and Biochemistry* **2017**, *81*, 222–225, doi:10.1080/09168451.2016.1246172.
92. Nguyen, H.M.; Ito, T.; Kurimoto, S.; Ogawa, M.; Win, N.N.; Hung, V.Q.; Nguyen, H.T.; Kubota, T.; Kobayashi, J.; Morita, H. New Merosesquiterpenes from a Vietnamese Marine Sponge of *Spongia* sp. and Their Biological Activities. *Bioorganic & Medicinal Chemistry Letters* **2017**, *27*, 3043–3047, doi:10.1016/j.bmcl.2017.05.060.
93. Youssef, D.T.A.; Ibrahim, A.K.; Khalifa, S.I.; Mesbah, M.K.; Mayer, A.M.S.; van Soest, R.W.M. New Antiinflammatory Sterols from the Red Sea Sponges Scalarispongia Aqabaensis and *Callyspongia siphonella*. *Natural Product Communications* **2010**, *5*, 27–31, doi:10.1177/1934578X1000500107.
94. Lee, Y.-J.; Lee, J.-W.; Lee, D.-G.; Lee, H.-S.; Kang, J.S.; Yun, J. Cytotoxic Sesterterpenoids Isolated from the Marine Sponge *Scalarispongia* sp. *International Journal of Molecular Sciences* **2014**, *15*, 20045–20053, doi:10.3390/ijms151120045.
95. Balansa, W.; Islam, R.; Fontaine, F.; Piggott, A.M.; Zhang, H.; Xiao, X.; Webb, T.I.; Gilbert, D.F.; Lynch, J.W.; Capon, R.J. Sesterterpene Glycinyl-Lactams: A New Class of Glycine Receptor Modulator from Australian Marine Sponges of the Genus *Psammocinia*. *Org. Biomol. Chem.* **2013**, *11*, 4695–4701, doi:10.1039/C3OB40861B.
96. He, W.; Lin, X.; Xu, T.; Jung, J.H.; Yin, H.; Yang, B.; Liu, Y. A New Norsesterterpenoid from the Sponge Species *Sarcotragus*. *Chemistry of Natural Compounds* **2012**, *48*, 208–210, doi:10.1007/s10600-012-0206-4.
97. Abed, C.; Legrave, N.; Dufies, M.; Robert, G.; Guérineau, V.; Vacelet, J.; Auberger, P.; Amade, P.; Mehiri, M. A New Hydroxylated Nonaprenylhydroquinone from the Mediterranean Marine Sponge *Sarcotragus spinosulus*. *Marine Drugs* **2011**, *9*, 1210–1219, doi:10.3390/md9071210.
98. Manzo, E.; Ciavatta, M.L.; Villani, G.; Varcamonti, M.; Sayem, S.M.A.; van Soest, R.; Gavagnin, M. Bioactive Terpenes from *Spongia officinalis*. *J. Nat. Prod.* **2011**, *74*, 1241–1247, doi:10.1021/np200226u.

99. Takahashi, Y.; Ushio, M.; Kubota, T.; Yamamoto, S.; Fromont, J.; Kobayashi, J. Nakijiquinones J−R, Sesquiterpenoid Quinones with an Amine Residue from Okinawan Marine Sponges. *J. Nat. Prod.* **2010**, *73*, 467–471, doi:10.1021/np900470e.
100. Ponomarenko, L.P.; Terent'eva, N.A.; Krasokhin, V.B.; Kalinovsky, A.I.; Rasskazov, V.A. Terpenoid Metabolites from *Spongia* sp. and Their Effects on Nucleic Acid Biosynthesis in Sea Urchin Eggs. *Nat Prod Commun* **2011**, *6*, 773–776.
101. Ovenden, S.P.B.; Nielson, J.L.; Liptrot, C.H.; Willis, R.H.; Tapiolas, D.M.; Wright, A.D.; Motti, C.A. Metachromins U–W: Cytotoxic Merosesquiterpenoids from an Australian Specimen of the Sponge *Thorecta reticulata*. *J. Nat. Prod.* **2011**, *74*, 1335–1338, doi:10.1021/np200041v.
102. Hassan, M.H.A.; Rateb, M.; Hetta, M.; Abdelaziz, T.A.; Sleim, M.A.; Jaspars, M.; Mohammed, R. Scalarane Sesterterpenes from the Egyptian Red Sea Sponge *Phyllospongia lamellose*. *Tetrahedron* **2015**, *71*, 577–583, doi:10.1016/j.tet.2014.12.035.
103. Lee, S.M.; Kim, N.-H.; Lee, S.; Kim, Y.N.; Heo, J.D.; Jeong, E.J.; Rho, J.-R. Deacetylphylloketal, a New Phylloketal Derivative from a Marine Sponge, Genus *Phyllospongia*, with Potent Anti-Inflammatory Activity in In Vitro Co-Culture Model of Intestine. *Mar Drugs* **2019**, *17*, E634, doi:10.3390/md17110634.
104. Woo, J.-K.; Jeon, J.; Kim, B.; Sim, C.J.; Oh, D.-C.; Oh, K.-B.; Shin, J. A New Sesterterpene from the Korean *Sarcotragus* sp. Sponge. *Nat Prod Sci* **2015**, *21*, 237–239, doi:10.20307/nps.2015.21.4.237.
105. Lee, Y.-J.; Kim, S.H.; Choi, H.; Lee, H.-S.; Lee, J.S.; Shin, H.J.; Lee, J. Cytotoxic Furan- and Pyrrole-Containing Scalarane Sesterterpenoids Isolated from the Sponge *Scalarispongia* sp. *Molecules* **2019**, *24*, E840, doi:10.3390/molecules24050840.
106. Van Kiem, P.; Huyen, L.T.; Hang, D.T.; Nhiem, N.X.; Tai, B.H.; Anh, H.L.T.; Cuong, P.V.; Quang, T.H.; Minh, C.V.; Dau, N.V.; et al. Sesquiterpene Derivatives from Marine Sponge *Smenospongia cerebriformis* and Their Anti-Inflammatory Activity. *Bioorganic & Medicinal Chemistry Letters* **2017**, *27*, 1525–1529, doi:10.1016/j.bmcl.2017.02.040.
107. Hwang, I.H.; Oh, J.; Zhou, W.; Park, S.; Kim, J.-H.; Chittiboyina, A.G.; Ferreira, D.; Song, G.Y.; Oh, S.; Na, M.; et al. Cytotoxic Activity of Rearranged Drimane Meroterpenoids against Colon Cancer Cells via Down-Regulation of β-Catenin Expression. *J. Nat. Prod.* **2015**, *78*, 453–461, doi:10.1021/np500843m.
108. El-Desoky, A.H.; Kato, H.; Angkouw, E.D.; Mangindaan, R.E.P.; de Voogd, N.J.; Tsukamoto, S. Ceylonamides A–F, Nitrogenous Spongian Diterpenes That Inhibit RANKL-Induced Osteoclastogenesis, from the Marine Sponge *Spongia ceylonensis*. *J. Nat. Prod.* **2016**, *79*, 1922–1928, doi:10.1021/acs.jnatprod.6b00158.
109. El-Desoky, A.H.; Kato, H.; Kagiyama, I.; Hitora, Y.; Losung, F.; Mangindaan, R.E.P.; de Voogd, N.J.; Tsukamoto, S. Ceylonins A–F, Spongian Diterpene Derivatives That Inhibit RANKL-Induced Formation of Multinuclear Osteoclasts, from the Marine Sponge *Spongia ceylonensis*. *J. Nat. Prod.* **2017**, *80*, 90–95, doi:10.1021/acs.jnatprod.6b00725.
110. Chen, Q.; Mao, Q.; Bao, M.; Mou, Y.; Fang, C.; Zhao, M.; Jiang, W.; Yu, X.; Wang, C.; Dai, L.; et al. Spongian Diterpenes Including One with a Rearranged Skeleton from the Marine Sponge *Spongia officinalis*. *J Nat Prod* **2019**, *82*, 1714–1718, doi:10.1021/acs.jnatprod.9b00270.

111. Nguyen, H.M.; Ito, T.; Win, N.N.; Kodama, T.; Hung, V.Q.; Nguyen, H.T.; Morita, H. New Antibacterial Sesquiterpene Aminoquinones from a Vietnamese Marine Sponge of *Spongia* sp. *Phytochemistry Letters* **2016**, *17*, 288–292, doi:10.1016/j.phytol.2016.08.012.
112. Ito, T.; Nguyen, H.M.; Win, N.N.; Vo, H.Q.; Nguyen, H.T.; Morita, H. Three New Sesquiterpene Aminoquinones from a Vietnamese *Spongia* sp. and Their Biological Activities. *J Nat Med* **2018**, *72*, 298–303, doi:10.1007/s11418-017-1130-5.
113. Phan, C.-S.; Kamada, T.; Hamada, T.; Vairappan, C.S. Cytotoxic Sesterterpenoids from Bornean Sponge *Spongia* sp. *Rec.Nat.Prod.* **2018**, *12*, 643–647, doi:10.25135/rnp.69.18.01.209.
114. Liang, Y.-Q.; Liao, X.-J.; Lin, J.-L.; Xu, W.; Chen, G.-D.; Zhao, B.-X.; Xu, S.-H. Spongiains A-C: Three New Spongian Diterpenes with Ring A Rearrangement from the Marine Sponge *Spongia* sp. *Tetrahedron* **2019**, *75*, 3802–3808, doi:10.1016/j.tet.2019.06.001.
115. Jomori, T.; Setiawan, A.; Sasaoka, M.; Arai, M. Cytotoxicity of New Diterpene Alkaloids, Ceylonamides G-I, Isolated From Indonesian Marine Sponge of *Spongia* sp. *Natural Product Communications* **2019**, *14*, 1934578X19857294, doi:10.1177/1934578X19857294.
116. Sánchez, J.A.; Alfonso, A.; Leirós, M.; Alonso, E.; Rateb, M.E.; Jaspars, M.; Houssen, W.E.; Ebel, R.; Botana, L.M. *Spongionella* Secondary Metabolites Regulate Store Operated Calcium Entry Modulating Mitochondrial Functioning in SH-SY5Y Neuroblastoma Cells. *CPB* **2015**, *37*, 779–792, doi:10.1159/000430395.
117. Xu, M.; Davis, R.A.; Feng, Y.; Sykes, M.L.; Shelper, T.; Avery, V.M.; Camp, D.; Quinn, R.J. Ianthelliformisamines A–C, Antibacterial Bromotyrosine-Derived Metabolites from the Marine Sponge *Suberea ianthelliformis*. *J. Nat. Prod.* **2012**, *75*, 1001–1005, doi:10.1021/np300147d.
118. Mani, L.; Jullian, V.; Mourkazel, B.; Valentin, A.; Dubois, J.; Cresteil, T.; Folcher, E.; Hooper, J.N.A.; Erpenbeck, D.; Aalbersberg, W.; et al. New Antiplasmodial Bromotyrosine Derivatives from *Suberea ianthelliformis* Lendenfeld, 1888. *Chemistry & Biodiversity* **2012**, *9*, 1436–1451, doi:10.1002/cbdv.201100309.
119. Lee, Y.-J.; Han, S.; Lee, H.-S.; Kang, J.S.; Yun, J.; Sim, C.J.; Shin, H.J.; Lee, J.S. Cytotoxic Psammaplysin Analogues from a *Suberea* sp. Marine Sponge and the Role of the Spirooxepinisoxazoline in Their Activity. *J. Nat. Prod.* **2013**, *76*, 1731–1736, doi:10.1021/np400448y.
120. El-Demerdash, A.; Moriou, C.; Toullec, J.; Besson, M.; Soulet, S.; Schmitt, N.; Petek, S.; Lecchini, D.; Debitus, C.; Al-Mourabit, A. Bioactive Bromotyrosine-Derived Alkaloids from the Polynesian Sponge *Suberea ianthelliformis*. *Mar Drugs* **2018**, *16*, E146, doi:10.3390/md16050146.
121. Shaala, L.A.; Youssef, D.T.A.; Badr, J.M.; Sulaiman, M.; Khedr, A. Bioactive Secondary Metabolites from the Red Sea Marine Verongid Sponge *Suberea* species. *Marine Drugs* **2015**, *13*, 1621–1631, doi:10.3390/md13041621.
122. Kurimoto, S.-I.; Seino, S.; Fromont, J.; Kobayashi, J.; Kubota, T. Ma'edamines C and D, New Bromotyrosine Alkaloids Possessing a Unique Tetrasubstituted Pyridinium Moiety from an Okinawan Marine Sponge *Suberea* sp. *Org Lett* **2019**, *21*, 8824–8826, doi:10.1021/acs.orglett.9b03457.
123. Shaala, L.A.; Bamane, F.H.; Badr, J.M.; Youssef, D.T.A. Brominated Arginine-Derived Alkaloids from the Red Sea Sponge *Suberea mollis*. *J. Nat. Prod.* **2011**, *74*, 1517–1520, doi:10.1021/np200120d.

124. Shaker, K.H.; Zinecker, H.; Ghani, M.A.; Imhoff, J.F.; Schneider, B. Bioactive Metabolites from the Sponge *Suberea* sp. *Chemistry & Biodiversity* **2010**, *7*, 2880–2887, doi:10.1002/cbdv.200900277.
125. Arai, M.; Han, C.; Yamano, Y.; Setiawan, A.; Kobayashi, M. Aaptamines, Marine Spongean Alkaloids, as Anti-Dormant Mycobacterial Substances. *J Nat Med* **2014**, *68*, 372–376, doi:10.1007/s11418-013-0811-y.
126. Yu, H.-B.; Yang, F.; Sun, F.; Li, J.; Jiao, W.-H.; Gan, J.-H.; Hu, W.-Z.; Lin, H.-W. Aaptamine Derivatives with Antifungal and Anti-HIV-1 Activities from the South China Sea Sponge *Aaptos aaptos*. *Marine Drugs* **2014**, *12*, 6003–6013, doi:10.3390/md12126003.
127. Yu, H.-B.; Yang, F.; Sun, F.; Ma, G.-Y.; Gan, J.-H.; Hu, W.-Z.; Han, B.-N.; Jiao, W.-H.; Lin, H.-W. Cytotoxic Aaptamine Derivatives from the South China Sea Sponge *Aaptos aaptos*. *J. Nat. Prod.* **2014**, *77*, 2124–2129, doi:10.1021/np500583z.
128. Farrugia, M.; Trotter, N.; Vijayasarathy, S.; Salim, A.A.; Khalil, Z.G.; Lacey, E.; Capon, R.J. Isolation and Synthesis of N-Acyladenine and Adenosine Alkaloids from a Southern Australian Marine Sponge, *Phoriospongia* sp. *Tetrahedron Letters* **2014**, *55*, 5902–5904, doi:10.1016/j.tetlet.2014.08.116.
129. Kusama, T.; Tanaka, N.; Sakai, K.; Gonoi, T.; Fromont, J.; Kashiwada, Y.; Kobayashi, J. Agelamadins A and B, Dimeric Bromopyrrole Alkaloids from a Marine Sponge *Agelas* sp. *Org Lett* **2014**, *16*, 3916–3918, doi:10.1021/ol501664b.
130. Kusama, T.; Tanaka, N.; Sakai, K.; Gonoi, T.; Fromont, J.; Kashiwada, Y.; Kobayashi, J. Agelamadins C–E, Bromopyrrole Alkaloids Comprising Oroidin and 3-Hydroxykynurenine from a Marine Sponge *Agelas* sp. *Org. Lett.* **2014**, *16*, 5176–5179, doi:10.1021/ol502528m.
131. Shubina, L.K.; Makarieva, T.N.; Dyshlovoy, S.A.; Fedorov, S.N.; Dmitrenok, P.S.; Stonik, V.A. Three New Aaptamines from the Marine Sponge *Aaptos* sp. and Their Proapoptotic Properties. *Natural Product Communications* **2010**, *5*, 1934578X1000501208, doi:10.1177/1934578X1000501208.
132. Pham, C.-D.; Hartmann, R.; Müller, W.E.G.; de Voogd, N.; Lai, D.; Proksch, P. Aaptamine Derivatives from the Indonesian Sponge Aaptos Suberitoides. *J. Nat. Prod.* **2013**, *76*, 103–106, doi:10.1021/np300794b.
133. Yang, F.; Ji, R.-H.; Li, J.; Gan, J.-H.; Lin, H.-W. N-Containing Metabolites from the Marine Sponge *Agelas clathrodes*. *Nat Prod Commun* **2013**, *8*, 1713–1714.
134. Hertiani, T.; Edrada-Ebel, R.; Ortlepp, S.; van Soest, R.W.M.; de Voogd, N.J.; Wray, V.; Hentschel, U.; Kozytska, S.; Müller, W.E.G.; Proksch, P. From Anti-Fouling to Biofilm Inhibition: New Cytotoxic Secondary Metabolites from Two Indonesian *Agelas sponges*. *Bioorg Med Chem* **2010**, *18*, 1297–1311, doi:10.1016/j.bmc.2009.12.028.
135. Yang, F.; Hamann, M.T.; Zou, Y.; Zhang, M.-Y.; Gong, X.-B.; Xiao, J.-R.; Chen, W.-S.; Lin, H.-W. Antimicrobial Metabolites from the Paracel Islands Sponge *Agelas mauritiana*. *J. Nat. Prod.* **2012**, *75*, 774–778, doi:10.1021/np2009016.
136. Tanaka, N.; Kusama, T.; Takahashi-Nakaguchi, A.; Gonoi, T.; Fromont, J.; Kobayashi, J. Nagelamides U–W, Bromopyrrole Alkaloids from a Marine Sponge *Agelas* sp. *Tetrahedron Letters* **2013**, *54*, 3794–3796, doi:10.1016/j.tetlet.2013.05.023.

137. Kura, K.; Kubota, T.; Fromont, J.; Kobayashi, J. Pyrinodemins E and F, New 3-Alkylpyridine Alkaloids from Sponge *Amphimedon* sp. *Bioorg Med Chem Lett* **2011**, *21*, 267–270, doi:10.1016/j.bmcl.2010.11.020.
138. Kubota, T.; Kamijyo, Y.; Takahashi-Nakaguchi, A.; Fromont, J.; Gonoi, T.; Kobayashi, J. Zamamiphidin A, a New Manzamine Related Alkaloid from an Okinawan Marine Sponge *Amphimedon* sp. *Org. Lett.* **2013**, *15*, 610–612, doi:10.1021/ol3034274.
139. Kubota, T.; Kura, K.; Fromont, J.; Kobayashi, J. Pyrinodemins G–I, New Bis-3-Alkylpyridine Alkaloids from a Marine Sponge *Amphimedon* sp. *Tetrahedron* **2013**, *69*, 96–100, doi:10.1016/j.tet.2012.10.062.
140. Russell, F.; Harmody, D.; McCarthy, P.J.; Pomponi, S.A.; Wright, A.E. Indolo[3,2-a]Carbazoles from a Deep-Water Sponge of the Genus *Asteropus*. *J. Nat. Prod.* **2013**, *76*, 1989–1992, doi:10.1021/np400501u.
141. Ueoka, R.; Ise, Y.; Okada, S.; Matsunaga, S. Cell Differentiation Inducers from a Marine Sponge *Biemna* sp. *Tetrahedron* **2011**, *67*, 6679–6681, doi:10.1016/j.tet.2011.04.098.
142. Roué, M.; Domart-Coulon, I.; Ereskovsky, A.; Djediat, C.; Perez, T.; Bourguet-Kondracki, M.-L. Cellular Localization of Clathridimine, an Antimicrobial 2-Aminoimidazole Alkaloid Produced by the Mediterranean Calcareous Sponge *Clathrina clathrus*. *J. Nat. Prod.* **2010**, *73*, 1277–1282, doi:10.1021/np100175x.
143. Bondu, S.; Genta-Jouve, G.; Leirós, M.; Vale, C.; Guigonis, J.-M.; Botana, L.M.; Thomas, O.P. Additional Bioactive Guanidine Alkaloids from the Mediterranean Sponge *Crambe crambe*. *RSC Adv.* **2012**, *2*, 2828–2835, doi:10.1039/C2RA00045H.
144. Guzii, A.G.; Makarieva, T.N.; Denisenko, V.A.; Dmitrenok, P.S.; Kuzmich, A.S.; Dyshlovoy, S.A.; Krasokhin, V.B.; Stonik, V.A. Monanchocidin: A New Apoptosis-Inducing Polycyclic Guanidine Alkaloid from the Marine Sponge *Monanchora pulchra*. *Org. Lett.* **2010**, *12*, 4292–4295, doi:10.1021/ol101716x.
145. Makarieva, T.N.; Tabakmaher, K.M.; Guzii, A.G.; Denisenko, V.A.; Dmitrenok, P.S.; Kuzmich, A.S.; Lee, H.-S.; Stonik, V.A. Monanchomycalins A and B, Unusual Guanidine Alkaloids from the Sponge *Monanchora pulchra*. *Tetrahedron Letters* **2012**, *53*, 4228–4231, doi:10.1016/j.tetlet.2012.05.162.
146. Guzii, A.G.; Makarieva, T.N.; Korolkova, Y.V.; Andreev, Y.A.; Mosharova, I.V.; Tabakmaher, K.M.; Denisenko, V.A.; Dmitrenok, P.S.; Ogurtsova, E.K.; Antonov, A.S.; et al. Pulchranin A, Isolated from the Far-Eastern Marine Sponge, *Monanchora pulchra*: The First Marine Non-Peptide Inhibitor of TRPV-1 Channels. *Tetrahedron Letters* **2013**, *54*, 1247–1250, doi:10.1016/j.tetlet.2012.12.099.
147. Tabakmakher, K.M.; Denisenko, V.A.; Guzii, A.G.; Dmitrenok, P.S.; Dyshlovoy, S.A.; Lee, H.-S.; Makarieva, T.N. Monanchomycalin C, a New Pentacyclic Guanidine Alkaloid from the Far-Eastern Marine Sponge *Monanchora pulchra*. *Nat Prod Commun* **2013**, *8*, 1399–1402.
148. Wei, X.; Nieves, K.; Rodríguez, A.D. Neopetrosiamine A, Biologically Active Bis-Piperidine Alkaloid from the Caribbean Sea Sponge *Neopetrosia proxima*. *Bioorganic & Medicinal Chemistry Letters* **2010**, *20*, 5905–5908, doi:10.1016/j.bmcl.2010.07.084.

149. Ilias, M.; Ibrahim, M.A.; Khan, S.I.; Jacob, M.R.; Tekwani, B.L.; Walker, L.A.; Samoylenko, V. Pentacyclic Ingamine Alkaloids, a New Antiplasmodial Pharmacophore from the Marine Sponge *Petrosid* Ng5 Sp5. *Planta Med* **2012**, *78*, 1690–1697, doi:10.1055/s-0032-1315213.
150. Nguyen, T.D.; Nguyen, X.C.; Longeon, A.; Keryhuel, A.; Le, M.H.; Kim, Y.H.; Chau, V.M.; Bourguet-Kondracki, M.-L. Antioxidant Benzylidene 2-Aminoimidazolones from the Mediterranean Sponge *Phorbas topsenti*. *Tetrahedron* **2012**, *68*, 9256–9259, doi:10.1016/j.tet.2012.08.074.
151. Jeon, J.; Na, Z.; Jung, M.; Lee, H.-S.; Sim, C.J.; Nahm, K.; Oh, K.-B.; Shin, J. Discorhabdins from the Korean Marine Sponge *Sceptrella* sp. *J. Nat. Prod.* **2010**, *73*, 258–262, doi:10.1021/np9005629.
152. Ovenden, S.P.B.; Nielson, J.L.; Liptrot, C.H.; Willis, R.H.; Tapiolas, D.M.; Wright, A.D.; Motti, C.A. A New Diketopiperazine, Cyclo-(4-S-Hydroxy-R-Proline-R-Isoleucine), from an Australian Specimen of the Sponge *Stelletta* sp. *Marine Drugs* **2011**, *9*, 2469–2478, doi:10.3390/md9112469.
153. Fouad, M.A.; Debbab, A.; Wray, V.; Müller, W.E.G.; Proksch, P. New Bioactive Alkaloids from the Marine Sponge *Stylissa* sp. *Tetrahedron* **2012**, *68*, 10176–10179, doi:10.1016/j.tet.2012.09.097.
154. Takahashi, Y.; Kubota, T.; Shibazaki, A.; Gonoi, T.; Fromont, J.; Kobayashi, J. Nakijinamines C–E, New Heteroaromatic Alkaloids from the Sponge *Suberites* species. *Org. Lett.* **2011**, *13*, 3016–3019, doi:10.1021/ol2008473.
155. Takahashi, Y.; Tanaka, N.; Kubota, T.; Ishiyama, H.; Shibazaki, A.; Gonoi, T.; Fromont, J.; Kobayashi, J. Heteroaromatic Alkaloids, Nakijinamines, from a Sponge *Suberites* sp. *Tetrahedron* **2012**, *68*, 8545–8550, doi:10.1016/j.tet.2012.08.018.
156. Angawi, R.F.; Bavestrello, G.; Calcinai, B.; Dien, H.A.; Donnarumma, G.; Tufano, M.A.; Paoletti, I.; Grimaldi, E.; Chianese, G.; Fattorusso, E.; et al. Aurantoside J: A New Tetramic Acid Glycoside from *Theonella swinhoei*. Insights into the Antifungal Potential of Aurantosides. *Marine Drugs* **2011**, *9*, 2809–2817, doi:10.3390/md9122809.
157. Festa, C.; De Marino, S.; Sepe, V.; D'Auria, M.V.; Bifulco, G.; Débitus, C.; Bucci, M.; Vellecco, V.; Zampella, A. Solomonamides A and B, New Anti-Inflammatory Peptides from *Theonella swinhoei*. *Org. Lett.* **2011**, *13*, 1532–1535, doi:10.1021/ol200221n.
158. Utkina, N.K.; Krasokhin, V.B. Tetrahydroisoquinoline Alkaloid N-Methylnorsalsolinol from the Australian Marine Sponge *Xestospongia* sp. *Chem Nat Compd* **2012**, *48*, 715–716, doi:10.1007/s10600-012-0364-4.
159. Davis, R.A.; Buchanan, M.S.; Duffy, S.; Avery, V.M.; Charman, S.A.; Charman, W.N.; White, K.L.; Shackleford, D.M.; Edstein, M.D.; Andrews, K.T.; et al. Antimalarial Activity of Pyrroloiminoquinones from the Australian Marine Sponge *Zyzzya* sp. *J. Med. Chem.* **2012**, *55*, 5851–5858, doi:10.1021/jm3002795.
160. Gan, J.-H.; Hu, W.-Z.; Yu, H.-B.; Yang, F.; Cao, M.-X.; Shi, H.-J.; Kang, Y.-F.; Han, B.-N. Three New Aaptamine Derivatives from the South China Sea Sponge *Aaptos aaptos*. *Journal of Asian Natural Products Research* **2015**, *17*, 1231–1238, doi:10.1080/10286020.2015.1118465.

161. Hamada, T.; Matsumoto, Y.; Phan, C.-S.; Kamada, T.; Onitsuka, S.; Okamura, H.; Iwagawa, T.; Arima, N.; Tani, F.; Vairappan, C.S. Aaptamine-Related Alkaloid from the Marine Sponge *Aaptos aaptos*. *Natural Product Communications* **2019**, *14*, 1934578X19863935, doi:10.1177/1934578X19863935.
162. Sirimangkalakitti, N.; Yokoya, M.; Chamni, S.; Chanvorachote, P.; Plubrukrn, A.; Saito, N.; Suwanborirux, K. Synthesis and Absolute Configuration of Acanthodendrilline, a New Cytotoxic Bromotyrosine Alkaloid from the Thai Marine Sponge *Acanthodendrilla* sp. *Chemical and Pharmaceutical Bulletin* **2016**, *64*, 258–262, doi:10.1248/cpb.c15-00901.
163. Esposito, G.; Bourguet-Kondracki, M.-L.; Mai, L.H.; Longeon, A.; Teta, R.; Meijer, L.; Van Soest, R.; Mangoni, A.; Costantino, V. Chloromethylhalicyclamine B, a Marine-Derived Protein Kinase CK1δ/ε Inhibitor. *J. Nat. Prod.* **2016**, *79*, 2953–2960, doi:10.1021/acs.jnatprod.6b00939.
164. Ibrahim, S.R.M.; Mohamed, G.A. Ingenines C and D, New Cytotoxic Pyrimidine-β-Carboline Alkaloids from the Indonesian Sponge *Acanthostrongylophora ingens*. *Phytochemistry Letters* **2016**, *18*, 168–171, doi:10.1016/j.phytol.2016.10.014.
165. Ibrahim, S.R.M.; Mohamed, G.A. Ingenine E, a New Cytotoxic β-Carboline Alkaloid from the Indonesian Sponge *Acanthostrongylophora ingens*. *Journal of Asian Natural Products Research* **2017**, *19*, 504–509, doi:10.1080/10286020.2016.1213723.
166. Ibrahim, S.; Mohamed, G.; Al Haidari, R.; El-Kholy, A.; Zayed, M. Ingenine F: A New Cytotoxic Tetrahydro Carboline Alkaloid from the Indonesian Marine Sponge *Acanthostrongylophora ingens*. *Pharmacogn Mag* **2018**, *14*, 231–234, doi:10.4103/pm.pm_489_17.
167. Kato, H.; El-Desoky, A.H.; Takeishi, Y.; Nehira, T.; Angkouw, E.D.; Mangindaan, R.E.P.; de Voogd, N.J.; Tsukamoto, S. Tetradehydrohalicyclamine B, a New Proteasome Inhibitor from the Marine Sponge *Acanthostrongylophora ingens*. *Bioorg Med Chem Lett* **2019**, *29*, 8–10, doi:10.1016/j.bmcl.2018.11.028.
168. Kim, C.-K.; Riswanto, R.; Won, T.H.; Kim, H.; Elya, B.; Sim, C.J.; Oh, D.-C.; Oh, K.-B.; Shin, J. Manzamine Alkaloids from an *Acanthostrongylophora* sp. Sponge. *J. Nat. Prod.* **2017**, *80*, 1575–1583, doi:10.1021/acs.jnatprod.7b00121.
169. Cychon, C.; Lichte, E.; Köck, M. The Marine Sponge *Agelas citrina* as a Source of the New Pyrrole–Imidazole Alkaloids Citrinamines A–D and N-Methylagelongine. *Beilstein J. Org. Chem.* **2015**, *11*, 2029–2037, doi:10.3762/bjoc.11.220.
170. Chu, M.-J.; Tang, X.-L.; Qin, G.-F.; de Voogd, N.J.; Li, P.-L.; Li, G.-Q. Three New Non-Brominated Pyrrole Alkaloids from the South China Sea Sponge *Agelas nakamurai*. *Chinese Chemical Letters* **2017**, *28*, 1210–1213, doi:10.1016/j.cclet.2017.01.009.
171. Chu, M.-J.; Tang, X.-L.; Qin, G.-F.; Sun, Y.-T.; Li, L.; de Voogd, N.J.; Li, P.-L.; Li, G.-Q. Pyrrole Derivatives and Diterpene Alkaloids from the South China Sea Sponge *Agelas nakamurai*. *Chemistry & Biodiversity* **2017**, *14*, e1600446, doi:10.1002/cbdv.201600446.
172. Li, T.; Wang, B.; de Voogd, N.J.; Tang, X.-L.; Wang, Q.; Chu, M.-J.; Li, P.-L.; Li, G.-Q. Two New Diterpene Alkaloids from the South China Sea Sponge *Agelas* aff. *nemoechinata*. *Chinese Chemical Letters* **2016**, *27*, 1048–1051, doi:10.1016/j.cclet.2016.05.017.

173. An, L.; Song, W.; Tang, X.; Voogd, N.J. de; Wang, Q.; Chu, M.; Li, P.; Li, G. Alkaloids and Polyketides from the South China Sea Sponge *Agelas* aff. *nemoechinata*. *RSC Adv.* **2017**, *7*, 14323–14329, doi:10.1039/C6RA27026C.
174. Li, T.; Li, P.-L.; Luo, X.-C.; Tang, X.-L.; Li, G.-Q. Three New Dibromopyrrole Alkaloids from the South China Sea Sponge *Agelas nemoechinata*. *Tetrahedron Letters* **2019**, *60*, 1996–1998, doi:10.1016/j.tetlet.2019.06.049.
175. Li, T.; Tang, X.; Luo, X.; Wang, Q.; Liu, K.; Zhang, Y.; de Voogd, N.J.; Yang, J.; Li, P.; Li, G. Agelanemoechine, a Dimeric Bromopyrrole Alkaloid with a Pro-Angiogenic Effect from the South China Sea Sponge *Agelas nemoechinata*. *Org. Lett.* **2019**, *21*, 9483–9486, doi:10.1021/acs.orglett.9b03683.
176. Zhu, Y.; Wang, Y.; Gu, B.-B.; Yang, F.; Jiao, W.-H.; Hu, G.-H.; Yu, H.-B.; Han, B.-N.; Zhang, W.; Shen, Y.; et al. Antifungal Bromopyrrole Alkaloids from the South China Sea Sponge *Agelas* sp. *Tetrahedron* **2016**, *72*, 2964–2971, doi:10.1016/j.tet.2016.04.020.
177. Sun, Y.-T.; Lin, B.; Li, S.-G.; Liu, M.; Zhou, Y.-J.; Xu, Y.; Hua, H.-M.; Lin, H.-W. New Bromopyrrole Alkaloids from the Marine Sponge *Agelas* sp. *Tetrahedron* **2017**, *73*, 2786–2792, doi:10.1016/j.tet.2017.03.078.
178. Kwon, O.-S.; Kim, D.; Kim, H.; Lee, Y.-J.; Lee, H.-S.; Sim, C.J.; Oh, D.-C.; Lee, S.K.; Oh, K.-B.; Shin, J. Bromopyrrole Alkaloids from the Sponge *Agelas kosrae*. *Mar Drugs* **2018**, *16*, E513, doi:10.3390/md16120513.
179. Kubota, T.; Nakamura, K.; Kurimoto, S.-I.; Sakai, K.; Fromont, J.; Gonoi, T.; Kobayashi, J. Zamamidine D, a Manzamine Alkaloid from an Okinawan *Amphimedon* sp. Marine Sponge. *J Nat Prod* **2017**, *80*, 1196–1199, doi:10.1021/acs.jnatprod.6b01110.
180. Gros, E.; Martin, M.-T.; Sorres, J.; Moriou, C.; Vacelet, J.; Frederich, M.; Aknin, M.; Kashman, Y.; Gauvin-Bialecki, A.; Al-Mourabit, A. Netamines O–S, Five New Tricyclic Guanidine Alkaloids from the Madagascar Sponge *Biemna laboutei*, and Their Antimalarial Activities. *Chemistry & Biodiversity* **2015**, *12*, 1725–1733, doi:10.1002/cbdv.201400350.
181. El-Hawary, S.S.; Sayed, A.M.; Mohammed, R.; Hassan, H.M.; Rateb, M.E.; Amin, E.; Mohammed, T.A.; El-Mesery, M.; Bin Muhsinah, A.; Alsayari, A.; et al. Bioactive Brominated Oxindole Alkaloids from the Red Sea Sponge *Callyspongia siphonella*. *Mar Drugs* **2019**, *17*, E465, doi:10.3390/md17080465.
182. Kim, C.-K.; Woo, J.-K.; Lee, Y.-J.; Lee, H.-S.; Sim, C.J.; Oh, D.-C.; Oh, K.-B.; Shin, J. Callyazepin and (3R)-Methylazacyclodecane, Nitrogenous Macrocycles from a *Callyspongia* sp. *sponge*. *J. Nat. Prod.* **2016**, *79*, 1179–1183, doi:10.1021/acs.jnatprod.5b01078.
183. Suo, R.; Takada, K.; Irie, R.; Watanabe, R.; Suzuki, T.; Ise, Y.; Ohtsuka, S.; Okada, S.; Matsunaga, S. Poecillastrin H, a Chondropsin-Type Macrolide with a Conjugated Pentaene Moiety, from a *Characella* sp. Marine Sponge. *J Nat Prod* **2018**, *81*, 1295–1299, doi:10.1021/acs.jnatprod.8b00180.
184. Mokhlesi, A.; Hartmann, R.; Kurtán, T.; Weber, H.; Lin, W.; Chaidir, C.; Müller, W.E.G.; Daletos, G.; Proksch, P. New 2-Methoxy Acetylenic Acids and Pyrazole Alkaloids from the Marine Sponge *Cinachyrella* sp. *Marine Drugs* **2017**, *15*, 356, doi:10.3390/md15110356.

185. Kasmiati, K.; Yoshioka, Y.; Okamoto, T.; Ojika, M. New Crambescidin-Type Alkaloids from the Indonesian Marine Sponge *Clathria bulbotoxa*. *Mar Drugs* **2018**, *16*, E84, doi:10.3390/md16030084.
186. Vlachou, P.; Le Goff, G.; Alonso, C.; Álvarez, P.A.; Gallard, J.-F.; Fokialakis, N.; Ouazzani, J. Innovative Approach to Sustainable Marine Invertebrate Chemistry and a Scale-Up Technology for Open Marine Ecosystems. *Mar Drugs* **2018**, *16*, E152, doi:10.3390/md16050152.
187. de Souza, R.T.M.P.; Freire, V.F.; Gubiani, J.R.; Ferreira, R.O.; Trivella, D.B.B.; Moraes, F.C.; Paradas, W.C.; Salgado, L.T.; Pereira, R.C.; Amado Filho, G.M.; et al. Bromopyrrole Alkaloid Inhibitors of the Proteasome Isolated from a *Dictyonella* sp. Marine Sponge Collected at the Amazon River Mouth. *J Nat Prod* **2018**, *81*, 2296–2300, doi:10.1021/acs.jnatprod.8b00533.
188. Cruz, P.G.; Martínez Leal, J.F.; Daranas, A.H.; Pérez, M.; Cuevas, C. On the Mechanism of Action of Dragmacidins I and J, Two New Representatives of a New Class of Protein Phosphatase 1 and 2A Inhibitors. *ACS Omega* **2018**, *3*, 3760–3767, doi:10.1021/acsomega.7b01786.
189. Santos, M.F.C.; Harper, P.M.; Williams, D.E.; Mesquita, J.T.; Pinto, É.G.; da Costa-Silva, T.A.; Hajdu, E.; Ferreira, A.G.; Santos, R.A.; Murphy, P.J.; et al. Anti-Parasitic Guanidine and Pyrimidine Alkaloids from the Marine Sponge *Monanchora arbuscula*. *J. Nat. Prod.* **2015**, *78*, 1101–1112, doi:10.1021/acs.jnatprod.5b00070.
190. Tabakmakher, K.M.; Makarieva, T.N.; Shubina, L.K.; Denisenko, V.A.; Popov, R.S.; Kuzmich, A.S.; Lee, H.-S.; Lee, Y.-J.; Stonik, V.A. Monanchoxymycalins A and B, New Hybrid Pentacyclic Guanidine Alkaloids from the Far-Eastern Marine Sponge *Monanchora pulchra*. *Nat Prod Commun* **2016**, *11*, 1817–1820.
191. Tabakmakher, K.M.; Makarieva, T.N.; Denisenko, V.A.; Popov, R.S.; Kuzmich, A.S.; Shubina, L.K.; Lee, H.-S.; Lee, Y.-J.; Fedorov, S.N. Normonanchocidins G and H, New Pentacyclic Guanidine Alkaloids from the Far-Eastern Marine Sponge *Monanchora pulchra*. *Natural Product Communications* **2017**, *12*, 1029–1032, doi:10.1177/1934578X1701200707.
192. Shubina, L.K.; Makarieva, T.N.; von Amsberg, G.; Denisenko, V.A.; Popov, R.S.; Dyshlovoy, S.A. Monanchoxymycalin C with Anticancer Properties, New Analogue of Crambescidin 800 from the Marine Sponge *Monanchora pulchra*. *Natural Product Research* **2019**, *33*, 1415–1422, doi:10.1080/14786419.2017.1419231.
193. El-Demerdash, A.; Moriou, C.; Martin, M.-T.; Rodrigues-Stien, A. de S.; Petek, S.; Demoy-Schneider, M.; Hall, K.; Hooper, J.N.A.; Debitus, C.; Al-Mourabit, A. Cytotoxic Guanidine Alkaloids from a French Polynesian *Monanchora* n. sp. Sponge. *J. Nat. Prod.* **2016**, *79*, 1929–1937, doi:10.1021/acs.jnatprod.6b00168.
194. Campos, P.-E.; Wolfender, J.-L.; Queiroz, E.F.; Marcourt, L.; Al-Mourabit, A.; Frederich, M.; Bordignon, A.; De Voogd, N.; Illien, B.; Gauvin-Bialecki, A. Unguiculin A and Ptilomycalins E–H, Antimalarial Guanidine Alkaloids from the Marine Sponge *Monanchora unguiculata*. *J. Nat. Prod.* **2017**, *80*, 1404–1410, doi:10.1021/acs.jnatprod.6b01079.
195. Hong, S.W.; Singh, A.J.; Patel, V.; Russell, E.R.; Field, J.J.; Miller, J.H.; Northcote, P.T. Peloruside E (22-Norpeloruside A), a Pelorusane Macrolide from the New Zealand Marine Sponge *Mycale hentscheli*, Retains Microtubule-Stabilizing Properties. *J Nat Prod* **2018**, *81*, 2125–2128, doi:10.1021/acs.jnatprod.8b00557.

196. Xue, D.-Q.; Liu, H.-L.; Chen, S.-H.; Mollo, E.; Gavagnin, M.; Li, J.; Li, X.-W.; Guo, Y.-W. 5-Alkylpyrrole-2-Carboxaldehyde Derivatives from the Chinese Sponge *Mycale lissochela* and Their PTP1B Inhibitory Activities. *Chinese Chemical Letters* **2017**, *28*, 1190–1193, doi:10.1016/j.cclet.2017.03.040.
197. Liang, Z.; Sulzmaier, F.J.; Yoshida, W.Y.; Kelly, M.; Ramos, J.W.; Williams, P.G. Neopetrocyclamines A and B, Polycyclic Diamine Alkaloids from the Sponge *Neopetrosia* cf *exigua*. *J. Nat. Prod.* **2015**, *78*, 543–547, doi:10.1021/np500759r.
198. Irie, R.; Hitora, Y.; Ise, Y.; Okada, S.; Takada, K.; Matsunaga, S. Poecillastrin E, F, and G, Cytotoxic Chondropsin-Type Macrolides from a Marine Sponge *Poecillastra* sp. *Tetrahedron* **2018**, *74*, 1430–1434, doi:10.1016/j.tet.2018.01.037.
199. Jamison, M.T.; Molinski, T.F. Antipodal Crambescin A2 Homologues from the Marine Sponge *Pseudaxinella reticulata*. Antifungal Structure–Activity Relationships. *J. Nat. Prod.* **2015**, *78*, 557–561, doi:10.1021/np501052a.
200. Miguel-Gordo, M.; Gegunde, S.; Calabro, K.; Jennings, L.K.; Alfonso, A.; Genta-Jouve, G.; Vacelet, J.; Botana, L.M.; Thomas, O.P. Bromotryptamine and Bromotyramine Derivatives from the Tropical Southwestern Pacific Sponge *Narrabeena nigra*. *Marine Drugs* **2019**, *17*, 319, doi:10.3390/md17060319.
201. Ebada, S.S.; Linh, M.H.; Longeon, A.; de Voogd, N.J.; Durieu, E.; Meijer, L.; Bourguet-Kondracki, M.-L.; Singab, A.N.B.; Müller, W.E.G.; Proksch, P. Dispacamide E and Other Bioactive Bromopyrrole Alkaloids from Two Indonesian Marine Sponges of the Genus *Stylissa*. *Natural Product Research* **2015**, *29*, 231–238, doi:10.1080/14786419.2014.947496.
202. Parra, L.L.L.; Bertonha, A.F.; Severo, I.R.M.; Aguiar, A.C.C.; de Souza, G.E.; Oliva, G.; Guido, R.V.C.; Grazzia, N.; Costa, T.R.; Miguel, D.C.; et al. Isolation, Derivative Synthesis, and Structure-Activity Relationships of Antiparasitic Bromopyrrole Alkaloids from the Marine Sponge *Tedania brasiliensis*. *J Nat Prod* **2018**, *81*, 188–202, doi:10.1021/acs.jnatprod.7b00876.
203. Liu, H.-B.; Lauro, G.; O'Connor, R.D.; Lohith, K.; Kelly, M.; Colin, P.; Bifulco, G.; Bewley, C.A. Tulongicin, an Antibacterial Tri-Indole Alkaloid from a Deep-Water *Topsentia* sp. Sponge. *J Nat Prod* **2017**, *80*, 2556–2560, doi:10.1021/acs.jnatprod.7b00452.
204. Kalinski, J.-C.J.; Waterworth, S.C.; Noundou, X.S.; Jiwaji, M.; Parker-Nance, S.; Krause, R.W.M.; McPhail, K.L.; Dorrington, R.A. Molecular Networking Reveals Two Distinct Chemotypes in Pyrroloiminoquinone-Producing *Tsitsikamma favus* Sponges. *Mar Drugs* **2019**, *17*, E60, doi:10.3390/md17010060.
205. Dung, D.T.; Hang, D.T.T.; Yen, P.H.; Quang, T.H.; Nhiem, N.X.; Tai, B.H.; Minh, C.V.; Kim, Y.-C.; Kim, D.C.; Oh, H.; et al. Macrocyclic Bis-Quinolizidine Alkaloids from *Xestospongia muta*. *Nat Prod Res* **2019**, *33*, 400–406, doi:10.1080/14786419.2018.1455043.
206. Arai, M.; Kamiya, K.; Shin, D.; Matsumoto, H.; Hisa, T.; Setiawan, A.; Kotoku, N.; Kobayashi, M. *N*-Methylniphatyne A, a New 3-Alkylpyridine Alkaloid as an Inhibitor of the Cancer Cells Adapted to Nutrient Starvation, from an Indonesian Marine Sponge of *Xestospongia* sp. *Chem. Pharm. Bull.* **2016**, *64*, 766–771, doi:10.1248/cpb.c16-00118.

207. Kusama, T.; Tanaka, N.; Takahashi-Nakaguchi, A.; Gonoi, T.; Fromont, J.; Kobayashi, J. Bromopyrrole Alkaloids from a Marine Sponge *Agelas* sp. *Chemical and Pharmaceutical Bulletin* **2014**, *62*, 499–503, doi:10.1248/cpb.c14-00077.
208. Abou-Hussein, D.R.; Badr, J.M.; Youssef, D.T.A. Dragmacidoside: A New Nucleoside from the Red Sea Sponge *Dragmacidon coccinea*. *Natural Product Research* **2014**, *28*, 1134–1141, doi:10.1080/14786419.2014.915828.
209. Grkovic, T.; Blees, J.S.; Bayer, M.M.; Colburn, N.H.; Thomas, C.L.; Henrich, C.J.; Peach, M.L.; McMahon, J.B.; Schmid, T.; Gustafson, K.R. Tricyclic Guanidine Alkaloids from the Marine Sponge *Acanthella cavernosa* That Stabilize the Tumor Suppressor PDCD4. *Marine Drugs* **2014**, *12*, 4593–4601, doi:10.3390/md12084593.
210. Pham, C.-D.; Hartmann, R.; Böhler, P.; Stork, B.; Wesselborg, S.; Lin, W.; Lai, D.; Proksch, P. Callyspongiolide, a Cytotoxic Macrolide from the Marine Sponge *Callyspongia* sp. *Org. Lett.* **2014**, *16*, 266–269, doi:10.1021/ol403241v.
211. El-Desoky, A.H.; Kato, H.; Eguchi, K.; Kawabata, T.; Fujiwara, Y.; Losung, F.; Mangindaan, R.E.P.; de Voogd, N.J.; Takeya, M.; Yokosawa, H.; et al. Acantholactam and Pre-Neo-Kauluamine, Manzamine-Related Alkaloids from the Indonesian Marine Sponge *Acanthostrongylophora ingens*. *J. Nat. Prod.* **2014**, *77*, 1536–1540, doi:10.1021/np500290a.
212. Gros, E.; Al-Mourabit, A.; Martin, M.-T.; Sorres, J.; Vacelet, J.; Frederich, M.; Aknin, M.; Kashman, Y.; Gauvin-Bialecki, A. Netamines H–N, Tricyclic Alkaloids from the Marine Sponge *Biemna laboutei* and Their Antimalarial Activity. *J. Nat. Prod.* **2014**, *77*, 818–823, doi:10.1021/np4009283.
213. Tran, T.D.; Pham, N.B.; Quinn, R.J. Structure Determination of Pentacyclic Pyridoacridine Alkaloids from the Australian Marine Organisms *Ancorina geodides* and Cnemidocarpa Stolonifera. *European Journal of Organic Chemistry* **2014**, *2014*, 4805–4816, doi:10.1002/ejoc.201402372.
214. Sun, J.-Z.; Jiang, C.-S.; Chen, X.-Q.; Chen, K.-S.; Zhen, X.-C.; van Soest, R.W.M.; Guo, Y.-W. Topsendines A–F, New 3-Alkylpyridine Alkaloids from a Hainan Sponge *Topsentia* sp. *Tetrahedron* **2014**, *70*, 3166–3171, doi:10.1016/j.tet.2014.03.051.
215. Makarieva, T.N.; Ogurtsova, E.K.; Denisenko, V.A.; Dmitrenok, P.S.; Tabakmakher, K.M.; Guzii, A.G.; Pislyagin, E.A.; Es'kov, A.A.; Kozhemyako, V.B.; Aminin, D.L.; et al. Urupocidin A: A New, Inducing INOS Expression Bicyclic Guanidine Alkaloid from the Marine Sponge *Monanchora pulchra*. *Org. Lett.* **2014**, *16*, 4292–4295, doi:10.1021/ol502013f.
216. Liang, L.-F.; Wang, T.; Cai, Y.-S.; He, W.-F.; Sun, P.; Li, Y.-F.; Huang, Q.; Taglialatela-Scafati, O.; Wang, H.-Y.; Guo, Y.-W. Brominated Polyunsaturated Lipids from the Chinese Sponge *Xestospongia testudinaria* as a New Class of Pancreatic Lipase Inhibitors. *European Journal of Medicinal Chemistry* **2014**, *79*, 290–297, doi:10.1016/j.ejmech.2014.04.003.
217. Ayyad, S.-E.N.; Angawy, R.; Alarif; Saqer, E.A.; Badria, F.A. Cytotoxic Polyacetylenes from the Red Sea Sponge *Siphonochalina siphonella*. *Zeitschrift für Naturforschung C* **2014**, *69*, 117–123, doi:10.5560/znc.2013-0088.

218. Machida, K.; Abe, T.; Arai, D.; Okamoto, M.; Shimizu, I.; de Voogd, N.J.; Fusetani, N.; Nakao, Y. Cinanthrenol A, an Estrogenic Steroid Containing Phenanthrene Nucleus, from a Marine Sponge *Cinachyrella* sp. *Org. Lett.* **2014**, *16*, 1539–1541, doi:10.1021/ol5000023.
219. Farokhi, F.; Grellier, P.; Clément, M.; Roussakis, C.; Loiseau, P.M.; Genin-Seward, E.; Kornprobst, J.-M.; Barnathan, G.; Wielgosz-Collin, G. Antimalarial Activity of Axidjiferosides, New β-Galactosylceramides from the African Sponge *Axinyssa djiferi*. *Marine Drugs* **2013**, *11*, 1304–1315, doi:10.3390/md11041304.
220. Murayama, S.; Imae, Y.; Takada, K.; Kikuchi, J.; Nakao, Y.; van Soest, R.W.M.; Okada, S.; Matsunaga, S. Shishicrellastatins, Inhibitors of Cathepsin B, from the Marine Sponge *Crella* (*Yvesia*) *spinulata*. *Bioorganic & Medicinal Chemistry* **2011**, *19*, 6594–6598, doi:10.1016/j.bmc.2011.06.052.
221. Chianese, G.; Fattorusso, E.; Taglialatela-Scafati, O.; Bavestrello, G.; Calcinai, B.; Dien, H.A.; Ligresti, A.; Di Marzo, V. Desulfohaplosamate, a New Phosphate-Containing Steroid from *Dasychalina* sp., Is a Selective Cannabinoid CB2 Receptor Ligand. *Steroids* **2011**, *76*, 998–1002, doi:10.1016/j.steroids.2011.03.013.
222. Wang, W.; Mun, B.; Lee, Y.; Venkat Reddy, M.; Park, Y.; Lee, J.; Kim, H.; Hahn, D.; Chin, J.; Ekins, M.; et al. Bioactive Sesterterpenoids from a Korean Sponge *Monanchora* sp. *J. Nat. Prod.* **2013**, *76*, 170–177, doi:10.1021/np300573m.
223. Cutignano, A.; Nuzzo, G.; D'Angelo, D.; Borbone, E.; Fusco, A.; Fontana, A. Mycalol: A Natural Lipid with Promising Cytotoxic Properties against Human Anaplastic Thyroid Carcinoma Cells. *Angewandte Chemie International Edition* **2013**, *52*, 9256–9260, doi:10.1002/anie.201303039.
224. Farokhi, F.; Wielgosz-Collin, G.; Robic, A.; Debitus, C.; Malleter, M.; Roussakis, C.; Kornprobst, J.-M.; Barnathan, G. Antiproliferative Activity against Human Non-Small Cell Lung Cancer of Two O-Alkyl-Diglycosylglycerols from the Marine Sponges *Myrmekioderma dendyi* and *Trikentrion laeve*. *European Journal of Medicinal Chemistry* **2012**, *49*, 406–410, doi:10.1016/j.ejmech.2012.01.029.
225. Ohta, S.; Ogawa, T.; Ohta, E.; Ikeuchi, T.; Kamemura, K.; Ikegami, S. Petroacetylene, a New Polyacetylene from the Marine Sponge *Petrosiasolida* That Inhibits Blastulation of Starfish Embryos. *Natural Product Research* **2013**, *27*, 1842–1847, doi:10.1080/14786419.2012.763128.
226. Hitora, Y.; Takada, K.; Okada, S.; Ise, Y.; Matsunaga, S. (-)-Duryne and Its Homologues, Cytotoxic Acetylenes from a Marine Sponge *Petrosia* sp. *J Nat Prod* **2011**, *74*, 1262–1267, doi:10.1021/np200271n.
227. Hitora, Y.; Takada, K.; Okada, S.; Matsunaga, S. Miyakosynes A–F, Cytotoxic Methyl Branched Acetylenes from a Marine Sponge *Petrosia* sp. *Tetrahedron* **2011**, *67*, 4530–4534, doi:10.1016/j.tet.2011.04.085.
228. Lee, Y.-J.; Yoo, S.-J.; Kang, J.S.; Yun, J.; Shin, H.J.; Lee, J.S.; Lee, H.-S. Cytotoxic Petrosiacetylenes from the Marine Sponge *Petrosia* sp. *Lipids* **2013**, *48*, 87–91, doi:10.1007/s11745-012-3727-5.
229. Mejia, E.J.; Magranet, L.B.; De Voogd, N.J.; TenDyke, K.; Qiu, D.; Shen, Y.Y.; Zhou, Z.; Crews, P. Structures and Cytotoxic Evaluation of New and Known Acyclic Ene-Ynes from an American Samoa *Petrosia* sp. Sponge. *J. Nat. Prod.* **2013**, *76*, 425–432, doi:10.1021/np3008446.

230. Horikawa, K.; Yagyu, T.; Yoshioka, Y.; Fujiwara, T.; Kanamoto, A.; Okamoto, T.; Ojika, M. Petrosiols A–E, Neurotrophic Diyne Tetraols Isolated from the Okinawan Sponge *Petrosia strongylata*. *Tetrahedron* **2013**, *69*, 101–106, doi:10.1016/j.tet.2012.10.063.
231. Kim, H.; Chin, J.; Choi, H.; Baek, K.; Lee, T.-G.; Park, S.E.; Wang, W.; Hahn, D.; Yang, I.; Lee, J.; et al. Phosphoiodyns A and B, Unique Phosphorus-Containing Iodinated Polyacetylenes from a Korean Sponge *Placospongia* sp. *Org. Lett.* **2013**, *15*, 100–103, doi:10.1021/ol3031318.
232. Li, J.; Zhu, H.; Ren, J.; Deng, Z.; Voogd, N.J. de; Proksch, P.; Lin, W. Globostelletins J–S, Isomalabaricanes with Unusual Cyclopentane Sidechains from the Marine Sponge *Rhabdastrella globostellata*. *Tetrahedron* **2012**, *68*, 559–565, doi:10.1016/j.tet.2011.11.001.
233. Jang, K.H.; Lee, Y.; Sim, C.J.; Oh, K.-B.; Shin, J. Bioactive Lipids from the Sponge *Spirastrella abata*. *Bioorganic & Medicinal Chemistry Letters* **2012**, *22*, 1078–1081, doi:10.1016/j.bmcl.2011.11.105.
234. Morinaka, B.I.; Molinski, T.F. Mollenyne A, a Long-Chain Chlorodibromohydrin Amide from the Sponge *Spirastrella mollis*. *Org. Lett.* **2011**, *13*, 6338–6341, doi:10.1021/ol2024778.
235. Hwang, B.S.; Lee, K.; Yang, C.; Jeong, E.J.; Rho, J.-R. Characterization and Anti-Inflammatory Effects of Iodinated Acetylenic Acids Isolated from the Marine Sponges *Suberites mammilaris* and *Suberites japonicus*. *J. Nat. Prod.* **2013**, *76*, 2355–2359, doi:10.1021/np400793r.
236. Costantino, V.; Fattorusso, E.; Mangoni, A.; Teta, R.; Panza, E.; Ianaro, A. Terpioside B, a Difucosyl GSL from the Marine Sponge *Terpios* sp. Is a Potent Inhibitor of NO Release. *Bioorg Med Chem* **2010**, *18*, 5310–5315, doi:10.1016/j.bmc.2010.05.048.
237. Aoki, N.; Yamamoto, K.; Ogawa, T.; Ohta, E.; Ikeuchi, T.; Kamemura, K.; Ikegami, S.; Ohta, S. Bromotheoynic Acid, a Brominated Acetylenic Acid from the Marine Sponge *Theonella swinhoei*. *Natural Product Research* **2013**, *27*, 117–122, doi:10.1080/14786419.2012.660636.
238. Dai, J.; Sorribas, A.; Yoshida, W.Y.; Kelly, M.; Williams, P.G. Topsentinols, 24-Isopropyl Steroids from the Marine Sponge *Topsentia* sp. *J. Nat. Prod.* **2010**, *73*, 1597–1600, doi:10.1021/np100374b.
239. Ankisetty, S.; Slattery, M. Antibacterial Secondary Metabolites from the Cave Sponge *Xestospongia* sp. *Marine Drugs* **2012**, *10*, 1037–1043, doi:10.3390/md10051037.
240. Zhou, X.; Lu, Y.; Lin, X.; Yang, B.; Yang, X.; Liu, Y. Brominated Aliphatic Hydrocarbons and Sterols from the Sponge *Xestospongia testudinaria* with Their Bioactivities. *Chemistry and Physics of Lipids* **2011**, *164*, 703–706, doi:10.1016/j.chemphyslip.2011.08.002.
241. Akiyama, T.; Takada, K.; Oikawa, T.; Matsuura, N.; Ise, Y.; Okada, S.; Matsunaga, S. Stimulators of Adipogenesis from the Marine Sponge *Xestospongia testudinaria*. *Tetrahedron* **2013**, *69*, 6560–6564, doi:10.1016/j.tet.2013.06.007.
242. Kim, H.; Kim, K.-J.; Yeon, J.-T.; Kim, S.H.; Won, D.H.; Choi, H.; Nam, S.-J.; Son, Y.-J.; Kang, H. Placotylene A, an Inhibitor of the Receptor Activator of Nuclear Factor-KB Ligand-Induced Osteoclast Differentiation, from a Korean Sponge *Placospongia* sp. *Marine Drugs* **2014**, *12*, 2054–2065, doi:10.3390/md12042054.

243. Chiu, C.-W.; Su, H.-J.; Lu, M.-C.; Wang, W.-H.; Sheu, J.-H.; Su, J.-H. Cytotoxic Polyacetylenes from a Formosan Marine Sponge *Callyspongia* sp. *BCSJ* **2014**, *87*, 1231–1234, doi:10.1246/bcsj.20140188.
244. Juan, Y.-S.; Lee, C.-C.; Tsao, C.-W.; Lu, M.-C.; El-Shazly, M.; Shih, H.-C.; Chen, Y.-C.; Wu, Y.-C.; Su, J.-H. Structure Elucidation and Cytotoxic Evaluation of New Polyacetylenes from a Marine Sponge *Petrosia* sp. *International Journal of Molecular Sciences* **2014**, *15*, 16511–16521, doi:10.3390/ijms150916511.
245. Abdel-Lateff, A.; Alarif, W.M.; Asfour, H.Z.; Ayyad, S.-E.N.; Khedr, A.; Badria, F.A.; Al-Lihaibi, S.S. Cytotoxic Effects of Three New Metabolites from Red Sea Marine Sponge, *Petrosia* sp. *Environ Toxicol Pharmacol* **2014**, *37*, 928–935, doi:10.1016/j.etap.2014.03.005.
246. Carballeira, N.M.; Montano, N.; Amador, L.A.; Rodríguez, A.D.; Golovko, M.Y.; Golovko, S.A.; Reguera, R.M.; Álvarez-Velilla, R.; Balaña-Fouce, R. Novel Very Long-Chain α-Methoxylated Δ5,9 Fatty Acids from the Sponge *Asteropus niger* Are Effective Inhibitors of Topoisomerases IB. *Lipids* **2016**, *51*, 245–256, doi:10.1007/s11745-015-4114-9.
247. Calabro, K.; Chalén, B.E.; Genta-Jouve, G.; Jaramillo, K.B.; Domínguez, C.; de la Cruz, M.; Cautain, B.; Reyes, F.; Thomas, O.P.; Rodríguez, J. Callyspongidic Acids: Amphiphilic Diacids from the Tropical Eastern Pacific Sponge *Callyspongia* cf. *californica*. *J Nat Prod* **2018**, *81*, 2301–2305, doi:10.1021/acs.jnatprod.8b00683.
248. Santalova, E.A.; Denisenko, V.A.; Dmitrenok, P.S.; Ha, D.T.; Hung, N.A.; Drozdov, A.L.; Malyarenko, O.S.; Thuy, T.T.T.; Quan, P.M.; Long, P.Q. Occurrence of Melibiose-Containing Glycosphingolipids in a Sample of a Sponge-Coral Association (*Desmapsamma anchorata*/*Carijoa riisei*). *Chem Biodivers* **2019**, *16*, e1800401, doi:10.1002/cbdv.201800401.
249. Woo, J.-K.; Yun, J.-H.; Ahn, S.; Sim, C.J.; Noh, M.; Oh, D.-C.; Oh, K.-B.; Shin, J. Dictyoneolone, a B/C Ring Juncture-Defused Steroid from a *Dictyonella* sp. Sponge. *Tetrahedron Letters* **2018**, *59*, 2021–2024, doi:10.1016/j.tetlet.2018.04.026.
250. Tan, K.C.; Wakimoto, T.; Abe, I. Sulfoureido Lipopeptides from the Marine Sponge *Discodermia kiiensis*. *J. Nat. Prod.* **2016**, *79*, 2418–2422, doi:10.1021/acs.jnatprod.6b00586.
251. Guzii, A.G.; Makarieva, T.N.; Denisenko, V.A.; Dmitrenok, P.S.; Kuzmich, A.S.; Dyshlovoy, S.A.; von Amsberg, G.; Krasokhin, V.B.; Stonik, V.A. Melonoside A: An ω-Glycosylated Fatty Acid Amide from the Far Eastern Marine Sponge *Melonanchora kobjakovae*. *Org. Lett.* **2016**, *18*, 3478–3481, doi:10.1021/acs.orglett.6b01678.
252. Guzii, A.G.; Makarieva, T.N.; Denisenko, V.A.; Dmitrenok, P.S.; Popov, R.S.; Kuzmich, A.S.; Fedorov, S.N.; Krasokhin, V.B.; Kim, N.Y.; Stonik, V.A. Melonoside B and Melonosins A and B, Lipids Containing Multifunctionalized ω-Hydroxy Fatty Acid Amides from the Far Eastern Marine Sponge *Melonanchora kobjakovae*. *J Nat Prod* **2018**, *81*, 2763–2767, doi:10.1021/acs.jnatprod.8b00785.

253. Raslan, A.E.; Radwan, M.M.; Ahmed, S.A.; Nafady, A.M.; Zaki, M.A.; Wanas, A.S.; Abou-Karam, M.; Shier, T.W.; Hassanean, H.A.; ElSohly, M.A. Monanchoramides A–D, Ceramides from the Marine Sponge *Monanchora clathrata* with Cytotoxic Activity. *Phytochemistry Letters* **2018**, *23*, 83–89, doi:10.1016/j.phytol.2017.11.015.
254. Sala, S.; Fromont, J.; Gomez, O.; Vuong, D.; Lacey, E.; Flematti, G.R. Albanitriles A-G: Antiprotozoal Polyacetylene Nitriles from a *Mycale* Marine Sponge. *J Nat Prod* **2019**, *82*, 3450–3455, doi:10.1021/acs.jnatprod.9b00840.
255. Einarsdottir, E.; Liu, H.-B.; Freysdottir, J.; Gotfredsen, C.H.; Omarsdottir, S. Immunomodulatory N-Acyl Dopamine Glycosides from the Icelandic Marine Sponge *Myxilla incrustans* Collected at a Hydrothermal Vent Site. *Planta Med* **2016**, *82*, 903–909, doi:10.1055/s-0042-105877.
256. Eltahawy, N.A.; Ibrahim, A.K.; Gomaa, M.S.; Zaitone, S.A.; Radwan, M.M.; Hassanean, H.A.; ElSohly, M.A.; Ahmed, S.A. Anxiolytic and Anticonvulsant Activity Followed by Molecular Docking Study of Ceramides from the Red Sea Sponge *Negombata* sp. *Med Chem Res* **2019**, *28*, 1818–1827, doi:10.1007/s00044-019-02408-3.
257. Takanashi, E.; Takada, K.; Hashimoto, M.; Itoh, Y.; Ise, Y.; Ohtsuka, S.; Okada, S.; Matsunaga, S. Cytotoxic Linear Acetylenes from a Marine Sponge *Pleroma* sp. *Tetrahedron* **2015**, *71*, 9564–9570, doi:10.1016/j.tet.2015.10.062.
258. Calabro, K.; Kalahroodi, E.L.; Rodrigues, D.; Díaz, C.; Cruz, M. de la; Cautain, B.; Laville, R.; Reyes, F.; Pérez, T.; Soussi, B.; et al. Poecillastrosides, Steroidal Saponins from the Mediterranean Deep-Sea Sponge *Poecillastra compressa* (Bowerbank, 1866). *Marine Drugs* **2017**, *15*, 199, doi:10.3390/md15070199.
259. Eltamany, E.E.; Ibrahim, A.K.; Radwan, M.M.; ElSohly, M.A.; Hassanean, H.A.; Ahmed, S.A. Cytotoxic Ceramides from the Red Sea Sponge *Spheciospongia vagabunda*. *Medicinal Chemistry Research* **2015**, *9*, 3467–3473, doi:10.1007/s00044-015-1394-9.
260. Lin, K.; Yang, P.; Yang, H.; Liu, A.-H.; Yao, L.-G.; Guo, Y.-W.; Mao, S.-C. Lysophospholipids from the Guangxi Sponge *Spirastrella purpurea*. *Lipids* **2015**, *50*, 697–703, doi:10.1007/s11745-015-4028-6.
261. Peddie, V.; Takada, K.; Okuda, S.; Ise, Y.; Morii, Y.; Yamawaki, N.; Takatani, T.; Arakawa, O.; Okada, S.; Matsunaga, S. Cytotoxic Glycosylated Fatty Acid Amides from a *Stelletta* sp. Marine Sponge. *J. Nat. Prod.* **2015**, *78*, 2808–2813, doi:10.1021/acs.jnatprod.5b00795.
262. Abou-Hussein, D.R.; Youssef, D.T.A. Mirabolides A and B; New Cytotoxic Glycerides from the Red Sea Sponge *Theonella mirabilis*. *Marine Drugs* **2016**, *14*, 155, doi:10.3390/md14080155.
263. Takada, K.; Imae, Y.; Ise, Y.; Ohtsuka, S.; Ito, A.; Okada, S.; Yoshida, M.; Matsunaga, S. Yakushinamides, Polyoxygenated Fatty Acid Amides That Inhibit HDACs and SIRTs, from the Marine Sponge *Theonella swinhoei*. *J. Nat. Prod.* **2016**, *79*, 2384–2390, doi:10.1021/acs.jnatprod.6b00588.
264. Ayyad, S.-E.N.; Katoua, D.F.; Alarif, W.M.; Sobahi, T.R.; Aly, M.M.; Shaala, L.A.; Ghandourah, M.A. Two New Polyacetylene Derivatives from the Red Sea Sponge *Xestospongia* sp. *Zeitschrift für Naturforschung C* **2015**, *70*, 297–303, doi:10.1515/znc-2015-5015.

265. He, W.-F.; Liang, L.-F.; Cai, Y.-S.; Gao, L.-X.; Li, Y.-F.; Li, J.; Liu, H.-L.; Guo, Y.-W. Brominated Polyunsaturated Lipids with Protein Tyrosine Phosphatase-1B Inhibitory Activity from Chinese Marine Sponge *Xestospongia testudinaria*. *Journal of Asian Natural Products Research* **2015**, *17*, 861–866, doi:10.1080/10286020.2015.1026334.
266. Yang, M.; Liang, L.-F.; Yao, L.-G.; Liu, H.-L.; Guo, Y.-W. A New Brominated Polyacetylene from Chinese Marine Sponge *Xestospongia testudinaria*. *Journal of Asian natural products research* **2019**, *21*, 573–578.
267. Song, J.; Jeon, J.-E.; Won, T.H.; Sim, C.J.; Oh, D.-C.; Oh, K.-B.; Shin, J. New Cyclic Cystine Bridged Peptides from the Sponge *Suberites waedoensis*. *Mar Drugs* **2014**, *12*, 2760–2770, doi:10.3390/md12052760.
268. Tan, K.C.; Wakimoto, T.; Abe, I. Lipodiscamides A–C, New Cytotoxic Lipopeptides from *Discodermia kiiensis*. *Org. Lett.* **2014**, *16*, 3256–3259, doi:10.1021/ol501271v.
269. Davis, R.A.; Duffy, S.; Avery, V.M.; Camp, D.; Hooper, J.N.A.; Quinn, R.J. (+)-7-Bromotrypargine: An Antimalarial β-Carboline from the Australian Marine Sponge *Ancorina* sp. *Tetrahedron Letters* **2010**, *51*, 583–585, doi:10.1016/j.tetlet.2009.11.055.
270. Ibrahim, S.R.M.; Min, C.C.; Teuscher, F.; Ebel, R.; Kakoschke, C.; Lin, W.; Wray, V.; Edrada-Ebel, R.; Proksch, P. Callyaerins A–F and H, New Cytotoxic Cyclic Peptides from the Indonesian Marine Sponge *Callyspongia aerizusa*. *Bioorganic & Medicinal Chemistry* **2010**, *18*, 4947–4956, doi:10.1016/j.bmc.2010.06.012.
271. Ueoka, R.; Ise, Y.; Ohtsuka, S.; Okada, S.; Yamori, T.; Matsunaga, S. Yaku'amides A and B, Cytotoxic Linear Peptides Rich in Dehydroamino Acids from the Marine Sponge *Ceratopsion* sp. *J. Am. Chem. Soc.* **2010**, *132*, 17692–17694, doi:10.1021/ja109275z.
272. Makarieva, T.N.; Tabakmaher, K.M.; Guzii, A.G.; Denisenko, V.A.; Dmitrenok, P.S.; Shubina, L.K.; Kuzmich, A.S.; Lee, H.-S.; Stonik, V.A. Monanchocidins B–E: Polycyclic Guanidine Alkaloids with Potent Antileukemic Activities from the Sponge *Monanchora pulchra*. *J. Nat. Prod.* **2011**, *74*, 1952–1958, doi:10.1021/np200452m.
273. Woo, J.-K.; Jeon, J.; Kim, C.-K.; Sim, C.J.; Oh, D.-C.; Oh, K.-B.; Shin, J. Gombamide A, a Cyclic Thiopeptide from the Sponge *Clathria gombawuiensis*. *J. Nat. Prod.* **2013**, *76*, 1380–1383, doi:10.1021/np4003367.
274. Kimura, M.; Wakimoto, T.; Egami, Y.; Tan, K.C.; Ise, Y.; Abe, I. Calyxamides A and B, Cytotoxic Cyclic Peptides from the Marine Sponge *Discodermia calyx*. *J. Nat. Prod.* **2012**, *75*, 290–294, doi:10.1021/np2009187.
275. Prasad, P.; Aalbersberg, W.; Feussner, K.-D.; Van Wagoner, R.M. Papuamides E and F, Cytotoxic Depsipeptides from the Marine Sponge *Melophlus* sp. *Tetrahedron* **2011**, *67*, 8529–8531, doi:10.1016/j.tet.2011.08.100.
276. Tran, T.D.; Pham, N.B.; Fechner, G.; Zencak, D.; Vu, H.T.; Hooper, J.N.A.; Quinn, R.J. Cytotoxic Cyclic Depsipeptides from the Australian Marine Sponge *Neamphius huxleyi*. *J. Nat. Prod.* **2012**, *75*, 2200–2208, doi:10.1021/np3006474.

277. Yamano, Y.; Arai, M.; Kobayashi, M. Neamphamide B, New Cyclic Depsipeptide, as an Anti-Dormant Mycobacterial Substance from a Japanese Marine Sponge of *Neamphius* sp. *Bioorganic & Medicinal Chemistry Letters* **2012**, *22*, 4877–4881, doi:10.1016/j.bmcl.2012.05.071.
278. Zhang, H.-J.; Yi, Y.-H.; Yang, G.-J.; Hu, M.-Y.; Cao, G.-D.; Yang, F.; Lin, H.-W. Proline-Containing Cyclopeptides from the Marine Sponge *Phakellia* sp. *J. Nat. Prod.* **2010**, *73*, 650–655, doi:10.1021/np9008267.
279. Sorres, J.; Martin, M.-T.; Petek, S.; Levaique, H.; Cresteil, T.; Ramos, S.; Thoison, O.; Debitus, C.; Al-Mourabit, A. Pipestelides A–C: Cyclodepsipeptides from the Pacific Marine Sponge *Pipestela candelabra*. *J. Nat. Prod.* **2012**, *75*, 759–763, doi:10.1021/np200714m.
280. Cheruku, P.; Plaza, A.; Lauro, G.; Keffer, J.; Lloyd, J.R.; Bifulco, G.; Bewley, C.A. Discovery and Synthesis of Namalide Reveals a New Anabaenopeptin Scaffold and Peptidase Inhibitor. *J. Med. Chem.* **2012**, *55*, 735–742, doi:10.1021/jm201238p.
281. Lu, Z.; Van Wagoner, R.M.; Harper, M.K.; Baker, H.L.; Hooper, J.N.A.; Bewley, C.A.; Ireland, C.M. Mirabamides E-H, HIV-Inhibitory Depsipeptides from the Sponge *Stelletta clavosa*. *J Nat Prod* **2011**, *74*, 185–193, doi:10.1021/np100613p.
282. Kita, M.; Gise, B.; Kawamura, A.; Kigoshi, H. Stylissatin A, a Cyclic Peptide That Inhibits Nitric Oxide Production from the Marine Sponge *Stylissa massa*. *Tetrahedron Letters* **2013**, *54*, 6826–6828, doi:10.1016/j.tetlet.2013.10.003.
283. Arai, M.; Yamano, Y.; Fujita, M.; Setiawan, A.; Kobayashi, M. Stylissamide X, a New Proline-Rich Cyclic Octapeptide as an Inhibitor of Cell Migration, from an Indonesian Marine Sponge of *Stylissa* sp. *Bioorganic & Medicinal Chemistry Letters* **2012**, *22*, 1818–1821, doi:10.1016/j.bmcl.2011.10.023.
284. Plaza, A.; Bifulco, G.; Masullo, M.; Lloyd, J.R.; Keffer, J.L.; Colin, P.L.; Hooper, J.N.A.; Bell, L.J.; Bewley, C.A. Mutremdamide A and Koshikamides C−H, Peptide Inhibitors of HIV-1 Entry from Different *Theonella* species. *J. Org. Chem.* **2010**, *75*, 4344–4355, doi:10.1021/jo100076g.
285. Festa, C.; De Marino, S.; D'Auria, M.V.; Monti, M.C.; Bucci, M.; Vellecco, V.; Debitus, C.; Zampella, A. Anti-Inflammatory Cyclopeptides from the Marine Sponge *Theonella swinhoei*. *Tetrahedron* **2012**, *68*, 2851–2857, doi:10.1016/j.tet.2012.01.097.
286. Sinisi, A.; Calcinai, B.; Cerrano, C.; Dien, H.A.; Zampella, A.; D'Amore, C.; Renga, B.; Fiorucci, S.; Taglialatela-Scafati, O. New Tridecapeptides of the Theonellapeptolide Family from the Indonesian Sponge *Theonella swinhoei*. *Beilstein J. Org. Chem.* **2013**, *9*, 1643–1651, doi:10.3762/bjoc.9.188.
287. Tran, T.D.; Pham, N.B.; Fechner, G.A.; Hooper, J.N.A.; Quinn, R.J. Potent Cytotoxic Peptides from the Australian Marine Sponge *Pipestela candelabra*. *Marine Drugs* **2014**, *12*, 3399–3415, doi:10.3390/md12063399.
288. Zhan, K.-X.; Jiao, W.-H.; Yang, F.; Li, J.; Wang, S.-P.; Li, Y.-S.; Han, B.-N.; Lin, H.-W. Reniochalistatins A–E, Cyclic Peptides from the Marine Sponge *Reniochalina stalagmitis*. *J. Nat. Prod.* **2014**, *77*, 2678–2684, doi:10.1021/np5006778.
289. Wang, X.; Morinaka, B.I.; Molinski, T.F. Structures and Solution Conformational Dynamics of Stylissamides G and H from the Bahamian Sponge *Stylissa caribica*. *J. Nat. Prod.* **2014**, *77*, 625–630, doi:10.1021/np400891s.

290. Youssef, D.T.A.; Shaala, L.A.; Mohamed, G.A.; Badr, J.M.; Bamanie, F.H.; Ibrahim, S.R.M. Theonellamide G, a Potent Antifungal and Cytotoxic Bicyclic Glycopeptide from the Red Sea Marine Sponge *Theonella swinhoei*. *Marine Drugs* **2014**, *12*, 1911–1923, doi:10.3390/md12041911.
291. Stierhof, M.; Hansen, K.Ø.; Sharma, M.; Feussner, K.; Subko, K.; Díaz-Rullo, F.F.; Isaksson, J.; Pérez-Victoria, I.; Clarke, D.; Hansen, E.; et al. New Cytotoxic Callipeltins from the Solomon Island Marine Sponge *Asteropus* sp. *Tetrahedron* **2016**, *72*, 6929–6934, doi:10.1016/j.tet.2016.09.016.
292. Shaala, L.A.; Youssef, D.T.A.; Ibrahim, S.R.M.; Mohamed, G.A. Callyptide A, a New Cytotoxic Peptide from the Red Sea Marine Sponge *Callyspongia* species. *Natural Product Research* **2016**, *30*, 2783–2790, doi:10.1080/14786419.2016.1155577.
293. Daletos, G.; Kalscheuer, R.; Koliwer-Brandl, H.; Hartmann, R.; de Voogd, N.J.; Wray, V.; Lin, W.; Proksch, P. Callyaerins from the Marine Sponge *Callyspongia aerizusa*: Cyclic Peptides with Antitubercular Activity. *J. Nat. Prod.* **2015**, *78*, 1910–1925, doi:10.1021/acs.jnatprod.5b00266.
294. Kapojos, M.M.; Abdjul, D.B.; Yamazaki, H.; Ohshiro, T.; Rotinsulu, H.; Wewengkang, D.S.; Sumilat, D.A.; Tomoda, H.; Namikoshi, M.; Uchida, R. Callyspongiamides A and B, Sterol O-Acyltransferase Inhibitors, from the Indonesian Marine Sponge *Callyspongia* sp. *Bioorg Med Chem Lett* **2018**, *28*, 1911–1914, doi:10.1016/j.bmcl.2018.03.077.
295. Afoullouss, S.; Calabro, K.; Genta-Jouve, G.; Gegunde, S.; Alfonso, A.; Nesbitt, R.; Morrow, C.; Alonso, E.; Botana, L.M.; Allcock, A.L.; et al. Treasures from the Deep: Characellides as Anti-Inflammatory Lipoglycotripeptides from the Sponge *Characella pachastrelloides*. *Org Lett* **2019**, *21*, 246–251, doi:10.1021/acs.orglett.8b03684.
296. Mokhlesi, A.; Stuhldreier, F.; Wex, K.W.; Berscheid, A.; Hartmann, R.; Rehberg, N.; Sureechatchaiyan, P.; Chaidir, C.; Kassack, M.U.; Kalscheuer, R.; et al. Cyclic Cystine-Bridged Peptides from the Marine Sponge *Clathria basilana* Induce Apoptosis in Tumor Cells and Depolarize the Bacterial Cytoplasmic Membrane. *J. Nat. Prod.* **2017**, *80*, 2941–2952, doi:10.1021/acs.jnatprod.7b00477.
297. Urda, C.; Pérez, M.; Rodríguez, J.; Jiménez, C.; Cuevas, C.; Fernández, R. Pembamide, a N-Methylated Linear Peptide from a Sponge *Cribrochalina* sp. *Tetrahedron Letters* **2016**, *57*, 3239–3242, doi:10.1016/j.tetlet.2016.05.054.
298. Urda, C.; Fernández, R.; Rodríguez, J.; Pérez, M.; Jiménez, C.; Cuevas, C. Daedophamide, a Cytotoxic Cyclodepsipeptide from a *Daedalopelta* sp. Sponge Collected in Indonesia. *J. Nat. Prod.* **2017**, *80*, 3054–3059, doi:10.1021/acs.jnatprod.7b00678.
299. Nakamukai, S.; Takada, K.; Furihata, K.; Ise, Y.; Okada, S.; Morii, Y.; Yamawaki, N.; Takatani, T.; Arakawa, O.; Gustafson, K.R.; et al. Stellatolide H, a Cytotoxic Peptide Lactone from a Deep-Sea Sponge *Discodermia* sp. *Tetrahedron Letters* **2018**, *59*, 2532–2536, doi:10.1016/j.tetlet.2018.05.033.
300. Hahn, D.; Kim, H.; Yang, I.; Chin, J.; Hwang, H.; Won, D.H.; Lee, B.; Nam, S.-J.; Ekins, M.; Choi, H.; et al. The Halicylindramides, Farnesoid X Receptor Antagonizing Depsipeptides from a *Petrosia* sp. Marine Sponge Collected in Korea. *J. Nat. Prod.* **2016**, *79*, 499–506, doi:10.1021/acs.jnatprod.5b00871.

301. Wu, Y.; Liu, L.; Chen, H.-F.; Jiao, W.-H.; Sun, F.; Liu, L.-Y.; Zhu, H.-R.; Wang, S.-P.; Lin, H.-W. Fuscasins A-D, Cycloheptapeptides from the Marine Sponge *Phakellia fusca*. *J Nat Prod* **2019**, *82*, 970–979, doi:10.1021/acs.jnatprod.8b01033.
302. Shin, H.J.; Rashid, M.A.; Cartner, L.K.; Bokesch, H.R.; Wilson, J.A.; McMahon, J.B.; Gustafson, K.R. Stellettapeptins A and B, HIV-Inhibitory Cyclic Depsipeptides from the Marine Sponge *Stelletta* sp. *Tetrahedron Lett* **2015**, *56*, 4215–4219, doi:10.1016/j.tetlet.2015.05.058.
303. Afifi, A.H.; El-Desoky, A.H.; Kato, H.; Mangindaan, R.E.P.; de Voogd, N.J.; Ammar, N.M.; Hifnawy, M.S.; Tsukamoto, S. Carteritins A and B, Cyclic Heptapeptides from the Marine Sponge *Stylissa carteri*. *Tetrahedron Letters* **2016**, *57*, 1285–1288, doi:10.1016/j.tetlet.2016.02.031.
304. Kwon, O.-S.; Kim, C.-K.; Byun, W.S.; Oh, J.; Lee, Y.-J.; Lee, H.-S.; Sim, C.J.; Oh, D.-C.; Lee, S.K.; Oh, K.-B.; et al. Cyclopeptides from the Sponge *Stylissa flabelliformis*. *J Nat Prod* **2018**, *81*, 1426–1434, doi:10.1021/acs.jnatprod.8b00121.
305. Sun, J.; Cheng, W.; de Voogd, N.J.; Proksch, P.; Lin, W. Stylissatins B–D, Cycloheptapeptides from the Marine Sponge *Stylissa massa*. *Tetrahedron Letters* **2016**, *57*, 4288–4292, doi:10.1016/j.tetlet.2016.08.024.
306. Fukuhara, K.; Takada, K.; Okada, S.; Matsunaga, S. Nazumazoles A–C, Cyclic Pentapeptides Dimerized through a Disulfide Bond from the Marine Sponge *Theonella swinhoei*. *Org. Lett.* **2015**, *17*, 2646–2648, doi:10.1021/acs.orglett.5b01020.
307. Fukuhara, K.; Takada, K.; Okada, S.; Matsunaga, S. Nazumazoles D–F, Cyclic Pentapeptides That Inhibit Chymotrypsin, from the Marine Sponge *Theonella swinhoei*. *J. Nat. Prod.* **2016**, *79*, 1694–1697, doi:10.1021/acs.jnatprod.6b00261.
308. Issac, M.; Aknin, M.; Gauvin-Bialecki, A.; De Voogd, N.; Ledoux, A.; Frederich, M.; Kashman, Y.; Carmeli, S. Cyclotheonellazoles A–C, Potent Protease Inhibitors from the Marine Sponge *Theonella* aff. *swinhoei*. *J. Nat. Prod.* **2017**, *80*, 1110–1116, doi:10.1021/acs.jnatprod.7b00028.
309. Oku, N.; Takada, K.; Fuller, R.W.; Wilson, J.A.; Peach, M.L.; Pannell, L.K.; McMahon, J.B.; Gustafson, K.R. Isolation, Structural Elucidation, and Absolute Stereochemistry of Enigmazole A, a Cytotoxic Phosphomacrolide from the Papua New Guinea Marine Sponge *Cinachyrella enigmatica*. *J. Am. Chem. Soc.* **2010**, *132*, 10278–10285, doi:10.1021/ja1016766.
310. Singh, A.J.; Xu, C.-X.; Xu, X.; West, L.M.; Wilmes, A.; Chan, A.; Hamel, E.; Miller, J.H.; Northcote, P.T.; Ghosh, A.K. Peloruside B, A Potent Antitumor Macrolide from the New Zealand Marine Sponge *Mycale hentscheli*: Isolation, Structure, Total Synthesis, and Bioactivity. *J. Org. Chem.* **2010**, *75*, 2–10, doi:10.1021/jo9021265.
311. Balansa, W.; Trianto, A.; de Voogd, N.J.; Tanaka, J. A New Cytotoxic Polyacetylenic Alcohol from a Sponge *Callyspongia* sp. *Natural Product Communications* **2017**, *12*, 1909 —1912, doi:10.1177/1934578X1701201224.
312. Tanokashira, N.; Kukita, S.; Kato, H.; Nehira, T.; Angkouw, E.D.; Mangindaan, R.E.P.; de Voogd, N.J.; Tsukamoto, S. Petroquinones: Trimeric and Dimeric Xestoquinone Derivatives Isolated from the Marine Sponge *Petrosia alfiani*. *Tetrahedron* **2016**, *72*, 5530–5540, doi:10.1016/j.tet.2016.07.045.

313. Lorente, A.; Gil, A.; Fernández, R.; Cuevas, C.; Albericio, F.; Álvarez, M. Phormidolides B and C, Cytotoxic Agents from the Sea: Enantioselective Synthesis of the *Macrocyclic core*. *Chemistry – A European Journal* **2015**, *21*, 150–156, doi:10.1002/chem.201404341.
314. Gabriel, A.F.; Li, Z.; Kusuda, R.; Tanaka, C.; Miyamoto, T. Six New Polyacetylenic Alcohols from the Marine Sponges Petrosia sp. and *Halichondria* sp. *Chem Pharm Bull (Tokyo)* **2015**, *63*, 469–475, doi:10.1248/cpb.c15-00203.
315. Rodríguez, J.; Jiménez, C.; Blanco, M.; Tarazona, G.; Fernández, R.; Cuevas, C. Lanesoic Acid: A Cytotoxic Zwitterion from *Theonella* sp. *Org. Lett.* **2016**, *18*, 5832–5835, doi:10.1021/acs.orglett.6b02832.
316. Saito, N.; Suwanborirux, K.; Hiramatsu, A.; Hirade, H.; Kubota, M.; Toyoshima, R.; Fujino, A.; Sirimangkalakitti, N.; P. Concepcion, G. Chemistry of Renieramycins. 16. Structure of 7-Desmethylrenieramycin O (= 14α-Hydroxyrenieramycin S) from Blue Sponge, *Xestospongia* sp. *Heterocycles* **2017**, *95*, 748, doi:10.3987/COM-16-S(S)77.
317. Prakasa Rao, T.S.; Sarma, N.S.; Murthy, Y.L.N.; Kantamreddi, V.S.S.N.; Wright, C.W.; Parameswaran, P.S. New Polyhydroxy Sterols from the Marine Sponge *Callyspongia fibrosa* (Ridley & Dendly). *Tetrahedron Letters* **2010**, *51*, 3583–3586, doi:10.1016/j.tetlet.2010.05.009.
318. Park, S.Y.; Choi, H.; Hwang, H.; Kang, H.; Rho, J.-R. Gukulenins A and B, Cytotoxic Tetraterpenoids from the Marine Sponge *Phorbas gukulensis*. *J. Nat. Prod.* **2010**, *73*, 734–737, doi:10.1021/np900606h.
319. Festa, C.; De Marino, S.; D'Auria, M.V.; Bifulco, G.; Renga, B.; Fiorucci, S.; Petek, S.; Zampella, A. Solomonsterols A and B from *Theonella swinhoei*. The First Example of C-24 and C-23 Sulfated Sterols from a Marine Source Endowed with a PXR Agonistic Activity. *J. Med. Chem.* **2011**, *54*, 401–405, doi:10.1021/jm100968b.
320. De Marino, S.; Ummarino, R.; D'Auria, M.V.; Chini, M.G.; Bifulco, G.; Renga, B.; D'Amore, C.; Fiorucci, S.; Debitus, C.; Zampella, A. Theonellasterols and Conicasterols from *Theonella swinhoei*. Novel Marine Natural Ligands for Human Nuclear Receptors. *J. Med. Chem.* **2011**, *54*, 3065–3075, doi:10.1021/jm200169t.
321. De Marino, S.; Sepe, V.; D'Auria, M.V.; Bifulco, G.; Renga, B.; Petek, S.; Fiorucci, S.; Zampella, A. Towards New Ligands of Nuclear Receptors. Discovery of Malaitasterol A, an Unique Bis-Secosterol from Marine Sponge *Theonella swinhoei*. *Org. Biomol. Chem.* **2011**, *9*, 4856–4862, doi:10.1039/C1OB05378G.
322. Sepe, V.; Ummarino, R.; D'Auria, M.V.; Renga, B.; Fiorucci, S.; Zampella, A. The First Total Synthesis of Solomonsterol B, a Marine Pregnane X Receptor Agonist. *European Journal of Organic Chemistry* **2012**, *2012*, 5187–5194, doi:10.1002/ejoc.201200619.
323. Chini, M.G.; Jones, C.R.; Zampella, A.; D'Auria, M.V.; Renga, B.; Fiorucci, S.; Butts, C.P.; Bifulco, G. Quantitative NMR-Derived Interproton Distances Combined with Quantum Mechanical Calculations of 13C Chemical Shifts in the Stereochemical Determination of Conicasterol F, a Nuclear Receptor Ligand from *Theonella swinhoei*. *J. Org. Chem.* **2012**, *77*, 1489–1496, doi:10.1021/jo2023763.

324. De Marino, S.; Ummarino, R.; D'Auria, M.V.; Chini, M.G.; Bifulco, G.; D'Amore, C.; Renga, B.; Mencarelli, A.; Petek, S.; Fiorucci, S.; et al. 4-Methylenesterols from *Theonella swinhoei* Sponge Are Natural Pregnane-X-Receptor Agonists and Farnesoid-X-Receptor Antagonists That Modulate Innate Immunity. *Steroids* **2012**, *77*, 484–495, doi:10.1016/j.steroids.2012.01.006.
325. Guo, J.-K.; Chiang, C.-Y.; Lu, M.-C.; Chang, W.-B.; Su, J.-H. 4-Methylenesterols from a Sponge *Theonella swinhoei*. *Marine Drugs* **2012**, *10*, 1536–1544, doi:10.3390/md10071536.
326. Nguyen, X.C.; Longeon, A.; Pham, V.C.; Urvois, F.; Bressy, C.; Trinh, T.T.V.; Nguyen, H.N.; Phan, V.K.; Chau, V.M.; Briand, J.-F.; et al. Antifouling 26,27-Cyclosterols from the Vietnamese Marine Sponge *Xestospongia testudinaria*. *J. Nat. Prod.* **2013**, *76*, 1313–1318, doi:10.1021/np400288j.
327. Sepe, V.; D'Amore, C.; Ummarino, R.; Renga, B.; D'Auria, M.V.; Novellino, E.; Sinisi, A.; Taglialatela-Scafati, O.; Nakao, Y.; Limongelli, V.; et al. Insights on Pregnane-X-Receptor Modulation. Natural and Semisynthetic Steroids from *Theonella* Marine Sponges. *Chim.Ther.* **2014**, *73*, 126–134, doi:10.1016/j.ejmech.2013.12.005.
328. Gong, J.; Sun, P.; Jiang, N.; Riccio, R.; Lauro, G.; Bifulco, G.; Li, T.-J.; Gerwick, W.H.; Zhang, W. New Steroids with a Rearranged Skeleton as (h)P300 Inhibitors from the Sponge *Theonella swinhoei*. *Org. Lett.* **2014**, *16*, 2224–2227, doi:10.1021/ol5007345.
329. Youssef, D.T.A.; Badr, J.M.; Shaala, L.A.; Mohamed, G.A.; Bamanie, F.H. Ehrenasterol and Biemnic Acid; New Bioactive Compounds from the Red Sea Sponge *Biemna ehrenbergi*. *Phytochemistry Letters* **2015**, *Complete*, 296–301, doi:10.1016/j.phytol.2015.04.024.
330. Abdelmohsen, U.R.; Cheng, C.; Reimer, A.; Kozjak-Pavlovic, V.; Ibrahim, A.K.; Rudel, T.; Hentschel, U.; Edrada-Ebel, R.; Ahmed, S.A. Antichlamydial Sterol from the Red Sea Sponge *Callyspongia* aff. *implexa*. *Planta Med* **2015**, *81*, 382–387, doi:10.1055/s-0035-1545721.
331. Woo, J.-K.; Ha, T.K.Q.; Oh, D.-C.; Oh, W.-K.; Oh, K.-B.; Shin, J. Polyoxygenated Steroids from the Sponge *Clathria gombawuiensis*. *J. Nat. Prod.* **2017**, *80*, 3224–3233, doi:10.1021/acs.jnatprod.7b00651.
332. Mun, B.; Wang, W.; Kim, H.; Hahn, D.; Yang, I.; Won, D.H.; Kim, E.; Lee, J.; Han, C.; Kim, H.; et al. Cytotoxic 5α,8α-Epidioxy Sterols from the Marine Sponge *Monanchora* sp. *Arch Pharm Res* **2015**, *38*, 18–25, doi:10.1007/s12272-014-0480-8.
333. Wang, W.; Lee, T.G.; Patil, R.S.; Mun, B.; Yang, I.; Kim, H.; Hahn, D.; Won, D.H.; Lee, J.; Lee, Y.; et al. Monanchosterols A and B, Bioactive Bicyclo[4.3.1]Steroids from a Korean Sponge *Monanchora* sp. *J. Nat. Prod.* **2015**, *78*, 368–373, doi:10.1021/np500760v.
334. Pailee, P.; Mahidol, C.; Ruchirawat, S.; Prachyawarakorn, V. Sterols from Thai Marine Sponge *Petrosia* (*Strongylophora*) sp. and Their Cytotoxicity. *Marine Drugs* **2017**, *15*, 54, doi:10.3390/md15030054.
335. Smyrniotopoulos, V.; Rae, M.; Soldatou, S.; Ding, Y.; Wolff, C.W.; McCormack, G.; Coleman, C.M.; Ferreira, D.; Tasdemir, D. Sulfated Steroid–Amino Acid Conjugates from the Irish Marine Sponge *Polymastia boletiformis*. *Marine Drugs* **2015**, *13*, 1632–1646, doi:10.3390/md13041632.

336. Yang, F.; Li, Y.-Y.; Tang, J.; Sun, F.; Lin, H.-W. New 4-Methylidene Sterols from the Marine Sponge *Theonella swinhoei*. *Fitoterapia* **2018**, *127*, 279–285, doi:10.1016/j.fitote.2018.03.006.
337. Li, J.; Tang, H.; Kurtán, T.; Mándi, A.; Zhuang, C.-L.; Su, L.; Zheng, G.-L.; Zhang, W. Swinhoeisterols from the South China Sea Sponge *Theonella swinhoei*. *J Nat Prod* **2018**, *81*, 1645–1650, doi:10.1021/acs.jnatprod.8b00281.
338. Chen, M.; Wu, X.-D.; Zhao, Q.; Wang, C.-Y. Topsensterols A–C, Cytotoxic Polyhydroxylated Sterol Derivatives from a Marine Sponge *Topsentia* sp. *Marine Drugs* **2016**, *14*, 146, doi:10.3390/md14080146.
339. Chen, M.; Wu, X.-D.; Hao, J.-D.; Wang, C.-Y. Two New Polyhydroxylated Sterol Derivatives from the Sponge *Topsentia* sp. Collected from the South China Sea. *Chem Nat Compd* **2018**, *54*, 301–304, doi:10.1007/s10600-018-2328-9.
340. Cheng, Z.; Liu, D.; de Voogd, N.J.; Proksch, P.; Lin, W. New Sterol Derivatives from the Marine Sponge *Xestospongia* sp. *Helvetica Chimica Acta* **2016**, *99*, 588–596, doi:10.1002/hlca.201600021.
341. He, W.-F.; Xue, D.-Q.; Yao, L.-G.; Li, J.; Liu, H.-L.; Guo, Y.-W. A New Bioactive Steroidal Ketone from the South China Sea Sponge *Xestospongia testudinaria*. *Journal of Asian Natural Products Research* **2016**, *18*, 195–199, doi:10.1080/10286020.2015.1056521.
342. El-Gamal, A.A.; Al-Massarani, S.M.; Shaala, L.A.; Alahdald, A.M.; Al-Said, M.S.; Ashour, A.E.; Kumar, A.; Abdel-Kader, M.S.; Abdel-Mageed, W.M.; Youssef, D.T.A. Cytotoxic Compounds from the Saudi Red Sea Sponge *Xestospongia testudinaria*. *Marine Drugs* **2016**, *14*, 82, doi:10.3390/md14050082.
343. Li, H.; Bowling, J.J.; Fronczek, F.R.; Hong, J.; Jabba, S.V.; Murray, T.F.; Ha, N.-C.; Hamann, M.T.; Jung, J.H. Asteropsin A: An Unusual Cystine-Crosslinked Peptide from Porifera Enhances Neuronal Ca2+ Influx. *Biochimica et Biophysica Acta (BBA) - General Subjects* **2013**, *1830*, 2591–2599, doi:10.1016/j.bbagen.2012.11.015.
344. Cheng, W.; Liu, D.; Zhang, F.; Zhang, Q.; Pedpradab, P.; Proksch, P.; Liang, H.; Lin, W. Formamidobisabolene-Based Derivatives from a Sponge *Axinyssa* sp. *Tetrahedron* **2014**, *70*, 3576–3583, doi:10.1016/j.tet.2014.04.008.
345. Tian, L.-W.; Feng, Y.; Shimizu, Y.; Pfeifer, T.A.; Wellington, C.; Hooper, J.N.A.; Quinn, R.J. ApoE Secretion Modulating Bromotyrosine Derivative from the Australian Marine Sponge *Callyspongia* sp. *Bioorg Med Chem Lett* **2014**, *24*, 3537–3540, doi:10.1016/j.bmcl.2014.05.054.
346. Yang, F.; Zou, Y.; Wang, R.-P.; Hamann, M.T.; Zhang, H.-J.; Jiao, W.-H.; Han, B.-N.; Song, S.-J.; Lin, H.-W. Relative and Absolute Stereochemistry of Diacarperoxides: Antimalarial Norditerpene Endoperoxides from Marine Sponge *Diacarnus megaspinorhabdosa*. *Marine Drugs* **2014**, *12*, 4399–4416, doi:10.3390/md12084399.
347. Woo, J.-K.; Kim, C.-K.; Kim, S.-H.; Kim, H.; Oh, D.-C.; Oh, K.-B.; Shin, J. Gombaspiroketals A–C, Sesterterpenes from the Sponge *Clathria gombawuiensis*. *Org. Lett.* **2014**, *16*, 2826–2829, doi:10.1021/ol500868s.

348. Angawi, R.F.; Saqer, E.; Abdel-Lateff, A.; Badria, F.A.; Ayyad, S.-E.N. Cytotoxic Neviotane Triterpene-Type from the Red Sea Sponge *Siphonochalina siphonella*. *Pharmacognosy Magazine* **2014**, *10*, 334, doi:10.4103/0973-1296.133292.
349. Lee, Y.; Wang, W.; Kim, H.; Giri, A.G.; Won, D.H.; Hahn, D.; Baek, K.R.; Lee, J.; Yang, I.; Choi, H.; et al. Phorbaketals L-N, Cytotoxic Sesterterpenoids Isolated from the Marine Sponge of the Genus *Phorbas*. *Bioorg Med Chem Lett* **2014**, *24*, 4095–4098, doi:10.1016/j.bmcl.2014.07.066.
350. Yamakuma, M.; Tsukamoto, S.; Kato, H.; Matsuo, K.; H. El-Desoky, A.; Kawabata, T.; Losung, F.; E. P. Mangindaan, R.; J. de Voogd, N.; Yokosawa, H. 1-Hydroxyethylhalenaquinone: A New Proteasome Inhibitor from the Marine Sponge *Xestospongia* sp. *Heterocycles* **2014**, *89*, 2605, doi:10.3987/COM-14-13087.
351. Göthel, Q.; Köck, M. New Sesquiterpene Hydroquinones from the Caribbean Sponge *Aka coralliphagum*. *Beilstein J. Org. Chem.* **2014**, *10*, 613–621, doi:10.3762/bjoc.10.52.
352. Rho, J.-R. Four sesquiterpenes isolated from a Marine Sponge *Topsentia* species. *Journal of the Korean Magnetic Resonance Society* **2014**, *18*, 82–88, doi:10.6564/JKMRS.2014.18.2.082.
353. Liu, W.; Liang, K.-J.; Chiang, C.-Y.; Lu, M.-C.; Su, J.-H. New Nitrogenous Bisabolene-Type Sesquiterpenes from a Formosan Sponge *Axinyssa* sp. *Chem Pharm Bull (Tokyo)* **2014**, *62*, 392–394, doi:10.1248/cpb.c13-00851.
354. Xu, Y.; Lang, J.-H.; Jiao, W.-H.; Wang, R.-P.; Peng, Y.; Song, S.-J.; Zhang, B.-H.; Lin, H.-W. Formamido-Diterpenes from the South China Sea Sponge *Acanthella cavernosa*. *Marine Drugs* **2012**, *10*, 1445–1458, doi:10.3390/md10071445.
355. Xu, Y.; Li, N.; Jiao, W.-H.; Wang, R.-P.; Peng, Y.; Qi, S.-H.; Song, S.-J.; Chen, W.-S.; Lin, H.-W. Antifouling and Cytotoxic Constituents from the South China Sea Sponge *Acanthella cavernosa*. *Tetrahedron* **2012**, *68*, 2876–2883, doi:10.1016/j.tet.2012.01.084.
356. Pettit, G.R.; Tang, Y.; Zhang, Q.; Bourne, G.T.; Arm, C.A.; Leet, J.E.; Knight, J.C.; Pettit, R.K.; Chapuis, J.-C.; Doubek, D.L.; et al. Isolation and Structures of Axistatins 1–3 from the Republic of Palau Marine Sponge *Agelas axifera* Hentschel. *J. Nat. Prod.* **2013**, *76*, 420–424, doi:10.1021/np300828y.
357. Stout, E.P.; Yu, L.C.; Molinski, T.F. Antifungal Diterpene Alkaloids from the Caribbean Sponge *Agelas citrina*: Unified Configurational Assignments of Agelasidines and Agelasines. *European Journal of Organic Chemistry* **2012**, *2012*, 5131–5135, doi:10.1002/ejoc.201200572.
358. Kubota, T.; Iwai, T.; Takahashi-Nakaguchi, A.; Fromont, J.; Gonoi, T.; Kobayashi, J. Agelasines O–U, New Diterpene Alkaloids with a 9-N-Methyladenine Unit from a Marine Sponge *Agelas* sp. *Tetrahedron* **2012**, *68*, 9738–9744, doi:10.1016/j.tet.2012.09.040.
359. Shubina, L.K.; Kalinovsky, A.I.; Makarieva, T.N.; Fedorov, S.N.; Dyshlovoy, S.A.; Dmitrenok, P.S.; Kapustina, I.I.; Mollo, E.; Utkina, N.K.; Krasokhin, V.B.; et al. New Meroterpenoids from the Marine Sponge *Aka coralliphaga*. *Natural Product Communications* **2012**, *7*, 487–490, doi:10.1177/1934578X1200700418.
360. Robinson, S.J.; Morinaka, B.I.; Amagata, T.; Tenney, K.; Bray, W.M.; Gassner, N.C.; Lokey, R.S.; Crews, P. New Structures and Bioactivity Properties of Jasplakinolide (Jaspamide) Analogues from Marine Sponges. *J. Med. Chem.* **2010**, *53*, 1651–1661, doi:10.1021/jm9013554.

361. Lefranc, F.; Nuzzo, G.; Hamdy, N.A.; Fakhr, I.; Moreno Y Banuls, L.; Van Goietsenoven, G.; Villani, G.; Mathieu, V.; van Soest, R.; Kiss, R.; et al. In Vitro Pharmacological and Toxicological Effects of Norterpene Peroxides Isolated from the Red Sea Sponge *Diacarnus erythraeanus* on Normal and Cancer Cells. *J. Nat. Prod.* **2013**, *76*, 1541–1547, doi:10.1021/np400107t.
362. Ibrahim, S.R.M. Diacarperoxide S, New Norterpene Cyclic Peroxide from the Sponge Diacarnus Megaspinorhabdosa. *Nat Prod Commun* **2012**, *7*, 9–12.
363. Kumar, R.; Subramani, R.; Feussner, K.-D.; Aalbersberg, W. Aurantoside K, a New Antifungal Tetramic Acid Glycoside from a Fijian Marine Sponge of the *Genus melophlus*. *Marine Drugs* **2012**, *10*, 200–208, doi:10.3390/md10010200.
364. Venturi, V.; Davies, C.; Singh, A.J.; Matthews, J.H.; Bellows, D.S.; Northcote, P.T.; Keyzers, R.A.; Teesdale-Spittle, P.H. The Protein Synthesis Inhibitors Mycalamides A and E Have Limited Susceptibility toward the Drug Efflux Network. *Journal of Biochemical and Molecular Toxicology* **2012**, *26*, 94–100, doi:10.1002/jbt.20414.
365. Yegdaneh, A.; Putchakarn, S.; Yuenyongsawad, S.; Ghannadi, A.; Plubrukarn, A. 3-Oxoabolene and 1-Oxocurcuphenol, Aromatic Bisabolanes from the Sponge *Myrmekioderma* sp. *Natural Product Communications* **2013**, *8*, 1355–1357, doi:10.1177/1934578X1300801002.
366. Chao, C.-H.; Chou, K.-J.; Wang, G.-H.; Wu, Y.-C.; Wang, L.-H.; Chen, J.-P.; Sheu, J.-H.; Sung, P.-J. Norterpenoids and Related Peroxides from the Formosan Marine Sponge *Negombata corticata*. *J. Nat. Prod.* **2010**, *73*, 1538–1543, doi:10.1021/np100353x.
367. Winder, P.L.; Baker, H.L.; Linley, P.; Guzmán, E.A.; Pomponi, S.A.; Cristina Diaz, M.; Reed, J.K.; Wright, A.E. Neopetrosiquinones A and B, Sesquiterpene Benzoquinones Isolated from the Deep-Water Sponge *Neopetrosia* cf. *proxima*. *Bioorganic & Medicinal Chemistry* **2011**, *19*, 6599–6603, doi:10.1016/j.bmc.2011.09.026.
368. Du, L.; Mahdi, F.; Datta, S.; Jekabsons, M.B.; Zhou, Y.-D.; Nagle, D.G. Structures and Mechanisms of Antitumor Agents: Xestoquinones Uncouple Cellular Respiration and Disrupt HIF Signaling in Human Breast Tumor Cells. *J. Nat. Prod.* **2012**, *75*, 1553–1559, doi:10.1021/np3002892.
369. Nhiem, N.X.; Quang, N.V.; Minh, C.V.; Hang, D.T.T.; Anh, H.L.T.; Tai, B.H.; Yen, P.H.; Hoai, N.T.; Thung, D.C.; Kiem, P.V. Biscembranoids from the Marine Sponge *Petrosia nigricans*. *Nat Prod Commun* **2013**, *8*, 1209–1212.
370. Jeon, J.; Liao, L.; Kim, H.; Sim, C.J.; Oh, D.-C.; Oh, K.-B.; Shin, J. Cytotoxic Diterpenoid Pseudodimers from the Korean Sponge *Phorbas gukhulensis*. *J. Nat. Prod.* **2013**, *76*, 1679–1685, doi:10.1021/np400389c.
371. Rho, J.-R.; Hwang, B.S.; Joung, S.; Byun, M.R.; Hong, J.-H.; Lee, H.-Y. Phorbasones A and B, Sesterterpenoids Isolated from the Marine Sponge *Phorbas* sp. and Induction of Osteoblast Differentiation. *Org. Lett.* **2011**, *13*, 884–887, doi:10.1021/ol1029386.
372. Wang, W.; Lee, Y.; Lee, T.G.; Mun, B.; Giri, A.G.; Lee, J.; Kim, H.; Hahn, D.; Yang, I.; Chin, J.; et al. Phorone A and Isophorbasone A, Sesterterpenoids Isolated from the Marine Sponge *Phorbas* sp. *Org. Lett.* **2012**, *14*, 4486–4489, doi:10.1021/ol3019874.

373. Daoust, J.; Chen, M.; Wang, M.; Williams, D.E.; Chavez, M.A.G.; Wang, Y.A.; Merchant, C.E.; Fontana, A.; Kieffer, T.J.; Andersen, R.J. Sesterterpenoids Isolated from a Northeastern Pacific *Phorbas* sp. *J. Org. Chem.* **2013**, *78*, 8267–8273, doi:10.1021/jo4014589.
374. Lamoral-Theys, D.; Fattorusso, E.; Mangoni, A.; Perinu, C.; Kiss, R.; Costantino, V. Evaluation of the Antiproliferative Activity of Diterpene Isonitriles from the Sponge *Pseudoaxinella flava* in Apoptosis-Sensitive and Apoptosis-Resistant Cancer Cell Lines. *J Nat Prod* **2011**, *74*, 2299–2303, doi:10.1021/np2005055.
375. Li, J.; Xu, B.; Cui, J.; Deng, Z.; de Voogd, N.J.; Proksch, P.; Lin, W. Globostelletins A–I, Cytotoxic Isomalabaricane Derivatives from the Marine Sponge *Rhabdastrella globostellata*. *Bioorganic & Medicinal Chemistry* **2010**, *18*, 4639–4647, doi:10.1016/j.bmc.2010.05.029.
376. Hirashima, M.; Tsuda, K.; Hamada, T.; Okamura, H.; Furukawa, T.; Akiyama, S.; Tajitsu, Y.; Ikeda, R.; Komatsu, M.; Doe, M.; et al. Cytotoxic Isomalabaricane Derivatives and a Monocyclic Triterpene Glycoside from the Sponge *Rhabdastrella globostellata*. *J. Nat. Prod.* **2010**, *73*, 1512–1518, doi:10.1021/np100302a.
377. Tanaka, N.; Momose, R.; Shibazaki, A.; Gonoi, T.; Fromont, J.; Kobayashi, J. Stelliferins J–N, Isomalabaricane-Type Triterpenoids from Okinawan Marine Sponge *Rhabdastrella* cf. *globostellata*. *Tetrahedron* **2011**, *67*, 6689–6696, doi:10.1016/j.tet.2011.04.095.
378. Chanthathamrongsiri, N.; Yuenyongsawad, S.; Wattanapiromsakul, C.; Plubrukarn, A. Bifunctionalized Amphilectane Diterpenes from the Sponge *Stylissa* cf. *massa*. *J. Nat. Prod.* **2012**, *75*, 789–792, doi:10.1021/np200959j.
379. Avilés, E.; Rodríguez, A.D.; Vicente, J. Two Rare-Class Tricyclic Diterpenes with Antitubercular Activity from the Caribbean Sponge *Svenzea flava*. Application of Vibrational Circular Dichroism Spectroscopy for Determining Absolute Configuration. *J. Org. Chem.* **2013**, *78*, 11294–11301, doi:10.1021/jo401846m.
380. Costantino, V.; Fattorusso, E.; Mangoni, A.; Perinu, C.; Teta, R.; Panza, E.; Ianaro, A. Tedarenes A and B: Structural and Stereochemical Analysis of Two New Strained Cyclic Diarylheptanoids from the Marine Sponge *Tedania ignis*. *J. Org. Chem.* **2012**, *77*, 6377–6383, doi:10.1021/jo300295j.
381. De Marino, S.; Festa, C.; D'Auria, M.V.; Cresteil, T.; Debitus, C.; Zampella, A. Swinholide J, a Potent Cytotoxin from the Marine Sponge *Theonella swinhoei*. *Marine Drugs* **2011**, *9*, 1133–1141, doi:10.3390/md9061133.
382. Sinisi, A.; Calcinai, B.; Cerrano, C.; Dien, H.A.; Zampella, A.; D'Amore, C.; Renga, B.; Fiorucci, S.; Taglialatela-Scafati, O. Isoswinholide B and Swinholide K, Potently Cytotoxic Dimeric Macrolides from *Theonella swinhoei*. *Bioorganic & Medicinal Chemistry* **2013**, *21*, 5332–5338, doi:10.1016/j.bmc.2013.06.015.
383. Longeon, A.; Copp, B.R.; Roué, M.; Dubois, J.; Valentin, A.; Petek, S.; Debitus, C.; Bourguet-Kondracki, M.-L. New Bioactive Halenaquinone Derivatives from South Pacific Marine Sponges of the *Genus xestospongia*. *Bioorganic & Medicinal Chemistry* **2010**, *18*, 6006–6011, doi:10.1016/j.bmc.2010.06.066.
384. Millán-Aguiñaga, N.; Soria-Mercado, I.E.; Williams, P. Xestosaprol D and E from the Indonesian Marine Sponge *Xestospongia* sp. *Tetrahedron Letters* **2010**, *51*, 751–753, doi:10.1016/j.tetlet.2009.11.132.

385. Dai, J.; Sorribas, A.; Yoshida, W.Y.; Kelly, M.; Williams, P.G. Xestosaprols from the Indonesian Marine Sponge *Xestospongia* sp. *J. Nat. Prod.* **2010**, *73*, 1188–1191, doi:10.1021/np100203x.
386. Pedpradab, P.; Suwanborirux, K. A New Acyclic Thiophene Sesterterpene from the Sikao Bay Sponge, *Xestospongia* sp. *J Asian Nat Prod Res* **2011**, *13*, 879–883, doi:10.1080/10286020.2011.591386.
387. Abdjul, D.B.; Yamazaki, H.; Kanno, S.; Takahashi, O.; Kirikoshi, R.; Ukai, K.; Namikoshi, M. Structures and Biological Evaluations of Agelasines Isolated from the Okinawan Marine Sponge *Agelas nakamurai*. *J. Nat. Prod.* **2015**, *78*, 1428–1433, doi:10.1021/acs.jnatprod.5b00375.
388. Ki, D.-W.; Awouafack, M.D.; Wong, C.P.; Nguyen, H.M.; Thai, Q.M.; Ton Nu, L.H.; Morita, H. Brominated Diphenyl Ethers Including a New Tribromoiododiphenyl Ether from the Vietnamese Marine Sponge *Arenosclera* sp. and Their Antibacterial Activities. *Chem Biodivers* **2019**, *16*, e1800593, doi:10.1002/cbdv.201800593.
389. Woo, J.-K.; Kim, C.-K.; Ahn, C.-H.; Oh, D.-C.; Oh, K.-B.; Shin, J. Additional Sesterterpenes and a Nortriterpene Saponin from the Sponge *Clathria gombawuiensis*. *J. Nat. Prod.* **2015**, *78*, 218–224, doi:10.1021/np500753q.
390. Wang, G.; Ji, R.; Cao, M.; Tang, W.; Yu, H.; Gu, B.; Ding, L.; Peng, S.; Jiao, W.; Sun, F.; et al. New Metabolites from the South China Sea Sponge *Diacarnus megaspinorhabdosa*. *Chemical and Pharmaceutical Bulletin* **2015**, *63*, 438–442, doi:10.1248/cpb.c15-00022.
391. Yang, F.; Wang, R.-P.; Xu, B.; Yu, H.-B.; Ma, G.-Y.; Wang, G.-F.; Dai, S.-W.; Zhang, W.; Jiao, W.-H.; Song, S.-J.; et al. New Antimalarial Norterpene Cyclic Peroxides from Xisha Islands Sponge *Diacarnus megaspinorhabdosa*. *Bioorganic & Medicinal Chemistry Letters* **2016**, *26*, 2084–2087, doi:10.1016/j.bmcl.2016.02.070.
392. Costa, M.; Coello, L.; Urbatzka, R.; Pérez, M.; Thorsteinsdottir, M. New Aromatic Bisabolane Derivatives with Lipid-Reducing Activity from the Marine Sponge *Myrmekioderma* sp. *Mar Drugs* **2019**, *17*, E375, doi:10.3390/md17060375.
393. Noda, A.; Sakai, E.; Kato, H.; Losung, F.; Mangindaan, R.E.P.; de Voogd, N.J.; Yokosawa, H.; Tsukamoto, S. Strongylophorines, Meroditerpenoids from the Marine Sponge *Petrosia corticata*, Function as Proteasome Inhibitors. *Bioorg Med Chem Lett* **2015**, *25*, 2650–2653, doi:10.1016/j.bmcl.2015.04.075.
394. Solanki, H.; Angulo-Preckler, C.; Calabro, K.; Kaur, N.; Lasserre, P.; Cautain, B.; de la Cruz, M.; Reyes, F.; Avila, C.; Thomas, O.P. Suberitane Sesterterpenoids from the Antarctic Sponge *Phorbas areolatus* (Thiele, 1905). *Tetrahedron Letters* **2018**, *59*, 3353–3356, doi:10.1016/j.tetlet.2018.07.055.
395. Wang, M.; Tietjen, I.; Chen, M.; Williams, D.E.; Daoust, J.; Brockman, M.A.; Andersen, R.J. Sesterterpenoids Isolated from the Sponge *Phorbas* sp. Activate Latent HIV-1 Provirus Expression. *J. Org. Chem.* **2016**, *81*, 11324–11334, doi:10.1021/acs.joc.6b02312.
396. Lhullier, C.; de Oliveira Tabalipa, E.; Nienkötter Sardá, F.; Sandjo, L.P.; Zanchett Schneider, N.F.; Carraro, J.L.; Oliveira Simões, C.M.; Schenkel, E.P. Clerodane Diterpenes from the Marine Sponge *Raspailia bouryesnaultae* Collected in South Brazil. *Marine Drugs* **2019**, *17*, 57, doi:10.3390/md17010057.

397. Dung, D.T.; Yen, P.H.; Nhiem, N.X.; Quang, T.H.; Tai, B.H.; Van Minh, C.; Kim, D.C.; Oh, H.; Kim, Y.C.; Van Kiem, P. New Acetylated Terpenoids from Sponge *Rhabdastrella providentiae* Inhibit NO Production in LPS Stimulated BV2 Cells. *Natural Product Communications* **2018**, *13*, 1934578X1801300602, doi:10.1177/1934578X1801300602.
398. Kiem, P.V.; Dung, D.T.; Yen, P.H.; Nhiem, N.X.; Quang, T.H.; Tai, B.H.; Minh, C.V. New Isomalabaricane Analogues from the Sponge *Rhabdastrella providentiae* and Their Cytotoxic Activities. *Phytochemistry Letters* **2018**, *26*, 199–204, doi:10.1016/j.phytol.2018.06.003.
399. El-Beih, A.A.; El-Desoky, A.H.; Al-Hammady, M.A.; Elshamy, A.I.; Hegazy, M.-E.F.; Kato, H.; Tsukamoto, S. New Inhibitors of RANKL-Induced Osteoclastogenesis from the Marine Sponge *Siphonochalina siphonella*. *Fitoterapia* **2018**, *128*, 43–49, doi:10.1016/j.fitote.2018.05.001.
400. Kolesnikova, S.A.; Lyakhova, E.G.; Kalinovsky, A.I.; Berdyshev, D.V.; Pislyagin, E.A.; Popov, R.S.; Grebnev, B.B.; Makarieva, T.N.; Minh, C.V.; Stonik, V.A. Cyclobutastellettolides A and B, C19 Norterpenoids from a *Stelletta* sp. Marine Sponge. *J Nat Prod* **2019**, *82*, 3196–3200, doi:10.1021/acs.jnatprod.9b00824.
401. Li, Y.; Tang, H.; Tian, X.; Lin, H.; Wang, M.; Yao, M. Three New Cytotoxic Isomalabaricane Triterpenes from the Marine Sponge *Stelletta tenuis*. *Fitoterapia* **2015**, *106*, 226–230, doi:10.1016/j.fitote.2015.09.012.
402. Lee, J.-S.; Abdjul, D.B.; Yamazaki, H.; Takahashi, O.; Kirikoshi, R.; Ukai, K.; Namikoshi, M. Strongylophorines, New Protein Tyrosine Phosphatase 1B Inhibitors, from the Marine Sponge *Strongylophora strongilata* Collected at Iriomote Island. *Bioorg Med Chem Lett* **2015**, *25*, 3900–3902, doi:10.1016/j.bmcl.2015.07.039.
403. Avilés, E.; Prudhomme, J.; Le Roch, K.G.; Rodríguez, A.D. Structures, Semisyntheses, and Absolute Configurations of the Antiplasmodial α-Substituted β-Lactam Monamphilectines B and C from the Sponge *Svenzea flava*. *Tetrahedron* **2015**, *71*, 487–494, doi:10.1016/j.tet.2014.11.060.
404. Davison, J.R.; Lohith, K.M.; Wang, X.; Bobyk, K.; Mandadapu, S.R.; Lee, S.-L.; Cencic, R.; Nelson, J.; Simpkins, S.; Frank, K.M.; et al. A New Natural Product Analog of Blasticidin S Reveals Cellular Uptake Facilitated by the NorA Multidrug Transporter. *Antimicrobial Agents and Chemotherapy* **2017**, doi:10.1128/AAC.02635-16.
405. He, F.; Mai, L.H.; Longeon, A.; Copp, B.R.; Loaëc, N.; Bescond, A.; Meijer, L.; Bourguet-Kondracki, M.-L. Novel Adociaquinone Derivatives from the Indonesian Sponge *Xestospongia* sp. *Marine Drugs* **2015**, *13*, 2617–2628, doi:10.3390/md13052617.
406. Huang, R.-Y.; Chen, W.-T.; Kurtán, T.; Mándi, A.; Ding, J.; Li, J.; Li, X.-W.; Guo, Y.-W. Bioactive Isoquinolinequinone Alkaloids from the South China Sea Nudibranch *Jorunna funebris* and Its Sponge-Prey Xestospongia sp. *Future Medicinal Chemistry* **2016**, *8*, 17–27, doi:10.4155/fmc.15.169.
407. Ren, S.; Ma, W.; Xu, T.; Lin, X.; Yin, H.; Yang, B.; Zhou, X.-F.; Yang, X.-W.; Long, L.; Lee, K.J.; et al. Two Novel Alkaloids from the South China Sea Marine Sponge *Dysidea* sp. *The Journal of Antibiotics* **2010**, *63*, 699–701, doi:10.1038/ja.2010.134.

408. Bishara, A.; Rudi, A.; Aknin, M.; Neumann, D.; Ben-Califa, N.; Kashman, Y. Salarins D–J, Seven New Nitrogenous Macrolides from the Madagascar Sponge *Fascaplysinopsis* sp. *Tetrahedron* **2010**, *66*, 4339–4345, doi:10.1016/j.tet.2010.04.035.
409. Yang, X.; Davis, R.A.; Buchanan, M.S.; Duffy, S.; Avery, V.M.; Camp, D.; Quinn, R.J. Antimalarial Bromotyrosine Derivatives from the Australian Marine Sponge *Hyattella* sp. *J. Nat. Prod.* **2010**, *73*, 985–987, doi:10.1021/np900834g.
410. Inman, W.D.; Bray, W.M.; Gassner, N.C.; Lokey, R.S.; Tenney, K.; Shen, Y.Y.; Tendyke, K.; Suh, T.; Crews, P. A Beta-Carboline Alkaloid from the Papua New Guinea Marine Sponge *Hyrtios reticulatus*. *J Nat Prod* **2010**, *73*, 255–257, doi:10.1021/np9005426.
411. Yamanokuchi, R.; Imada, K.; Miyazaki, M.; Kato, H.; Watanabe, T.; Fujimuro, M.; Saeki, Y.; Yoshinaga, S.; Terasawa, H.; Iwasaki, N.; et al. Hyrtioreticulins A–E, Indole Alkaloids Inhibiting the Ubiquitin-Activating Enzyme, from the Marine Sponge *Hyrtios reticulatus*. *Bioorganic & Medicinal Chemistry* **2012**, *20*, 4437–4442, doi:10.1016/j.bmc.2012.05.044.
412. Longeon, A.; Copp, B.R.; Quévrain, E.; Roué, M.; Kientz, B.; Cresteil, T.; Petek, S.; Debitus, C.; Bourguet-Kondracki, M.-L. Bioactive Indole Derivatives from the South Pacific Marine Sponges *Rhopaloeides odorabile* and Hyrtios sp. *Mar Drugs* **2011**, *9*, 879–888, doi:10.3390/md9050879.
413. Youssef, D.T.A.; Shaala, L.A.; Asfour, H.Z. Bioactive Compounds from the Red Sea Marine Sponge *Hyrtios* species. *Marine Drugs* **2013**, *11*, 1061–1070, doi:10.3390/md11041061.
414. Momose, R.; Tanaka, N.; Fromont, J.; Kobayashi, J. Hyrtimomines A–C, New Heteroaromatic Alkaloids from a Sponge *Hyrtios* sp. *Org. Lett.* **2013**, *15*, 2010–2013, doi:10.1021/ol400687b.
415. Tanaka, N.; Momose, R.; Takahashi, Y.; Kubota, T.; Takahashi-Nakaguchi, A.; Gonoi, T.; Fromont, J.; Kobayashi, J. Hyrtimomines D and E, Bisindole Alkaloids from a Marine Sponge *Hyrtios* sp. *Tetrahedron Letters* **2013**, *54*, 4038–4040, doi:10.1016/j.tetlet.2013.05.073.
416. Taufa, T.; Singh, A.J.; Harland, C.R.; Patel, V.; Jones, B.; Halafihi, T.I.; Miller, J.H.; Keyzers, R.A.; Northcote, P.T. Zampanolides B-E from the Marine Sponge *Cacospongia mycofijiensis*: Potent Cytotoxic Macrolides with Microtubule-Stabilizing Activity. *J Nat Prod* **2018**, *81*, 2539–2544, doi:10.1021/acs.jnatprod.8b00641.
417. Wang, Q.; Tang, X.; Luo, X.; de Voogd, N.J.; Li, P.; Li, G. (+)- and (−)-Spiroreticulatine, A Pair of Unusual Spiro Bisheterocyclic Quinoline-Imidazole Alkaloids from the South China Sea Sponge *Fascaplysinopsis reticulata*. *Org. Lett.* **2015**, *17*, 3458–3461, doi:10.1021/acs.orglett.5b01503.
418. Wang, Q.; Tang, X.-L.; Luo, X.-C.; de Voog, N.J.; Li, P.-L.; Li, G.-Q. Aplysinopsin-Type and Bromotyrosine-Derived Alkaloids from the South China Sea Sponge *Fascaplysinopsis reticulata*. *Sci Rep* **2019**, *9*, 2248, doi:10.1038/s41598-019-38696-3.
419. Campos, P.-E.; Pichon, E.; Moriou, C.; Clerc, P.; Trépos, R.; Frederich, M.; De Voogd, N.; Hellio, C.; Gauvin-Bialecki, A.; Al-Mourabit, A. New Antimalarial and Antimicrobial Tryptamine Derivatives from the Marine Sponge *Fascaplysinopsis reticulata*. *Mar Drugs* **2019**, *17*, E167, doi:10.3390/md17030167.

420. Tarazona, G.; Santamaría, G.; Cruz, P.G.; Fernández, R.; Pérez, M.; Martínez-Leal, J.F.; Rodríguez, J.; Jiménez, C.; Cuevas, C. Cytotoxic Anomoian B and Aplyzanzine B, New Bromotyrosine Alkaloids from Indonesian Sponges. *ACS Omega* **2017**, *2*, 3494–3501, doi:10.1021/acsomega.7b00417.
421. Kubota, T.; Nakamura, K.; Sakai, K.; Fromont, J.; Gonoi, T.; Kobayashi, J. Hyrtinadines C and D, New Azepinoindole-Type Alkaloids from a Marine Sponge *Hyrtios* sp. *Chem Pharm Bull (Tokyo)* **2016**, *64*, 975–978, doi:10.1248/cpb.c16-00201.
422. Park, S.-I.; Lee, Y.-J.; Won, H.; Oh, K.-B.; Lee, H.-S. Indole Alkaloids from Tropical Sponge *Hyrtios* sp. as Isocitrate Lyase Inhibitors. *Natural Product Communications* **2018**, *13*, 1934578X1801300608, doi:10.1177/1934578X1801300608.
423. Takahashi, H.; Kurimoto, S.; Kobayashi, J.; Kubota, T. Ishigadine A, a New Canthin-6-One Alkaloid from an Okinawan Marine Sponge *Hyrtios* sp. *Tetrahedron Letters* **2018**, *59*, 4500–4502, doi:10.1016/j.tetlet.2018.11.019.
424. He, W.-F.; Xue, D.-Q.; Yao, L.-G.; Li, J.-Y.; Li, J.; Guo, Y.-W. Hainanerectamines A–C, Alkaloids from the Hainan Sponge *Hyrtios erecta*. *Marine Drugs* **2014**, *12*, 3982–3993, doi:10.3390/md12073982.
425. Tanaka, N.; Momose, R.; Takahashi-Nakaguchi, A.; Gonoi, T.; Fromont, J.; Kobayashi, J. Hyrtimomines, Indole Alkaloids from Okinawan Marine Sponges *Hyrtios* spp. *Tetrahedron* **2014**, *70*, 832–837, doi:10.1016/j.tet.2013.12.032.
426. Khokhar, S.; Feng, Y.; Campitelli, M.R.; Ekins, M.G.; Hooper, J.N.A.; Beattie, K.D.; Sadowski, M.C.; Nelson, C.C.; Davis, R.A. Isolation, Structure Determination and Cytotoxicity Studies of Tryptophan Alkaloids from an Australian Marine Sponge *Hyrtios* sp. *Bioorganic & Medicinal Chemistry Letters* **2014**, *24*, 3329–3332, doi:10.1016/j.bmcl.2014.05.104.
427. Sakai, E.; Kato, H.; Rotinsulu, H.; Losung, F.; Mangindaan, R.E.P.; de Voogd, N.J.; Yokosawa, H.; Tsukamoto, S. Variabines A and B: New β-Carboline Alkaloids from the Marine Sponge *Luffariella variabilis*. *J Nat Med* **2014**, *68*, 215–219, doi:10.1007/s11418-013-0778-8.
428. Trianto, A.; de Voodg, N.J.; Tanaka, J. Two New Compounds from an Indonesian Sponge *Dysidea* sp. *Journal of Asian Natural Products Research* **2014**, *16*, 163–168, doi:10.1080/10286020.2013.844128.
429. Harinantenaina, L.; Brodie, P.J.; Maharavo, J.; Bakary, G.; TenDyke, K.; Shen, Y.; Kingston, D.G.I. Antiproliferative Homoscalarane Sesterterpenes from Two Madagascan Sponges. *Bioorganic & Medicinal Chemistry* **2013**, *21*, 2912–2917, doi:10.1016/j.bmc.2013.03.078.
430. Fuh, Y.-M.; Lu, M.-C.; Lee, C.-H.; Su, J.-H. Cytotoxic Scalarane Sesterterpenoids from a Marine Sponge *Hippospongia* sp. *Natural Product Communications* **2013**, *8*, 1934578X1300800504, doi:10.1177/1934578X1300800504.
431. Kim, C.-K.; Song, I.-H.; Park, H.Y.; Lee, Y.-J.; Lee, H.-S.; Sim, C.J.; Oh, D.-C.; Oh, K.-B.; Shin, J. Suvanine Sesterterpenes and Deacyl Irciniasulfonic Acids from a Tropical *Coscinoderma* sp. Sponge. *J Nat Prod* **2014**, *77*, 1396–1403, doi:10.1021/np500156n.

432. Lai, K.-H.; Liu, Y.-C.; Su, J.-H.; El-Shazly, M.; Wu, C.-F.; Du, Y.-C.; Hsu, Y.-M.; Yang, J.-C.; Weng, M.-K.; Chou, C.-H.; et al. Antileukemic Scalarane Sesterterpenoids and Meroditerpenoid from *Carteriospongia* (*Phyllospongia*) sp., Induce Apoptosis via Dual Inhibitory Effects on Topoisomerase II and Hsp90. *Sci Rep* **2016**, *6*, 36170, doi:10.1038/srep36170.
433. Lu, Y.; Zhao, M. Two Highly Acetylated Sterols from the Marine Sponge *Dysidea* sp. *Zeitschrift für Naturforschung B* **2017**, *72*, 49–52, doi:10.1515/znb-2016-0156.
434. Elhady, S.S.; Al-Abd, A.M.; El-Halawany, A.M.; Alahdal, A.M.; Hassanean, H.A.; Ahmed, S.A. Antiproliferative Scalarane-Based Metabolites from the Red Sea Sponge *Hyrtios erectus*. *Marine Drugs* **2016**, *14*, 130, doi:10.3390/md14070130.
435. Elhady, S.S.; El-Halawany, A.M.; Alahdal, A.M.; Hassanean, H.A.; Ahmed, S.A. A New Bioactive Metabolite Isolated from the Red Sea Marine Sponge *Hyrtios erectus*. *Molecules* **2016**, *21*, 82, doi:10.3390/molecules21010082.
436. Takahashi, Y.; Iinuma, Y.; Kubota, T.; Tsuda, M.; Sekiguchi, M.; Mikami, Y.; Fromont, J.; Kobayashi, J. Hyrtioseragamines A and B, New Alkaloids from the Sponge *Hyrtios* species. *Org. Lett.* **2011**, *13*, 628–631, doi:10.1021/ol102867x.
437. Oda, Y.; Zhang, Q.; Matsunaga, S.; Fujita, M.J.; Sakai, R. Two New Mycosporine-like Amino Acids LC-343 and Mycosporine-Ethanolamine from the Micronesian Marine Sponge *Lendenfeldia chondrodes*. *Chem. Lett.* **2017**, *46*, 1272–1274, doi:10.1246/cl.170490.
438. Piao, S.-J.; Song, Y.-L.; Jiao, W.-H.; Yang, F.; Liu, X.-F.; Chen, W.-S.; Han, B.-N.; Lin, H.-W. Hippolachnin A, a New Antifungal Polyketide from the South China Sea Sponge *Hippospongia lachne*. *Org. Lett.* **2013**, *15*, 3526–3529, doi:10.1021/ol400933x.
439. Kaweetripob, W.; Mahidol, C.; Wongbundit, S.; Tuntiwachwuttikul, P.; Ruchirawat, S.; Prawat, H. Sesterterpenes and Phenolic Alkenes from the Thai Sponge *Hyrtios erectus*. *Tetrahedron* **2018**, *74*, 316–323, doi:10.1016/j.tet.2017.11.073.
440. Hawas, U.W.; Abou El-Kassem, L.T.; Abdelfattah, M.S.; Elmallah, M.I.Y.; Eid, M.A.G.; Monier, M.; Marimuthu, N. Cytotoxic Activity of Alkyl Benzoate and Fatty Acids from the Red Sea Sponge *Hyrtios erectus*. *Nat Prod Res* **2018**, *32*, 1369–1374, doi:10.1080/14786419.2017.1344662.
441. Kotoku, N.; Ishida, R.; Matsumoto, H.; Arai, M.; Toda, K.; Setiawan, A.; Muraoka, O.; Kobayashi, M. Biakamides A–D, Unique Polyketides from a Marine Sponge, Act as Selective Growth Inhibitors of Tumor Cells Adapted to Nutrient Starvation. *J. Org. Chem.* **2017**, *82*, 1705–1718, doi:10.1021/acs.joc.6b02948.
442. GOVINDAM, S.V.S.; CHOI, B.-K.; YOSHIOKA, Y.; KANAMOTO, A.; FUJIWARA, T.; OKAMOTO, T.; OJIKA, M. Novel Cytotoxic Polyoxygenated Steroids from an Okinawan Sponge *Dysidea* sp sp. *Bioscience, Biotechnology, and Biochemistry* **2012**, *76*, 999–1002, doi:10.1271/bbb.120017.
443. Yang, I.; Choi, H.; Won, D.H.; Nam, S.J.; Kang, H. An Antibacterial 9,11-Secosterol from a Marine Sponge *Ircinia* sp. *Bulletin of the Korean Chemical Society* **2014**, *35*, 3360–3362, doi:10.5012/bkcs.2014.35.11.3360.

444. Li, J.; Wang, Z.; Yang, F.; Jiao, W.-H.; Lin, H.-W.; Xu, S.-H. Two New Steroids with Cytotoxicity from the Marine Sponge *Dactylospongia elegans* Collected from the South China Sea. *Natural Product Research* **2019**, *33*, 1340–1344, doi:10.1080/14786419.2018.1475385.
445. Trinh, T.T.V.; Truong, B.N.; Longeon, A.; Doan, T.M.H.; Deville, A.; Chau, V.M.; Pham, V.C.; Bourguet-Kondracki, M.-L. New 9α-Hydroxy-5α,6α-Epoxyhydroxysterols from the Vietnamese Marine Sponge *Ircinia echinata*. *Mar Drugs* **2018**, *16*, E424, doi:10.3390/md16110424.
446. Yang, I.; Choi, H.; Nam, S.-J.; Kang, H. A New 9,11-Secosterol with a 1,4-Quinone from a Korean Marine Sponge *Ircinia* sp. *Arch Pharm Res* **2015**, *38*, 1970–1974, doi:10.1007/s12272-015-0620-9.
447. Murali Krishna Kumar, M.; Devilal Naik, J.; Satyavathi, K.; Ramana, H.; Raghuveer Varma, P.; Purna Nagasree, K.; Smitha, D.; Venkata Rao, D. Denigrins A–C: New Antitubercular 3,4-Diarylpyrrole Alkaloids from *Dendrilla nigra*. *Natural Product Research* **2014**, *28*, 888–894, doi:10.1080/14786419.2014.891112.
448. Jiao, W.-H.; Xu, T.-T.; Yu, H.-B.; Mu, F.-R.; Li, J.; Li, Y.-S.; Yang, F.; Han, B.-N.; Lin, H.-W. Dysidaminones A–M, Cytotoxic and NF-KB Inhibitory Sesquiterpene Aminoquinones from the South China Sea Sponge *Dysidea fragilis*. *RSC Adv.* **2014**, *4*, 9236–9246, doi:10.1039/C3RA47265E.
449. Jiao, W.-H.; Xu, T.-T.; Yu, H.-B.; Chen, G.-D.; Huang, X.-J.; Yang, F.; Li, Y.-S.; Han, B.-N.; Liu, X.-Y.; Lin, H.-W. Dysideanones A-C, Unusual Sesquiterpene Quinones from the South China Sea Sponge *Dysidea avara*. *J Nat Prod* **2014**, *77*, 346–350, doi:10.1021/np4009392.
450. Jiao, W.-H.; Li, J.; Liu, Q.; Xu, T.-T.; Shi, G.-H.; Yu, H.-B.; Yang, F.; Han, B.-N.; Li, M.; Lin, H.-W. Dysidinoid A, an Unusual Meroterpenoid with Anti-MRSA Activity from the South China Sea Sponge *Dysidea* sp. *Molecules* **2014**, *19*, 18025–18032, doi:10.3390/molecules191118025.
451. Piao, S.-J.; Jiao, W.-H.; Yang, F.; Yi, Y.-H.; Di, Y.-T.; Han, B.-N.; Lin, H.-W. New Hippolide Derivatives with Protein Tyrosine Phosphatase 1B Inhibitory Activity from the Marine Sponge *Hippospongia lachne*. *Marine Drugs* **2014**, *12*, 4096–4109, doi:10.3390/md12074096.
452. Daletos, G.; de Voogd, N.J.; Müller, W.E.G.; Wray, V.; Lin, W.; Feger, D.; Kubbutat, M.; Aly, A.H.; Proksch, P. Cytotoxic and Protein Kinase Inhibiting Nakijiquinones and Nakijiquinols from the Sponge *Dactylospongia metachromia*. *J Nat Prod* **2014**, *77*, 218–226, doi:10.1021/np400633m.
453. Festa, C.; Cassiano, C.; D'Auria, M.V.; Debitus, C.; Monti, M.C.; Marino, S.D. Scalarane Sesterterpenes from *Thorectidae* Sponges as Inhibitors of TDP-43 Nuclear Factor. *Org. Biomol. Chem.* **2014**, *12*, 8646–8655, doi:10.1039/C4OB01510J.
454. Johnson, T.A.; Sohn, J.; Inman, W.D.; Estee, S.A.; Loveridge, S.T.; Vervoort, H.C.; Tenney, K.; Liu, J.; Ang, K.K.-H.; Ratnam, J.; et al. Natural Product Libraries to Accelerate the High-Throughput Discovery of Therapeutic Leads. *J Nat Prod* **2011**, *74*, 2545–2555, doi:10.1021/np200673b.
455. Whitson, E.L.; Pluchino, K.M.; Hall, M.D.; McMahon, J.B.; McKee, T.C. New Candidaspongiolides, Tedanolide Analogues That Selectively Inhibit Melanoma Cell Growth. *Org Lett* **2011**, *13*, 3518–3521, doi:10.1021/ol201329p.
456. Diyabalanage, T.; Ratnayake, R.; Bokesch, H.R.; Ransom, T.T.; Henrich, C.J.; Beutler, J.A.; McMahon, J.B.; Gustafson, K.R. Flabelliferins A and B, Sesterterpenoids from the South Pacific Sponge *Carteriospongia flabellifera*. *J. Nat. Prod.* **2012**, *75*, 1490–1494, doi:10.1021/np3003518.

457. Gupta, P.; Sharma, U.; Schulz, T.C.; McLean, A.B.; Robins, A.J.; West, L.M. Bicyclic C21 Terpenoids from the Marine Sponge *Clathria compressa*. *J. Nat. Prod.* **2012**, *75*, 1223–1227, doi:10.1021/np300265p.
458. Bae, J.; Jeon, J.-E.; Lee, Y.-J.; Lee, H.-S.; Sim, C.J.; Oh, K.-B.; Shin, J. Sesterterpenes from the Tropical Sponge *Coscinoderma* sp. *J Nat Prod* **2011**, *74*, 1805–1811, doi:10.1021/np200492k.
459. Jeon, J.; Bae, J.; Lee, K.J.; Oh, K.-B.; Shin, J. Scalarane Sesterterpenes from the Sponge *Hyatella* sp. *J Nat Prod* **2011**, *74*, 847–851, doi:10.1021/np1006873.
460. Ovenden, S.P.B.; Nielson, J.L.; Liptrot, C.H.; Willis, R.H.; Tapiolas, D.M.; Wright, A.D.; Motti, C.A. Sesquiterpene Benzoxazoles and Sesquiterpene Quinones from the Marine Sponge *Dactylospongia elegans*. *J. Nat. Prod.* **2011**, *74*, 65–68, doi:10.1021/np100669p.
461. Du, L.; Zhou, Y.-D.; Nagle, D.G. Inducers of Hypoxic Response: Marine Sesquiterpene Quinones Activate HIF-1. *J. Nat. Prod.* **2013**, *76*, 1175–1181, doi:10.1021/np400320r.
462. Jiao, W.-H.; Huang, X.-J.; Yang, J.-S.; Yang, F.; Piao, S.-J.; Gao, H.; Li, J.; Ye, W.-C.; Yao, X.-S.; Chen, W.-S.; et al. Dysidavarones A–D, New Sesquiterpene Quinones from the Marine Sponge *Dysidea avara*. *Org. Lett.* **2012**, *14*, 202–205, doi:10.1021/ol202994c.
463. Hamed, A.N.E.-S.; Wätjen, W.; Schmitz, R.; Chovolou, Y.; Edrada-Ebel, R.; Youssef, D.T.A.; Kamel, M.S.; Proksch, P. A New Bioactive Sesquiterpenoid Quinone from the Mediterranean Sea Marine Sponge *Dysidea avara*. *Nat Prod Commun* **2013**, *8*, 289–292.
464. Utkina, N.K.; Denisenko, V.A.; Krasokhin, V.B. Sesquiterpenoid Aminoquinones from the Marine Sponge *Dysidea* sp. *J. Nat. Prod.* **2010**, *73*, 788–791, doi:10.1021/np1000285.
465. Utkina, N.K.; Denisenko, V.A.; Krasokhin, V.B. Diplopuupehenone, a New Unsymmetrical Puupehenone-Related Dimer from the Marine Sponge *Dysidea* sp. *Tetrahedron Letters* **2011**, *52*, 3765–3768, doi:10.1016/j.tetlet.2011.05.059.
466. Zhang, H.; Khalil, Z.G.; Capon, R.J. Fascioquinols A–F: Bioactive Meroterpenes from a Deep-Water Southern Australian Marine Sponge, *Fasciospongia* sp. *Tetrahedron* **2011**, *67*, 2591–2595, doi:10.1016/j.tet.2011.02.015.
467. Piao, S.-J.; Zhang, H.-J.; Lu, H.-Y.; Yang, F.; Jiao, W.-H.; Yi, Y.-H.; Chen, W.-S.; Lin, H.-W. Hippolides A–H, Acyclic Manoalide Derivatives from the Marine Sponge *Hippospongia lachne*. *J. Nat. Prod.* **2011**, *74*, 1248–1254, doi:10.1021/np200227s.
468. Chang, Y.-C.; Tseng, S.-W.; Liu, L.-L.; Chou, Y.; Ho, Y.-S.; Lu, M.-C.; Su, J.-H. Cytotoxic Sesterterpenoids from a Sponge *Hippospongia* sp. *Marine Drugs* **2012**, *10*, 987–997, doi:10.3390/md10050987.
469. Li, J.; Du, L.; Kelly, M.; Zhou, Y.-D.; Nagle, D.G. Structures and Potential Antitumor Activity of Sesterterpenes from the Marine Sponge *Hyrtios communis*. *J. Nat. Prod.* **2013**, *76*, 1492–1497, doi:10.1021/np400350k.

470. Su, J.-H.; Tseng, S.-W.; Lu, M.-C.; Liu, L.-L.; Chou, Y.; Sung, P.-J. Cytotoxic C21 and C22 Terpenoid-Derived Metabolites from the Sponge *Ircinia* sp. *J. Nat. Prod.* **2011**, *74*, 2005–2009, doi:10.1021/np2004209.
471. Hiromi Hirade; Nicole J. de Voogd; Toshimasa Suzuka; Junichi Tanaka Trunculins X and Y from an Okinawan Sponge *Sigmosceptrella* sp. *Tetrahedron* **2019**, *75*, 4620–4625, doi:10.1016/j.tet.2019.07.005.
472. Cao, F.; Wu, Z.-H.; Shao, C.-L.; Pang, S.; Liang, X.-Y.; Voogd, N.J. de; Wang, C.-Y. Cytotoxic Scalarane Sesterterpenoids from the South China Sea Sponge *Carteriospongia foliascens*. *Org. Biomol. Chem.* **2015**, *13*, 4016–4024, doi:10.1039/C4OB02532F.
473. Lee, J.-W.; Lee, H.-S.; Shin, J.; Kang, J.S.; Yun, J.; Shin, H.J.; Lee, J.S.; Lee, Y.-J. Suvanine Analogs from a *Coscinoderma* sp. Marine Sponge and Their Cytotoxicities against Human Cancer Cell Lines. *Arch Pharm Res* **2015**, *38*, 1005–1010, doi:10.1007/s12272-014-0479-1.
474. Yu, H.-B.; Gu, B.-B.; Wang, S.-P.; Cheng, C.-W.; Yang, F.; Lin, H.-W. New Diterpenoids from the Marine Sponge *Dactylospongia elegans*. *Tetrahedron* **2017**, *73*, 6657–6661, doi:10.1016/j.tet.2017.10.023.
475. Li, J.; Wu, W.; Yang, F.; Liu, L.; Wang, S.-P.; Jiao, W.-H.; Xu, S.-H.; Lin, H.-W. Popolohuanones G - I, Dimeric Sesquiterpene Quinones with IL-6 Inhibitory Activity from the Marine Sponge *Dactylospongia elegans*. *Chem Biodivers* **2018**, *15*, e1800078, doi:10.1002/cbdv.201800078.
476. Neupane, R.P.; Parrish, S.M.; Neupane, J.B.; Yoshida, W.Y.; Yip, M.L.R.; Turkson, J.; Harper, M.K.; Head, J.D.; Williams, P.G. Cytotoxic Sesquiterpenoid Quinones and Quinols, and an 11-Membered Heterocycle, Kauamide, from the Hawaiian Marine Sponge *Dactylospongia elegans*. *Mar Drugs* **2019**, *17*, E423, doi:10.3390/md17070423.
477. Balansa, W.; Mettal, U.; Wuisan, Z.G.; Plubrukarn, A.; Ijong, F.G.; Liu, Y.; Schäberle, T.F. A New Sesquiterpenoid Aminoquinone from an Indonesian Marine Sponge. *Mar Drugs* **2019**, *17*, E158, doi:10.3390/md17030158.
478. Bonneau, N.; Chen, G.; Lachkar, D.; Boufridi, A.; Gallard, J.-F.; Retailleau, P.; Petek, S.; Debitus, C.; Evanno, L.; Beniddir, M.A.; et al. An Unprecedented Blue Chromophore Found in Nature Using a “Chemistry First” and Molecular Networking Approach: Discovery of Dactylocyanines A–H. *Chemistry – A European Journal* **2017**, *23*, 14454–14461, doi:10.1002/chem.201702336.
479. Hagiwara, K.; Garcia Hernandez, J.E.; Harper, M.K.; Carroll, A.; Motti, C.A.; Awaya, J.; Nguyen, H.-Y.; Wright, A.D. Puupehenol, a Potent Antioxidant Antimicrobial Meroterpenoid from a Hawaiian Deep-Water *Dactylospongia* sp. Sponge. *J. Nat. Prod.* **2015**, *78*, 325–329, doi:10.1021/np500793g.
480. Li, J.; Yang, F.; Wang, Z.; Wu, W.; Liu, L.; Wang, S.-P.; Zhao, B.-X.; Jiao, W.-H.; Xu, S.-H.; Lin, H.-W. Unusual Anti-Inflammatory Meroterpenoids from the Marine Sponge *Dactylospongia* sp. *Org Biomol Chem* **2018**, *16*, 6773–6782, doi:10.1039/c8ob01580e.

481. von Salm, J.L.; Witowski, C.G.; Fleeman, R.M.; McClintock, J.B.; Amsler, C.D.; Shaw, L.N.; Baker, B.J. Darwinolide, a New Diterpene Scaffold That Inhibits Methicillin-Resistant *Staphylococcus aureus* Biofilm from the Antarctic Sponge Dendrilla Membranosa. *Org. Lett.* **2016**, *18*, 2596–2599, doi:10.1021/acs.orglett.6b00979.
482. Hayton, J.B.; Grant, G.D.; Carroll, A.R.; Hayton, J.B.; Grant, G.D.; Carroll, A.R. Three New Spongian Diterpenes from the Marine Sponge *Dendrilla rosea*. *Aust. J. Chem.* **2019**, *72*, 964–968, doi:10.1071/CH19299.
483. Barber, J.M.; Leahy, D.C.; Miller, J.H.; Northcote, P.T. Luakuliides A–C, Cytotoxic Labdane Diterpenes from a Tongan *Dictyoceratid* Sponge. *Tetrahedron Letters* **2015**, *56*, 6314–6318, doi:10.1016/j.tetlet.2015.06.013.
484. Jiao, W.-H.; Cheng, B.-H.; Chen, G.-D.; Shi, G.-H.; Li, J.; Hu, T.-Y.; Lin, H.-W. Dysiarenone, a Dimeric C21 Meroterpenoid with Inhibition of COX-2 Expression from the Marine Sponge *Dysidea arenaria*. *Org Lett* **2018**, *20*, 3092–3095, doi:10.1021/acs.orglett.8b01148.
485. Jiao, W.-H.; Xu, T.-T.; Gu, B.-B.; Shi, G.-H.; Zhu, Y.; Yang, F.; Han, B.-N.; Wang, S.-P.; Li, Y.-S.; Zhang, W.; et al. Bioactive Sesquiterpene Quinols and Quinones from the Marine Sponge *Dysidea avara*. *RSC Adv.* **2015**, *5*, 87730–87738, doi:10.1039/C5RA18876H.
486. Jiao, W.-H.; Li, J.; Wang, D.; Zhang, M.-M.; Liu, L.-Y.; Sun, F.; Li, J.-Y.; Capon, R.J.; Lin, H.-W. Cinerols, Nitrogenous Meroterpenoids from the Marine Sponge *Dysidea cinerea*. *J Nat Prod* **2019**, *82*, 2586–2593, doi:10.1021/acs.jnatprod.9b00471.
487. Jiao, W.-H.; Xu, T.-T.; Zhao, F.; Gao, H.; Shi, G.-H.; Wang, J.; Hong, L.-L.; Yu, H.-B.; Li, Y.-S.; Yang, F.; et al. Dysifragilones A–C, Unusual Sesquiterpene Aminoquinones and Inhibitors of NO Production from the South China Sea Sponge *Dysidea fragilis*. *European Journal of Organic Chemistry* **2015**, *2015*, 960–966, doi:10.1002/ejoc.201403487.
488. Jiao, W.-H.; Li, J.; Zhang, M.-M.; Cui, J.; Gui, Y.-H.; Zhang, Y.; Li, J.-Y.; Liu, K.-C.; Lin, H.-W. Frondoplysins A and B, Unprecedented Terpene-Alkaloid Bioconjugates from *Dysidea frondosa*. *Org Lett* **2019**, *21*, 6190–6193, doi:10.1021/acs.orglett.9b01754.
489. Kim, C.-K.; Woo, J.-K.; Kim, S.-H.; Cho, E.; Lee, Y.-J.; Lee, H.-S.; Sim, C.J.; Oh, D.-C.; Oh, K.-B.; Shin, J. Meroterpenoids from a Tropical *Dysidea* sp. Sponge. *J Nat Prod* **2015**, *78*, 2814–2821, doi:10.1021/acs.jnatprod.5b00867.
490. Abdjul, D.B.; Yamazaki, H.; Takahashi, O.; Kirikoshi, R.; Ukai, K.; Namikoshi, M. Sesquiterpene Hydroquinones with Protein Tyrosine Phosphatase 1B Inhibitory Activities from a *Dysidea* sp. Marine Sponge Collected in Okinawa. *J. Nat. Prod.* **2016**, *79*, 1842–1847, doi:10.1021/acs.jnatprod.6b00367.
491. Jiao, W.-H.; Shi, G.-H.; Xu, T.-T.; Chen, G.-D.; Gu, B.-B.; Wang, Z.; Peng, S.; Wang, S.-P.; Li, J.; Han, B.-N.; et al. Dysiherbols A–C and Dysideanone E, Cytotoxic and NF-KB Inhibitory Tetracyclic Meroterpenes from a *Dysidea* sp. Marine Sponge. *J. Nat. Prod.* **2016**, *79*, 406–411, doi:10.1021/acs.jnatprod.5b01079.
492. Jiao, W.-H.; Shi, G.-H.; Xu, T.-T.; Chen, G.-D.; Gu, B.-B.; Wang, Z.; Peng, S.; Wang, S.-P.; Li, J.; Han, B.-N.; et al. Dysiherbols A–C and Dysideanone E, Cytotoxic and NF-KB Inhibitory Tetracyclic Meroterpenes from a *Dysidea* sp. Marine Sponge. *J. Nat. Prod.* **2016**, *79*, 406–411, doi:10.1021/acs.jnatprod.5b01079.

493. Jiao, W.-H.; Cheng, B.-H.; Shi, G.-H.; Chen, G.-D.; Gu, B.-B.; Zhou, Y.-J.; Hong, L.-L.; Yang, F.; Liu, Z.-Q.; Qiu, S.-Q.; et al. Dysivillosins A–D, Unusual Anti-Allergic Meroterpenoids from the Marine Sponge *Dysidea villosa*. *Sci Rep* **2017**, *7*, 8947, doi:10.1038/s41598-017-04021-z.
494. Yamazaki, H.; Takahashi, O.; Kanno, S.-I.; Nakazawa, T.; Takahashi, S.; Ukai, K.; Sumilat, D.A.; Ishikawa, M.; Namikoshi, M. Absolute Structures and Bioactivities of Euryspongins and Eurydiene Obtained from the Marine Sponge *Euryspongia* sp. Collected at Iriomote Island. *Bioorg Med Chem* **2015**, *23*, 797–802, doi:10.1016/j.bmc.2014.12.049.
495. Jiao, W.-H.; Hong, L.-L.; Sun, J.-B.; Piao, S.-J.; Chen, G.-D.; Deng, H.; Wang, S.-P.; Yang, F.; Lin, H.-W. (±)-Hippolide J – A Pair of Unusual Antifungal Enantiomeric Sesterterpenoids from the Marine Sponge *Hippospongia lachne*. *European Journal of Organic Chemistry* **2017**, *2017*, 3421–3426, doi:10.1002/ejoc.201700248.
496. Su, C.; Su, H.; Liang, K.; Tsaif, S.; Su, J. A Norsesterterpene Peroxide from a Marine Sponge *Hippospongia* sp. *Nat Prod Commun* **2016**, *11*, 445–446.
497. Abdjul, D.B.; Yamazaki, H.; Takahashi, O.; Kirikoshi, R.; Mangindaan, R.E.P.; Namikoshi, M. Two New Protein Tyrosine Phosphatase 1B Inhibitors, Hyattellactones A and B, from the Indonesian Marine Sponge *Hyattella* sp. *Bioorganic & Medicinal Chemistry Letters* **2015**, *25*, 904–907, doi:10.1016/j.bmcl.2014.12.058.
498. Wahab, H.A.; Pham, N.B.; Muhammad, T.S.T.; Hooper, J.N.A.; Quinn, R.J. Merosesquiterpene Congeners from the Australian Sponge *Hyrtios digitatus* as Potential Drug Leads for Atherosclerosis Disease. *Marine Drugs* **2017**, *15*, 6, doi:10.3390/md15010006.
499. Kaweetripob, W.; Mahidol, C.; Tuntiwachwuttikul, P.; Ruchirawat, S.; Prawat, H. Cytotoxic Sesterterpenes from Thai Marine Sponge *Hyrtios erectus*. *Mar Drugs* **2018**, *16*, E474, doi:10.3390/md16120474.
500. Alahdal, A.M.; Asfour, H.Z.; Ahmed, S.A.; Noor, A.O.; Al-Abd, A.M.; Elfaky, M.A.; Elhady, S.S. Anti-Helicobacter, Antitubercular and Cytotoxic Activities of Scalaranes from the Red Sea Sponge *Hyrtios erectus*. *Molecules* **2018**, *23*, 978, doi:10.3390/molecules23040978.
501. Wang, J.; Mu, F.-R.; Jiao, W.-H.; Huang, J.; Hong, L.-L.; Yang, F.; Xu, Y.; Wang, S.-P.; Sun, F.; Lin, H.-W. Meroterpenoids with Protein Tyrosine Phosphatase 1B Inhibitory Activity from a *Hyrtios* sp. Marine Sponge. *J. Nat. Prod.* **2017**, *80*, 2509–2514, doi:10.1021/acs.jnatprod.7b00435.
502. Lai, Y.-Y.; Lu, M.-C.; Wang, L.-H.; Chen, J.-J.; Fang, L.-S.; Wu, Y.-C.; Sung, P.-J. New Scalarane Sesterterpenoids from the Formosan Sponge *Ircinia felix*. *Marine Drugs* **2015**, *13*, 4296–4309, doi:10.3390/md13074296.
503. Lai, Y.-Y.; Chen, L.-C.; Wu, C.-F.; Lu, M.-C.; Wen, Z.-H.; Wu, T.-Y.; Fang, L.-S.; Wang, L.-H.; Wu, Y.-C.; Sung, P.-J. New Cytotoxic 24-Homoscalarane Sesterterpenoids from the Sponge *Ircinia felix*. *International Journal of Molecular Sciences* **2015**, *16*, 21950–21958, doi:10.3390/ijms160921950.

504. Kapojos, M.M.; Abdjul, D.B.; Yamazaki, H.; Kirikoshi, R.; Takahashi, O.; Rotinsulu, H.; Wewengkang, D.S.; Sumilat, D.A.; Ukai, K.; Namikoshi, M. Protein Tyrosine Phosphatase 1B Inhibitory Polybromobiphenyl Ethers and Monocyclofarnesol-Type Sesquiterpenes from the Indonesian Marine Sponge *Lamellodysidea* cf. *herbacea*. *Phytochemistry Letters* **2018**, *24*, 10–14, doi:10.1016/j.phytol.2017.11.016.
505. Su, T.-R.; Liao, Z.-J.; Lu, M.-C.; Wu, Y.-J.; Su, J.-H. Cytotoxic Monocarbocyclic Sesterterpenoids from a Marine Sponge *Luffariella* sp. *BCSJ* **2015**, *88*, 176–182, doi:10.1246/bcsj.20140251.
506. Hahn, D.; Chin, J.; Kim, H.; Yang, I.; Won, D.H.; Ekins, M.; Choi, H.; Nam, S.-J.; Kang, H. Sesquiterpenoids with PPARδ Agonistic Effect from a Korean Marine Sponge *Ircinia* sp. *Tetrahedron Letters* **2014**, *55*, 4716–4719, doi:10.1016/j.tetlet.2014.07.019.
507. Mudianta, I.W.; Skinner-Adams, T.; Andrews, K.T.; Davis, R.A.; Hadi, T.A.; Hayes, P.Y.; Garson, M.J. Psammaplysin Derivatives from the Balinese Marine Sponge *Aplysinella strongylata*. *J. Nat. Prod.* **2012**, *75*, 2132–2143, doi:10.1021/np300560b.
508. Carroll, A.R.; Kaiser, S.M.; Davis, R.A.; Moni, R.W.; Hooper, J.N.A.; Quinn, R.J. A Bastadin with Potent and Selective δ-Opioid Receptor Binding Affinity from the Australian Sponge *Ianthella flabelliformis*. *J. Nat. Prod.* **2010**, *73*, 1173–1176, doi:10.1021/np100010z.
509. Feng, Y.; Bowden, B.F.; Kapoor, V. Ianthellamide A, a Selective Kynurenine-3-Hydroxylase Inhibitor from the Australian Marine Sponge *Ianthella quadrangulata*. *Bioorganic & Medicinal Chemistry Letters* **2012**, *22*, 3398–3401, doi:10.1016/j.bmcl.2012.04.002.
510. Calcul, L.; Inman, W.D.; Morris, A.A.; Tenney, K.; Ratnam, J.; McKerrow, J.H.; Valeriote, F.A.; Crews, P. Additional Insights on the Bastadins: Isolation of Analogues from the Sponge *Ianthella* cf. *reticulata* and Exploration of the Oxime Configurations. *J. Nat. Prod.* **2010**, *73*, 365–372, doi:10.1021/np9005986.
511. Zhang, H.; Conte, M.M.; Huang, X.-C.; Khalil, Z.; Capon, R.J. A Search for BACE Inhibitors Reveals New Biosynthetically Related Pyrrolidones, Furanones and Pyrroles from a Southern Australian Marine Sponge, *Ianthella* sp. *Org. Biomol. Chem.* **2012**, *10*, 2656–2663, doi:10.1039/C2OB06747A.
512. Zhang, H.; Conte, M.M.; Khalil, Z.; Huang, X.-C.; Capon, R.J. New Dictyodendrins as BACE Inhibitors from a Southern Australian Marine Sponge, *Ianthella* sp. *RSC Adv.* **2012**, *2*, 4209–4214, doi:10.1039/C2RA20322G.
513. Shaala, L.A.; Youssef, D.T.A.; Sulaiman, M.; Behery, F.A.; Foudah, A.I.; Sayed, K.A.E. Subereamolline A as a Potent Breast Cancer Migration, Invasion and Proliferation Inhibitor and Bioactive Dibrominated Alkaloids from the Red Sea Sponge *Pseudoceratina arabica*. *Marine Drugs* **2012**, *10*, 2492–2508, doi:10.3390/md10112492.
514. Kon, Y.; Kubota, T.; Shibazaki, A.; Gonoi, T.; Kobayashi, J. Ceratinadins A-C, New Bromotyrosine Alkaloids from an Okinawan Marine Sponge *Pseudoceratina* sp. *Bioorg Med Chem Lett* **2010**, *20*, 4569–4572, doi:10.1016/j.bmcl.2010.06.015.
515. Xu, M.; Andrews, K.T.; Birrell, G.W.; Tran, T.L.; Camp, D.; Davis, R.A.; Quinn, R.J. Psammaplysin H, a New Antimalarial Bromotyrosine Alkaloid from a Marine Sponge of the Genus *Pseudoceratina*. *Bioorganic & Medicinal Chemistry Letters* **2011**, *21*, 846–848, doi:10.1016/j.bmcl.2010.11.081.

516. Tran, T.D.; Pham, N.B.; Fechner, G.; Hooper, J.N.A.; Quinn, R.J. Bromotyrosine Alkaloids from the Australian Marine Sponge *Pseudoceratina verrucosa*. *J Nat Prod* **2013**, *76*, 516–523, doi:10.1021/np300648d.
517. Göthel, Q.; Sirirak, T.; Köck, M. Bromotyrosine-Derived Alkaloids from the Caribbean Sponge *Aplysina lacunosa*. *Beilstein J. Org. Chem.* **2015**, *11*, 2334–2342, doi:10.3762/bjoc.11.254.
518. Dai, J.; Parrish, S.M.; Yoshida, W.Y.; Yip, M.L.R.; Turkson, J.; Kelly, M.; Williams, P. Bromotyrosine-Derived Metabolites from an Indonesian Marine Sponge in the Family Aplysinellidae (Order Verongiida). *Bioorganic & Medicinal Chemistry Letters* **2016**, *26*, 499–504, doi:10.1016/j.bmcl.2015.11.086.
519. Shaala, L.A.; Youssef, D.T.A. Cytotoxic Psammaplysin Analogues from the Verongid Red Sea Sponge *Aplysinella* Species. *Biomolecules* **2019**, *9*, E841, doi:10.3390/biom9120841.
520. Shaala, L.A.; Youssef, D.T.A.; Badr, J.M.; Sulaiman, M.; Khedr, A.; El Sayed, K.A. Bioactive Alkaloids from the Red Sea Marine Verongid Sponge *Pseudoceratina arabica*. *Tetrahedron* **2015**, *71*, 7837–7841, doi:10.1016/j.tet.2015.08.024.
521. Gotsbacher, M.P.; Karuso, P. New Antimicrobial Bromotyrosine Analogues from the Sponge Pseudoceratina Purpurea and Its Predator *Tylodina corticalis*. *Marine Drugs* **2015**, *13*, 1389–1409, doi:10.3390/md13031389.
522. Ragini, K.; Fromont, J.; Piggott, A.M.; Karuso, P. Enantiodivergence in the Biosynthesis of Bromotyrosine Alkaloids from Sponges? *J. Nat. Prod.* **2017**, *80*, 215–219, doi:10.1021/acs.jnatprod.6b01038.
523. Kurimoto, S.-I.; Ohno, T.; Hokari, R.; Ishiyama, A.; Iwatsuki, M.; Ōmura, S.; Kobayashi, J.; Kubota, T. Ceratinadins E and F, New Bromotyrosine Alkaloids from an Okinawan Marine Sponge *Pseudoceratina* sp. *Mar Drugs* **2018**, *16*, E463, doi:10.3390/md16120463.
524. Yin, S.; Davis, R.A.; Shelper, T.; Sykes, M.L.; Avery, V.M.; Elofsson, M.; Sundin, C.; Quinn, R.J. Pseudoceramines A–D, New Antibacterial Bromotyrosine Alkaloids from the Marine Sponge *Pseudoceratina* sp. *Org. Biomol. Chem.* **2011**, *9*, 6755–6760, doi:10.1039/C1OB05581J.
525. Tian, L.-W.; Feng, Y.; Shimizu, Y.; Pfeifer, T.; Wellington, C.; Hooper, J.N.A.; Quinn, R.J. Aplysinellamides A-C, Bromotyrosine-Derived Metabolites from an Australian *Aplysinella* sp. Marine Sponge. *J Nat Prod* **2014**, *77*, 1210–1214, doi:10.1021/np500119e.
526. Niemann, H.; Lin, W.; Müller, W.E.G.; Kubbutat, M.; Lai, D.; Proksch, P. Trimeric Hemibastadin Congener from the Marine Sponge *Ianthella basta*. *J. Nat. Prod.* **2013**, *76*, 121–125, doi:10.1021/np300764u.
527. Davis, R.A.; Duffy, S.; Fletcher, S.; Avery, V.M.; Quinn, R.J. Thiaplakortones A–D: Antimalarial Thiazine Alkaloids from the Australian Marine Sponge *Plakortis lita*. *J. Org. Chem.* **2013**, *78*, 9608–9613, doi:10.1021/jo400988y.
528. Kobayashi, J.; Kubota, T.; Ishiguro, Y.; Yamamoto, S.; Fromont, J. Platisidines A-C, N-Methylpyridinium Alkaloids from an Okinawan Marine Sponge of *Plakortis* Species. *Heterocycles* **2010**, *80*, 1407, doi:10.3987/COM-09-S(S)131.

529. Hanif, N.; Yamada, K.; Kitamura, M.; Kawazoe, Y.; de Voogd, N.J.; Uemura, D. New Indole Alkaloids from the Sponge *Plakortis* sp. *Chem Nat Compd* **2015**, *51*, 1130–1133, doi:10.1007/s10600-015-1508-0.
530. Lu, Z.; Koch, M.; Harper, M.K.; Matainaho, T.K.; Barrows, L.R.; Van Wagoner, R.M.; Ireland, C.M. Plakinamine M, a Steroidal Alkaloid from the Marine Sponge *Corticium* sp. *J. Nat. Prod.* **2013**, *76*, 2150–2152, doi:10.1021/np400649e.
531. Katavic, P.L.; Yong, K.W.L.; Herring, J.N.; Deseo, M.A.; Blanchfield, J.T.; Ferro, V.; Garson, M.J. Structure and Stereochemistry of an Anti-Inflammatory Anhydrosugar from the Australian Marine Sponge *Plakinastrella clathrata* and the Synthesis of Two Analogues. *Tetrahedron* **2013**, *69*, 8074–8079, doi:10.1016/j.tet.2013.06.079.
532. Mohammed, R.; Peng, J.; Kelly, M.; Yousaf, M.; Winn, E.; Odde, S.; Bie, Z.; Xie, A.; Doerksen, R.J.; Hamann, M.T.; et al. Polyketide-Peroxides from a Species of Jamaican *Plakortis* (Porifera: Demospongiae). *Aust. J. Chem.* **2010**, *63*, 877–885, doi:10.1071/CH09665.
533. Ankisetty, S.; Gochfeld, D.J.; Diaz, M.C.; Khan, S.I.; Slattery, M. Chemical Constituents of the Deep Reef Caribbean Sponges Plakortis Angulospiculatus and *Plakortis halichondrioides* and Their Anti-Inflammatory Activities. *J. Nat. Prod.* **2010**, *73*, 1494–1498, doi:10.1021/np100233d.
534. Chianese, G.; Scala, F.; Calcinai, B.; Cerrano, C.; Dien, H.A.; Kaiser, M.; Tasdemir, D.; Taglialatela-Scafati, O. Natural and Semisynthetic Analogues of Manadoperoxide B Reveal New Structural Requirements for Trypanocidal Activity. *Marine Drugs* **2013**, *11*, 3297–3308, doi:10.3390/md11093297.
535. Fattorusso, C.; Persico, M.; Calcinai, B.; Cerrano, C.; Parapini, S.; Taramelli, D.; Novellino, E.; Romano, A.; Scala, F.; Fattorusso, E.; et al. Manadoperoxides A−D from the Indonesian Sponge *Plakortis* cfr. *simplex*. Further Insights on the Structure−Activity Relationships of Simple 1,2-Dioxane Antimalarials. *J. Nat. Prod.* **2010**, *73*, 1138–1145, doi:10.1021/np100196b.
536. Oh, J.S.; Hwang, B.S.; Kang, O.-H.; Kwon, D.-Y.; Rho, J.-R. New Constituents from the Korean Sponge *Plakortis simplex*. *Marine Drugs* **2013**, *11*, 4407–4418, doi:10.3390/md11114407.
537. Tanaka, N.; Asai, M.; Takahashi-Nakaguchi, A.; Gonoi, T.; Fromont, J.; Kobayashi, J. Manzamenone O, New Trimeric Fatty Acid Derivative from a Marine Sponge *Plakortis* sp. *Org. Lett.* **2013**, *15*, 2518–2521, doi:10.1021/ol4009975.
538. Sunassee, S.N.; Ransom, T.; Henrich, C.J.; Beutler, J.A.; Covell, D.G.; McMahon, J.B.; Gustafson, K.R. Steroidal Alkaloids from the Marine Sponge *Corticium niger* That Inhibit Growth of Human Colon Carcinoma Cells. *J. Nat. Prod.* **2014**, *77*, 2475–2480, doi:10.1021/np500556t.
539. Rodrigues Felix, C.; Roberts, J.C.; Winder, P.L.; Gupta, R.; Diaz, M.C.; Pomponi, S.A.; Wright, A.E.; Rohde, K.H. Plakinamine P, A Steroidal Alkaloid with Bactericidal Activity against *Mycobacterium tuberculosis*. *Mar Drugs* **2019**, *17*, E707, doi:10.3390/md17120707.
540. Kwon, I.-S.; Kwak, J.H.; Pyo, S.; Lee, H.-W.; Kim, A.; Schmitz, F.J. Oscarellin, an Anthranilic Acid Derivative from a Philippine Sponge, *Oscarella stillans*, as an Inhibitor of Inflammatory Cytokines in Macrophages. *J. Nat. Prod.* **2017**, *80*, 149–155, doi:10.1021/acs.jnatprod.6b00787.

541. Festa, C.; De Marino, S.; D'Auria, M.V.; Taglialatela-Scafati, O.; Deharo, E.; Petek, S.; Zampella, A. New Antimalarial Polyketide Endoperoxides from the Marine Sponge *Plakinastrella mamillaris* Collected at Fiji Islands. *Tetrahedron* **2013**, *69*, 3706–3713, doi:10.1016/j.tet.2013.03.006.
542. Liu, X.-F.; Song, Y.-L.; Zhang, H.-J.; Yang, F.; Yu, H.-B.; Jiao, W.-H.; Piao, S.-J.; Chen, W.-S.; Lin, H.-W. Simplextones A and B, Unusual Polyketides from the Marine Sponge *Plakortis simplex*. *Org. Lett.* **2011**, *13*, 3154–3157, doi:10.1021/ol201055w.
543. Zhang, J.; Tang, X.; Li, J.; Li, P.; de Voogd, N.J.; Ni, X.; Jin, X.; Yao, X.; Li, P.; Li, G. Cytotoxic Polyketide Derivatives from the South China Sea Sponge *Plakortis simplex*. *J. Nat. Prod.* **2013**, *76*, 600–606, doi:10.1021/np300771p.
544. Kubota, T.; Ishiguro, Y.; Takahashi-Nakaguchi, A.; Fromont, J.; Gonoi, T.; Kobayashi, J. Manzamenones L–N, New Dimeric Fatty-Acid Derivatives from an Okinawan Marine Sponge *Plakortis* sp. *Bioorganic & Medicinal Chemistry Letters* **2013**, *23*, 244–247, doi:10.1016/j.bmcl.2012.10.109.
545. Chianese, G.; Persico, M.; Yang, F.; Lin, H.-W.; Guo, Y.-W.; Basilico, N.; Parapini, S.; Taramelli, D.; Taglialatela-Scafati, O.; Fattorusso, C. Endoperoxide Polyketides from a Chinese *Plakortis simplex*: Further Evidence of the Impact of Stereochemistry on Antimalarial Activity of Simple 1,2-Dioxanes. *Bioorg Med Chem* **2014**, *22*, 4572–4580, doi:10.1016/j.bmc.2014.07.034.
546. Gushiken, M.; Kagiyama, I.; Kato, H.; Kuwana, T.; Losung, F.; Mangindaan, R.E.P.; de Voogd, N.J.; Tsukamoto, S. Manadodioxans A-E: Polyketide Endoperoxides from the Marine Sponge *Plakortis bergquistae*. *J Nat Med* **2015**, *69*, 595–600, doi:10.1007/s11418-015-0920-x.
547. Jiménez-Romero, C.; Rodríguez, A.D.; Nam, S. Plakortinic Acids A and B: Cytotoxic Cycloperoxides with a Bicyclo[4.2.0]Octene Unit from Sponges of the Genera *Plakortis* and *Xestospongia*. *Org. Lett.* **2017**, *19*, 1486–1489, doi:10.1021/acs.orglett.7b00547.
548. Jiménez-Romero, C.; Rode, J.E.; Pérez, Y.M.; Franzblau, S.G.; Rodríguez, A.D. Exploring the Sponge Consortium *Plakortis symbiotica–Xestospongia deweerdtae* as a Potential Source of Antimicrobial Compounds and Probing the Pharmacophore for Antituberculosis Activity of Smenothiazole A by Diverted Total Synthesis. *J. Nat. Prod.* **2017**, *80*, 2295–2303, doi:10.1021/acs.jnatprod.7b00300.
549. Chianese, G.; Yu, H.-B.; Yang, F.; Sirignano, C.; Luciano, P.; Han, B.-N.; Khan, S.; Lin, H.-W.; Taglialatela-Scafati, O. PPAR Modulating Polyketides from a Chinese *Plakortis simplex* and Clues on the Origin of Their Chemodiversity. *J. Org. Chem.* **2016**, *81*, 5135–5143, doi:10.1021/acs.joc.6b00695.
550. Chianese, G.; Sepe, V.; Limongelli, V.; Renga, B.; D'Amore, C.; Zampella, A.; Taglialatela-Scafati, O.; Fiorucci, S. Incisterols, Highly Degraded Marine Sterols, Are a New Chemotype of PXR Agonists. *Steroids* **2014**, *83*, 80–85, doi:10.1016/j.steroids.2014.02.003.
551. Festa, C.; Lauro, G.; De Marino, S.; D'Auria, M.V.; Monti, M.C.; Casapullo, A.; D'Amore, C.; Renga, B.; Mencarelli, A.; Petek, S.; et al. Plakilactones from the Marine Sponge *Plakinastrella mamillaris*. Discovery of a New Class of Marine Ligands of Peroxisome Proliferator-Activated Receptor γ. *J. Med. Chem.* **2012**, *55*, 8303–8317, doi:10.1021/jm300911g.

552. Festa, C.; De Marino, S.; D'Auria, M.V.; Deharo, E.; Gonzalez, G.; Deyssard, C.; Petek, S.; Bifulco, G.; Zampella, A. Gracilioethers E–J, New Oxygenated Polyketides from the Marine Sponge *Plakinastrella mamillaris*. *Tetrahedron* **2012**, *68*, 10157–10163, doi:10.1016/j.tet.2012.09.106.
553. Chianese, G.; Fattorusso, E.; Scala, F.; Teta, R.; Calcinai, B.; Bavestrello, G.; Dien, H.A.; Kaiser, M.; Tasdemir, D.; Taglialatela-Scafati, O. Manadoperoxides, a New Class of Potent Antitrypanosomal Agents of Marine Origin. *Org. Biomol. Chem.* **2012**, *10*, 7197–7207, doi:10.1039/C2OB26124C.
554. Feng, Y.; Davis, R.A.; Sykes, M.; Avery, V.M.; Camp, D.; Quinn, R.J. Antitrypanosomal Cyclic Polyketide Peroxides from the Australian Marine Sponge *Plakortis* sp. *J. Nat. Prod.* **2010**, *73*, 716–719, doi:10.1021/np900535z.
555. Yu, H.-B.; Liu, X.-F.; Xu, Y.; Gan, J.-H.; Jiao, W.-H.; Shen, Y.; Lin, H.-W. Woodylides A–C, New Cytotoxic Linear Polyketides from the South China Sea Sponge *Plakortis simplex*. *Marine Drugs* **2012**, *10*, 1027–1036, doi:10.3390/md10051027.
556. Liu, X.-F.; Shen, Y.; Yang, F.; Hamann, M.T.; Jiao, W.-H.; Zhang, H.-J.; Chen, W.-S.; Lin, H.-W. Simplexolides A–E and Plakorfuran A, Six Butyrate Derived Polyketides from the Marine Sponge *Plakortis simplex*. *Tetrahedron* **2012**, *68*, 4635–4640, doi:10.1016/j.tet.2012.04.025.
557. Barber, J.M.; Quek, N.C.H.; Leahy, D.C.; Miller, J.H.; Bellows, D.S.; Northcote, P.T. Lehualides E-K, Cytotoxic Metabolites from the Tongan Marine Sponge *Plakortis* sp. *J Nat Prod* **2011**, *74*, 809–815, doi:10.1021/np100868t.
558. Santos, E.A.; Quintela, A.L.; Ferreira, E.G.; Sousa, T.S.; Pinto, F. das C.L.; Hajdu, E.; Carvalho, M.S.; Salani, S.; Rocha, D.D.; Wilke, D.V.; et al. Cytotoxic Plakortides from the Brazilian Marine Sponge *Plakortis angulospiculatus*. *J. Nat. Prod.* **2015**, *78*, 996–1004, doi:10.1021/np5008944.
559. Hoye, T.R.; Alarif, W.M.; Basaif, S.S.; Abo-Elkarm, M.; Hamann, M.T.; Wahba, A.E.; Ayyad, S.-E.N. New Cytotoxic Cyclic Peroxide Acids from *Plakortis* sp. Marine Sponge. *Arkivoc* **2015**, *2015*, 164–175, doi:10.3998/ark.5550190.p008.948.
560. Hwang, I.H.; Oh, J.; Kochanowska-Karamyan, A.; Doerksen, R.J.; Na, M.; Hamann, M.T. A Novel Natural Phenyl Alkene with Cytotoxic Activity. *Tetrahedron Letters* **2013**, *54*, 3872–3876, doi:10.1016/j.tetlet.2013.05.032.
561. Uddin, M.H.; Hossain, M.K.; Nigar, M.; Roy, M.C.; Tanaka, J. New Cytotoxic Spongian-Class Rearranged Diterpenes from a Marine Sponge. *Chem Nat Compd* **2012**, *48*, 412–415, doi:10.1007/s10600-012-0262-9.
562. Cen-Pacheco, F.; Norte, M.; Fernández, J.J.; Daranas, A.H. Zoaramine, a Zoanthamine-like Alkaloid with a New Skeleton. *Org. Lett.* **2014**, *16*, 2880–2883, doi:10.1021/ol500860v.
563. Cheng, Y.-B.; Lo, I.-W.; Shyur, L.-F.; Yang, C.-C.; Hsu, Y.-M.; Su, J.-H.; Lu, M.-C.; Chiou, S.-F.; Lan, C.-C.; Wu, Y.-C.; et al. New Alkaloids from Formosan Zoanthid *Zoanthus kuroshio*. *Tetrahedron* **2015**, *71*, 8601–8606, doi:10.1016/j.tet.2015.09.023.
564. Hsu, Y.-M.; Chang, F.-R.; Lo, I.-W.; Lai, K.-H.; El-Shazly, M.; Wu, T.-Y.; Du, Y.-C.; Hwang, T.-L.; Cheng, Y.-B.; Wu, Y.-C. Zoanthamine-Type Alkaloids from the Zoanthid *Zoanthus kuroshio* Collected in Taiwan and Their Effects on Inflammation. *J. Nat. Prod.* **2016**, *79*, 2674–2680, doi:10.1021/acs.jnatprod.6b00625.

565. Chen, S.-R.; Wang, S.-W.; Su, C.-J.; Hu, H.-C.; Yang, Y.-L.; Hsieh, C.-T.; Peng, C.-C.; Chang, F.-R.; Cheng, Y.-B. Anti-Lymphangiogenesis Components from Zoanthid *Palythoa tuberculosa*. *Marine Drugs* **2018**, *16*, 47, doi:10.3390/md16020047.

566. Guillen, P.; Gegunde, S.; Jaramillo, K.; Alfonso, A.; Calabro, K.; Alonso, E.; Rodriguez, J.; Botana, L.; Thomas, O. Zoanthamine Alkaloids from the Zoantharian *Zoanthus* cf. *pulchellus* and Their Effects in Neuroinflammation. *Marine Drugs* **2018**, *16*, 242, doi:10.3390/md16070242.

567. Chen, S.-R.; Wang, S.-W.; Chang, F.-R.; Cheng, Y.-B. Anti-Lymphangiogenic Alkaloids from the Zoanthid *Zoanthus vietnamensis* Collected in Taiwan. *J. Nat. Prod.* **2019**, *82*, 2790–2799, doi:10.1021/acs.jnatprod.9b00451.

568. Guillen, P.O.; Jaramillo, K.B.; Jennings, L.; Genta-Jouve, G.; de la Cruz, M.; Cautain, B.; Reyes, F.; Rodríguez, J.; Thomas, O.P. Halogenated Tyrosine Derivatives from the Tropical Eastern Pacific Zoantharians *Antipathozoanthus Hickmani* and *Parazoanthus darwini*. *J. Nat. Prod.* **2019**, *82*, 1354–1360, doi:10.1021/acs.jnatprod.9b00173.

569. Al-Lihaibi, S.S.; Ayyad, S.-E.N.; Shaher, F.; Alarif, W.M. Antibacterial Sphingolipid and Steroids from the Black Coral *Antipathes dichotoma*. *Chem. Pharm. Bull.* **2010**, *58*, 1635–1638, doi:10.1248/cpb.58.1635.

570. Kim, C.-H.; Lee, Y.J.; Go, H.-J.; Oh, H.Y.; Lee, T.K.; Park, J.B.; Park, N.G. Defensin-Neurotoxin Dyad in a Basally Branching Metazoan Sea Anemone. *FEBS J* **2017**, *284*, 3320–3338, doi:10.1111/febs.14194.

571. Moreels, L.; Peigneur, S.; Galan, D.; De Pauw, E.; Béress, L.; Waelkens, E.; Pardo, L.; Quinton, L.; Tytgat, J. APETx4, a Novel Sea Anemone Toxin and a Modulator of the Cancer-Relevant Potassium Channel KV10.1. *Marine Drugs* **2017**, *15*, 287, doi:10.3390/md15090287.

572. Sintsova, O.V.; Pislyagin, E.A.; Gladkikh, I.N.; Monastyrnaya, M.M.; Menchinskaya, E.S.; Leychenko, E.V.; Aminin, D.L.; Kozlovskaya, E.P. Kunitz-Type Peptides of the Sea Anemone *Heteractis crispa*: Potential Anti-Inflammatory Compounds. *Russ J Bioorg Chem* **2017**, *43*, 91–97, doi:10.1134/S1068162016060121.

573. Logashina, Y.A.; Mosharova, I.V.; Korolkova, Y.V.; Shelukhina, I.V.; Dyachenko, I.A.; Palikov, V.A.; Palikova, Y.A.; Murashev, A.N.; Kozlov, S.A.; Stensvåg, K.; et al. Peptide from Sea Anemone *Metridium senile* Affects Transient Receptor Potential Ankyrin-Repeat 1 (TRPA1) Function and Produces Analgesic Effect. *Journal of Biological Chemistry* **2017**, *292*, 2992–3004, doi:10.1074/jbc.M116.757369.

574. Kalina, R.; Gladkikh, I.; Dmitrenok, P.; Chernikov, O.; Koshelev, S.; Kvetkina, A.; Kozlov, S.; Kozlovskaya, E.; Monastyrnaya, M. New APETx-like Peptides from Sea Anemone *Heteractis crispa* Modulate ASIC1a Channels. *Peptides* **2018**, *104*, 41–49, doi:10.1016/j.peptides.2018.04.013.

575. Wu, Z.-Z.; Ding, G.-F.; Huang, F.-F.; Yang, Z.-S.; Yu, F.-M.; Tang, Y.-P.; Jia, Y.-L.; Zheng, Y.-Y.; Chen, R. Anticancer Activity of *Anthopleura anjunae* Oligopeptides in Prostate Cancer DU-145 Cells. *Marine Drugs* **2018**, *16*, 125, doi:10.3390/md16040125.

576. Madio, B.; Peigneur, S.; Chin, Y.K.Y.; Hamilton, B.R.; Henriques, S.T.; Smith, J.J.; Cristofori-Armstrong, B.; Dekan, Z.; Boughton, B.A.; Alewood, P.F.; et al. PHAB Toxins: A Unique Family of Predatory Sea Anemone Toxins Evolving via Intra-Gene Concerted Evolution Defines a New Peptide Fold. *Cell. Mol. Life Sci.* **2018**, *75*, 4511–4524, doi:10.1007/s00018-018-2897-6.

577. Sintsova, O.; Gladkikh, I.; Kalinovskii, A.; Zelepuga, E.; Monastyrnaya, M.; Kim, N.; Shevchenko, L.; Peigneur, S.; Tytgat, J.; Kozlovskaya, E.; et al. Magnificamide, a β-Defensin-Like Peptide from the Mucus of the Sea Anemone *Heteractis magnifica*, Is a Strong Inhibitor of Mammalian α-Amylases. *Marine Drugs* **2019**, *17*, 542, doi:10.3390/md17100542.

578. Kodani, S.; Sato, K.; Higuchi, T.; Casareto, B.E.; Suzuki, Y. Montiporic Acid D, a New Polyacetylene Carboxylic Acid from Scleractinian Coral *Montipora digitata*. *Natural Product Research* **2013**, *27*, 1859–1862, doi:10.1080/14786419.2013.768992.

579. Soto, C.; del Valle, A.; Valiente, P.A.; Ros, U.; Lanio, M.E.; Hernández, A.M.; Alvarez, C. Differential Binding and Activity of the Pore-Forming Toxin Sticholysin II in Model Membranes Containing Diverse Ceramide-Derived Lipids. *Biochimie* **2017**, *138*, 20–31, doi:10.1016/j.biochi.2017.04.003.

580. Rivera-de-Torre, E.; Martínez-del-Pozo, Á.; Garb, J.E. Stichodactyla Helianthus' de Novo Transcriptome Assembly: Discovery of a New Actinoporin Isoform. *Toxicon* **2018**, *150*, 105–114, doi:10.1016/j.toxicon.2018.05.014.

581. Ben-Ari, H.; Paz, M.; Sher, D. The Chemical Armament of Reef-Building Corals: Inter- and Intra-Specific Variation and the Identification of an Unusual Actinoporin in *Stylophora pistilata*. *Sci Rep* **2018**, *8*, 251, doi:10.1038/s41598-017-18355-1.

582. Yu, S.; Ye, X.; Chen, L.; Lian, X.-Y.; Zhang, Z. Polyoxygenated 24,28-Epoxyergosterols Inhibiting the Proliferation of Glioma Cells from Sea Anemone *Anthopleura midori*. *Steroids* **2014**, *88*, 19–25, doi:10.1016/j.steroids.2014.06.013.

583. Elbagory, A.M.; Meyer, M.; Ali, A.-H.A.M.; Ameer, F.; Parker-Nance, S.; Benito, M.T.; Doyagüez, E.G.; Jimeno, M.L.; Hussein, A.A. New Polyhydroxylated Sterols from *Palythoa tuberculosa* and Their Apoptotic Activity in Cancer Cells. *Steroids* **2015**, *101*, 110–115, doi:10.1016/j.steroids.2015.06.009.

584. Cheng, Y.-B.; Lee, J.-C.; Lo, I.-W.; Chen, S.-R.; Hu, H.-C.; Wu, Y.-H.; Wu, Y.-C.; Chang, F.-R. Ecdysones from *Zoanthus* spp. with Inhibitory Activity against *Dengue* Virus 2. *Bioorganic & Medicinal Chemistry Letters* **2016**, *26*, 2344–2348, doi:10.1016/j.bmcl.2016.03.029.

585. Lee, J.-C.; Chang, F.-R.; Chen, S.-R.; Wu, Y.-H.; Hu, H.-C.; Wu, Y.-C.; Backlund, A.; Cheng, Y.-B. Anti-*Dengue* Virus Constituents from Formosan Zoanthid *Palythoa mutuki*. *Marine Drugs* **2016**, *14*, 151, doi:10.3390/md14080151.

586. Pinto, F.; Almeida, J.G.; Silveira, E.; Costa, A.; Guimarães, L.; Wilke, D.; Costa-Lotufo, L.; Torres, M. da C.; Pessoa, O.D. Steroids from the Brazilian Zoanthids *Palythoa caribaeorum* and Palythoa Variabilis. *J. Braz. Chem. Soc.* **2016**, doi:10.21577/0103-5053.20160323.

587. Roy, P.; Maarisit, W.; Roy, M.; Taira, J.; Ueda, K. Five New Diterpenoids from an Okinawan Soft Coral, *Cespitularia* sp. *Marine Drugs* **2012**, *10*, 2741–2748, doi:10.3390/md10122741.

588. Bhujanga Rao, C.; Babu, D.C.; Bharadwaj, T.V.; Srikanth, D.; Vardhan, K.S.; Raju, T.V.; Bunce, R.A.; Venkateswarlu, Y. Isolation, Structural Assignment and Synthesis of (*S E*)-2-Methyloctyl 3-(4-Methoxyphenyl) Propenoate from the Marine Soft Coral *Sarcophyton ehrenbergi*. *Natural Product Research* **2015**, *29*, 70–76, doi:10.1080/14786419.2014.958742.
589. Sun, Z.-H.; Cai, Y.-H.; Fan, C.-Q.; Tang, G.-H.; Luo, H.-B.; Yin, S. Six New Tetraprenylated Alkaloids from the South China Sea *Gorgonian echinogorgia* Pseudossapo. *Marine Drugs* **2014**, *12*, 672–681, doi:10.3390/md12020672.
590. Guzii, A.G.; Makarieva, T.N.; Fedorov, S.N.; Denisenko, V.A.; Dmitrenok, P.S.; Kuzmich, A.S.; Krasokhin, V.B.; Lee, H.-S.; Lee, Y.-J.; Stonik, V.A. Gramine-Derived Bromo-Alkaloids Activating NF-KB-Dependent Transcription from the Marine Hydroid *Abietinaria abietina*. *Natural Product Communications* **2016**, *11*, 1934578X1601100, doi:10.1177/1934578X1601100918.
591. Li, C.; La, M.-P.; Tang, H.; Sun, P.; Liu, B.-S.; Zhuang, C.-L.; Yi, Y.-H.; Zhang, W. Chemistry and Bioactivity of Briaranes from the South China Sea Gorgonian *Dichotella gemmacea*. *Marine Drugs* **2016**, *14*, 201, doi:10.3390/md14110201.
592. Gao, C.-H.; Wang, Y.-F.; Li, S.; Qian, P.-Y.; Qi, S.-H. Alkaloids and Sesquiterpenes from the South China Sea Gorgonian *Echinogorgia pseudossapo*. *Marine Drugs* **2011**, *9*, 2479–2487, doi:10.3390/md9112479.
593. Pénez, N.; Culioli, G.; Pérez, T.; Briand, J.-F.; Thomas, O.P.; Blache, Y. Antifouling Properties of Simple Indole and Purine Alkaloids from the Mediterranean Gorgonian *Paramuricea clavata*. *J. Nat. Prod.* **2011**, *74*, 2304–2308, doi:10.1021/np200537v.
594. Zhang, J.-R.; Li, P.-L.; Tang, X.-L.; Qi, X.; Li, G.-Q. Cytotoxic Tetraprenylated Alkaloids from the South China Sea Gorgonian *Euplexaura robusta*. *Chemistry & Biodiversity* **2012**, *9*, 2218–2224, doi:10.1002/cbdv.201100374.
595. Keyzers, R.A.; Gray, C.A.; Schleyer, M.H.; Whibley, C.E.; Hendricks, D.T.; Davies-Coleman, M.T. Malonganenones A–C, Novel Tetraprenylated Alkaloids from the Mozambique Gorgonian *Leptogorgia gilchristi*. *Tetrahedron* **2006**, *62*, 2200–2206, doi:10.1016/j.tet.2005.12.018.
596. Putra, M.Y.; Ianaro, A.; Panza, E.; Bavestrello, G.; Cerrano, C.; Fattorusso, E.; Taglialatela-Scafati, O. Sinulasulfoxide and Sinulasulfone, Sulfur-Containing Alkaloids from the Indonesian Soft Coral *Sinularia* sp. *Tetrahedron Letters* **2012**, *53*, 3937–3939, doi:10.1016/j.tetlet.2012.05.095.
597. Zhang, J.; Liang, Y.; Liao, X.-J.; Deng, Z.; Xu, S.-H. Isolation of a New Butenolide from the South China Sea Gorgonian Coral *Subergorgia suberosa*. *Natural Product Research* **2014**, *28*, 150–155, doi:10.1080/14786419.2013.857668.
598. Gao, C.; Lin, L.; Long, B.; Chen, Y.; He, B.; Sun, H.; Huang, R. A New Diketopiperazine from the Gorgonian Coral *Menella kanisa*. *Natural Product Research* **2014**, *28*, 473–476, doi:10.1080/14786419.2013.879134.
599. Shi, H.; Yu, S.; Liu, D.; van Ofwegen, L.; Proksch, P.; Lin, W. Sinularones A–I, New Cyclopentenone and Butenolide Derivatives from a Marine Soft Coral *Sinularia* sp. and Their Antifouling Activity. *Marine Drugs* **2012**, *10*, 1331–1344, doi:10.3390/md10061331.

600. Chao, C.-H.; Huang, T.-Z.; Wu, C.-Y.; Chen, B.-W.; Huang, C.-Y.; Hwang, T.-L.; Dai, C.-F.; Sheu, J.-H. Steroidal and α-Tocopherylhydroquinone Glycosides from Two Soft Corals *Cladiella hirsuta* and Sinularia Nanolobata. *RSC Adv.* **2015**, *5*, 74256–74262, doi:10.1039/C5RA13436F.
601. Zhou, Y.-M.; Shao, C.-L.; Wang, C.-Y.; Huang, H.; Xu, Y.; Qian, P.-Y. Chemical Constituents of the Gorgonian *Dichotella fragilis* (Ridleg) from the South China Sea. *Natural Product Communications* **2011**, *6*, 1934578X1100600, doi:10.1177/1934578X1100600907.
602. Cheng, Z.-B.; Deng, Y.-L.; Fan, C.-Q.; Han, Q.-H.; Lin, S.-L.; Tang, G.-H.; Luo, H.-B.; Yin, S. Prostaglandin Derivatives: Nonaromatic Phosphodiesterase-4 Inhibitors from the Soft Coral *Sarcophyton ehrenbergi*. *J. Nat. Prod.* **2014**, *77*, 1928–1936, doi:10.1021/np500394d.
603. Shen, Y.-C.; Lo, K.-L.; Chang, J.-Y.; Lin, Y.-S.; Mendbayar, K.; Kuo, Y.-H.; Lin, Y.-C. New Cytotoxic Prostanoids from Taiwanese Soft Coral *Clavularia viridis*. *Chemistry & Biodiversity* **2010**, *7*, 2702–2708, doi:10.1002/cbdv.200900378.
604. Putra, M.Y.; Ianaro, A.; Panza, E.; Bavestrello, G.; Cerrano, C.; Fattorusso, E.; Taglialatela-Scafati, O. Sinularioside, a Triacetylated Glycolipid from the Indonesian Soft Coral *Sinularia* sp., Is an Inhibitor of NO Release. *Bioorganic & Medicinal Chemistry Letters* **2012**, *22*, 2723–2725, doi:10.1016/j.bmcl.2012.02.102.
605. Gao, C.; Wang, Y.; Chen, Y.; He, B.; Zhang, R.; Xu, M.; Huang, R. Two New Avermectin Derivatives from the Beibu Gulf Gorgonian *Anthogorgia Caerulea*. *Chemistry & Biodiversity* **2014**, *11*, 812–818, doi:10.1002/cbdv.201300265.
606. Nuzzo, G.; Gomes, B.A.; Luongo, E.; Torres, M.C.M.; Santos, E.A.; Cutignano, A.; Pessoa, O.D.L.; Costa-Lotufo, L.V.; Fontana, A. Dinoflagellate-Related Amphidinolides from the Brazilian Octocoral *Stragulum bicolor*. *J. Nat. Prod.* **2016**, *79*, 1881–1885, doi:10.1021/acs.jnatprod.6b00259.
607. Nuzzo, G.; Gomes, B.; Gallo, C.; Amodeo, P.; Sansone, C.; Pessoa, O.; Manzo, E.; Vitale, R.; Ianora, A.; Santos, E.; et al. Potent Cytotoxic Analogs of Amphidinolides from the Atlantic Octocoral *Stragulum bicolor*. *Marine Drugs* **2019**, *17*, 58, doi:10.3390/md17010058.
608. Zhao, M.; He, T.; Shi, S.-K.; Song, L.-X.; Lu, Y. A New Dolabellane Diterpene and a New Polyacetylene from the Soft Coral *Clavularia viridis*. *Natural Product Research* **2018**, *32*, 1104–1108, doi:10.1080/14786419.2017.1380025.
609. Lin, Y.-F.; Kuo, C.-Y.; Wen, Z.-H.; Lin, Y.-Y.; Wang, W.-H.; Su, J.-H.; Sheu, J.-H.; Sung, P.-J. Flexibilisquinone, a New Anti-Inflammatory Quinone from the Cultured Soft Coral *Sinularia flexibilis*. *Molecules* **2013**, *18*, 8160–8167, doi:10.3390/molecules18078160.
610. Cheng, S.-Y.; Huang, K.-J.; Wang, S.-K.; Duh, C.-Y. Capilloquinol: A Novel Farnesyl Quinol from the Dongsha Atoll Soft Coral *Sinularia capillosa*. *Marine Drugs* **2011**, *9*, 1469–1476, doi:10.3390/md9091469.
611. Lu, Y.-N.; Cui, P.; Tian, X.-Q.; Lou, L.-G.; Fan, C.-Q. Unusual Cytotoxic Steroidal Saponins from the Gorgonian *Astrogorgia dumbea*. *Planta Med* **2016**, *82*, 882–887, doi:10.1055/s-0042-106168.
612. Zhao, H.-Y.; Shao, C.-L.; Li, Z.-Y.; Han, L.; Cao, F.; Wang, C.-Y. Bioactive Pregnane Steroids from a South China Sea Gorgonian *Carijoa* sp. *Molecules* **2013**, *18*, 3458–3466, doi:10.3390/molecules18033458.

613. Wang, P.; Tang, H.; Liu, B.-S.; Li, T.-J.; Sun, P.; Zhu, W.; Luo, Y.-P.; Zhang, W. Tumor Cell Growth Inhibitory Activity and Structure–Activity Relationship of Polyoxygenated Steroids from the Gorgonian *Menella kanisa*. *Steroids* **2013**, *78*, 951–958, doi:10.1016/j.steroids.2013.05.019.
614. Liu, T.-F.; Lu, X.; Tang, H.; Zhang, M.-M.; Wang, P.; Sun, P.; Liu, Z.-Y.; Wang, Z.-L.; Li, L.; Rui, Y.-C.; et al. 3β,5α,6β-Oxygenated Sterols from the South China Sea Gorgonian *Muriceopsis flavida* and Their Tumor Cell Growth Inhibitory Activity and Apoptosis-Inducing Function. *Steroids* **2013**, *78*, 108–114, doi:10.1016/j.steroids.2012.10.003.
615. Wang, S.-K.; Puu, S.-Y.; Duh, C.-Y. New Steroids from the Soft Coral *Nephthea chabrolii*. *Marine Drugs* **2013**, *11*, 571–580, doi:10.3390/md11020571.
616. Zhang, J.; Liao, X.-J.; Wang, K.-L.; Deng, Z.; Xu, S.-H. Cytotoxic Cholesta-1,4-Dien-3-One Derivatives from Soft Coral *Nephthea* sp. *Steroids* **2013**, *78*, 396–400, doi:10.1016/j.steroids.2012.12.012.
617. Zhang, J.; Li, L.-C.; Wang, K.-L.; Liao, X.-J.; Deng, Z.; Xu, S.-H. Pentacyclic Hemiacetal Sterol with Antifouling and Cytotoxic Activities from the Soft Coral *Nephthea* sp. *Bioorganic & Medicinal Chemistry Letters* **2013**, *23*, 1079–1082, doi:10.1016/j.bmcl.2012.12.012.
618. Chao, C.-H.; Wu, Y.-C.; Wen, Z.-H.; Sheu, J.-H. Steroidal Carboxylic Acids from Soft Coral *Paraminabea acronocephala*. *Marine Drugs* **2013**, *11*, 136–145, doi:10.3390/md11010136.
619. Gong, K.-K.; Tang, X.-L.; Zhang, G.; Cheng, C.-L.; Zhang, X.-W.; Li, P.-L.; Li, G.-Q. Polyhydroxylated Steroids from the South China Sea Soft Coral *Sarcophyton* sp. and Their Cytotoxic and Antiviral Activities. *Marine Drugs* **2013**, *11*, 4788–4798, doi:10.3390/md11124788.
620. Wang, Z.; Tang, H.; Wang, P.; Gong, W.; Xue, M.; Zhang, H.; Liu, T.; Liu, B.; Yi, Y.; Zhang, W. Bioactive Polyoxygenated Steroids from the South China Sea Soft Coral, *Sarcophyton* sp. *Marine Drugs* **2013**, *11*, 775–787, doi:10.3390/md11030775.
621. Sun, L.-L.; Fu, X.-M.; Li, X.-B.; Xing, Q.; Wang, C.-Y. New 18-Oxygenated Polyhydroxy Steroid from a South China Sea Soft Coral *Sarcophyton* sp. *Natural Product Research* **2013**, *27*, 2006–2011, doi:10.1080/14786419.2013.814053.
622. Shaaban, M.; Ghani, M.A.; Shaaban, K.A. Zahramycins A-B, Two New Steroids from the Coral *Sarcophyton trocheliophorum*. *Zeitschrift für Naturforschung B* **2013**, *68*, 939–945, doi:10.5560/znb.2013-3131.
623. Fang, H.-Y.; Hsu, C.-H.; Chao, C.-H.; Wen, Z.-H.; Wu, Y.-C.; Dai, C.-F.; Sheu, J.-H. Cytotoxic and Anti-Inflammatory Metabolites from the Soft Coral *Scleronephthya gracillimum*. *Marine Drugs* **2013**, *11*, 1853–1865, doi:10.3390/md11061853.
624. Huang, C.-Y.; Liaw, C.-C.; Chen, B.-W.; Chen, P.-C.; Su, J.-H.; Sung, P.-J.; Dai, C.-F.; Chiang, M.Y.; Sheu, J.-H. Withanolide-Based Steroids from the Cultured Soft Coral *Sinularia brassica*. *J. Nat. Prod.* **2013**, *76*, 1902–1908, doi:10.1021/np400454q.
625. Ahmed, S.; Ibrahim, A.; Arafa, A.S. Anti-H5N1 Virus Metabolites from the Red Sea Soft Coral, *Sinularia candidula*. *Tetrahedron Letters* **2013**, *54*, 2377–2381, doi:10.1016/j.tetlet.2013.02.088.

626. Tseng, Y.-J.; Wang, S.-K.; Duh, C.-Y. Secosteroids and Norcembranoids from the Soft Coral *Sinularia nanolobata*. *Marine Drugs* **2013**, *11*, 3288–3296, doi:10.3390/md11093288.
627. Zhang, G.; Tang, X.; Cheng, C.; Gong, K.; Zhang, X.; Zhu, H.; Wu, R.; Li, P.; Li, G. Cytotoxic 9,11-Secosteroids from the South China Sea Gorgonian *Subergorgia suberosa*. *Steroids* **2013**, *78*, 845–850, doi:10.1016/j.steroids.2013.05.009.
628. Zhang, J.; Liang, Y.; Wang, K.-L.; Liao, X.-J.; Deng, Z.; Xu, S.-H. Antifouling Steroids from the South China Sea Gorgonian Coral *Subergorgia suberosa*. *Steroids* **2014**, *79*, 1–6, doi:10.1016/j.steroids.2013.10.007.
629. Cao, F.; Shao, C.-L.; Chen, M.; Zhang, M.-Q.; Xu, K.-X.; Meng, H.; Wang, C.-Y. Antiviral C-25 Epimers of 26-Acetoxy Steroids from the South China Sea Gorgonian *Echinogorgia rebekka*. *J. Nat. Prod.* **2014**, *77*, 1488–1493, doi:10.1021/np500252q.
630. Xu, R.; Jin, M.-H.; Jiao, Y.; Xing, G.-S.; Zhao, W.-J.; Zhao, C.; Duan, H.-Q.; Tang, S.-A. Novel Polyhydroxylated Steroids from the East China Sea Gorgonian *Echinogorgia Sassapo reticulata* with Suppressive Activity of Leukotriene C4 Generation and Degranulation in Bone Marrow-Derived Mast Cells. *Journal of Asian Natural Products Research* **2014**, *16*, 351–357, doi:10.1080/10286020.2013.879469.
631. Moritz, M.; Marostica, L.; Bianco, É.; Almeida, M.; Carraro, J.; Cabrera, G.; Palermo, J.; Simões, C.; Schenkel, E. Polyoxygenated Steroids from the Octocoral *Leptogorgia punicea* and in Vitro Evaluation of Their Cytotoxic Activity. *Marine Drugs* **2014**, *12*, 5864–5880, doi:10.3390/md12125864.
632. Thao, N.P.; Luyen, B.T.T.; Sun, Y.N.; Song, S.B.; Thanh, N.V.; Cuong, N.X.; Nam, N.H.; Kiem, P.V.; Kim, Y.H.; Minh, C.V. NF-KB Inhibitory Activity of Polyoxygenated Steroids from the Vietnamese Soft Coral *Sarcophyton pauciplicatum*. *Bioorganic & Medicinal Chemistry Letters* **2014**, *24*, 2834–2838, doi:10.1016/j.bmcl.2014.04.103.
633. Chen, W.-T.; Liu, H.-L.; Yao, L.-G.; Guo, Y.-W. 9,11-Secosteroids and Polyhydroxylated Steroids from Two South China Sea Soft Corals *Sarcophyton trocheliophorum* and *Sinularia flexibilis*. *Steroids* **2014**, *92*, 56–61, doi:10.1016/j.steroids.2014.08.027.
634. Liu, M.; Shao, C.-L.; Chen, M.; Qi, J.; Wang, Y.; Fang, Y.-C.; Wang, C.-Y. Bioactive 9,11-Secosteroids from Gorgonian *Subergorgia suberosa* Collected from the South China Sea. *Chemistry & Biodiversity* **2014**, *11*, 1109–1120, doi:10.1002/cbdv.201400021.
635. Cao, F.; Shao, C.-L.; Wang, Y.; Xu, K.-X.; Qi, X.; Wang, C.-Y. Polyhydroxylated Sterols from the South China Sea Gorgonian *Verrucella umbraculum*. *HCA* **2014**, *97*, 900–908, doi:10.1002/hlca.201300397.
636. Cao, F.; Liu, H.-Y.; Zhao, J.; Fang, Y.-C.; Wang, C.-Y. New 19-Oxygenated Steroid from the South China Sea Gorgonian *Dichotella gemmacea*. *Natural Product Research* **2015**, *29*, 169–173, doi:10.1080/14786419.2014.971321.
637. Tsai, C.-R.; Huang, C.-Y.; Chen, B.-W.; Tsai, Y.-Y.; Shih, S.-P.; Hwang, T.-L.; Dai, C.-F.; Wang, S.-Y.; Sheu, J.-H. New Bioactive Steroids from the Soft Coral *Klyxum flaccidum*. *RSC Adv.* **2015**, *5*, 12546–12554, doi:10.1039/C4RA13977A.

638. Tu, V.A.; Lyakhova, E.G.; Diep, C.N.; Kalinovsky, A.I.; Dmitrenok, P.S.; Cuong, N.X.; Thanh, N.V.; Menchinskaya, E.S.; Pislyagin, E.A.; Nam, N.H.; et al. Polyoxygenated Steroids from the Gorgonian *Menella woodin* with Capabilities to Modulate ROS Levels in Macrophages at Response to LPS. *Steroids* **2015**, *104*, 246–251, doi:10.1016/j.steroids.2015.10.010.
639. Zhang, N.-X.; Tang, X.-L.; van Ofwegen, L.; Xue, L.; Song, W.-J.; Li, P.-L.; Li, G.-Q. Cyclopentenone Derivatives and Polyhydroxylated Steroids from the Soft Coral *Sinularia acuta*. *Chemistry & Biodiversity* **2015**, *12*, 273–283, doi:10.1002/cbdv.201400044.
640. Sun, X.-P.; Cao, F.; Shao, C.-L.; Wang, M.; Zhang, X.-L.; Wang, C.-Y. Antibacterial Δ^1-3-Ketosteroids from the South China Sea Gorgonian Coral *Subergorgia rubra*. *Chemistry & Biodiversity* **2015**, *12*, 1068–1074, doi:10.1002/cbdv.201400217.
641. Inuzuka, T.; Kawazoe, Y.; Kobayashi, S.; Matsumoto, R.; Yabe, J.; Ohmura, S.; Uemura, D. Haebaruol, a 9,11-Secosteroid Isolated from the Soft Coral *Clavularia* sp. *Chem. Lett.* **2016**, *45*, 81–82, doi:10.1246/cl.150938.
642. Cardoso-Martínez, F.; de la Rosa, J.M.; Díaz-Marrero, A.R.; Darias, J.; D'Croz, L.; Jiménez-Antón, M.D.; Corral, M.J.; García, R.; Alunda, J.M.; Cueto, M. Oxysterols from an Octocoral of the Genus *Gorgonia* from the Eastern Pacific of Panama. *RSC Adv.* **2016**, *6*, 38579–38591, doi:10.1039/C6RA04521A.
643. Tseng, W.-R.; Huang, C.-Y.; Tsai, Y.-Y.; Lin, Y.-S.; Hwang, T.-L.; Su, J.-H.; Sung, P.-J.; Dai, C.-F.; Sheu, J.-H. New Cytotoxic and Anti-Inflammatory Steroids from the Soft Coral *Klyxum flaccidum*. *Bioorganic & Medicinal Chemistry Letters* **2016**, *26*, 3253–3257, doi:10.1016/j.bmcl.2016.05.060.
644. Zovko Končić, M.; Ioannou, E.; Sawadogo, W.R.; Abdel-Razik, A.F.; Vagias, C.; Diederich, M.; Roussis, V. 4α-Methylated Steroids with Cytotoxic Activity from the Soft Coral *Litophyton mollis*. *Steroids* **2016**, *115*, 130–135, doi:10.1016/j.steroids.2016.08.017.
645. Whuang, T.-Y.; Tsai, W.-C.; Chen, N.-F.; Chen, Z.-C.; Tsui, K.-H.; Wen, Z.-H.; Su, Y.-D.; Chang, Y.-C.; Chen, Y.-H.; Lu, M.-C.; et al. Columnaristerol A, a Novel 19-Norsterol from the Formosan Octocoral *Nephthea columnaris*. *Bioorganic & Medicinal Chemistry Letters* **2016**, *26*, 4966–4969, doi:10.1016/j.bmcl.2016.09.007.
646. Tsai, T.-C.; Huang, Y.-T.; Chou, S.-K.; Shih, M.-C.; Chiang, C.-Y.; Su, J.-H. Cytotoxic Oxygenated Steroids from the Soft Coral *Nephthea erecta*. *Chem. Pharm. Bull.* **2016**, *64*, 1519–1522, doi:10.1248/cpb.c16-00426.
647. Hegazy, M.-E.F.; Gamal-Eldeen, A.M.; Mohamed, T.A.; Alhammady, M.A.; Hassanien, A.A.; Shreadah, M.A.; Abdelgawad, I.I.; Elkady, E.M.; Paré, P.W. New Cytotoxic Constituents from the Red Sea Soft Coral *Nephthea* sp. *Natural Product Research* **2016**, *30*, 1266–1272, doi:10.1080/14786419.2015.1055266.
648. Chen, M.; Han, L.; Zhang, X.-L.; Wang, C.-Y. Cytotoxic 19-Oxygenated Steroids from the South China Sea Gorgonian, *Pacifigorgia senta*. *Natural Product Research* **2016**, *30*, 1431–1435, doi:10.1080/14786419.2015.1063055.
649. Chang, Y.-C.; Kuo, L.-M.; Su, J.-H.; Hwang, T.-L.; Kuo, Y.-H.; Lin, C.-S.; Wu, Y.-C.; Sheu, J.-H.; Sung, P.-J. Pinnigorgiols A–C, 9,11-Secosterols with a Rare Ring Arrangement from a Gorgonian Coral *Pinnigorgia* sp. *Tetrahedron* **2016**, *72*, 999–1004, doi:10.1016/j.tet.2015.12.072.

650. Chang, Y.-C.; Hwang, T.-L.; Sheu, J.-H.; Wu, Y.-C.; Sung, P.-J. New Anti-Inflammatory 9,11-Secosterols with a Rare Tricyclo[5,2,1,1]Decane Ring from a Formosan Gorgonian *Pinnigorgia* sp. *Marine Drugs* **2016**, *14*, 218, doi:10.3390/md14120218.
651. Su, Y.-D.; Cheng, C.-H.; Wen, Z.-H.; Wu, Y.-C.; Sung, P.-J. New Anti-Inflammatory Sterols from a Gorgonian *Pinnigorgia* sp. *Bioorganic & Medicinal Chemistry Letters* **2016**, *26*, 3060–3063, doi:10.1016/j.bmcl.2016.05.015.
652. Thanh, N.V.; Ngoc, N.T.; Anh, H.L.T.; Thung, D.C.; Thao, D.T.; Cuong, N.X.; Nam, N.H.; Kiem, P.V.; Minh, C.V. Steroid Constituents from the Soft Coral *Sinularia microspiculata*. *Journal of Asian Natural Products Research* **2016**, *18*, 938–944, doi:10.1080/10286020.2016.1173676.
653. Ngoc, N.T.; Huong, P.T.M.; Thanh, N.V.; Cuong, N.X.; Nam, N.H.; Thung, D.C.; Kiem, P.V.; Minh, C.V. Steroid Constituents from the Soft Coral *Sinularia nanolobata*. *Chem. Pharm. Bull.* **2016**, *64*, 1417–1419, doi:10.1248/cpb.c16-00385.
654. Sun, H.; Liu, F.; Feng, M.-R.; Peng, Q.; Liao, X.-J.; Liu, T.-T.; Zhang, J.; Xu, S.-H. Isolation of a New Cytotoxic Polyhydroxysterol from the South China Sea Soft Coral *Sinularia* sp. *Natural Product Research* **2016**, *30*, 2819–2824, doi:10.1080/14786419.2016.1166495.
655. Cheng, W.; Ren, J.; Huang, Q.; Long, H.; Jin, H.; Zhang, L.; Liu, H.; van Ofwegen, L.; Lin, W. Pregnane Steroids from a Gorgonian Coral *Subergorgia suberosa* with Anti-Flu Virus Effects. *Steroids* **2016**, *108*, 99–104, doi:10.1016/j.steroids.2016.02.003.
656. Huang, C.-Y.; Chang, C.-W.; Tseng, Y.-J.; Lee, J.; Sung, P.-J.; Su, J.-H.; Hwang, T.-L.; Dai, C.-F.; Wang, H.-C.; Sheu, J.-H. Bioactive Steroids from the Formosan Soft Coral *Umbellulifera petasites*. *Marine Drugs* **2016**, *14*, 180, doi:10.3390/md14100180.
657. Tung, N.H.; Van Minh, C.; Van Kiem, P.; Huong, H.T.; Nam, N.H.; Cuong, N.X.; Quang, T.H.; Nhiem, N.X.; Hyun, J.-H.; Kang, H.-K.; et al. Chemical Components from the Vietnamese Soft Coral *Lobophytum* sp. *Arch. Pharm. Res.* **2010**, *33*, 503–508, doi:10.1007/s12272-010-0402-3.
658. Cheng, W.; Liu, Z.; Yu, Y.; van Ofwegen, L.; Proksch, P.; Yu, S.; Lin, W. An Unusual Spinaceamine-Bearing Pregnane from a Soft Coral *Scleronephthya* sp. Inhibits the Migration of Tumor Cells. *Bioorganic & Medicinal Chemistry Letters* **2017**, *27*, 2736–2741, doi:10.1016/j.bmcl.2017.04.058.
659. Chang, Y.-C.; Hwang, T.-L.; Kuo, L.-M.; Sung, P.-J. Pinnisterols D–J, New 11-Acetoxy-9,11-Secosterols with a 1,4-Quinone Moiety from Formosan Gorgonian Coral *Pinnigorgia* sp. (Gorgoniidae). *Marine Drugs* **2017**, *15*, 11, doi:10.3390/md15010011.
660. Nguyen Hoai, N.; Nguyen Thi, H.; Tran Thi Hong, H.; Nguyen Van, T.; Nguyen Xuan, C.; Do Cong, T.; Phan Van, K.; Chau Van, M. Pregnane Steroids from the Vietnamese Octocoral *Carijoa riisei*. *Natural Product Research* **2017**, *31*, 2435–2440, doi:10.1080/14786419.2017.1324964.
661. Tsai, Y.-Y.; Huang, C.-Y.; Tseng, W.-R.; Chiang, P.-L.; Hwang, T.-L.; Su, J.-H.; Sung, P.-J.; Dai, C.-F.; Sheu, J.-H. Klyflaccisteroids K–M, Bioactive Steroidal Derivatives from a Soft Coral *Klyxum flaccidum*. *Bioorganic & Medicinal Chemistry Letters* **2017**, *27*, 1220–1224, doi:10.1016/j.bmcl.2017.01.060.
662. Aboutabl, E.A.; Selim, N.M.; Azzam, S.M.; Michel, C.G.; Hegazy, M.F.; Ali, A.M.; Hussein, A.A. Polyhydroxy Sterols Isolated from the Red Sea Soft Coral *Lobophytum crassum* and Their Cytotoxic Activity. *Natural Product Communications* **2017**, *12*, 1934578X1701200, doi:10.1177/1934578X1701200223.

663. Whuang, T.-Y.; Tsai, H.-C.; Su, Y.-D.; Hwang, T.-L.; Sung, P.-J. Sterols from the Octocoral *Nephthea columnaris*. *Marine Drugs* **2017**, *15*, 212, doi:10.3390/md15070212.
664. Chung, T.-W.; Su, J.-H.; Lin, C.-C.; Li, Y.-R.; Chao, Y.-H.; Lin, S.-H.; Chan, H.-L. 24-Methyl-Cholesta-5,24(28)-Diene-3β,19-Diol-7β-Monoacetate Inhibits Human Small Cell Lung Cancer Growth In Vitro and In Vivo via Apoptosis Induction. *Marine Drugs* **2017**, *15*, 210, doi:10.3390/md15070210.
665. Chang, Y.-C.; Hwang, T.-L.; Chao, C.-H.; Sung, P.-J. New Marine Sterols from a Gorgonian *Pinnigorgia* sp. *Molecules* **2017**, *22*, 393, doi:10.3390/molecules22030393.
666. He, Y.-Q.; Lee Caplan, S.; Scesa, P.; West, L.M. Cyclized 9,11-Secosterol Enol-Ethers from the Gorgonian *Pseudopterogorgia americana*. *Steroids* **2017**, *125*, 47–53, doi:10.1016/j.steroids.2017.06.008.
667. Tran, H.H.T.; Nguyen Viet, P.; Nguyen Van, T.; Tran, H.T.; Nguyen Xuan, C.; Nguyen Hoai, N.; Do Cong, T.; Phan Van, K.; Chau Van, M. Cytotoxic Steroid Derivatives from the Vietnamese Soft Coral *Sinularia brassica*. *Journal of Asian Natural Products Research* **2017**, *19*, 1183–1190, doi:10.1080/10286020.2017.1307192.
668. Huang, C.-Y.; Su, J.-H.; Liaw, C.-C.; Sung, P.-J.; Chiang, P.-L.; Hwang, T.-L.; Dai, C.-F.; Sheu, J.-H. Bioactive Steroids with Methyl Ester Group in the Side Chain from a Reef Soft Coral Sinularia *Brassica cultured* in a Tank. *Marine Drugs* **2017**, *15*, 280, doi:10.3390/md15090280.
669. Ngoc, N.T.; Huong, P.T.M.; Thanh, N.V.; Chi, N.T.P.; Dang, N.H.; Cuong, N.X.; Nam, N.H.; Thung, D.C.; Kiem, P.V.; Minh, C.V. Cytotoxic Steroids from the Vietnamese Soft Coral *Sinularia conferta*. *Chem. Pharm. Bull.* **2017**, *65*, 300–305, doi:10.1248/cpb.c16-00881.
670. Ngoc, N.T.; Hanh, T.T.H.; Thanh, N.V.; Thao, D.T.; Cuong, N.X.; Nam, N.H.; Thung, D.C.; Kiem, P.V.; Minh, C.V. Cytotoxic Steroids from the Vietnamese Soft Coral *Sinularia Leptoclados*. *Chem. Pharm. Bull.* **2017**, *65*, 593–597, doi:10.1248/cpb.c17-00129.
671. Ayyad, S.-E.N.; Alarif, W.M.; Al-Footy, K.O.; Selim, E.A.; Ghandourah, M.A.; Aly, M.M.; Alorfi, H.S. Isolation, Antimicrobial and Antitumor Activities of a New Polyhydroxysteroid and a New Diterpenoid from the Soft Coral *Xenia umbellata*. *Zeitschrift für Naturforschung C* **2017**, *72*, 27–34, doi:10.1515/znc-2015-0228.
672. Huang, C.-Y.; Ahmed, A.F.; Su, J.-H.; Sung, P.-J.; Hwang, T.-L.; Chiang, P.-L.; Dai, C.-F.; Liaw, C.-C.; Sheu, J.-H. Bioactive New Withanolides from the Cultured Soft Coral *Sinularia brassica*. *Bioorganic & Medicinal Chemistry Letters* **2017**, *27*, 3267–3271, doi:10.1016/j.bmcl.2017.06.029.
673. Nam, N.H.; Ngoc, N.T.; Hanh, T.T.H.; Cuong, N.X.; Thanh, N.V.; Thao, D.T.; Thung, D.C.; Kiem, P.V.; Minh, C.V. Cytotoxic Steroids from the Vietnamese Gorgonian *Verrucella corona*. *Steroids* **2018**, *138*, 57–63, doi:10.1016/j.steroids.2018.06.006.
674. Li, S.-W.; Chen, W.-T.; Yao, L.-G.; Guo, Y.-W. Two New Cytotoxic Steroids from the Chinese Soft Coral *Sinularia* sp. *Steroids* **2018**, *136*, 17–21, doi:10.1016/j.steroids.2018.05.009.

675. Huang, C.-Y.; Tseng, W.-R.; Ahmed, A.; Chiang, P.-L.; Tai, C.-J.; Hwang, T.-L.; Dai, C.-F.; Sheu, J.-H. Anti-Inflammatory Polyoxygenated Steroids from the Soft Coral *Lobophytum michaelae*. *Marine Drugs* **2018**, *16*, 93, doi:10.3390/md16030093.
676. Wu, J.; Xi, Y.; Huang, L.; Li, G.; Mao, Q.; Fang, C.; Shan, T.; Jiang, W.; Zhao, M.; He, W.; et al. A Steroid-Type Antioxidant Targeting the Keap1/Nrf2/ARE Signaling Pathway from the Soft Coral *Dendronephthya gigantea*. *J. Nat. Prod.* **2018**, *81*, 2567–2575, doi:10.1021/acs.jnatprod.8b00728.
677. Jiang, C.-S.; Ru, T.; Huan, X.-J.; Miao, Z.-H.; Guo, Y.-W. New Cytotoxic Ergostane-Type Sterols from the Chinese Soft Coral *Sinularia* sp. *Steroids* **2019**, *149*, 108425, doi:10.1016/j.steroids.2019.108425.
678. Liu, C.-Y.; Hwang, T.-L.; Lin, M.-R.; Chen, Y.-H.; Chang, Y.-C.; Fang, L.-S.; Wang, W.-H.; Wu, Y.-C.; Sung, P.-J. Carijoside A, a Bioactive Sterol Glycoside from an Octocoral *Carijoa* sp. (Clavulariidae). *Marine Drugs* **2010**, *8*, 2014–2020, doi:10.3390/md8072014.
679. Lai, D.; Yu, S.; van Ofwegen, L.; Totzke, F.; Proksch, P.; Lin, W. 9,10-Secosteroids, Protein Kinase Inhibitors from the Chinese Gorgonian *Astrogorgia* sp. *Bioorganic & Medicinal Chemistry* **2011**, *19*, 6873–6880, doi:10.1016/j.bmc.2011.09.028.
680. Díaz-Marrero, A.R.; Porras, G.; Aragón, Z.; de la Rosa, J.M.; Dorta, E.; Cueto, M.; D'Croz, L.; Maté, J.; Darias, J. Carijodienone from the Octocoral *Carijoa multiflora* . A Spiropregnane-Based Steroid. *J. Nat. Prod.* **2011**, *74*, 292–295, doi:10.1021/np1007608.
681. Ngoc, N.T.; Hanh, T.T.H.; Cuong, N.X.; Nam, N.H.; Thung, D.C.; Ivanchina, N.V.; Dang, N.H.; Kicha, A.A.; Kiem, P.V.; Minh, C.V. Steroids from Dendronephthya Mucronata and Their Inhibitory Effects on Lipopolysaccharide-Induced No Formation in RAW264.7 Cells. *Chem Nat Compd* **2019**, *55*, 1090–1093, doi:10.1007/s10600-019-02901-6.
682. Huynh, T.-H.; Chen, P.-C.; Yang, S.-N.; Lin, F.-Y.; Su, T.-P.; Chen, L.-Y.; Peng, B.-R.; Hu, C.-C.; Chen, Y.-Y.; Wen, Z.-H.; et al. New 1,4-Dienonesteroids from the Octocoral *Dendronephthya* sp. *Marine Drugs* **2019**, *17*, 530, doi:10.3390/md17090530.
683. Zhang, Q.; Liang, L.-F.; Miao, Z.-H.; Wu, B.; Guo, Y.-W. Cytotoxic Polyhydroxylated Steroids from the South China Sea Soft Coral *Lobophytum* sp. *Steroids* **2019**, *141*, 76–80, doi:10.1016/j.steroids.2018.11.015.
684. Chen, B.-W.; Chang, S.-M.; Huang, C.-Y.; Su, J.-H.; Wen, Z.-H.; Wu, Y.-C.; Sheu, J.-H. Hirsutosterols A–G, Polyoxygenated Steroids from a Formosan Soft Coral *Cladiella hirsuta*. *Org. Biomol. Chem.* **2011**, *9*, 3272, doi:10.1039/c1ob05106g.
685. Chen, W.-H.; Wang, S.-K.; Duh, C.-Y. Polyhydroxylated Steroids from the Octocoral *Isis hippuris*. *Tetrahedron* **2011**, *67*, 8116–8119, doi:10.1016/j.tet.2011.08.053.
686. Uddin, M.H.; Hanif, N.; Trianto, A.; Agarie, Y.; Higa, T.; Tanaka, J. Four New Polyoxygenated Gorgosterols from the Gorgonian *Isis hippuris*. *Natural Product Research* **2011**, *25*, 585–591, doi:10.1080/14786419.2010.485303.
687. Chen, W.-H.; Wang, S.-K.; Duh, C.-Y. Polyhydroxylated Steroids from the Bamboo Coral *Isis hippuris*. *Marine Drugs* **2011**, *9*, 1829–1839, doi:10.3390/md9101829.

688. Quang, T.H.; Ha, T.T.; Minh, C.V.; Kiem, P.V.; Huong, H.T.; Ngan, N.T.T.; Nhiem, N.X.; Tung, N.H.; Thao, N.P.; Thuy, D.T.T.; et al. Cytotoxic and PPARs Transcriptional Activities of Sterols from the Vietnamese Soft Coral *Lobophytum laevigatum*. *Bioorganic & Medicinal Chemistry Letters* **2011**, *21*, 2845–2849, doi:10.1016/j.bmcl.2011.03.089.
689. Wang, P.; Qi, S.-H.; Liu, K.-S.; Huang, L.-S.; He, F.; Wang, Y.-F. Steroids from the South China Sea Gorgonian Coral *Muricella flexuosa*. *Zeitschrift für Naturforschung B* **2011**, *66*, 635–640, doi:10.1515/znb-2011-0612.
690. Uddin, M.H.; Roy, M.C.; Tanaka, J. Cytotoxic Cholic Acid Type Sterones from a Marine Soft Coral *Paraminabea* sp. *Chem Nat Compd* **2011**, *47*, 64–67, doi:10.1007/s10600-011-9831-6.
691. Qi, S.-H.; Miao, L.; Gao, C.-H.; Xu, Y.; Zhang, S.; Qian, P.-Y. New Steroids and a New Alkaloid from the Gorgonian *Isis minorbrachyblasta:* Structures, Cytotoxicity, and Antilarval Activity. *HCA* **2010**, *93*, 511–516, doi:10.1002/hlca.200900250.
692. Yeffet, D.; Rudi, A.; Ketzinel, S.; Benayahu, Y.; Kashman, Y. Auroside, a Xylosyl-Sterol, and Patusterol A and B, Two Hydroxylated Sterols, from Two Soft Corals *Eleutherobia aurea* and *Lobophytum patulum*. *Natural Product Communications* **2010**, *5*, 1934578X1000500, doi:10.1177/1934578X1000500207.
693. Lu, Y.; Lin, Y.-C.; Wen, Z.-H.; Su, J.-H.; Sung, P.-J.; Hsu, C.-H.; Kuo, Y.-H.; Chiang, M.Y.; Dai, C.-F.; Sheu, J.-H. Steroid and Cembranoids from the Dongsha Atoll Soft Coral *Lobophytum sarcophytoides*. *Tetrahedron* **2010**, *66*, 7129–7135, doi:10.1016/j.tet.2010.06.094.
694. Chai, X.-Y.; Sun, J.-F.; Tang, L.-Y.; Yang, X.-W.; Li, Y.-Q.; Huang, H.; Zhou, X.-F.; Yang, B.; Liu, Y. A Novel Cyclopentene Derivative and a Polyhydroxylated Steroid from a South China Sea Gorgonian *Menella* sp. *Chem. Pharm. Bull.* **2010**, *58*, 1391–1394, doi:10.1248/cpb.58.1391.
695. Chao, C.-H.; Chou, K.-J.; Wen, Z.-H.; Wang, G.-H.; Wu, Y.-C.; Dai, C.-F.; Sheu, J.-H. Paraminabeolides A–F, Cytotoxic and Anti-Inflammatory Marine Withanolides from the Soft Coral *Paraminabea acronocephala*. *J. Nat. Prod.* **2011**, *74*, 1132–1141, doi:10.1021/np2000705.
696. Kuo, C.-Y.; Juan, Y.-S.; Lu, M.-C.; Chiang, M.; Dai, C.-F.; Wu, Y.-C.; Sung, P.-J. Pregnane-Type Steroids from the Formosan Soft Coral *Scleronephthya flexilis*. *IJMS* **2014**, *15*, 10136–10149, doi:10.3390/ijms150610136.
697. Fang, H.-Y.; Liaw, C.-C.; Chao, C.-H.; Wen, Z.-H.; Wu, Y.-C.; Hsu, C.-H.; Dai, C.-F.; Sheu, J.-H. Bioactive Pregnane-Type Steroids from the Soft Coral *Scleronephthya gracillimum*. *Tetrahedron* **2012**, *68*, 9694–9700, doi:10.1016/j.tet.2012.09.060.
698. Chao, C.-H.; Chou, K.-J.; Huang, C.-Y.; Wen, Z.-H.; Hsu, C.-H.; Wu, Y.-C.; Dai, C.-F.; Sheu, J.-H. Steroids from the Soft Coral *Sinularia crassa*. *Marine Drugs* **2012**, *10*, 439–450, doi:10.3390/md10020439.
699. Huang, C.-Y.; Su, J.-H.; Duh, C.-Y.; Chen, B.-W.; Wen, Z.-H.; Kuo, Y.-H.; Sheu, J.-H. A New 9,11-Secosterol from the Soft Coral *Sinularia granosa*. *Bioorganic & Medicinal Chemistry Letters* **2012**, *22*, 4373–4376, doi:10.1016/j.bmcl.2012.05.002.

700. Sun, P.; Meng, L.-Y.; Tang, H.; Liu, B.-S.; Li, L.; Yi, Y.; Zhang, W. Sinularosides A and B, Bioactive 9,11-Secosteroidal Glycosides from the South China Sea Soft Coral *Sinularia humilis* Ofwegen. *J. Nat. Prod.* **2012**, *75*, 1656–1659, doi:10.1021/np300475d.
701. Liao, X.-J.; Tang, L.-D.; Liang, Y.-W.; Jian, S.-L.; Xu, S.-H.; Liu, Y.-H. New Pentahydroxylated Steroids from the South China Sea Gorgonian *Subergorgia suberosa*. *Chemistry & Biodiversity* **2012**, *9*, 370–375, doi:10.1002/cbdv.201100031.
702. Su, J.-H.; Chen, B.-Y.; Hwang, T.-L.; Chen, Y.-H.; Huang, I.-C.; Lin, M.-R.; Chen, J.-J.; Fang, L.-S.; Wang, W.-H.; Li, J.-J.; et al. Excavatoids L-N, New 12-Hydroxybriaranes from the Cultured Octocoral *Briareum excavatum* (Briareidae). *Chem. Pharm. Bull.* **2010**, *58*, 662–665, doi:10.1248/cpb.58.662.
703. Chen, D.; Yu, S.; van Ofwegen, L.; Proksch, P.; Lin, W. Anthogorgienes A–O, New Guaiazulene-Derived Terpenoids from a Chinese Gorgonian Anthogorgia Species, and Their Antifouling and Antibiotic Activities. *J. Agric. Food Chem.* **2012**, *60*, 112–123, doi:10.1021/jf2040862.
704. Chen, D.; Liu, D.; Shen, S.; Cheng, W.; Lin, W. Terpenoids from a Chinese Gorgonian *Anthogorgia* sp. and Their Antifouling Activities. *Chin. J. Chem.* **2012**, *30*, 1459–1463, doi:10.1002/cjoc.201200412.
705. Lai, D.; Liu, D.; Deng, Z.; van Ofwegen, L.; Proksch, P.; Lin, W. Antifouling Eunicellin-Type Diterpenoids from the Gorgonian *Astrogorgia* sp. *J. Nat. Prod.* **2012**, *75*, 1595–1602, doi:10.1021/np300404f.
706. Gómez-Reyes, J.; Salazar, A.; Guzmán, H.; González, Y.; Fernández, P.; Ariza-Castolo, A.; Gutiérrez, M. Seco-Briarellinone and Briarellin S, Two New Eunicellin-Based Diterpenoids from the Panamanian Octocoral *Briareum asbestinum*. *Marine Drugs* **2012**, *10*, 2608–2617, doi:10.3390/md10112608.
707. Meginley, R.J.; Gupta, P.; Schulz, T.C.; McLean, A.B.; Robins, A.J.; West, L.M. Briareolate Esters from the Gorgonian *Briareum asbestinum*. *Marine Drugs* **2012**, *10*, 1662–1670, doi:10.3390/md10081662.
708. Yeh, T.-T.; Wang, S.-K.; Dai, C.-F.; Duh, C.-Y. Briacavatolides A–C, New Briaranes from the Taiwanese Octocoral *Briareum excavatum*. *Marine Drugs* **2012**, *10*, 1019–1026, doi:10.3390/md10051019.
709. Hong, P.-H.; Su, Y.-D.; Su, J.-H.; Chen, Y.-H.; Hwang, T.-L.; Weng, C.-F.; Lee, C.-H.; Wen, Z.-H.; Sheu, J.-H.; Lin, N.-C.; et al. Briarenolides F and G, New Briarane Diterpenoids from a *Briareum* sp. Octocoral. *Marine Drugs* **2012**, *10*, 1156–1168, doi:10.3390/md10051156.
710. Su, Y.-D.; Hwang, T.-L.; Lin, N.-C.; Chen, Y.-H.; Wu, Y.-C.; Sheu, J.-H.; Sung, P.-J. Briarenolides H and I: New 8-Hydroxybriarane Diterpenoids from a Formosan Octocoral *Briareum* sp. (Briareidae). *BCSJ* **2012**, *85*, 1031–1036, doi:10.1246/bcsj.20120147.
711. Hong, P.-H.; Su, Y.-D.; Lin, N.-C.; Chen, Y.-H.; Kuo, Y.-H.; Hwang, T.-L.; Wang, W.-H.; Chen, J.-J.; Sheu, J.-H.; Sung, P.-J. Briarenolide E: The First 2-Ketobriarane Diterpenoid from an Octocoral *Briareum* sp. (Briareidae). *Tetrahedron Letters* **2012**, *53*, 1710–1712, doi:10.1016/j.tetlet.2012.01.077.
712. Sung, P.-J.; Li, G.-Y.; Su, Y.-D.; Lin, M.-R.; Chang, Y.-C.; Kung, T.-H.; Lin, C.-S.; Chen, Y.-H.; Su, J.-H.; Lu, M.-C.; et al. Excavatoids O and P, New 12-Hydroxybriaranes from the Octocoral *Briareum excavatum*. *Marine Drugs* **2010**, *8*, 2639–2646, doi:10.3390/md8102639.

713. Chang, J.-Y.; Fazary, A.E.; Lin, Y.-C.; Hwang, T.-L.; Shen, Y.-C. New Verticillane Diterpenoids from *Cespitularia taeniata*. *Chemistry & Biodiversity* **2012**, *9*, 654–661, doi:10.1002/cbdv.201100122.
714. Chen, Y.-H.; Tai, C.-Y.; Hwang, T.-L.; Sung, P.-J. Cladieunicellin H, a New Hemiketal Eunicellin-Based Diterpenoid from the Octocoral *Cladiella* sp. *Natural Product Communications* **2012**, *7*, 1934578X1200700, doi:10.1177/1934578X1200700416.
715. Chen, Y.-H.; Hwang, T.-L.; Su, Y.-D.; Chang, Y.-C.; Chen, Y.-H.; Hong, P.-H.; Hu, L.-C.; Yen, W.-H.; Hsu, H.-Y.; Huang, S.-J.; et al. New 6-Hydroxyeunicellins from a Soft Coral *Cladiella* sp. *Chem. Pharm. Bull.* **2012**, *60*, 160–163, doi:10.1248/cpb.60.160.
716. Li, C.; La, M.-P.; Tang, H.; Pan, W.-H.; Sun, P.; Krohn, K.; Yi, Y.-H.; Li, L.; Zhang, W. Bioactive Briarane Diterpenoids from the South China Sea Gorgonian *Dichotella gemmacea*. *Bioorganic & Medicinal Chemistry Letters* **2012**, *22*, 4368–4372, doi:10.1016/j.bmcl.2012.05.001.
717. Cheng, C.-H.; Chung, H.-M.; Hwang, T.-L.; Lu, M.-C.; Wen, Z.-H.; Kuo, Y.-H.; Wang, W.-H.; Sung, P.-J. Echinoclerodane A: A New Bioactive Clerodane-Type Diterpenoid from a Gorgonian Coral *Echinomuricea* sp. *Molecules* **2012**, *17*, 9443–9450, doi:10.3390/molecules17089443.
718. Chung, H.-M.; Hu, L.-C.; Yen, W.-H.; Su, J.-H.; Lu, M.-C.; Hwang, T.-L.; Wang, W.-H.; Sung, P.-J. Echinohalimane A, a Bioactive Halimane-Type Diterpenoid from a Formosan Gorgonian *Echinomuricea* sp. (Plexauridae). *Marine Drugs* **2012**, *10*, 2246–2253, doi:10.3390/md10102246.
719. Chung, H.-M.; Hong, P.-H.; Su, J.-H.; Hwang, T.-L.; Lu, M.-C.; Fang, L.-S.; Wu, Y.-C.; Li, J.-J.; Chen, J.-J.; Wang, W.-H.; et al. Bioactive Compounds from a Gorgonian Coral *Echinomuricea* sp. (Plexauridae). *Marine Drugs* **2012**, *10*, 1169–1179, doi:10.3390/md10051169.
720. Marchbank, D.H.; Berrue, F.; Kerr, R.G. Eunicidiol, an Anti-Inflammatory Dilophol Diterpene from *Eunicea fusca*. *J. Nat. Prod.* **2012**, *75*, 1289–1293, doi:10.1021/np300149y.
721. Tello, E.; Castellanos, L.; Arévalo-Ferro, C.; Duque, C. Disruption in Quorum-Sensing Systems and Bacterial Biofilm Inhibition by Cembranoid Diterpenes Isolated from the Octocoral *Eunicea knighti*. *J. Nat. Prod.* **2012**, *75*, 1637–1642, doi:10.1021/np300313k.
722. Wei, X.; Nieves, K.; Rodríguez, A.D. Bioactive Cubitane Diterpenoids from a Colombian Gorgonian Species of the *Genus eunicea*. *Pure and Applied Chemistry* **2012**, *84*, 1847–1855, doi:10.1351/PAC-CON-11-10-20.
723. Ospina, C.A.; Rodríguez, A.D.; Ortega-Barria, E.; Capson, T.L. Briarellins J−P and Polyanthellin A: New Eunicellin-Based Diterpenes from the Gorgonian Coral *Briareum polyanthes* and Their Antimalarial Activity. *J. Nat. Prod.* **2003**, *66*, 357–363, doi:10.1021/np0204500.
724. Chang, J.-Y.; Liaw, C.-C.; Fazary, A.E.; Hwang, T.-L.; Shen, Y.-C. New Briarane Diterpenoids from the Gorgonian Coral *Junceella juncea*. *Marine Drugs* **2012**, *10*, 1321–1330, doi:10.3390/md10061321.
725. Wu, S.-L.; Su, J.-H.; Huang, C.-Y.; Tai, C.-J.; Sung, P.-J.; Liaw, C.-C.; Sheu, J.-H. Simplexins P–S, Eunicellin-Based Diterpenes from the Soft Coral *Klyxum simplex*. *Marine Drugs* **2012**, *10*, 1203–1211, doi:10.3390/md10061203.

726. Wang, S.-K.; Duh, C.-Y. New Cytotoxic Cembranolides from the Soft Coral *Lobophytum michaelae*. *Marine Drugs* **2012**, *10*, 306–318, doi:10.3390/md10020306.
727. Govindam, S.V.S.; Yoshioka, Y.; Kanamoto, A.; Fujiwara, T.; Okamoto, T.; Ojika, M. Cyclolobatriene, a Novel Prenylated Germacrene Diterpene, from the Soft Coral *Lobophytum pauciflorum*. *Bioorganic & Medicinal Chemistry* **2012**, *20*, 687–692, doi:10.1016/j.bmc.2011.12.012.
728. Huang, L.-S.; He, F.; Huang, H.; Zhang, X.-Y.; Qi, S.-H. Carbamate Derivatives and Sesquiterpenoids from the South China Sea Gorgonian *Melitodes squamata*. *Beilstein J. Org. Chem.* **2012**, *8*, 170–176, doi:10.3762/bjoc.8.18.
729. Lee, C.-H.; Kao, C.-Y.; Kao, S.-Y.; Chang, C.-H.; Su, J.-H.; Hwang, T.-L.; Kuo, Y.-H.; Wen, Z.-H.; Sung, P.-J. Terpenoids from the Octocorals Menella sp. (Plexauridae) and *Lobophytum crassum* (Alcyonacea). *Marine Drugs* **2012**, *10*, 427–438, doi:10.3390/md10020427.
730. Lin, W.-Y.; Lu, Y.; Chen, B.-W.; Huang, C.-Y.; Su, J.-H.; Wen, Z.-H.; Dai, C.-F.; Kuo, Y.-H.; Sheu, J.-H. Sarcocrassocolides M–O, Bioactive Cembranoids from the Dongsha Atoll Soft Coral *Sarcophyton crassocaule*. *Marine Drugs* **2012**, *10*, 617–626, doi:10.3390/md10030617.
731. Wang, S.-K.; Hsieh, M.-K.; Duh, C.-Y. Three New Cembranoids from the Taiwanese Soft Coral *Sarcophyton ehrenbergi*. *Marine Drugs* **2012**, *10*, 1433–1444, doi:10.3390/md10071433.
732. Hegazy, M.-E.F.; Eldeen, A.M.G.; Shahat, A.A.; Abdel-Latif, F.F.; Mohamed, T.A.; Whittlesey, B.R.; Paré, P.W. Bioactive Hydroperoxyl Cembranoids from the Red Sea Soft Coral *Sarcophyton glaucum*. *Marine Drugs* **2012**, *10*, 209–222, doi:10.3390/md10010209.
733. Chen, S.-P.; Chen, B.-W.; Dai, C.-F.; Sung, P.-J.; Wu, Y.-C.; Sheu, J.-H. Sarcophytonins F and G, New Dihydrofuranocembranoids from a Dongsha Atoll Soft Coral *Sarcophyton* sp. *BCSJ* **2012**, *85*, 920–922, doi:10.1246/bcsj.20120100.
734. Sung, P.-J.; Lin, M.-R.; Chiang, M.Y.; Huang, I.-C.; Syu, S.-M.; Fang, L.-S.; Wang, W.-H.; Sheu, J.-H. Briarenolide D, a New Hydroperoxybriarane Diterpenoid from a Cultured Octocoral *Briareum* sp. *Chem. Lett.* **2010**, *39*, 1030–1032, doi:10.1246/cl.2010.1030.
735. Yao, L.-G.; Zhang, H.-Y.; Liang, L.-F.; Guo, X.-J.; Mao, S.-C.; Guo, Y.-W. Yalongenes A and B, Two New Cembranoids with Cytoprotective Effects from the Hainan Soft Coral *Sarcophyton trocheliophorum* Marenzeller. *HCA* **2012**, *95*, 235–239, doi:10.1002/hlca.201100278.
736. Shih, H.-J.; Tseng, Y.-J.; Huang, C.-Y.; Wen, Z.-H.; Dai, C.-F.; Sheu, J.-H. Cytotoxic and Anti-Inflammatory Diterpenoids from the Dongsha Atoll Soft Coral *Sinularia flexibilis*. *Tetrahedron* **2012**, *68*, 244–249, doi:10.1016/j.tet.2011.10.054.
737. Tseng, Y.-J.; Shen, K.-P.; Lin, H.-L.; Huang, C.-Y.; Dai, C.-F.; Sheu, J.-H. Lochmolins A–G, New Sesquiterpenoids from the Soft Coral *Sinularia lochmodes*. *Marine Drugs* **2012**, *10*, 1572–1581, doi:10.3390/md10071572.
738. Shen, S.; Zhu, H.; Chen, D.; Liu, D.; Ofwegen, L. van; Proksch, P.; Lin, W. Pavidolides A–E, New Cembranoids from the Soft Coral *Sinularia pavida*. *Tetrahedron Letters* **2012**, *53*, 5759–5762, doi:10.1016/j.tetlet.2012.08.049.

739. Su, J.-H.; Huang, C.-Y.; Li, P.-J.; Lu, Y.; Wen, Z.-H.; Kao, Y.-H.; Sheu, J.-H. Bioactive Cadinane-Type Compounds from the Soft Coral *Sinularia scabra*. *Arch. Pharm. Res.* **2012**, *35*, 779–784, doi:10.1007/s12272-012-0503-2.
740. Wright, A.D.; Nielson, J.L.; Tapiolas, D.M.; Liptrot, C.H.; Motti, C.A. A Great Barrier Reef Sinularia sp. Yields Two New Cytotoxic Diterpenes. *Marine Drugs* **2012**, *10*, 1619–1630, doi:10.3390/md10081619.
741. Yen, W.-H.; Hu, L.-C.; Su, J.-H.; Lu, M.-C.; Twan, W.-H.; Yang, S.-Y.; Kuo, Y.-C.; Weng, C.-F.; Lee, C.-H.; Kuo, Y.-H.; et al. Norcembranoidal Diterpenes from a Formosan Soft Coral *Sinularia* sp. *Molecules* **2012**, *17*, 14058–14066, doi:10.3390/molecules171214058.
742. Chen, B.-W.; Chang, S.-M.; Huang, C.-Y.; Chao, C.-H.; Su, J.-H.; Wen, Z.-H.; Hsu, C.-H.; Dai, C.-F.; Wu, Y.-C.; Sheu, J.-H. Hirsutalins A−H, Eunicellin-Based Diterpenoids from the Soft Coral *Cladiella hirsuta*. *J. Nat. Prod.* **2010**, *73*, 1785–1791, doi:10.1021/np100401f.
743. Hassan, H.M.; Khanfar, M.A.; Elnagar, A.Y.; Mohammed, R.; Shaala, L.A.; Youssef, D.T.A.; Hifnawy, M.S.; El Sayed, K.A. Pachycladins A−E, Prostate Cancer Invasion and Migration Inhibitory Eunicellin-Based Diterpenoids from the Red Sea Soft Coral *Cladiella pachyclados*. *J. Nat. Prod.* **2010**, *73*, 848–853, doi:10.1021/np900787p.
744. Chen, B.-W.; Wang, S.-Y.; Huang, C.-Y.; Chen, S.-L.; Wu, Y.-C.; Sheu, J.-H. Hirsutalins I–M, Eunicellin-Based Diterpenoids from the Soft *Coral cladiella* Hirsuta. *Tetrahedron* **2013**, *69*, 2296–2301, doi:10.1016/j.tet.2013.01.015.
745. Lee, Y.-N.; Tai, C.-J.; Hwang, T.-L.; Sheu, J.-H. Krempfielins J-M, New Eunicellin-Based Diterpenoids from the Soft Coral *Cladiella krempfi*. *Marine Drugs* **2013**, *11*, 2741–2750, doi:10.3390/md11082741.
746. Tai, C.-J.; Su, J.-H.; Huang, C.-Y.; Huang, M.-S.; Wen, Z.-H.; Dai, C.-F.; Sheu, J.-H. Cytotoxic and Anti-Inflammatory Eunicellin-Based Diterpenoids from the Soft Coral *Cladiella krempfi*. *Marine Drugs* **2013**, *11*, 788–799, doi:10.3390/md11030788.
747. Chen, T.-H.; Lu, M.-C.; Chang, Y.-C.; Su, Y.-D.; Chen, Y.-H.; Lin, N.-C.; Fang, L.-S.; Wu, Y.-C.; Sung, P.-J. Discovery of New Eunicellin-Based Diterpenoids from a Formosan Soft Coral *Cladiella* sp. *Marine Drugs* **2013**, *11*, 4585–4593, doi:10.3390/md11114585.
748. Shih, F.-Y.; Chen, T.-H.; Lu, M.-C.; Chen, W.-F.; Wen, Z.-H.; Kuo, Y.-H.; Sung, P.-J. Cladieunicellins K and L, New Eunicellin-Based Diterpenoids from an Octocoral *Cladiella* sp. *IJMS* **2013**, *14*, 21781–21789, doi:10.3390/ijms141121781.
749. Williams, D.E.; Amlani, A.; Dewi, A.S.; Patrick, B.O.; van Ofwegen, L.; Mui, A.L.-F.; Andersen, R.J. Australin E Isolated from the Soft Coral *Cladiella* sp. Collected in Pohnpei Activates the Inositol 5-Phosphatase SHIP1. *Aust. J. Chem.* **2010**, *63*, 895, doi:10.1071/CH10053.
750. Li, C.; Jiang, M.; La, M.-P.; Li, T.-J.; Tang, H.; Sun, P.; Liu, B.-S.; Yi, Y.-H.; Liu, Z.; Zhang, W. Chemistry and Tumor Cell Growth Inhibitory Activity of 11,20-Epoxy-3Z,5(6)E-Diene Briaranes from the South China Sea Gorgonian *Dichotella gemmacea*. *Marine Drugs* **2013**, *11*, 1565–1582, doi:10.3390/md11051565.

751. Sun, J.-F.; Han, Z.; Zhou, X.-F.; Yang, B.; Lin, X.; Liu, J.; Peng, Y.; Yang, X.-W.; Liu, Y. Antifouling Briarane Type Diterpenoids from South China Sea Gorgonians *Dichotella gemmacea. Tetrahedron* **2013**, *69*, 871–880, doi:10.1016/j.tet.2012.10.102.
752. Liaw, C.-C.; Lin, Y.-C.; Lin, Y.-S.; Chen, C.-H.; Hwang, T.-L.; Shen, Y.-C. Four New Briarane Diterpenoids from Taiwanese Gorgonian *Junceella fragilis*. *Marine Drugs* **2013**, *11*, 2042–2053, doi:10.3390/md11062042.
753. Lin, M.-C.; Chen, B.-W.; Huang, C.-Y.; Dai, C.-F.; Hwang, T.-L.; Sheu, J.-H. Eunicellin-Based Diterpenoids from the Formosan Soft Coral *Klyxum molle* with Inhibitory Activity on Superoxide Generation and Elastase Release by Neutrophils. *J. Nat. Prod.* **2013**, *76*, 1661–1667, doi:10.1021/np400372v.
754. Xio, Y.-J.; Su, J.-H.; Chen, B.-W.; Tseng, Y.-J.; Wu, Y.-C.; Sheu, J.-H. Oxygenated Ylangene-Derived Sesquiterpenoids from the Soft Coral *Lemnalia philippinensis*. *Marine Drugs* **2013**, *11*, 3735–3741, doi:10.3390/md11103735.
755. Yan, P.; Lv, Y.; van Ofwegen, L.; Proksch, P.; Lin, W. Lobophytones A−G, New Isobiscembranoids from the Soft Coral *Lobophytum pauciflorum*. *Org. Lett.* **2010**, *12*, 2484–2487, doi:10.1021/ol100567d.
756. Zhao, M.; Li, X.; Zhao, F.; Cheng, S.; Xiang, Z.; Dong, J.; Huang, K.; Yan, P. Four New 7,8-Epoxycembranoids from a Chinese Soft Coral *Lobophytum* sp. *Chem. Pharm. Bull.* **2013**, *61*, 1323–1328, doi:10.1248/cpb.c13-00612.
757. Zhao, M.; Yin, J.; Jiang, W.; Ma, M.; Lei, X.; Xiang, Z.; Dong, J.; Huang, K.; Yan, P. Cytotoxic and Antibacterial Cembranoids from a South China Sea Soft Coral, *Lobophytum* sp. *Marine Drugs* **2013**, *11*, 1162–1172, doi:10.3390/md11041162.
758. Li, T.-T.; Tang, X.-L.; Chen, C.-L.; Zhang, X.-W.; Wu, R.-C.; Zhu, H.-Y.; Li, P.-L.; Li, G.-Q. New Eunicellin Diterpenes and 9,10-Secosteroids from the Gorgonian *Muricella sibogae*. *HCA* **2013**, *96*, 1188–1196, doi:10.1002/hlca.201200489.
759. Tseng, Y.-J.; Lee, Y.-S.; Wang, S.-K.; Sheu, J.-H.; Duh, C.-Y. Parathyrsoidins A–D, Four New Sesquiterpenoids from the Soft Coral *Paralemnalia thyrsoides*. *Marine Drugs* **2013**, *11*, 2501–2509, doi:10.3390/md11072501.
760. Rojo de Almeida, M.T.; Siless, G.E.; Perez, C.D.; Veloso, M.J.; Schejter, L.; Puricelli, L.; Palermo, J.A. Dolabellane Diterpenoids from the South Atlantic Gorgonian *Convexella magelhaenica*. *J. Nat. Prod.* **2010**, *73*, 1714–1717, doi:10.1021/np100337j.
761. Chung, H.-M.; Wang, W.-H.; Hwang, T.-L.; Wu, Y.-C.; Sung, P.-J. Natural Clovanes from the Gorgonian Coral *Rumphella antipathies*. *Natural Product Communications* **2013**, *8*, 1934578X1300800, doi:10.1177/1934578X1300800801.
762. Wang, S.-K.; Hsieh, M.-K.; Duh, C.-Y. New Diterpenoids from Soft Coral Sarcophyton Ehrenbergi. *Marine Drugs* **2013**, *11*, 4318–4327, doi:10.3390/md11114318.
763. Xi, Z.; Bie, W.; Chen, W.; Liu, D.; Ofwegen, L.; Proksch, P.; Lin, W. Sarcophyolides B–E, New Cembranoids from the Soft Coral *Sarcophyton elegans*. *Marine Drugs* **2013**, *11*, 3186–3196, doi:10.3390/md11093186.

764. Zhang, C.-X.; He, X.-X.; Zhang, J.; Guo, Q.; Lei, L.-F.; Su, J.-Y.; Zeng, L.-M. New Precursor of Tetraterpenoids from the Soft Coral *Sarcophyton glaucum*. *Natural Product Research* **2013**, *27*, 782–786, doi:10.1080/14786419.2012.701211.
765. Abou El-Ezz, R.F.; Ahmed, S.A.; Radwan, M.M.; Ayoub, N.A.; Afifi, M.S.; Ross, S.A.; Szymanski, P.T.; Fahmy, H.; Khalifa, S.I. Bioactive Cembranoids from the Red Sea Soft Coral *Sarcophyton glaucum*. *Tetrahedron Letters* **2013**, *54*, 989–992, doi:10.1016/j.tetlet.2012.12.037.
766. Xi, Z.; Bie, W.; Chen, W.; Liu, D.; van Ofwegen, L.; Proksch, P.; Lin, W. Sarcophytolides G-L, New Biscembranoids from the Soft Coral *Sarcophyton elegans*. *HCA* **2013**, *96*, 2218–2227, doi:10.1002/hlca.201300086.
767. Liang, L.-F.; Kurtán, T.; Mándi, A.; Yao, L.-G.; Li, J.; Zhang, W.; Guo, Y.-W. Unprecedented Diterpenoids as a PTP1B Inhibitor from the Hainan Soft Coral *Sarcophyton trocheliophorum* Marenzeller. *Org. Lett.* **2013**, *15*, 274–277, doi:10.1021/ol303110d.
768. Chen, D.; Chen, W.; Liu, D.; van Ofwegen, L.; Proksch, P.; Lin, W. Asteriscane-Type Sesquiterpenoids from the Soft Coral *Sinularia capillosa*. *J. Nat. Prod.* **2013**, *76*, 1753–1763, doi:10.1021/np400480p.
769. Hu, L.-C.; Su, J.-H.; Chiang, M.; Lu, M.-C.; Hwang, T.-L.; Chen, Y.-H.; Hu, W.-P.; Lin, N.-C.; Wang, W.-H.; Fang, L.-S.; et al. Flexibilins A–C, New Cembrane-Type Diterpenoids from the Formosan Soft Coral, *Sinularia flexibilis*. *Marine Drugs* **2013**, *11*, 1999–2012, doi:10.3390/md11061999.
770. Su, C.-C.; Wong, B.-S.; Chin, C.; Wu, Y.-J.; Su, J.-H. Oxygenated Cembranoids from the Soft Coral *Sinularia flexibilis*. *IJMS* **2013**, *14*, 4317–4325, doi:10.3390/ijms14024317.
771. Chen, B.-W.; Chao, C.-H.; Su, J.-H.; Wen, Z.-H.; Sung, P.-J.; Sheu, J.-H. Anti-Inflammatory Eunicellin-Based Diterpenoids from the Cultured Soft Coral *Klyxum simplex*. *Org. Biomol. Chem.* **2010**, *8*, 2363, doi:10.1039/b926353e.
772. Hu, L.-C.; Yen, W.-H.; Su, J.-H.; Chiang, M.; Wen, Z.-H.; Chen, W.-F.; Lu, T.-J.; Chang, Y.-W.; Chen, Y.-H.; Wang, W.-H.; et al. Cembrane Derivatives from the Soft Corals, *Sinularia gaweli* and *Sinularia flexibilis*. *Marine Drugs* **2013**, *11*, 2154–2167, doi:10.3390/md11062154.
773. Tsai, T.-C.; Wu, Y.-J.; Su, J.-H.; Lin, W.-T.; Lin, Y.-S. A New Spatane Diterpenoid from the Cultured Soft Coral *Sinularia leptoclados*. *Marine Drugs* **2013**, *11*, 114–123, doi:10.3390/md11010114.
774. Thao, N.P.; Nam, N.H.; Cuong, N.X.; Quang, T.H.; Tung, P.T.; Dat, L.D.; Chae, D.; Kim, S.; Koh, Y.-S.; Kiem, P.V.; et al. Anti-Inflammatory Norditerpenoids from the Soft Coral *Sinularia maxima*. *Bioorganic & Medicinal Chemistry Letters* **2013**, *23*, 228–231, doi:10.1016/j.bmcl.2012.10.129.
775. Lai, D.; Geng, Z.; Deng, Z.; van Ofwegen, L.; Proksch, P.; Lin, W. Cembranoids from the Soft Coral Sinularia Rigida with Antifouling Activities. *J. Agric. Food Chem.* **2013**, *61*, 4585–4592, doi:10.1021/jf401303q.
776. Yin, J.; Zhao, M.; Ma, M.; Xu, Y.; Xiang, Z.; Cai, Y.; Dong, J.; Lei, X.; Huang, K.; Yan, P. New Casbane Diterpenoids from a South China Sea Soft Coral, *Sinularia* sp. *Marine Drugs* **2013**, *11*, 455–465, doi:10.3390/md11020455.

777. Yang, B.; Liao, S.; Lin, X.; Wang, J.; Liu, J.; Zhou, X.; Yang, X.; Liu, Y. New Sinularianin Sesquiterpenes from Soft Coral *Sinularia* sp. *Marine Drugs* **2013**, *11*, 4741–4750, doi:10.3390/md11124741.
778. Wanzola, M.; Furuta, T.; Kohno, Y.; Fukumitsu, S.; Yasukochi, S.; Watari, K.; Tanaka, C.; Higuchi, R.; Miyamoto, T. Four New Cembrane Diterpenes Isolated from an Okinawan Soft Coral *Lobophytum crassum* with Inhibitory Effects on Nitric Oxide Production. *Chem. Pharm. Bull.* **2010**, *58*, 1203–1209, doi:10.1248/cpb.58.1203.
779. Yan, P.; Deng, Z.; Ofwegen, L. van; Proksch, P.; Lin, W. Lobophytones O–T, New Biscembranoids and Cembranoid from Soft Coral *Lobophytum pauciflorum*. *Marine Drugs* **2010**, *8*, 2837–2848, doi:10.3390/md8112848.
780. Gao, C.-H.; He, B.-J.; Chen, Y.-N.; Ke, K.; Lin, L.; Long, B.; Huang, R.-M. Two New Diterpenoids from the Beibu Gulf Gorgonian *Anthogorgia caerulea*. *Zeitschrift für Naturforschung B* **2014**, *69*, 116–120, doi:10.5560/znb.2014-3213.
781. Su, Y.-D.; Cheng, C.-H.; Chen, W.-F.; Chang, Y.-C.; Chen, Y.-H.; Hwang, T.-L.; Wen, Z.-H.; Wang, W.-H.; Fang, L.-S.; Chen, J.-J.; et al. Briarenolide J, the First 12-Chlorobriarane Diterpenoid from an Octocoral *Briareum* sp. (Briareidae). *Tetrahedron Letters* **2014**, *55*, 6065–6067, doi:10.1016/j.tetlet.2014.09.032.
782. Liaw, C.-C.; Cheng, Y.-B.; Lin, Y.-S.; Kuo, Y.-H.; Hwang, T.-L.; Shen, Y.-C. New Briarane Diterpenoids from Taiwanese Soft Coral *Briareum violacea*. *Marine Drugs* **2014**, *12*, 4677–4692, doi:10.3390/md12084677.
783. Lin, Y.-C.; Wang, S.-S.; Chen, C.-H.; Kuo, Y.-H.; Shen, Y.-C. Cespitulones A and B, Cytotoxic Diterpenoids of a New Structure Class from the Soft Coral *Cespitularia taeniata*. *Marine Drugs* **2014**, *12*, 3477–3486, doi:10.3390/md12063477.
784. Chen, T.-H.; Cheng, C.-H.; Chen, Y.-H.; Lu, M.-C.; Fang, L.-S.; Chen, W.-F.; Wen, Z.-H.; Wang, W.-H.; Wu, Y.-C.; Sung, P.-J. Cladieunicellin J, a New Hydroperoxyeunicellin from *Cladiella* sp. *Natural Product Communications* **2014**, *9*, 613–619, doi:10.1177/1934578X1400900504.
785. Huang, T.-Z.; Chen, B.-W.; Huang, C.-Y.; Hwang, T.-L.; Dai, C.-F.; Sheu, J.-H. Eunicellin-Based Diterpenoids, Hirsutalins N–R, from the Formosan Soft Coral *Cladiella hirsuta*. *Marine Drugs* **2014**, *12*, 2446–2457, doi:10.3390/md12052446.
786. Tai, C.-J.; Chokkalingam, U.; Cheng, Y.; Shih, S.-P.; Lu, M.-C.; Su, J.-H.; Hwang, T.-L.; Sheu, J.-H. Krempfielins Q and R, Two New Eunicellin-Based Diterpenoids from the Soft Coral *Cladiella krempfi*. *IJMS* **2014**, *15*, 21865–21874, doi:10.3390/ijms151221865.
787. Lee, Y.-N.; Tai, C.-J.; Hwang, T.-L.; Sheu, J.-H. Krempfielins N–P, New Anti-Inflammatory Eunicellins from a Taiwanese Soft Coral *Cladiella krempfi*. *Marine Drugs* **2014**, *12*, 1148–1156, doi:10.3390/md12021148.
788. Chen, T.-H.; Chen, W.-F.; Wen, Z.-H.; Lu, M.-C.; Wang, W.-H.; Li, J.-J.; Wu, Y.-C.; Sung, P.-J. Cladieunicellins M–Q, New Eunicellins from *Cladiella* sp. *Marine Drugs* **2014**, *12*, 2144–2155, doi:10.3390/md12042144.

789. Yamada, K.; Ryu, K.; Miyamoto, T.; Higuchi, R. Bioactive Terpenoids from Octocorallia. 4. Three New Cembrane-Type Diterpenoids from the Soft Coral *Lobophytum schoedei*. *J. Nat. Prod.* **1997**, *60*, 798–801, doi:10.1021/np960728m.
790. Elkhayat, E.S.; Ibrahim, S.R.M.; Fouad, M.A.; Mohamed, G.A. Dendronephthols A–C, New Sesquiterpenoids from the Red Sea Soft Coral *Dendronephthya* sp. *Tetrahedron* **2014**, *70*, 3822–3825, doi:10.1016/j.tet.2014.03.056.
791. La, M.-P.; Li, J.; Li, C.; Tang, H.; Liu, B.-S.; Sun, P.; Zhuang, C.-L.; Li, T.-J.; Zhang, W. Briarane Diterpenoids from the Gorgonian *Dichotella gemmacea*. *Marine Drugs* **2014**, *12*, 6178–6189, doi:10.3390/md12126178.
792. Figueroa, J.; Vera, B.; Rodríguez, A.D. Pintoxolanes A-C, Highly Functionalized 3,14-Oxa-Bridged Cembranoids from the Caribbean Gorgonian Coral *Eunicea pinta*. *HCA* **2014**, *97*, 712–721, doi:10.1002/hlca.201300282.
793. Lei, H.; Sun, J.-F.; Han, Z.; Zhou, X.-F.; Yang, B.; Liu, Y. Fragilisinins A–L, New Briarane-Type Diterpenoids from Gorgonian *Junceella fragilis*. *RSC Adv.* **2014**, *4*, 5261, doi:10.1039/c3ra46163g.
794. Chang, F.-Y.; Hsu, F.-J.; Tai, C.-J.; Wei, W.-C.; Yang, N.-S.; Sheu, J.-H. Klymollins T–X, Bioactive Eunicellin-Based Diterpenoids from the Soft Coral *Klyxum molle*. *Marine Drugs* **2014**, *12*, 3060–3071, doi:10.3390/md12053060.
795. Cheng, S.-Y.; Wang, S.-K.; Duh, C.-Y. Secocrassumol, a Seco-Cembranoid from the Dongsha Atoll Soft Coral *Lobophytum crassum. Marine Drugs* **2014**, *12*, 6028–6037, doi:10.3390/md12126028.
796. Chen, M.; Han, L.; Wang, Y.; Zhang, X.-L.; Wang, C.-Y. A New Sesquiterpene from the Hainan Gorgonian *Menella kanisa* Grassoff. *Natural Product Research* **2014**, *28*, 1147–1151, doi:10.1080/14786419.2014.918122.
797. Zhou, Y.-M.; Chen, M.; Fu, X.-M.; Fang, Y.-C.; Wang, C.-Y. Two New Eunicellin-Based Diterpenoids from the South China Sea Gorgonian *Muricella sibogae* Nutting. *Natural Product Research* **2014**, *28*, 1176–1181, doi:10.1080/14786419.2014.923423.
798. Cheng, S.-Y.; Shih, N.-L.; Hou, K.-Y.; Ger, M.-J.; Yang, C.-N.; Wang, S.-K.; Duh, C.-Y. Kelsoenethiol and Dikelsoenyl Ether, Two Unique Kelsoane-Type Sesquiterpenes, from the Formosan Soft Coral *Nephthea erecta*. *Bioorganic & Medicinal Chemistry Letters* **2014**, *24*, 473–475, doi:10.1016/j.bmcl.2013.12.037.
799. Bonnard, I.; Jhaumeer-Laulloo, S.B.; Bontemps, N.; Banaigs, B.; Aknin, M. New Lobane and Cembrane Diterpenes from Two Comorian Soft Corals. *Marine Drugs* **2010**, *8*, 359–372, doi:10.3390/md8020359.
800. Bahl, A.; Jachak, S.M.; Palaniveloo, K.; Ramachandram, T.; Vairappan, C.S.; Chopra, H.K. 2-Acetoxyverecynarmin C, a New Briarane COX Inhibitory Diterpenoid from *Pennatula aculeata*. *Natural Product Communications* **2014**, *9*, 1934578X1400900, doi:10.1177/1934578X1400900820.
801. Chung, H.-M.; Wang, W.-H.; Hwang, T.-L.; Chen, J.-J.; Fang, L.-S.; Wen, Z.-H.; Wang, Y.-B.; Wu, Y.-C.; Sung, P.-J. Rumphellols A and B, New Caryophyllene Sesquiterpenoids from a Formosan Gorgonian Coral, *Rumphella antipathies*. *IJMS* **2014**, *15*, 15679–15688, doi:10.3390/ijms150915679.

802. Chung, H.-M.; Wang, W.-H.; Hwang, T.-L.; Fang, L.-S.; Wen, Z.-H.; Chen, J.-J.; Wu, Y.-C.; Sung, P.-J. Rumphellaoic Acid A, a Novel Sesquiterpenoid from the Formosan Gorgonian Coral *Rumphella antipathies*. *Marine Drugs* **2014**, *12*, 5856–5863, doi:10.3390/md12125856.
803. Chung, H.-M.; Wang, W.-H.; Hwang, T.-L.; Li, J.-J.; Fang, L.-S.; Wu, Y.-C.; Sung, P.-J. Rumphellaones B and C, New 4,5-Seco-Caryophyllane Sesquiterpenoids from *Rumphella*. *Molecules* **2014**, *19*, 12320–12327, doi:10.3390/molecules190812320.
804. Eltahawy, N.A.; Ibrahim, A.K.; Radwan, M.M.; ElSohly, M.A.; Hassanean, H.A.; Ahmed, S.A. Cytotoxic Cembranoids from the Red Sea Soft Coral, *Sarcophyton auritum*. *Tetrahedron Letters* **2014**, *55*, 3984–3988, doi:10.1016/j.tetlet.2014.05.013.
805. Lin, W.-Y.; Chen, B.-W.; Huang, C.-Y.; Wen, Z.-H.; Sung, P.-J.; Su, J.-H.; Dai, C.-F.; Sheu, J.-H. Bioactive Cembranoids, Sarcocrassocolides P–R, from the Dongsha Atoll Soft Coral *Sarcophyton crassocaule*. *Marine Drugs* **2014**, *12*, 840–850, doi:10.3390/md12020840.
806. Elkhateeb, A.; El-Beih, A.; Gamal-Eldeen, A.; Alhammady, M.; Ohta, S.; Paré, P.; Hegazy, M.-E. New Terpenes from the Egyptian Soft Coral *Sarcophyton ehrenbergi*. *Marine Drugs* **2014**, *12*, 1977–1986, doi:10.3390/md12041977.
807. Al-Lihaibi, S.S.; Alarif, W.M.; Abdel-Lateff, A.; Ayyad, S.-E.N.; Abdel-Naim, A.B.; El-Senduny, F.F.; Badria, F.A. Three New Cembranoid-Type Diterpenes from Red Sea Soft Coral *Sarcophyton glaucum*: Isolation and Antiproliferative Activity against HepG2 Cells. *European Journal of Medicinal Chemistry* **2014**, *81*, 314–322, doi:10.1016/j.ejmech.2014.05.016.
808. Cheng, Z.-B.; Liao, Q.; Chen, Y.; Fan, C.-Q.; Huang, Z.-Y.; Xu, X.-J.; Yin, S. Four New Cembranoids from the Soft Coral *Sarcophyton* sp.: Four New Cembranoids from the Soft Coral *Sarcophyton* sp. *Magn. Reson. Chem.* **2014**, *52*, 515–520, doi:10.1002/mrc.4108.
809. Lin, K.-H.; Tseng, Y.-J.; Chen, B.-W.; Hwang, T.-L.; Chen, H.-Y.; Dai, C.-F.; Sheu, J.-H. Tortuosenes A and B, New Diterpenoid Metabolites from the Formosan Soft Coral *Sarcophyton tortuosum*. *Org. Lett.* **2014**, *16*, 1314–1317, doi:10.1021/ol403723b.
810. Wang, G.-H.; Huang, H.-C.; Su, J.-H.; Wu, Y.-C.; Sheu, J.-H. Paralemnolins J-P, New Sesquiterpenoids from the Soft Coral *Paralemnalia thyrsoide*. *Chem. Pharm. Bull.* **2010**, *58*, 30–33, doi:10.1248/cpb.58.30.
811. Liang, L.-F.; Kurtán, T.; Mándi, A.; Gao, L.-X.; Li, J.; Zhang, W.; Guo, Y.-W. Sarsolenane and Capnosane Diterpenes from the Hainan Soft Coral *Sarcophyton trocheliophorum* Marenzeller as PTP1B Inhibitors: Sarsolenane and Capnosane Diterpenes as PTP1B Inhibitors. *Eur. J. Org. Chem.* **2014**, *2014*, 1841–1847, doi:10.1002/ejoc.201301683.
812. Chen, K.-H.; Dai, C.-F.; Hwang, T.-L.; Chen, C.-Y.; Li, J.-J.; Chen, J.-J.; Wu, Y.-C.; Sheu, J.-H.; Wang, W.-H.; Sung, P.-J. Discovery of Novel Diterpenoids from *Sinularia arborea*. *Marine Drugs* **2014**, *12*, 385–393, doi:10.3390/md12010385.
813. Cheng, S.-Y.; Shih, N.-L.; Chuang, C.-T.; Chiou, S.-F.; Yang, C.-N.; Wang, S.-K.; Duh, C.-Y. Sinugyrosanolide A, an Unprecedented C-4 Norcembranoid, from the Formosan Soft Coral *Sinularia gyrosa*. *Bioorganic & Medicinal Chemistry Letters* **2014**, *24*, 1562–1564, doi:10.1016/j.bmcl.2014.01.073.

814. Lei, L.-F.; Chen, M.-F.; Wang, T.; He, X.-X.; Liu, B.-X.; Deng, Y.; Chen, X.-J.; Li, Y.-T.; Guan, S.-Y.; Yao, J.-H.; et al. Novel Cytotoxic Nine-Membered Macrocyclic Polysulfur Cembranoid Lactones from the Soft Coral *Sinularia* sp. *Tetrahedron* **2014**, *70*, 6851–6858, doi:10.1016/j.tet.2014.07.042.
815. Andrianasolo, E.; Haramaty, L.; White, E.; Lutz, R.; Falkowski, P. Mode of Action of Diterpene and Characterization of Related Metabolites from the Soft Coral, *Xenia elongata*. *Marine Drugs* **2014**, *12*, 1102–1115, doi:10.3390/md12021102.
816. Phan, C.-S.; Ng, S.-Y.; Kim, E.-A.; Jeon, Y.-J.; Palaniveloo, K.; Vairappan, C. Capgermacrenes A and B, Bioactive Secondary Metabolites from a Bornean Soft Coral, *Capnella* sp. *Marine Drugs* **2015**, *13*, 3103–3115, doi:10.3390/md13053103.
817. Cheng, S.-Y.; Lin, E.-H.; Huang, J.-S.; Wen, Z.-H.; Duh, C.-Y. Ylangene-Type and Nardosinane-Type Sesquiterpenoids from the Soft Corals Lemnalia Flava and *Paralemnalia thyrsoides*. *Chem. Pharm. Bull.* **2010**, *58*, 381–385, doi:10.1248/cpb.58.381.
818. Su, Y.-D.; Su, T.-R.; Wen, Z.-H.; Hwang, T.-L.; Fang, L.-S.; Chen, J.-J.; Wu, Y.-C.; Sheu, J.-H.; Sung, P.-J. Briarenolides K and L, New Anti-Inflammatory Briarane Diterpenoids from an Octocoral *Briareum* sp. (Briareidae). *Marine Drugs* **2015**, *13*, 1037–1050, doi:10.3390/md13021037.
819. Su, Y.-D.; Wu, T.-Y.; Wen, Z.-H.; Su, C.-C.; Chen, Y.-H.; Chang, Y.-C.; Wu, Y.-C.; Sheu, J.-H.; Sung, P.-J. Briarenolides U–Y, New Anti-Inflammatory Briarane Diterpenoids from an Octocoral *Briareum* sp. (Briareidae). *Marine Drugs* **2015**, *13*, 7138–7149, doi:10.3390/md13127060.
820. Wang, S.-S.; Cheng, Y.-B.; Lin, Y.-C.; Liaw, C.-C.; Chang, J.-Y.; Kuo, Y.-H.; Shen, Y.-C. Nitrogen-Containing Diterpenoids, Sesquiterpenoids, and Nor-Diterpenoids from *Cespitularia taeniata*. *Marine Drugs* **2015**, *13*, 5796–5814, doi:10.3390/md13095796.
821. Ghandourah, M.A.; Alarif, W.M.; Abdel-Lateff, A.; Al-Lihaibi, S.S.; Ayyad, S.-E.N.; Basaif, S.A.; Badria, F.A. Two New Terpenoidal Derivatives: A Himachalene-Type Sesquiterpene and 13,14-Secosteroid from the Soft Coral *Litophyton arboreum*. *Med Chem Res* **2015**, *24*, 4070–4077, doi:10.1007/s00044-015-1456-z.
822. Hsiao, T.-H.; Sung, C.-S.; Lan, Y.-H.; Wang, Y.-C.; Lu, M.-C.; Wen, Z.-H.; Wu, Y.-C.; Sung, P.-J. New Anti-Inflammatory Cembranes from the Cultured Soft Coral *Nephthea columnaris*. *Marine Drugs* **2015**, *13*, 3443–3453, doi:10.3390/md13063443.
823. Martín, M.J.; Fernández, R.; Francesch, A.; Amade, P.; de Matos-Pita, S.S.; Reyes, F.; Cuevas, C. Plumisclerin A, a Diterpene with a New Skeleton from the Soft Coral *Plumigorgia terminosclera*. *Org. Lett.* **2010**, *12*, 912–914, doi:10.1021/ol902802h.
824. Eltahawy, N.A.; Ibrahim, A.K.; Radwan, M.M.; Zaitone, S.A.; Gomaa, M.; ElSohly, M.A.; Hassanean, H.A.; Ahmed, S.A. Mechanism of Action of Antiepileptic Ceramide from Red Sea Soft Coral *Sarcophyton auritum*. *Bioorganic & Medicinal Chemistry Letters* **2015**, *25*, 5819–5824, doi:10.1016/j.bmcl.2015.08.039.
825. Cheng, S.-Y.; Wang, S.-K.; Hsieh, M.-K.; Duh, C.-Y. Polyoxygenated Cembrane Diterpenoids from the Soft Coral *Sarcophyton ehrenbergi*. *IJMS* **2015**, *16*, 6140–6152, doi:10.3390/ijms16036140.

826. Huang, C.-Y.; Sung, P.-J.; Uvarani, C.; Su, J.-H.; Lu, M.-C.; Hwang, T.-L.; Dai, C.-F.; Wu, S.-L.; Sheu, J.-H. Glaucumolides A and B, Biscembranoids with New Structural Type from a Cultured Soft Coral *Sarcophyton glaucum*. *Sci Rep* **2015**, *5*, 15624, doi:10.1038/srep15624.
827. Abdel-Lateff, A.; Alarif, W.M.; Ayyad, S.-E.N.; Al-Lihaibi, S.S.; Basaif, S.A. New Cytotoxic Isoprenoid Derivatives from the Red Sea Soft Coral *Sarcophyton glaucum*. *Natural Product Research* **2015**, *29*, 24–30, doi:10.1080/14786419.2014.952637.
828. Nam, N.H.; Tung, P.T.; Ngoc, N.T.; Hanh, T.T.H.; Thao, N.P.; Thanh, N.V.; Cuong, N.X.; Thao, D.T.; Huong, T.T.; Thung, D.C.; et al. Cytotoxic Biscembranoids from the Soft Coral *Sarcophyton pauciplicatum*. *Chem. Pharm. Bull.* **2015**, *63*, 636–640, doi:10.1248/cpb.c15-00273.
829. Al-Footy, K.O.; Alarif, W.M.; Asiri, F.; Aly, M.M.; Ayyad, S.-E.N. Rare Pyrane-Based Cembranoids from the Red Sea Soft Coral *Sarcophyton trocheliophorum* as Potential Antimicrobial–Antitumor Agents. *Med Chem Res* **2015**, *24*, 505–512, doi:10.1007/s00044-014-1147-1.
830. Chen, W.-T.; Li, J.; Wang, J.-R.; Li, X.-W.; Guo, Y.-W. Structural Diversity of Terpenoids in the Soft Coral *Sinularia flexibilis*, Evidenced by a Collection from the South China Sea. *RSC Adv.* **2015**, *5*, 23973–23980, doi:10.1039/C5RA01151E.
831. Lin, W.-J.; Wu, T.-Y.; Su, T.-R.; Wen, Z.-H.; Chen, J.-J.; Fang, L.-S.; Wu, Y.-C.; Sung, P.-J. Terpenoids from the Octocoral *Sinularia gaweli*. *IJMS* **2015**, *16*, 19508–19517, doi:10.3390/ijms160819508.
832. Cheng, S.-Y.; Wang, S.-K.; Chen, P.-W.; Duh, C.-Y. Sandensone A, a Novel Sesquiterpenoid from the Formosan Soft Coral *Sinularia sandensis*. *Bioorganic & Medicinal Chemistry Letters* **2015**, *25*, 2353–2355, doi:10.1016/j.bmcl.2015.04.022.
833. Yin, C.-T.; Wen, Z.-H.; Lan, Y.-H.; Chang, Y.-C.; Wu, Y.-C.; Sung, P.-J. New Anti-Inflammatory Norcembranoids from the Soft Coral *Sinularia numerosa*. *Chem. Pharm. Bull.* **2015**, *63*, 752–756, doi:10.1248/cpb.c15-00414.
834. Kate, A.S.; Richard, K.; Ramanathan, B.; Kerr, R.G. A Halogenated Pseudopterane Diterpene from the Bahamian Octocoral *Pseudopterogorgia acerosa*. *Can. J. Chem.* **2010**, *88*, 318–322, doi:10.1139/V09-168.
835. Tsai, T.-C.; Chen, H.-Y.; Sheu, J.-H.; Chiang, M.Y.; Wen, Z.-H.; Dai, C.-F.; Su, J.-H. Structural Elucidation and Structure–Anti-Inflammatory Activity Relationships of Cembranoids from Cultured Soft Corals *Sinularia sandensis* and *Sinularia Flexibilis*. *J. Agric. Food Chem.* **2015**, *63*, 7211–7218, doi:10.1021/acs.jafc.5b01931.
836. Huang, Q.; Cheng, W.; Long, H.; Liu, H.; van Ofwegen, L.; Lin, W. Subergane-Type Sesquiterpenes from Gorgonian Coral *Subergorgia suberosa* with Antibacterial Activities. *HCA* **2015**, *98*, 1202–1209, doi:10.1002/hlca.201500086.
837. Ueda, K.; Maarisit, W.; Yano, K.; Miyazato, T.; K. Roy, P.; Taira, J. Structures and Bioactivities of Xenicanes from an Okinawan Soft Coral *Xenia* sp. *Heterocycles* **2015**, *91*, 505, doi:10.3987/COM-14-13149.

838. Chen, B.-W.; Uvarani, C.; Huang, C.-Y.; Hwang, T.-L.; Dai, C.-F.; Sheu, J.-H. New Anti-Inflammatory Tocopherol-Derived Metabolites from the Taiwanese Soft Coral *Cladiella hirsuta*. *Bioorganic & Medicinal Chemistry Letters* **2015**, *25*, 92–95, doi:10.1016/j.bmcl.2014.11.002.

839. Lin, W.-Y.; Su, J.-H.; Lu, Y.; Wen, Z.-H.; Dai, C.-F.; Kuo, Y.-H.; Sheu, J.-H. Cytotoxic and Anti-Inflammatory Cembranoids from the Dongsha Atoll Soft Coral *Sarcophyton crassocaule*. *Bioorganic & Medicinal Chemistry* **2010**, *18*, 1936–1941, doi:10.1016/j.bmc.2010.01.036.

840. Cheng, S.-Y.; Wang, S.-K.; Chiou, S.-F.; Hsu, C.-H.; Dai, C.-F.; Chiang, M.Y.; Duh, C.-Y. Cembranoids from the Octocoral *Sarcophyton ehrenbergi*. *J. Nat. Prod.* **2010**, *73*, 197–203, doi:10.1021/np900693r.

841. Su, Y.-D.; Sung, C.-S.; Wen, Z.-H.; Chen, Y.-H.; Chang, Y.-C.; Chen, J.-J.; Fang, L.-S.; Wu, Y.-C.; Sheu, J.-H.; Sung, P.-J. New 9-Hydroxybriarane Diterpenoids from a Gorgonian Coral *Briareum* sp. (Briareidae). *IJMS* **2016**, *17*, 79, doi:10.3390/ijms17010079.

842. Su, Y.-D.; Wen, Z.-H.; Wu, Y.-C.; Fang, L.-S.; Chen, Y.-H.; Chang, Y.-C.; Sheu, J.-H.; Sung, P.-J. Briarenolides M–T, New Briarane Diterpenoids from a Formosan Octocoral *Briareum* sp. *Tetrahedron* **2016**, *72*, 944–951, doi:10.1016/j.tet.2015.12.058.

843. Peng, K.-Y.; Chen, N.-F.; Chen, Z.-C.; Tsui, K.-H.; Wen, Z.-H.; Su, Y.-D.; Chang, Y.-C.; Chen, Y.-H.; Lu, M.-C.; Fang, L.-S.; et al. Cladieunicellins R and S, New Eunicellins from the Formosan Octocoral *Cladiella tuberculosa*. *Tetrahedron Letters* **2016**, *57*, 4239–4242, doi:10.1016/j.tetlet.2016.08.028.

844. Sun, X.-P.; Wang, C.-Y.; Shao, C.-L.; Li, L.; Li, X.-B.; Chen, M.; Qian, P.-Y. Chemical Constituents of the Soft Coral *Sarcophyton infundibuliforme* from the South China Sea. *Natural Product Communications* **2010**, *5*, 1934578X1000500, doi:10.1177/1934578X1000500803.

845. Torres-Mendoza, D.; González, Y.; Gómez-Reyes, J.; Guzmán, H.; López-Perez, J.; Gerwick, W.; Fernandez, P.; Gutiérrez, M. Uprolides N, O and P from the Panamanian Octocoral *Eunicea succinea*. *Molecules* **2016**, *21*, 819, doi:10.3390/molecules21060819.

846. Angulo-Preckler, C.; Genta-Jouve, G.; Mahajan, N.; de la Cruz, M.; de Pedro, N.; Reyes, F.; Iken, K.; Avila, C.; Thomas, O.P. Gersemiols A–C and Eunicellol A, Diterpenoids from the Arctic Soft Coral *Gersemia fruticosa*. *J. Nat. Prod.* **2016**, *79*, 1132–1136, doi:10.1021/acs.jnatprod.6b00040.

847. Abdel-Razik, A.F.; Nassar, M.I.; Elshamy, A.I.; Kubacy, T.M.; Hegazy, M.-E.F.; Ibrahim, N.; Lamer, A.-C.L.; Farrag, A.-R.H. A New Cytotoxic Ceramide from *Heteroxenia ghardaqensis* and Protective Effect of Chloroform Extract against Cadmium Toxicity in Rats. *Arabian Journal of Chemistry* **2016**, *9*, 649–655, doi:10.1016/j.arabjc.2014.11.055.

848. Chang, F.-Y.; Chokkalingam, U.; Tai, C.-J.; Huang, C.-Y.; Wei, W.-C.; Yang, N.-S.; Su, J.-H.; Sung, P.-J.; Sheu, J.-H. New Eunicellin-Derived Diterpenoids from a Taiwanese Soft Coral *Klyxum molle*. *Tetrahedron* **2016**, *72*, 192–198, doi:10.1016/j.tet.2015.11.025.

849. Zhao, M.; Cheng, S.; Yuan, W.; Xi, Y.; Li, X.; Dong, J.; Huang, K.; Gustafson, K.; Yan, P. Cembranoids from a Chinese Collection of the Soft Coral *Lobophytum crassum*. *Marine Drugs* **2016**, *14*, 111, doi:10.3390/md14060111.

850. Roy, P.; Ashimine, R.; Miyazato, H.; Taira, J.; Ueda, K. New Casbane and Cembrane Diterpenoids from an Okinawan Soft Coral, *Lobophytum* sp. *Molecules* **2016**, *21*, 679, doi:10.3390/molecules21050679.
851. Peng, Q.; Liu, F.; Sun, H.; Liao, X.-J.; Feng, M.-R.; Liu, T.-T.; Xu, Z.-X.; Zhang, J.; Xu, S.-H. A New Sesquiterpene from the Gorgonian Coral *Menella* sp. *Natural Product Research* **2016**, *30*, 2299–2304, doi:10.1080/14786419.2016.1166496.
852. Ishii, T.; Kamada, T.; Vairappan, C.S. Three New Cembranoids from the Bornean Soft Coral *Nephthea* sp. *Journal of Asian Natural Products Research* **2016**, *18*, 415–422, doi:10.1080/10286020.2016.1145670.
853. Georgantea, P.; Ioannou, E.; Evain-Bana, E.; Bagrel, D.; Martinet, N.; Vagias, C.; Roussis, V. Sesquiterpenes with Inhibitory Activity against CDC25 Phosphatases from the Soft Coral *Pseudopterogorgia rigida*. *Tetrahedron* **2016**, *72*, 3262–3269, doi:10.1016/j.tet.2016.04.059.
854. Cheng, S.-Y.; Huang, K.-J.; Wang, S.-K.; Wen, Z.-H.; Chen, P.-W.; Duh, C.-Y. Antiviral and Anti-Inflammatory Metabolites from the Soft Coral *Sinularia capillosa*. *J. Nat. Prod.* **2010**, *73*, 771–775, doi:10.1021/np9008078.
855. Kamada, T.; Phan, C.-S.; Tin, H.-S.; Vairappan, C.S.; Muhammad, T.S.T. 16-Hydroxycembra-1,3,7,11-Tetraene, a New Cembrane Diterpene from Malaysian Soft Coral Genus *Sarcophyton*. *Natural Product Communications* **2016**, *11*, 1934578X1601100, doi:10.1177/1934578X1601100810.
856. Zubair, M.; Alarif, W.; Al-Footy, K.; Ph, M.; Ali, M.; Basaif, S.; Al-LIHAIBI, S.; Ayyad, S.-E. New Antimicrobial Biscembrane Hydrocarbon and Cembranoid Diterpenes from the Soft Coral *Sarcophyton trocheliophorum*. *Turk J Chem* 8.
857. Huang, C.-Y.; Tseng, Y.-J.; Chokkalingam, U.; Hwang, T.-L.; Hsu, C.-H.; Dai, C.-F.; Sung, P.-J.; Sheu, J.-H. Bioactive Isoprenoid-Derived Natural Products from a Dongsha Atoll Soft Coral *Sinularia erecta*. *J. Nat. Prod.* **2016**, *79*, 1339–1346, doi:10.1021/acs.jnatprod.5b01142.
858. Chitturi, B.R.; Tatipamula, V.B.; Dokuburra, C.B.; Mangamuri, U.K.; Tuniki, V.R.; Kalivendi, S.V.; Bunce, R.A.; Yenamandra, V. Pambanolides A–C from the South Indian Soft Coral *Sinularia inelegans*. *Tetrahedron* **2016**, *72*, 1933–1940, doi:10.1016/j.tet.2016.02.056.
859. Cheng, S.-Y.; Wang, S.-K.; Ou, Y.-H.; Duh, C.-Y. Mollisolactones A and B, Novel Dinormonoterpenes from the Soft Coral *Sinularia mollis*. *Bioorganic & Medicinal Chemistry Letters* **2016**, *26*, 879–881, doi:10.1016/j.bmcl.2015.12.076.
860. Roy, P.K.; Ashimine, R.; Miyazato, H.; Taira, J.; Ueda, K. Endoperoxy and Hydroperoxy Cadinane-Type Sesquiterpenoids from an Okinawan Soft Coral, *Sinularia* sp. *Arch. Pharm. Res.* **2016**, *39*, 778–784, doi:10.1007/s12272-016-0759-z.
861. Phan, C.-S.; Ng, S.-Y.; Kamada, T.; Vairappan, C.S. Two New Lobane Diterpenes from a Bornean Soft Coral *Sinularia* sp. *Natural Product Communications* **2016**, *11*, 1934578X1601100, doi:10.1177/1934578X1601100708.
862. Miyazato, H.; Taira, J.; Ueda, K. Hydrogen Peroxide Derived from Marine Peroxy Sesquiterpenoids Induces Apoptosis in HCT116 Human Colon Cancer Cells. *Bioorganic & Medicinal Chemistry Letters* **2016**, *26*, 4641–4644, doi:10.1016/j.bmcl.2016.08.057.

863. Xu, M.-W.; Hao, Y.-T. A New Sesquiterpene from the South China Sea Gorgonian Coral *Subergorgia suberosa*. *Natural Product Research* **2016**, *30*, 2402–2406, doi:10.1080/14786419.2016.1190720.
864. Li, Y.; Carbone, M.; Vitale, R.M.; Amodeo, P.; Castelluccio, F.; Sicilia, G.; Mollo, E.; Nappo, M.; Cimino, G.; Guo, Y.-W.; et al. Rare Casbane Diterpenoids from the Hainan Soft Coral *Sinularia depressa*. *J. Nat. Prod.* **2010**, *73*, 133–138, doi:10.1021/np900484k.
865. Gao, Y.; Xiao, W.; Liu, H.-C.; Wang, J.-R.; Yao, L.-G.; Ouyang, P.-K.; Wang, D.-C.; Guo, Y.-W. Clavuridins A and B, Two New Trinor-Guaiane Sesquiterpenes Isolated from the Xisha Soft Coral *Clavularia viridis*. *Chinese Journal of Natural Medicines* **2017**, *15*, 855–859, doi:10.1016/S1875-5364(18)30019-0.
866. Chen, B.-W.; Chao, C.-H.; Su, J.-H.; Huang, C.-Y.; Dai, C.-F.; Wen, Z.-H.; Sheu, J.-H. A Novel Symmetric Sulfur-Containing Biscembranoid from the Formosan Soft Coral *Sinularia flexibilis*. *Tetrahedron Letters* **2010**, *51*, 5764–5766, doi:10.1016/j.tetlet.2010.08.027.
867. Cheng, S.-Y.; Chuang, C.-T.; Wang, S.-K.; Wen, Z.-H.; Chiou, S.-F.; Hsu, C.-H.; Dai, C.-F.; Duh, C.-Y. Antiviral and Anti-Inflammatory Diterpenoids from the Soft Coral *Sinularia gyrosa*. *J. Nat. Prod.* **2010**, *73*, 1184–1187, doi:10.1021/np100185a.
868. Li, M.-J.; Su, Y.-D.; Liao, Z.-J.; Wen, Z.-H.; Su, J.-H.; Wu, Y.-C.; Sung, P.-J. Briarenol B, a New Polyoxygenated Briarane from the Octocoral *Briareum excavatum*. *Natural Product Communications* **2017**, *12*, 1934578X1701200, doi:10.1177/1934578X1701200220.
869. Chen, N.-F.; Su, Y.-D.; Hwang, T.-L.; Liao, Z.-J.; Tsui, K.-H.; Wen, Z.-H.; Wu, Y.-C.; Sung, P.-J. Briarenols C–E, New Polyoxygenated Briaranes from the Octocoral *Briareum EXCAVATUM*. *Molecules* **2017**, *22*, 475, doi:10.3390/molecules22030475.
870. Rahelivao, M.P.; Lübken, T.; Gruner, M.; Kataeva, O.; Ralambondrahety, R.; Andriamanantoanina, H.; Checinski, M.P.; Bauer, I.; Knölker, H.-J. Isolation and Structure Elucidation of Natural Products of Three Soft Corals and a Sponge from the Coast of Madagascar. *Org. Biomol. Chem.* **2017**, *15*, 2593–2608, doi:10.1039/C7OB00191F.
871. Elshamy, A.I.; El-Kashak, W.A.; Abdallah, H.M.I.; Farrag, A.H.; Nassar, M.I. Soft Coral Cespitularia Stolonifera : New Cytotoxic Ceramides and Gastroprotective Activity. *Chinese Journal of Natural Medicines* **2017**, *15*, 105–114, doi:10.1016/S1875-5364(17)30026-2.
872. Cao, F.; Shao, C.-L.; Liu, Y.-F.; Zhu, H.-J.; Wang, C.-Y. Cytotoxic Serrulatane-Type Diterpenoids from the Gorgonian Euplexaura sp. and Their Absolute Configurations by Vibrational Circular Dichroism. *Sci Rep* **2017**, *7*, 12548, doi:10.1038/s41598-017-12841-2.
873. Cheng, W.; Ji, M.; Li, X.; Ren, J.; Yin, F.; van Ofwegen, L.; Yu, S.; Chen, X.; Lin, W. Fragilolides A-Q, Norditerpenoid and Briarane Diterpenoids from the Gorgonian Coral *Junceella fragilis*. *Tetrahedron* **2017**, *73*, 2518–2528, doi:10.1016/j.tet.2017.03.037.
874. Cheng, W.; Li, X.; Yin, F.; van Ofwegen, L.; Lin, W. Halogenated Briarane Diterpenes with Acetyl Migration from the Gorgonian Coral Junceella Fragilis. *Chem. Biodiversity* **2017**, *14*, e1700053, doi:10.1002/cbdv.201700053.

875. Ahmed, A.; Tsai, C.-R.; Huang, C.-Y.; Wang, S.-Y.; Sheu, J.-H. Klyflaccicembranols A–I, New Cembranoids from the Soft Coral *Klyxum flaccidum*. *Marine Drugs* **2017**, *15*, 23, doi:10.3390/md15010023.

876. Cheng, S.-Y.; Chuang, C.-T.; Wen, Z.-H.; Wang, S.-K.; Chiou, S.-F.; Hsu, C.-H.; Dai, C.-F.; Duh, C.-Y. Bioactive Norditerpenoids from the Soft Coral *Sinularia gyrosa*. *Bioorganic & Medicinal Chemistry* **2010**, *18*, 3379–3386, doi:10.1016/j.bmc.2010.04.012.

877. Lai, K.-H.; You, W.-J.; Lin, C.-C.; El-Shazly, M.; Liao, Z.-J.; Su, J.-H. Anti-Inflammatory Cembranoids from the Soft Coral *Lobophytum crassum*. *Marine Drugs* **2017**, *15*, 327, doi:10.3390/md15100327.

878. Ahmed, A.; Teng, W.-T.; Huang, C.-Y.; Dai, C.-F.; Hwang, T.-L.; Sheu, J.-H. Anti-Inflammatory Lobane and Prenyleudesmane Diterpenoids from the Soft Coral *Lobophytum varium*. *Marine Drugs* **2017**, *15*, 300, doi:10.3390/md15100300.

879. Li, P.; Liu, X.; Zhu, H.; Tang, X.; Shi, X.; Liu, Y.; Li, G. Unusual Inner-Salt Guaiazulene Alkaloids and Bis-Sesquiterpene from the South China Sea Gorgonian *Muriceides collaris*. *Sci Rep* **2017**, *7*, 7697, doi:10.1038/s41598-017-08100-z.

880. Lee, Y.-S.; Duh, T.-H.; Siao, S.-S.; Chang, R.-C.; Wang, S.-K.; Duh, C.-Y. New Cytotoxic Terpenoids from Soft Corals *Nephthea chabroli* and *Paralemnalia thyrsoides*. *Marine Drugs* **2017**, *15*, 392, doi:10.3390/md15120392.

881. Hegazy, M.-E.F.; Elshamy, A.I.; Mohamed, T.A.; Hamed, A.R.; Ibrahim, M.A.A.; Ohta, S.; Paré, P.W. Cembrene Diterpenoids with Ether Linkages from *Sarcophyton ehrenbergi*: An Anti-Proliferation and Molecular-Docking Assessment. *Marine Drugs* **2017**, *15*, 192, doi:10.3390/md15060192.

882. Ghandourah, M.A.; Alarif, W.M.; Abdel-Lateff, A.; Al-Footy, K.O.; Halid, M.; Al-Lihaibi, S.S.; Alorfi, H.S. Antiproliferative Effects of Isoprenoids from *Sarcophyton glaucum* on Breast Cancer MCF-7 Cells. *Trop. J. Pharm Res* **2017**, *16*, 501, doi:10.4314/tjpr.v16i3.2.

883. Chao, C.-H.; Li, W.-L.; Huang, C.-Y.; Ahmed, A.F.; Dai, C.-F.; Wu, Y.-C.; Lu, M.-C.; Liaw, C.-C.; Sheu, J.-H. Isoprenoids from the Soft Coral *Sarcophyton glaucum*. *Marine Drugs* **2017**, *15*, 202, doi:10.3390/md15070202.

884. Wang, J.; Su, P.; Gu, Q.; Li, W.D.; Guo, J.L.; Qiao, W.; Feng, D.Q.; Tang, S.A. Antifouling Activity against Bryozoan and Barnacle by Cembrane Diterpenes from the Soft Coral *Sinularia flexibilis*. *International Biodeterioration & Biodegradation* **2017**, *120*, 97–103, doi:10.1016/j.ibiod.2017.02.013.

885. Ye, F.; Zhu, Z.-D.; Chen, J.-S.; Li, J.; Gu, Y.-C.; Zhu, W.-L.; Li, X.-W.; Guo, Y.-W. Xishacorenes A–C, Diterpenes with Bicyclo[3.3.1]Nonane Nucleus from the Xisha Soft Coral *Sinularia polydactyla*. *Org. Lett.* **2017**, *19*, 4183–4186, doi:10.1021/acs.orglett.7b01716.

886. Huang, C.-Y.; Su, J.-H.; Liu, Y.-C.; Wen, Z.-H.; Hsu, C.-H.; Chiang, M.Y.; Sheu, J.-H. Oppositane-Type Sesquiterpenoids from the Formosan Soft Coral *Sinularia leptoclados*. *BCSJ* **2010**, *83*, 678–682, doi:10.1246/bcsj.20090263.

887. Urda, C.; Fernández, R.; Pérez, M.; Rodríguez, J.; Jiménez, C.; Cuevas, C. Protoxenicins A and B, Cytotoxic Long-Chain Acylated Xenicanes from the Soft Coral *Protodendron repens*. *J. Nat. Prod.* **2017**, *80*, 713–719, doi:10.1021/acs.jnatprod.7b00046.

888. Zheng, L.-G.; Chang, Y.-C.; Hu, C.-C.; Wen, Z.-H.; Wu, Y.-C.; Sung, P.-J. Fragilides K and L, New Briaranes from the Gorgonian Coral *Junceella fragilis*. *Molecules* **2018**, *23*, 1510, doi:10.3390/molecules23071510.
889. Gallardo, A.B.; Díaz-Marrero, A.R.; De la Rosa, J.M.; D'Croz, L.; Perdomo, G.; Cózar-Castellano, I.; Darias, J.; Cueto, M. Chloro-Furanocembranolides from *Leptogorgia* sp. Improve Pancreatic Beta-Cell Proliferation. *Marine Drugs* **2018**, *16*, 49, doi:10.3390/md16020049.
890. Ye, F.; Li, J.; Wu, Y.; Zhu, Z.-D.; Mollo, E.; Gavagnin, M.; Gu, Y.-C.; Zhu, W.-L.; Li, X.-W.; Guo, Y.-W. Sarinfacetamides A and B, Nitrogenous Diterpenoids with Tricyclo[6.3.1.0 ^{1,5}]Dodecane Scaffold from the South China Sea Soft Coral *Sarcophyton infundibuliforme*. *Org. Lett.* **2018**, *20*, 2637–2640, doi:10.1021/acs.orglett.8b00842.
891. Shaaban, M.; El-Hagrassi, A.M.; Abdelghani, M.A.; Osman, A.F. Diverse Bioactive Compounds from *Sarcophtyton glaucom* : Structure Elucidation and Cytotoxic Activity Studies. *Zeitschrift für Naturforschung C* **2018**, *73*, 325–334, doi:10.1515/znc-2017-0106.
892. Peng, C.-C.; Huang, C.-Y.; Ahmed, A.; Hwang, T.-L.; Dai, C.-F.; Sheu, J.-H. New Cembranoids and a Biscembranoid Peroxide from the Soft Coral *Sarcophyton cherbonnieri*. *Marine Drugs* **2018**, *16*, 276, doi:10.3390/md16080276.
893. Li, S.; Ye, F.; Zhu, Z.; Huang, H.; Mao, S.; Guo, Y. Cembrane-Type Diterpenoids from the South China Sea Soft Coral Sarcophyton Mililatensis. *Acta Pharmaceutica sinica B* **2018**, *8*, 944–955, doi:10.1016/j.apsb.2018.06.004.
894. Ru, T.; Cai, Y.-S.; Li, H.; Tang, W.; Wang, H.; Guo, Y.-W. Further New Eunicellin-Based Diterpenoids from the Guangxi Weizhou Soft Coral *Cladiella krempfi*. *Fitoterapia* **2018**, *131*, 200–203, doi:10.1016/j.fitote.2018.10.012.
895. Qin, G.-F.; Tang, X.-L.; Sun, Y.-T.; Luo, X.-C.; Zhang, J.; van Ofwegen, L.; Sung, P.-J.; Li, P.-L.; Li, G.-Q. Terpenoids from the Soft Coral *Sinularia* sp. Collected in Yongxing Island. *Marine Drugs* **2018**, *16*, 127, doi:10.3390/md16040127.
896. Kamada, T.; Kang, M.-C.; Phan, C.-S.; Zanil, I.; Jeon, Y.-J.; Vairappan, C. Bioactive Cembranoids from the Soft Coral Genus *Sinularia* sp. in Borneo. *Marine Drugs* **2018**, *16*, 99, doi:10.3390/md16040099.
897. Wu, C.-H.; Chao, C.-H.; Huang, T.-Z.; Huang, C.-Y.; Hwang, T.-L.; Dai, C.-F.; Sheu, J.-H. Cembranoid-Related Metabolites and Biological Activities from the Soft Coral *Sinularia Flexibilis*. *Marine Drugs* **2018**, *16*, 278, doi:10.3390/md16080278.
898. Huong, N.T.; Ngoc, N.T.; Thanh, N.V.; Dang, N.H.; Cuong, N.X.; Nam, N.H.; Thung, D.C.; The, H.V.; Tuan, V.S.; Kiem, P.V.; et al. Eudesmane and Aromadendrane Sesquiterpenoids from the Vietnamese Soft Coral *Sinularia erecta*. *Natural Product Research* **2018**, *32*, 1798–1802, doi:10.1080/14786419.2017.1402326.
899. Chu, M.-J.; Tang, X.-L.; Han, X.; Li, T.; Luo, X.-C.; Jiang, M.-M.; van Ofwegen, L.; Luo, L.-Z.; Zhang, G.; Li, P.-L.; et al. Metabolites from the Paracel Islands Soft Coral *Sinularia* cf. *molesta*. *Marine Drugs* **2018**, *16*, 517, doi:10.3390/md16120517.

900. Ye, F.; Zhu, Z.-D.; Gu, Y.-C.; Li, J.; Zhu, W.-L.; Guo, Y.-W. Further New Diterpenoids as PTP1B Inhibitors from the Xisha Soft Coral *Sinularia polydactyla*. *Marine Drugs* **2018**, *16*, 103, doi:10.3390/md16040103.
901. Xu, J.-H.; Lai, K.-H.; Su, Y.-D.; Chang, Y.-C.; Peng, B.-R.; Backlund, A.; Wen, Z.-H.; Sung, P.-J. Briaviolides K–N, New Briarane-Type Diterpenoids from Cultured Octocoral *Briareum violaceum*. *Marine Drugs* **2018**, *16*, 75, doi:10.3390/md16030075.
902. Abou El-Kassem, L.T.; Hawas, U.W.; El-Desouky, S.K.; Al-Farawati, R. Sesquiterpenes from the Saudi Red Sea: *Litophyton arboreum* with Their Cytotoxic and Antimicrobial Activities. *Zeitschrift für Naturforschung C* **2018**, *73*, 9–14, doi:10.1515/znc-2017-0037.
903. Fernando, I.P.S.; Sanjeewa, K.K.A.; Ann, Y.-S.; Ko, C.; Lee, S.-H.; Lee, W.W.; Jeon, Y.-J. Apoptotic and Antiproliferative Effects of Stigmast-5-En-3-Ol from *Dendronephthya gigantea* on Human Leukemia HL-60 and Human Breast Cancer MCF-7 Cells. *Toxicology in Vitro* **2018**, *52*, 297–305, doi:10.1016/j.tiv.2018.07.007.
904. Wu, Q.; Gao, Y.; Zhang, M.-M.; Sheng, L.; Li, J.; Li, X.-W.; Wang, H.; Guo, Y.-W. New Sesquiterpenoids from the South China Sea Soft Corals *Clavularia Viridis* and *Lemnalia flava*. *Beilstein J. Org. Chem.* **2019**, *15*, 695–702, doi:10.3762/bjoc.15.64.
905. Wang, Q.; Hu, Z.; Luo, X.; Liu, J.; Li, G.; Cao, S.; Liu, Q. Clavukoellians A–F, Highly Rearranged Nardosinane Sesquiterpenoids with Antiangiogenic Activity from *Clavularia koellikeri*. *J. Nat. Prod.* **2019**, *82*, 1331–1337, doi:10.1021/acs.jnatprod.9b00100.
906. Huang, P.-C.; Tseng, C.-C.; Peng, B.-R.; Hu, C.-C.; Lin, N.-C.; Chen, N.-F.; Chen, J.-J.; Wen, Z.-H.; Wu, Y.-C.; Sung, P.-J. Briaviodiols B–E, New Anti-Inflammatory Hydroperoxyfurancembranoids from *Briareum violaceum*. *Tetrahedron* **2019**, *75*, 921–927, doi:10.1016/j.tet.2019.01.010.
907. Huang, P.-C.; Lin, W.-S.; Peng, B.-R.; Chang, Y.-C.; Fang, L.-S.; Li, G.-Q.; Hwang, T.-L.; Wen, Z.-H.; Sung, P.-J. New Furanocembranoids from *Briareum violaceum*. *Marine Drugs* **2019**, *17*, 214, doi:10.3390/md17040214.
908. Tseng; Ahmed; Huang; Tsai; Tai; Orfali; Hwang; Wang; Dai; Sheu Bioactive Capnosanes and Cembranes from the Soft Coral *Klyxum flaccidum*. *Marine Drugs* **2019**, *17*, 461, doi:10.3390/md17080461.
909. Tani, K.; Kamada, T.; Phan, C.-S.; Vairappan, C.S. New Cembrane-Type Diterpenoids from Bornean Soft Coral *Nephthea* sp. with Antifungal Activity against *Lagenidium thermophilum*. *Natural Product Research* **2019**, *33*, 3343–3349, doi:10.1080/14786419.2018.1475387.
910. Bauer, K.; Puyana, M.; Castellanos, L.; Tello, E. New Diterpenes Isolated from the Colombian Caribbean Soft Coral *Pseudoplexaura flagellosa* and Their Cytotoxic Properties. *Rec.Nat.Prod.* **2019**, *13*, 243–253, doi:10.25135/rnp.100.18.07.325.1.
911. Tani, K.; Kamada, T.; Phan, C.-S.; Vairappan, C.S. A New Bioactive Cembranolide Sarcophytonolide V from Bornean Soft Coral Genus *Sarcophyton*. *Natural Product Communications* **2019**, *14*, 1934578X1986837, doi:10.1177/1934578X19868375.
912. Hassan, H.M.; Rateb, M.E.; Hassan, M.H.; Sayed, A.M.; Shabana, S.; Raslan, M.; Amin, E.; Behery, F.A.; Ahmed, O.M.; Bin Muhsinah, A.; et al. New Antiproliferative Cembrane Diterpenes from the Red Sea *Sarcophyton* Species. *Marine Drugs* **2019**, *17*, 411, doi:10.3390/md17070411.

913. Li, G.; Li, H.; Zhang, Q.; Yang, M.; Gu, Y.-C.; Liang, L.-F.; Tang, W.; Guo, Y.-W. Rare Cembranoids from Chinese Soft Coral *Sarcophyton ehrenbergi* : Structural and Stereochemical Studies. *J. Org. Chem.* **2019**, *84*, 5091–5098, doi:10.1021/acs.joc.9b00030.

914. Yang, M.; Li, X.-L.; Wang, J.-R.; Lei, X.; Tang, W.; Li, X.-W.; Sun, H.; Guo, Y.-W. Sarcomililate A, an Unusual Diterpenoid with Tricyclo[11.3.0.0 ^{2,16}]Hexadecane Carbon Skeleton, and Its Potential Biogenetic Precursors from the Hainan Soft Coral *Sarcophyton mililatensis*. *J. Org. Chem.* **2019**, *84*, 2568–2576, doi:10.1021/acs.joc.8b03020.

915. Jiang, C.-S.; Ru, T.; Yao, L.-G.; Miao, Z.-H.; Guo, Y.-W. Four New Cembranoids from the Chinese Soft Coral *Sinularia* sp. and Their Anti-Aβ Aggregation Activities. *Fitoterapia* **2019**, *136*, 104176, doi:10.1016/j.fitote.2019.104176.

916. Wu, Q.; Li, X.-W.; Li, H.; Yao, L.-G.; Tang, W.; Miao, Z.-H.; Wang, H.; Guo, Y.-W. Bioactive Polyoxygenated Cembranoids from a Novel Hainan Chemotype of the Soft Coral *Sinularia flexibilis*. *Bioorganic & Medicinal Chemistry Letters* **2019**, *29*, 185–188, doi:10.1016/j.bmcl.2018.12.004.

917. Phan, C.-S.; Yee, C.-S.; Vairappan, C.S.; Ishii, T.; Kamada, T. Sinulaflexiolide P, A Cembrane-Type Diterpenoid from Bornean Soft Coral *Sinularia flexibilis. Chem Nat Compd* **2019**, *55*, 285–288, doi:10.1007/s10600-019-02668-w.

918. Yang, M.; Li, H.; Zhang, Q.; Wu, Q.-H.; Li, G.; Chen, K.-X.; Guo, Y.-W.; Tang, W.; Li, X.-W. Highly Diverse Cembranoids from the South China Sea Soft Coral *Sinularia scabra* as a New Class of Potential Immunosuppressive Agents. *Bioorganic & Medicinal Chemistry* **2019**, *27*, 3469–3476, doi:10.1016/j.bmc.2019.06.030.

919. Sun, P.; Cai, F.-Y.; Lauro, G.; Tang, H.; Su, L.; Wang, H.-L.; Li, H.H.; Mándi, A.; Kurtán, T.; Riccio, R.; et al. Immunomodulatory Biscembranoids and Assignment of Their Relative and Absolute Configurations: Data Set Modulation in the Density Functional Theory/Nuclear Magnetic Resonance Approach. *J. Nat. Prod.* **2019**, *82*, 1264–1273, doi:10.1021/acs.jnatprod.8b01037.

920. Thomas, S.A.L.; Sanchez, A.; Kee, Y.; Wilson, N.G.; Baker, B.J. Bathyptilones: Terpenoids from an Antarctic Sea Pen, *Anthoptilum grandiflorum* (Verrill, 1879). *Marine Drugs* **2019**, *17*, 513, doi:10.3390/md17090513.

921. Yao, J.-W.; Chi, W.-C.; Xu, J.-H.; Lee, G.-H.; Chiang, M.Y.; Peng, B.-R.; Lin, N.-C.; Hu, C.-C.; Fang, L.-S.; Li, G.-Q.; et al. New Hydroperoxybriarane Diterpenoids from the Octocoral *Briareum violaceum*. *Tetrahedron* **2019**, *75*, 1510–1516, doi:10.1016/j.tet.2019.01.069.

922. Chen, Y.-Y.; Fang, L.-S.; Chen, Y.-H.; Peng, B.-R.; Su, T.-P.; Huynh, T.-H.; Lin, F.-Y.; Hu, C.-C.; Lin, N.-C.; Wen, Z.-H.; et al. New 8-Hydroxybriaranes from the Gorgonian Coral *junceella fragilis* (Ellisellidae). *Marine Drugs* **2019**, *17*, 534, doi:10.3390/md17090534.

923. Su, T.-P.; Yuan, C.-H.; Jhu, Y.-M.; Peng, B.-R.; Wen, Z.-H.; Wu, Y.-J.; Wu, T.-Y.; Liu, H.-W.; Sung, P.-J. Fragilides U–W: New 11,20-Epoxybriaranes from the Sea Whip Gorgonian Coral *Junceella fragilis*. *Marine Drugs* **2019**, *17*, 706, doi:10.3390/md17120706.

924. Chen, Y.-F.; Chen, W.-F.; Wen, Z.-H.; Hwang, T.-L.; Zhang, Z.-J.; Sung, P.-J. New Bioactive Δ11(17)-Furanoeunicellins from an Octocoral *Cladiella* sp. *Phytochemistry Letters* **2019**, *33*, 31–35, doi:10.1016/j.phytol.2019.07.002.

925. Zhang, Z.-J.; Wang, Y.-H.; Chen, S.-R.; Peng, B.-R.; Yang, S.-N.; Hu, C.-C.; Fang, L.-S.; Hwang, T.-L.; Sung, P.-J. Novel Secoeunicellins Produced by an Octocoral *Cladiella* sp. *Tetrahedron Letters* **2019**, *60*, 151300, doi:10.1016/j.tetlet.2019.151300.

926. Li, G.; Li, H.; Tang, W.; Guo, Y.-W.; Li, X.-W. Klyflaccilides A and B, Diterpenoids with 6/5/8/3 Fused Tetracyclic Carbon Skeleton from the Hainan Soft Coral *Klyxum flaccidum*. *Org. Lett.* **2019**, *21*, 5660–5664, doi:10.1021/acs.orglett.9b01998.

927. Phan, C.-S.; Kamada, T.; Ishii, T.; Hamada, T.; Vairappan, C.S. 12- *Epi* -9-Deacetoxyxenicin, New Cytotoxic Diterpenoid from a Bornean Soft Coral, *Xenia* sp. *Natural Product Research* **2019**, *33*, 808–813, doi:10.1080/14786419.2017.1410812.

928. Zhang, J.; Ling, W.; Yang, Z.; Liang, Y.; Zhang, L.; Guo, C.; Wang, K.; Zhong, B.; Xu, S.; Xu, Y. Isolation and Structure-Activity Relationship of Subergorgic Acid and Synthesis of Its Derivatives as Antifouling Agent. *Marine Drugs* **2019**, *17*, 101, doi:10.3390/md17020101.

929. Wu, Y.-J.; Su, T.-R.; Dai, G.-F.; Su, J.-H.; Liu, C.-I. Flaccidoxide-13-Acetate-Induced Apoptosis in Human Bladder Cancer Cells Is through Activation of P38/JNK, Mitochondrial Dysfunction, and Endoplasmic Reticulum Stress Regulated Pathway. *Marine Drugs* **2019**, *17*, 287, doi:10.3390/md17050287.

930. Lin, Y.-S.; Eid Fazary, A.; Chen, C.-H.; Kuo, Y.-H.; Shen, Y.-C. Bioactive Xenicane Diterpenoids from the Taiwanese Soft Coral *Asterospicularia laurae*. *Chemistry & Biodiversity* **2011**, *8*, 1310–1317, doi:10.1002/cbdv.201000173.

931. Gupta, P.; Sharma, U.; Schulz, T.C.; Sherrer, E.S.; McLean, A.B.; Robins, A.J.; West, L.M. Bioactive Diterpenoid Containing a Reversible "Spring-Loaded" (*E,Z*)-Dieneone Michael Acceptor. *Org. Lett.* **2011**, *13*, 3920–3923, doi:10.1021/ol201443k.

932. Joyner, P.M.; Waters, A.L.; Williams, R.B.; Powell, D.R.; Janakiram, N.B.; Rao, C.V.; Cichewicz, R.H. Briarane Diterpenes Diminish *COX-2* Expression in Human Colon Adenocarcinoma Cells. *J. Nat. Prod.* **2011**, *74*, 857–861, doi:10.1021/np100775a.

933. Tai, C.-J.; Su, J.-H.; Huang, M.-S.; Wen, Z.-H.; Dai, C.-F.; Sheu, J.-H. Bioactive Eunicellin-Based Diterpenoids from the Soft Coral *Cladiella krempfi*. *Marine Drugs* **2011**, *9*, 2036–2045, doi:10.3390/md9102036.

934. Tai, C.-Y.; Chen, Y.-H.; Hwang, T.-L.; Fang, L.-S.; Wang, W.-H.; Liu, M.-C.; Su, J.-H.; Wu, Y.-C.; Sung, P.-J. Cladielloides C and D: Novel Eunicellin-Based Diterpenoids from an Indonesian Octocoral *Cladiella* sp. *BCSJ* **2011**, *84*, 531–536, doi:10.1246/bcsj.20100348.

935. Chen, Y.-H.; Tai, C.-Y.; Su, Y.-D.; Chang, Y.-C.; Lu, M.-C.; Weng, C.-F.; Su, J.-H.; Hwang, T.-L.; Wu, Y.-C.; Sung, P.-J. Discovery of New Eunicellins from an Indonesian Octocoral *Cladiella* sp. *Marine Drugs* **2011**, *9*, 934–943, doi:10.3390/md9060934.

936. Chen, Y.-H.; Tai, C.-Y.; Kuo, Y.-H.; Kao, C.-Y.; Li, J.-J.; Hwang, T.-L.; Fang, L.-S.; Wang, W.-H.; Sheu, J.-H.; Sung, P.-J. Cladieunicellins A-E, New Eunicellins from an Indonesian Soft Coral *Cladiella* sp. *Chem. Pharm. Bull.* **2011**, *59*, 353–358, doi:10.1248/cpb.59.353.

937. Li, C.; La, M.-P.; Sun, P.; Kurtan, T.; Mandi, A.; Tang, H.; Liu, B.-S.; Yi, Y.-H.; Li, L.; Zhang, W. Bioactive (3Z,5E)-11,20-Epoxybriara-3,5-Dien-7,18-Olide Diterpenoids from the South China Sea Gorgonian *Dichotella gemmacea*. *Marine Drugs* **2011**, *9*, 1403–1418, doi:10.3390/md9081403.

938. Li, C.; La, M.-P.; Li, L.; Li, X.-B.; Tang, H.; Liu, B.-S.; Krohn, K.; Sun, P.; Yi, Y.-H.; Zhang, W. Bioactive 11,20-Epoxy-3,5(16)-Diene Briarane Diterpenoids from the South China Sea Gorgonian *Dichotella gemmacea*. *J. Nat. Prod.* **2011**, *74*, 1658–1662, doi:10.1021/np200330c.
939. Sun, J.-F.; Huang, H.; Chai, X.-Y.; Yang, X.-W.; Meng, L.; Huang, C.-G.; Zhou, X.-F.; Yang, B.; Hu, J.; Chen, X.-Q.; et al. Dichotellides A–E, Five New Iodine-Containing Briarane Type Diterpenoids from *Dichotella gemmacea*. *Tetrahedron* **2011**, *67*, 1245–1250, doi:10.1016/j.tet.2010.11.087.
940. Wang, C.-Y.; Zhao, J.; Liu, H.-Y.; Shao, C.-L.; Liu, Q.-A.; Liu, Y.; Gu, Y.-C. Two New Eicosanoids with a Unique Isovalerianic Acid Ester Moiety from the South China Sea Gorgonian *Dichotella gemmacea*. *Lipids* **2011**, *46*, 81–85, doi:10.1007/s11745-010-3489-x.
941. Reina, E.; Puentes, C.; Rojas, J.; García, J.; Ramos, F.A.; Castellanos, L.; Aragón, M.; Ospina, L.F. Fuscoside E: A Strong Anti-Inflammatory Diterpene from Caribbean Octocoral *Eunicea fusca*. *Bioorganic & Medicinal Chemistry Letters* **2011**, *21*, 5888–5891, doi:10.1016/j.bmcl.2011.07.092.
942. Mayorga, H.; Urrego, N.F.; Castellanos, L.; Duque, C. Cembradienes from the Caribbean Sea Whip *Eunicea* sp. *Tetrahedron Letters* **2011**, *52*, 2515–2518, doi:10.1016/j.tetlet.2011.03.023.
943. Liaw, C.-C.; Kuo, Y.-H.; Lin, Y.-S.; Hwang, T.-L.; Shen, Y.-C. Frajunolides L–O, Four New 8-Hydroxybriarane Diterpenoids from the *Gorgonian junceella* Fragilis. *Marine Drugs* **2011**, *9*, 1477–1486, doi:10.3390/md9091477.
944. Murthy, Y.L.N.; Mallika, D.; Rajack, A.; Damodar Reddy, G. A New Antifungal Briarane Diterpenoid from the Gorgonian *Junceella juncea* Pallas. *Bioorganic & Medicinal Chemistry Letters* **2011**, *21*, 7522–7525, doi:10.1016/j.bmcl.2011.06.087.
945. Hsu, F.-J.; Chen, B.-W.; Wen, Z.-H.; Huang, C.-Y.; Dai, C.-F.; Su, J.-H.; Wu, Y.-C.; Sheu, J.-H. Klymollins A–H, Bioactive Eunicellin-Based Diterpenoids from the Formosan Soft Coral *Klyxum molle*. *J. Nat. Prod.* **2011**, *74*, 2467–2471, doi:10.1021/np200589n.
946. Chen, B.-W.; Chao, C.-H.; Su, J.-H.; Tsai, C.-W.; Wang, W.-H.; Wen, Z.-H.; Huang, C.-Y.; Sung, P.-J.; Wu, Y.-C.; Sheu, J.-H. Klysimplexins I–T, Eunicellin-Based Diterpenoids from the Cultured Soft Coral *Klyxum simplex*. *Org. Biomol. Chem.* **2011**, *9*, 834–844, doi:10.1039/C0OB00351D.
947. Wu, S.-L.; Su, J.-H.; Lu, Y.; Chen, B.-W.; Huang, C.-Y.; Wen, Z.-H.; Kuo, Y.-H.; Sheu, J.-H. Simplexins J–O, Eunicellin-Based Diterpenoids from a Dongsha Atoll Soft Coral *Klyxum simplex*. *BCSJ* **2011**, *84*, 626–632, doi:10.1246/bcsj.20110013.
948. Lu, Y.; Li, P.-J.; Hung, W.-Y.; Su, J.-H.; Wen, Z.-H.; Hsu, C.-H.; Dai, C.-F.; Chiang, M.Y.; Sheu, J.-H. Nardosinane Sesquiterpenoids from the Formosan Soft Coral *Lemnalia flava*. *J. Nat. Prod.* **2011**, *74*, 169–174, doi:10.1021/np100541a.
949. Minh, C.V.; Kiem, P.V.; Nhiem, N.X.; Cuong, N.X.; Thao, N.P.; Nam, N.H.; Anh, H.L.T.; Thung, D.C.; Thuy, D.T.T.; Kang, H.-K.; et al. Cytotoxic and Antioxidant Activities of Diterpenes and Sterols from the Vietnamese Soft Coral *Lobophytum compactum*. *Bioorganic & Medicinal Chemistry Letters* **2011**, *21*, 2155–2159, doi:10.1016/j.bmcl.2011.01.072.

950. Kao, C.-Y.; Su, J.-H.; Lu, M.-C.; Hwang, T.-L.; Wang, W.-H.; Chen, J.-J.; Sheu, J.-H.; Kuo, Y.-H.; Weng, C.-F.; Fang, L.-S.; et al. Lobocrassins A–E: New Cembrane-Type Diterpenoids from the Soft Coral *Lobophytum crassum*. *Marine Drugs* **2011**, *9*, 1319–1331, doi:10.3390/md9081319.
951. Lin, S.-T.; Wang, S.-K.; Duh, C.-Y. Cembranoids from the Dongsha Atoll Soft Coral *Lobophytum crassum*. *Marine Drugs* **2011**, *9*, 2705–2716, doi:10.3390/md9122705.
952. Lee, N.-L.; Su, J.-H. Tetrahydrofuran Cembranoids from the Cultured Soft Coral *Lobophytum crassum*. *Marine Drugs* **2011**, *9*, 2526–2536, doi:10.3390/md9122526.
953. Li, L.; Sheng, L.; Wang, C.-Y.; Zhou, Y.-B.; Huang, H.; Li, X.-B.; Li, J.; Mollo, E.; Gavagnin, M.; Guo, Y.-W. Diterpenes from the Hainan Soft Coral *Lobophytum cristatum* Tixier-Durivault. *J. Nat. Prod.* **2011**, *74*, 2089–2094, doi:10.1021/np2003325.
954. Cheng, S.-Y.; Chen, P.-W.; Chen, H.-P.; Wang, S.-K.; Duh, C.-Y. New Cembranolides from the Dongsha Atoll Soft Coral *Lobophytum durum*. *Marine Drugs* **2011**, *9*, 1307–1318, doi:10.3390/md9081307.
955. Quang, T.H.; Ha, T.T.; Minh, C.V.; Kiem, P.V.; Huong, H.T.; Ngan, N.T.T.; Nhiem, N.X.; Tung, N.H.; Tai, B.H.; Thuy, D.T.T.; et al. Cytotoxic and Anti-Inflammatory Cembranoids from the Vietnamese Soft Coral *Lobophytum laevigatum*. *Bioorganic & Medicinal Chemistry* **2011**, *19*, 2625–2632, doi:10.1016/j.bmc.2011.03.009.
956. Yan, P.; Deng, Z.; van Ofwegen, L.; Proksch, P.; Lin, W. Lobophytones U - Z1, Biscembranoids from the Chinese Soft Coral *Lobophytum pauciflorum*. *Chemistry & Biodiversity* **2011**, *8*, 1724–1734, doi:10.1002/cbdv.201000244.
957. Kao, S.-Y.; Su, J.-H.; Hwang, T.-L.; Sheu, J.-H.; Wen, Z.-H.; Wu, Y.-C.; Sung, P.-J. Menelloides C and D, New Sesquiterpenoids from the Gorgonian Coral *Menella* sp. *Marine Drugs* **2011**, *9*, 1534–1542, doi:10.3390/md9091534.
958. Kao, S.-Y.; Su, J.-H.; Hwang, T.-L.; Sheu, J.-H.; Su, Y.-D.; Lin, C.-S.; Chang, Y.-C.; Wang, W.-H.; Fang, L.-S.; Sung, P.-J. Discovery of Novel Sesquiterpenoids from a Gorgonian *Menella* sp. *Tetrahedron* **2011**, *67*, 7311–7315, doi:10.1016/j.tet.2011.07.043.
959. Kao, S.-Y.; Chang, Y.-C.; Su, J.-H.; Lu, M.-C.; Chen, Y.-H.; Sheu, J.-H.; Wen, Z.-H.; Wang, W.-H.; Kuo, Y.-H.; Hwang, T.-L.; et al. (-)-Hydroxylindestrenolide, a New Sesquiterpenoid from a Gorgonian Coral *Menella* sp. (Plexauridae). *Chem. Pharm. Bull.* **2011**, *59*, 1048–1050, doi:10.1248/cpb.59.1048.
960. Huang, C.-Y.; Su, J.-H.; Chen, B.-W.; Wen, Z.-H.; Hsu, C.-H.; Dai, C.-F.; Sheu, J.-H.; Sung, P.-J. Nardosinane-Type Sesquiterpenoids from the Formosan Soft Coral *Paralemnalia thyrsoides*. *Marine Drugs* **2011**, *9*, 1543–1553, doi:10.3390/md9091543.
961. Tello, E.; Castellanos, L.; Arevalo-Ferro, C.; Rodríguez, J.; Jiménez, C.; Duque, C. Absolute Stereochemistry of Antifouling Cembranoid Epimers at C-8 from the Caribbean Octocoral *Pseudoplexaura flagellosa*. Revised Structures of Plexaurolones. *Tetrahedron* **2011**, *67*, 9112–9121, doi:10.1016/j.tet.2011.09.094.
962. Chung, H.-M.; Hwang, T.-L.; Chen, Y.-H.; Su, J.-H.; Lu, M.-C.; Chen, J.-J.; Li, J.-J.; Fang, L.-S.; Wang, W.-H.; Sung, P.-J. Rumphellclovane B, a Novel Clovane Analogue from the Gorgonian Coral *Rumphella antipathies*. *BCSJ* **2011**, *84*, 119–121, doi:10.1246/bcsj.20100253.

963. Wang, G.-H.; Huang, H.-C.; Su, J.-H.; Huang, C.-Y.; Hsu, C.-H.; Kuo, Y.-H.; Sheu, J.-H. Crassocolides N–P, Three Cembranoids from the Formosan Soft Coral *Sarcophyton crassocaule*. *Bioorganic & Medicinal Chemistry Letters* **2011**, *21*, 7201–7204, doi:10.1016/j.bmcl.2011.09.052.
964. Chao, C.-H.; Chou, K.-J.; Huang, C.-Y.; Wen, Z.-H.; Hsu, C.-H.; Wu, Y.-C.; Dai, C.-F.; Sheu, J.-H. Bioactive Cembranoids from the Soft Coral *Sinularia crassa*. *Marine Drugs* **2011**, *9*, 1955–1968, doi:10.3390/md9101955.
965. Lai, D.; Li, Y.; Xu, M.; Deng, Z.; van Ofwegen, L.; Qian, P.; Proksch, P.; Lin, W. Sinulariols A–S, 19-Oxygenated Cembranoids from the Chinese Soft Coral *Sinularia rigida*. *Tetrahedron* **2011**, *67*, 6018–6029, doi:10.1016/j.tet.2011.06.029.
966. Fattorusso, E.; Luciano, P.; Putra, M.Y.; Taglialatela-Scafati, O.; Ianaro, A.; Panza, E.; Bavestrello, G.; Cerrano, C. Chloroscabrolides, Chlorinated Norcembranoids from the Indonesian Soft Coral *Sinularia* sp. *Tetrahedron* **2011**, *67*, 7983–7988, doi:10.1016/j.tet.2011.08.024.
967. Lu, M.-C.; Lee, N.-L.; Tseng, S.-W.; Su, J.-H. Sinutriangulin A, a Novel Diterpenoid from the Soft Coral *Sinularia triangula*. *Tetrahedron Letters* **2011**, *52*, 5869–5871, doi:10.1016/j.tetlet.2011.08.161.
968. Limna Mol, V.P.; Raveendran, T.V.; Naik, B.G.; Kunnath, R.J.; Parameswaran, P.S. Calamenenes – Aromatic Bicyclic Sesquiterpenes – from the Indian Gorgonian *Subergorgia reticulata* (Ellis and Solander, 1786). *Natural Product Research* **2011**, *25*, 169–174, doi:10.1080/14786419.2010.495069.
969. Ishigami, S.-T.; Goto, Y.; Inoue, N.; Kawazu, S.-I.; Matsumoto, Y.; Imahara, Y.; Tarumi, M.; Nakai, H.; Fusetani, N.; Nakao, Y. Cristaxenicin A, an Antiprotozoal Xenicane Diterpenoid from the Deep Sea Gorgonian *Acanthoprimnoa cristata*. *J. Org. Chem.* **2012**, *77*, 10962–10966, doi:10.1021/jo302109g.
970. Shen, H.; Liu, X.; Jiang, M.; Luo, G.; Wu, Z.; Chen, B.; Li, J.; Liu, L.; Chen, S. Anti-Inflammatory Cembrane-Type Diterpenoids and Prostaglandins from Soft Coral *Lobophytum sarcophytoides*. *Marine Drugs* **2019**, *17*, 481, doi:10.3390/md17080481.
971. Yan, P.; Ritt, D.A.; Zlotkowski, K.; Bokesch, H.R.; Reinhold, W.C.; Schneekloth, J.S.; Morrison, D.K.; Gustafson, K.R. Macrophilones from the Marine Hydroid *Macrorhynchia philippina* Can Inhibit ERK Cascade Signaling. *J. Nat. Prod.* **2018**, *81*, 1666–1672, doi:10.1021/acs.jnatprod.8b00343.
972. Hansen, K.Ø.; Andersen, J.H.; Bayer, A.; Pandey, S.K.; Lorentzen, M.; Jørgensen, K.B.; Sydnes, M.O.; Guttormsen, Y.; Baumann, M.; Koch, U.; et al. Kinase Chemodiversity from the Arctic: The Breitfussins. *J. Med. Chem.* **2019**, *62*, 10167–10181, doi:10.1021/acs.jmedchem.9b01006.
973. Zlotkowski, K.; Hewitt, W.M.; Yan, P.; Bokesch, H.R.; Peach, M.L.; Nicklaus, M.C.; O'Keefe, B.R.; McMahon, J.B.; Gustafson, K.R.; Schneekloth, J.S. Macrophilone A: Structure Elucidation, Total Synthesis, and Functional Evaluation of a Biologically Active Iminoquinone from the Marine Hydroid *Macrorhynchia philippina*. *Org. Lett.* **2017**, *19*, 1726–1729, doi:10.1021/acs.orglett.7b00496.
974. von Salm, J.L.; Wilson, N.G.; Vesely, B.A.; Kyle, D.E.; Cuce, J.; Baker, B.J. Shagenes A and B, New Tricyclic Sesquiterpenes Produced by an Undescribed Antarctic Octocoral. *Org. Lett.* **2014**, *16*, 2630–2633, doi:10.1021/ol500792x.

975. Maoka, T.; Etoh, T.; Borodina, A.V.; Soldatov, A.A. A Series of 19′-Hexanoyloxyfucoxanthin Derivatives from the Sea Mussel, *Mytilus galloprovincialis*, Grown in the Black Sea, Ukraine. *J Agric Food Chem* **2011**, *59*, 13059–13064, doi:10.1021/jf2035115.
976. Bigot, L.; Zatylny-Gaudin, C.; Rodet, F.; Bernay, B.; Boudry, P.; Favrel, P. Characterization of GnRH-Related Peptides from the Pacific Oyster Crassostrea Gigas. *Peptides* **2012**, *34*, 303–310, doi:10.1016/j.peptides.2012.01.017.
977. Jia, W.; Peng, Q.; Su, L.; Yu, X.; Ma, C.W.; Liang, M.; Yin, X.; Zou, Y.; Huang, Z. Novel Bioactive Peptides from Meretrix Meretrix Protect *Caenorhabditis elegans* against Free Radical-Induced Oxidative Stress through the Stress Response Factor DAF-16/FOXO. *Marine Drugs* **2018**, *16*, 444, doi:10.3390/md16110444.
978. Greco, S.; Gerdol, M.; Edomi, P.; Pallavicini, A. Molecular Diversity of Mytilin-Like Defense Peptides in Mytilidae (Mollusca, Bivalvia). *Antibiotics* **2020**, *9*, 37, doi:10.3390/antibiotics9010037.
979. Lee, M.J.; Oh, R.; Kim, Y.-O.; Nam, B.-H.; Kong, H.J.; Kim, J.-W.; Park, J.Y.; Seo, J.-K.; Kim, D.-G. Mytilin B, an Antimicrobial Peptide from the Hemocyte of the Hard-shelled Mussel, *Mytilus coruscus*: Isolation, Purification, and Characterization. *Journal of Life Science* **2018**, *28*, 1301–1315, doi:10.5352/JLS.2018.28.11.1301.
980. Leoni, G.; De Poli, A.; Mardirossian, M.; Gambato, S.; Florian, F.; Venier, P.; Wilson, D.N.; Tossi, A.; Pallavicini, A.; Gerdol, M. Myticalins: A Novel Multigenic Family of Linear, Cationic Antimicrobial Peptides from Marine Mussels (*Mytilus* Spp.). *Marine Drugs* **2017**, *15*, 261, doi:10.3390/md15080261.
981. Kilcoyne, J.; Twiner, M.J.; McCarron, P.; Crain, S.; Giddings, S.D.; Foley, B.; Rise, F.; Hess, P.; Wilkins, A.L.; Miles, C.O. Structure Elucidation, Relative LC–MS Response and In Vitro Toxicity of Azaspiracids 7–10 Isolated from Mussels (*Mytilus edulis*). *J. Agric. Food Chem.* **2015**, *63*, 5083–5091, doi:10.1021/acs.jafc.5b01320.
982. Joy, M.; Chakraborty, K. Biogenic Antioxidative and Anti-Inflammatory Aryl Polyketides from the Venerid Bivalve Clam *Paphia malabarica*. *Food Chem* **2017**, *237*, 169–180, doi:10.1016/j.foodchem.2017.05.087.
983. Watanabe, M.; Fuda, H.; Jin, S.; Sakurai, T.; Ohkawa, F.; Hui, S.-P.; Takeda, S.; Watanabe, T.; Koike, T.; Chiba, H. Isolation and Characterization of a Phenolic Antioxidant from the Pacific Oyster (*Crassostrea gigas*). *J Agric Food Chem* **2012**, *60*, 830–835, doi:10.1021/jf2038532.
984. Andrianasolo, E.H.; Haramaty, L.; McPhail, K.L.; White, E.; Vetriani, C.; Falkowski, P.; Lutz, R. Bathymodiolamides A and B, Ceramide Derivatives from a Deep-Sea Hydrothermal Vent Invertebrate Mussel, *Bathymodiolus thermophilus*. *J Nat Prod* **2011**, *74*, 842–846, doi:10.1021/np100601w.
985. Sun, Y.; Lin, Y.; Cao, X.; Xiang, L.; Qi, J. Sterols from Mytilidae Show Anti-Aging and Neuroprotective Effects via Anti-Oxidative Activity. *International Journal of Molecular Sciences* **2014**, *15*, 21660–21673, doi:10.3390/ijms151221660.
986. Joy, M.; Chakraborty, K. Previously Undisclosed Bioactive Sterols from Corbiculid Bivalve Clam *Villorita cyprinoides* with Anti-Inflammatory and Antioxidant Potentials. *Steroids* **2018**, *135*, 1–8, doi:10.1016/j.steroids.2018.04.007.

987. Joy, M.; Chakraborty, K. An Unprecedented Antioxidative Isopimarane Norditerpenoid from Bivalve Clam, *Paphia malabarica* with Anti-Cyclooxygenase and Lipoxygenase Potential. *Pharm Biol* **2017**, *55*, 819–824, doi:10.1080/13880209.2017.1280061.
988. Joy, M.; Chakraborty, K. Antioxidative and Anti-Inflammatory Pyranoids and Isochromenyl Analogues from Corbiculid Bivalve Clam, *Villorita cyprinoides*. *Food Chemistry* **2018**, *251*, 125–134, doi:10.1016/j.foodchem.2018.01.059.
989. Chakraborty, K.; Krishnan, S.; Joy, M. Macrocyclic Lactones from Seafood *Amphioctopus neglectus*: Newly Described Natural Leads to Attenuate Angiotensin-II Induced Cardiac Hypertrophy. *Biomedicine & Pharmacotherapy* **2019**, *110*, 155–167, doi:10.1016/j.biopha.2018.11.034.
990. Krishnan, S.; Chakraborty, K.; Joy, M. First Report of Chromenyl Derivatives from Spineless Marine Cuttlefish *Sepiella inermis*: Prospective Antihyperglycemic Agents Attenuate Serine Protease Dipeptidyl Peptidase-IV. *J Food Biochem* **2019**, *43*, e12824, doi:10.1111/jfbc.12824.
991. Neves, J.L.B.; Lin, Z.; Imperial, J.S.; Antunes, A.; Vasconcelos, V.; Olivera, B.M.; Schmidt, E.W. Small Molecules in the Cone Snail Arsenal. *Org. Lett.* **2015**, *17*, 4933–4935, doi:10.1021/acs.orglett.5b02389.
992. Franklin, J.B.; Rajesh, R.P. A Sleep-Inducing Peptide from the Venom of the Indian Cone Snail *Conus araneosus*. *Toxicon* **2015**, *103*, 39–47, doi:10.1016/j.toxicon.2015.06.017.
993. Lebbe, E.K.M.; Peigneur, S.; Maiti, M.; Mille, B.G.; Devi, P.; Ravichandran, S.; Lescrinier, E.; Waelkens, E.; D'Souza, L.; Herdewijn, P.; et al. Discovery of a New Subclass of α-Conotoxins in the Venom of *Conus australis*. *Toxicon* **2014**, *91*, 145–154, doi:10.1016/j.toxicon.2014.08.074.
994. Nguyen, B.; Le Caer, J.-P.; Aráoz, R.; Thai, R.; Lamthanh, H.; Benoit, E.; Molgó, J. Isolation, Purification and Functional Characterization of Alpha-BnIA from *Conus bandanus* Venom. *Toxicon* **2014**, *91*, 155–163, doi:10.1016/j.toxicon.2014.10.006.
995. Favreau, P.; Benoit, E.; Hocking, H.G.; Carlier, L.; D' hoedt, D.; Leipold, E.; Markgraf, R.; Schlumberger, S.; Córdova, M.A.; Gaertner, H.; et al. A Novel M-Conopeptide, CnIIIC, Exerts Potent and Preferential Inhibition of NaV1.2/1.4 Channels and Blocks Neuronal Nicotinic Acetylcholine Receptors. *Br J Pharmacol* **2012**, *166*, 1654–1668, doi:10.1111/j.1476-5381.2012.01837.x.
996. Rajesh, R.P. Novel M-Superfamily and T-Superfamily Conotoxins and Contryphans from the Vermivorous Snail *Conus figulinus*. *J Pept Sci* **2015**, *21*, 29–39, doi:10.1002/psc.2715.
997. Ye, M.; Khoo, K.K.; Xu, S.; Zhou, M.; Boonyalai, N.; Perugini, M.A.; Shao, X.; Chi, C.; Galea, C.A.; Wang, C.; et al. A Helical Conotoxin from *Conus imperialis* Has a Novel Cysteine Framework and Defines a New Superfamily. *J Biol Chem* **2012**, *287*, 14973–14983, doi:10.1074/jbc.M111.334615.
998. Jimenez, E.C.; Olivera, B.M. Divergent M- and O-Superfamily Peptides from Venom of Fish-Hunting *Conus parius*. *Peptides* **2010**, *31*, 1678–1683, doi:10.1016/j.peptides.2010.05.020.

999. Franco, A.; Kompella, S.N.; Akondi, K.B.; Melaun, C.; Daly, N.L.; Luetje, C.W.; Alewood, P.F.; Craik, D.J.; Adams, D.J.; Marí, F. RegIIA: An A4/7-Conotoxin from the Venom of *Conus regius* That Potently Blocks A3β4 NAChRs. *Biochem Pharmacol* **2012**, *83*, 419–426, doi:10.1016/j.bcp.2011.11.006.

1000. Campos-Lira, E.; Carrillo, E.; Aguilar, M.B.; Gajewiak, J.; Gómez-Lagunas, F.; López-Vera, E. Conorfamide-Sr3, a Structurally Novel Specific Inhibitor of the Shaker K+ Channel. *Toxicon* **2017**, *138*, 53–58, doi:10.1016/j.toxicon.2017.07.024.

1001. Wang, S.; Du, T.; Liu, Z.; Wang, S.; Wu, Y.; Ding, J.; Jiang, L.; Dai, Q. Characterization of a T-Superfamily Conotoxin TxVC from *Conus textile* That Selectively Targets Neuronal NAChR Subtypes. *Biochem Biophys Res Commun* **2014**, *454*, 151–156, doi:10.1016/j.bbrc.2014.10.055.

1002. Wu, Y.; Wu, X.; Yu, J.; Zhu, X.; Zhangsun, D.; Luo, S. Influence of Disulfide Connectivity on Structure and Bioactivity of α-Conotoxin TxIA. *Molecules* **2014**, *19*, 966–979, doi:10.3390/molecules19010966.

1003. Sonti, R.; Rao, K.N.S.; Chidanand, S.; Gowd, K.H.; Raghothama, S.; Balaram, P. Conformational Analysis of a 20-Membered Cyclic Peptide Disulfide from *Conus virgo* with a WPW Segment: Evidence for an Aromatic–Proline Sandwich. *Chemistry – A European Journal* **2014**, *20*, 5075–5086, doi:10.1002/chem.201303687.

1004. Dolashka, P.; Dolashki, A.; Beeumen, J.V.; Floetenmeyer, M.; Velkova, L.; Stevanovic, S.; Voelter, W. Antimicrobial Activity of Molluscan Hemocyanins from *Helix* and *Rapana* Snails. *Current Pharmaceutical Biotechnology* **2016**, *17*, 263–270.

1005. Lee, Y.-J.; Han, S.; Kim, S.H.; Lee, H.-S.; Shin, H.J.; Lee, J.S.; Lee, J. Three New Cytotoxic Steroidal Glycosides Isolated from *Conus pulicarius* Collected in Kosrae, Micronesia. *Mar Drugs* **2017**, *15*, 379, doi:10.3390/md15120379.

1006. Carbone, M.; Li, Y.; Irace, C.; Mollo, E.; Castelluccio, F.; Di Pascale, A.; Cimino, G.; Santamaria, R.; Guo, Y.-W.; Gavagnin, M. Structure and Cytotoxicity of Phidianidines A and B: First Finding of 1,2,4-Oxadiazole System in a Marine Natural Product. *Org. Lett.* **2011**, *13*, 2516–2519, doi:10.1021/ol200234r.

1007. Vitale, R.M.; Gatti, M.; Carbone, M.; Barbieri, F.; Felicità, V.; Gavagnin, M.; Florio, T.; Amodeo, P. Minimalist Hybrid Ligand/Receptor-Based Pharmacophore Model for CXCR4 Applied to a Small-Library of Marine Natural Products Led to the Identification of Phidianidine A as a New CXCR4 Ligand Exhibiting Antagonist Activity. *ACS Chem. Biol.* **2013**, *8*, 2762–2770, doi:10.1021/cb400521b.

1008. Liu, J.; Li, H.; Chen, K.-X.; Zuo, J.-P.; Guo, Y.-W.; Tang, W.; Li, X.-W. Design and Synthesis of Marine Phidianidine Derivatives as Potential Immunosuppressive Agents. *J. Med. Chem.* **2018**, *61*, 11298–11308, doi:10.1021/acs.jmedchem.8b01430.

1009. Labriere, C.; Elumalai, V.; Staffansson, J.; Cervin, G.; Le Norcy, T.; Denardou, H.; Réhel, K.; Moodie, L.W.K.; Hellio, C.; Pavia, H.; et al. Phidianidine A and Synthetic Analogues as Naturally Inspired Marine Antifoulants. *J. Nat. Prod.* **2020**, *83*, 3413–3423, doi:10.1021/acs.jnatprod.0c00881.

1010. Tan, K.C.; Wakimoto, T.; Takada, K.; Ohtsuki, T.; Uchiyama, N.; Goda, Y.; Abe, I. Cycloforskamide, a Cytotoxic Macrocyclic Peptide from the Sea Slug *Pleurobranchus forskalii*. *J Nat Prod* **2013**, *76*, 1388–1391, doi:10.1021/np400404r.

1011. Carbone, M.; Irace, C.; Costagliola, F.; Castelluccio, F.; Villani, G.; Calado, G.; Padula, V.; Cimino, G.; Lucas Cervera, J.; Santamaria, R.; et al. A New Cytotoxic Tambjamine Alkaloid from the Azorean Nudibranch *Tambja ceutae*. *Bioorg Med Chem Lett* **2010**, *20*, 2668–2670, doi:10.1016/j.bmcl.2010.02.020.
1012. Kamio, M.; Kicklighter, C.E.; Nguyen, L.; Germann, M.W.; Derby, C.D. Isolation and Structural Elucidation of Novel Mycosporine-Like Amino Acids as Alarm Cues in the Defensive Ink Secretion of the Sea Hare *Aplysia californica*. *Helvetica Chimica Acta* **2011**, *94*, 1012–1018, doi:10.1002/hlca.201100117.
1013. Ciavatta, M.L.; Devi, P.; Carbone, M.; Mathieu, V.; Kiss, R.; Casapullo, A.; Gavagnin, M. Kahalalide F Analogues from the Mucous Secretion of Indian Sacoglossan Mollusc *Elysia ornata*. *Tetrahedron* **2016**, *72*, 625–631, doi:10.1016/j.tet.2015.12.003.
1014. Manzo, E.; Carbone, M.; Mollo, E.; Irace, C.; Di Pascale, A.; Li, Y.; Ciavatta, M.L.; Cimino, G.; Guo, Y.-W.; Gavagnin, M. Structure and Synthesis of a Unique Isonitrile Lipid Isolated from the Marine Mollusk *Actinocyclus papillatus*. *Org. Lett.* **2011**, *13*, 1897–1899, doi:10.1021/ol200377w.
1015. Hanaki, Y.; Kikumori, M.; Ueno, S.; Tokuda, H.; Suzuki, N.; Irie, K. Structure–Activity Studies at Position 27 of Aplog-1, a Simplified Analog of Debromoaplysiatoxin with Anti-Proliferative Activity. *Tetrahedron* **2013**, *69*, 7636–7645, doi:10.1016/j.tet.2013.02.008.
1016. Nuzzo, G.; Ciavatta, M.L.; Kiss, R.; Mathieu, V.; Leclercqz, H.; Manzo, E.; Villani, G.; Mollo, E.; Lefranc, F.; D'Souza, L.; et al. Chemistry of the Nudibranch Aldisa Andersoni: Structure and Biological Activity of Phorbazole Metabolites. *Mar Drugs* **2012**, *10*, 1799–1811, doi:10.3390/md10081799.
1017. Kita, M.; Hirayama, Y.; Yamagishi, K.; Yoneda, K.; Fujisawa, R.; Kigoshi, H. Interactions of the Antitumor Macrolide Aplyronine A with Actin and Actin-Related Proteins Established by Its Versatile Photoaffinity Derivatives. *J Am Chem Soc* **2012**, *134*, 20314–20317, doi:10.1021/ja310495p.
1018. Ojika, M.; Kigoshi, H.; Suenaga, K.; Imamura, Y.; Yoshikawa, K.; Ishigaki, T.; Sakakura, A.; Mutou, T.; Yamada, K. Aplyronines D–H from the Sea Hare *Aplysia kurodai*: Isolation, Structures, and Cytotoxicity. *Tetrahedron* **2012**, *68*, 982–987, doi:10.1016/j.tet.2011.11.095.
1019. Yamada, K.; Ojika, M.; Kigoshi, H.; Suenaga, K. Cytotoxic Substances from Two Species of Japanese Sea Hares: Chemistry and Bioactivity. *Proc Jpn Acad Ser B Phys Biol Sci* **2010**, *86*, 176–189, doi:10.2183/pjab.86.176.
1020. Carbone, M.; Ciavatta, M.L.; Wang, J.-R.; Cirillo, I.; Mathieu, V.; Kiss, R.; Mollo, E.; Guo, Y.-W.; Gavagnin, M. Extending the Record of Bis-γ-Pyrone Polypropionates from Marine Pulmonate Mollusks. *Journal of Natural Products* **2013**, *76*, 2065–2073, doi:10.1021/np400483c.
1021. Zhou, Z.-F.; Li, X.-L.; Yao, L.-G.; Li, J.; Gavagnin, M.; Guo, Y.-W. Marine Bis-γ-Pyrone Polypropionates of Onchidione Family and Their Effects on the XBP1 Gene Expression. *Bioorganic & Medicinal Chemistry Letters* **2018**, *28*, 1093–1096, doi:10.1016/j.bmcl.2018.02.010.
1022. Cutignano, A.; Avila, C.; Rosica, A.; Romano, G.; Laratta, B.; Domenech-Coll, A.; Cimino, G.; Mollo, E.; Fontana, A. Biosynthesis and Cellular Localization of Functional Polyketides in the Gastropod Mollusc *Scaphander lignarius*. *ChemBioChem* **2012**, *13*, 1759–1766.
1023. Llorach-Pares, L.; Rodriguez-Urgelles, E.; Nonell-Canals, A.; Alberch, J.; Avila, C.; Sanchez-Martinez, M.; Giralt, A. Meridianins and Lignarenone B as Potential GSK3β Inhibitors and Inductors of Structural Neuronal Plasticity. *Biomolecules* **2020**, *10*, doi:10.3390/biom10040639.

1024. Kawamura, A.; Kita, M.; Kigoshi, H. Aplysiasecosterol A: A 9,11-Secosteroid with an Unprecedented Tricyclic γ-Diketone Structure from the Sea Hare *Aplysia kurodai. Angewandte Chemie International Edition* **2015**, *54*, 7073–7076, doi:10.1002/anie.201501749.

1025. Clark, K.E.; Capper, A.; Togna, G.D.; Paul, V.J.; Romero, L.I.; Johns, T.; Cubilla-Rios, L.; Capson, T.L. Ecology- and Bioassay-Guided Drug Discovery for Treatments of Tropical Parasitic Disease: 5α,8α-Epidioxycholest-6-En-3β-Ol Isolated from the Mollusk *Dolabrifera dolabrifera* Shows Significant Activity against Leishmania Donovani. *Natural Product Communications* **2013**, *8*, 1934578X1300801109, doi:10.1177/1934578X1300801109.

1026. Carbone, M.; Ciavatta, M.L.; Mathieu, V.; Ingels, A.; Kiss, R.; Pascale, P.; Mollo, E.; Ungur, N.; Guo, Y.-W.; Gavagnin, M. Marine Terpenoid Diacylguanidines: Structure, Synthesis, and Biological Evaluation of Naturally Occurring Actinofide and Synthetic Analogues. *J. Nat. Prod.* **2017**, *80*, 1339–1346, doi:10.1021/acs.jnatprod.6b00941.

1027. Jiménez-Romero, C.; Mayer, A.M.S.; Rodríguez, A.D. Dactyloditerpenol Acetate, a New Prenylbisabolane-Type Diterpene from *Aplysia dactylomela* with Significant in Vitro Anti-Neuroinflammatory Activity. *Bioorg Med Chem Lett* **2014**, *24*, 344–348, doi:10.1016/j.bmcl.2013.11.008.

1028. Vera, B.; Rodríguez, A.D.; La Clair, J.J. Aplysqualenol A Binds to the Light Chain of Dynein Type 1 (DYNLL1). *Angewandte Chemie International Edition* **2011**, *50*, 8134–8138, doi:10.1002/anie.201102546.

1029. Hegazy, M.-E.F.; Moustfa, A.Y.; Mohamed, A.E.-H.H.; Alhammady, M.A.; Elbehairi, S.E.I.; Ohta, S.; Paré, P.W. New Cytotoxic Halogenated Sesquiterpenes from the Egyptian Sea Hare, *Aplysia oculifera*. *Tetrahedron Letters* **2014**, *55*, 1711–1714, doi:10.1016/j.tetlet.2014.01.096.

1030. Maschek, J.A.; Mevers, E.; Diyabalanage, T.; Chen, L.; Ren, Y.; McClintock, J.B.; Amsler, C.D.; Wu, J.; Baker, B.J. Palmadorin Chemodiversity from the Antarctic Nudibranch *Austrodoris kerguelenensis* and Inhibition of Jak2/STAT5-Dependent HEL Leukemia Cells. *Tetrahedron* **2012**, *68*, 9095–9104, doi:10.1016/j.tet.2012.08.045.

1031. Daoust, J.; Fontana, A.; Merchant, C.E.; de Voogd, N.J.; Patrick, B.O.; Kieffer, T.J.; Andersen, R.J. Ansellone A, a Sesterterpenoid Isolated from the Nudibranch *Cadlina luteromarginata* and the Sponge *Phorbas* sp., Activates the CAMP Signaling Pathway. *Org. Lett.* **2010**, *12*, 3208–3211, doi:10.1021/ol101151f.

1032. Hirayama, Y.; Katavic, P.L.; White, A.M.; Pierens, G.K.; Lambert, L.K.; Winters, A.E.; Kigoshi, H.; Kita, M.; Garson, M.J.; Hirayama, Y.; et al. New Cytotoxic Norditerpenes from the Australian Nudibranchs *Goniobranchus splendidus* and *Goniobranchus daphne*. *Aust. J. Chem.* **2015**, *69*, 136–144, doi:10.1071/CH15203.

1033. Mudianta, I.W.; Challinor, V.L.; Winters, A.E.; Cheney, K.L.; De Voss, J.J.; Garson, M.J. Synthesis and Determination of the Absolute Configuration of (-)-(5R,6Z)-Dendrolasin-5-Acetate from the Nudibranch *Hypselodoris jacksoni*. *Beilstein J Org Chem* **2013**, *9*, 2925–2933, doi:10.3762/bjoc.9.329.

1034. Jaisamut, S.; Prabpai, S.; Tancharoen, C.; Yuenyongsawad, S.; Hannongbua, S.; Kongsaeree, P.; Plubrukarn, A. Bridged Tricyclic Sesquiterpenes from the Tubercle Nudibranch *Phyllidia coelestis* Bergh. *J Nat Prod* **2013**, *76*, 2158–2161, doi:10.1021/np4007074.

1035. White, A.M.; Pierens, G.K.; Skinner-Adams, T.; Andrews, K.T.; Bernhardt, P.V.; Krenske, E.H.; Mollo, E.; Garson, M.J. Antimalarial Isocyano and Isothiocyanato Sesquiterpenes with Tri- and Bicyclic Skeletons from the Nudibranch *Phyllidia ocellata*. *J. Nat. Prod.* **2015**, *78*, 1422–1427, doi:10.1021/acs.jnatprod.5b00354.
1036. White, A.M.; Dao, K.; Vrubliauskas, D.; Könst, Z.A.; Pierens, G.K.; Mándi, A.; Andrews, K.T.; Skinner-Adams, T.S.; Clarke, M.E.; Narbutas, P.T.; et al. Catalyst-Controlled Stereoselective Synthesis Secures the Structure of the Antimalarial Isocyanoterpene Pustulosaisonitrile-1. *J. Org. Chem.* **2017**, *82*, 13313–13323, doi:10.1021/acs.joc.7b02421.
1037. Kawashima, H.; Ohnishi, M.; Ogawa, S. Distribution of Unusual Cholesterol Precursors, 4-Methyl- and 4, 4-Dimethylsterols with Δ8 Unsaturation, in Gonads of Marine Archaeogastropods. *J Oleo Sci* **2013**, *62*, 465–470, doi:10.5650/jos.62.465.
1038. Huong, P.T.T.; Huong, P.T.M.; Dang, N.H.; Thanh, N.V.; Cuong, N.X.; Nam, N.H.; Kiem, P.V.; Minh, C.V. Cytotoxic Constituents of the Vietnamese Sea Snail *Monodonta labio* (Linnaeus, 1758). *Letters in Organic Chemistry* **2017**, *14*, 310–314.
1039. Chand, S.; Karuso, P. Isolation and Total Synthesis of Two Novel Metabolites from the Fissurellid Mollusc *Scutus antipodes*. *Tetrahedron Letters* **2017**, *58*, 1020–1023, doi:10.1016/j.tetlet.2017.01.096.
1040. Du, L.; Li, Z.-J.; Xu, J.; Wang, J.-F.; Xue, Y.; Xue, C.-H.; Takahashi, K.; Wang, Y.-M. The Anti-Tumor Activities of Cerebrosides Derived from Sea Cucumber Acaudina Molpadioides and Starfish *Asterias amurensis* in Vitro and in Vivo. *J Oleo Sci* **2012**, *61*, 321–330, doi:10.5650/jos.61.321.
1041. Yamagishi, M.; Hosoda-Yabe, R.; Tamai, H.; Konishi, M.; Imamura, A.; Ishida, H.; Yabe, T.; Ando, H.; Kiso, M. Structure-Activity Relationship Study of the Neuritogenic Potential of the Glycan of Starfish Ganglioside LLG-3 (‡). *Mar Drugs* **2015**, *13*, 7250–7274, doi:10.3390/md13127062.
1042. Farokhi, F.; Wielgosz-Collin, G.; Clement, M.; Kornprobst, J.-M.; Barnathan, G. Cytotoxicity on Human Cancer Cells of Ophidiacerebrosides Isolated from the African Starfish *Narcissia canariensis*. *Mar Drugs* **2010**, *8*, 2988–2998, doi:10.3390/md8122988.
1043. Kim, C.-H.; Go, H.-J.; Oh, H.Y.; Park, J.B.; Lee, T.K.; Seo, J.-K.; Elphick, M.R.; Park, N.G. Identification of a Novel Antimicrobial Peptide from the Sea Star *Patiria pectinifera*. *Dev Comp Immunol* **2018**, *86*, 203–213, doi:10.1016/j.dci.2018.05.002.
1044. Kang, J.-X.; Kang, Y.-F.; Han, H. Three New Cytotoxic Polyhydroxysteroidal Glycosides from Starfish *Craspidaster hesperus*. *Mar Drugs* **2016**, *14*, E189, doi:10.3390/md14100189.
1045. Lu, Y.; Li, H.; Wang, M.; Liu, Y.; Feng, Y.; Liu, K.; Tang, H. Cytotoxic Polyhydroxysteroidal Glycosides from Starfish *Culcita novaeguineae*. *Mar Drugs* **2018**, *16*, E92, doi:10.3390/md16030092.

1046. Malyarenko, T.V.; Ivanchina, N.V.; Malyarenko, O.S.; Kalinovsky, A.I.; Dmitrenok, P.S.; Evtushenko, E.V.; Minh, C.V.; Kicha, A.A. Two New Steroidal Monoglycosides, Anthenosides A_1 and A_2, and Revision of the Structure of Known Anthenoside A with Unusual Monosaccharide Residue from the Starfish *Anthenea aspera*. *Molecules* **2018**, *23*, E1077, doi:10.3390/molecules23051077.

1047. Peng, Y.; Zheng, J.; Huang, R.; Wang, Y.; Xu, T.; Zhou, X.; Liu, Q.; Zeng, F.; Ju, H.; Yang, X.; et al. Polyhydroxy Steroids and Saponins from China Sea Starfish *Asterina pectinifera* and Their Biological Activities. *Chem Pharm Bull (Tokyo)* **2010**, *58*, 856–858, doi:10.1248/cpb.58.856.

1048. Malyarenko, T.V.; Kicha, A.A.; Ivanchina, N.V.; Kalinovsky, A.I.; Dmitrenok, P.S.; Ermakova, S.P.; Stonik, V.A. Cariniferosides A-F and Other Steroidal Biglycosides from the Starfish *Asteropsis carinifera*. *Steroids* **2011**, *76*, 1280–1287, doi:10.1016/j.steroids.2011.06.006.

1049. Vien, L.T.; Hanh, T.T.H.; Hong, P.T.; Thanh, N.V.; Huong, T.T.; Cuong, N.X.; Nam, N.H.; Thung, D.C.; Kiem, P.V.; Minh, C.V. Polar Steroid Derivatives from the Vietnamese Starfish *Astropecten polyacanthus*. *Nat Prod Res* **2018**, *32*, 54–59, doi:10.1080/14786419.2017.1329733.

1050. Malyarenko, T.V.; Kicha, A.A.; Malyarenko, O.S.; Zakharenko, V.M.; Kotlyarov, I.P.; Kalinovsky, A.I.; Popov, R.S.; Svetashev, V.I.; Ivanchina, N.V. New Conjugates of Polyhydroxysteroids with Long-Chain Fatty Acids from the Deep-Water Far Eastern Starfish *Ceramaster patagonicus* and Their Anticancer Activity. *Mar Drugs* **2020**, *18*, E260, doi:10.3390/md18050260.

1051. Ivanchina, N.V.; Kicha, A.A.; Malyarenko, T.V.; Ermolaeva, S.D.; Yurchenko, E.A.; Pislyagin, E.A.; Van Minh, C.; Dmitrenok, P.S. Granulatosides D, E and Other Polar Steroid Compounds from the Starfish *Choriaster granulatus*. Their Immunomodulatory Activity and Cytotoxicity. *Nat Prod Res* **2019**, *33*, 2623–2630, doi:10.1080/14786419.2018.1463223.

1052. Ngoan, B.T.; Hanh, T.T.H.; Vien, L.T.; Diep, C.N.; Thao, N.P.; Thao, D.T.; Thanh, N.V.; Cuong, N.X.; Nam, N.H.; Thung, D.C.; et al. Asterosaponins and Glycosylated Polyhydroxysteroids from the Starfish *Culcita novaeguineae* and Their Cytotoxic Activities. *J Asian Nat Prod Res* **2015**, *17*, 1010–1017, doi:10.1080/10286020.2015.1041930.

1053. Malyarenko, O.S.; Dyshlovoy, S.A.; Kicha, A.A.; Ivanchina, N.V.; Malyarenko, T.V.; Carsten, B.; Gunhild, von A.; Stonik, V.A.; Ermakova, S.P. The Inhibitory Activity of Luzonicosides from the Starfish *Echinaster luzonicus* against Human Melanoma Cells. *Mar Drugs* **2017**, *15*, E227, doi:10.3390/md15070227.

1054. Kicha, A.A.; Ha, D.T.; Ivanchina, N.V.; Malyarenko, T.V.; Kalinovsky, A.I.; Dmitrenok, P.S.; Ermakova, S.P.; Malyarenko, O.S.; Hung, N.A.; Thuy, T.T.T.; et al. Six New Polyhydroxysteroidal Glycosides, Anthenosides S1 - S6, from the Starfish *Anthenea sibogae*. *Chem Biodivers* **2018**, *15*, e1700553, doi:10.1002/cbdv.201700553.

1055. Levina, E.V.; Kalinovsky, A.I.; Dmitrenok, P.S.; Martyyas, E.A.; Stonik, V.A. Two New Steroidal Saponins, Hylodoside A and Novaeguinoside Y, from the Starfish *Leptasterias hylodes reticulata* and *Culcita novaeguineae* (Juvenile). *Natural Product Communications* **2010**, *5*, 1934578X1000501107, doi:10.1177/1934578X1000501107.

1056. Vien, L.T.; Ngoan, B.T.; Hanh, T.T.H.; Vinh, L.B.; Thung, D.C.; Thao, D.T.; Thanh, N.V.; Cuong, N.X.; Nam, N.H.; Kiem, P.V.; et al. Steroid Glycosides from the Starfish *Pentaceraster gracilis*. *J Asian Nat Prod Res* **2017**, *19*, 474–480, doi:10.1080/10286020.2016.1235038.
1057. Vien, L.T.; Hanh, T.T.H.; Quang, T.H.; Cuong, N.X.; Thao, D.T.; Nam, N.H.; Thung, D.C.; Kiem, P.V.; Minh, C.V. Polyhydroxylated Steroid Derivatives from the Starfish *Pentaceraster regulus*. *Nat Prod Res* **2020**, 1–7, doi:10.1080/14786419.2020.1826478.
1058. Kicha, A.A.; Dinh, T.H.; Ivanchina, N.V.; Malyarenko, T.V.; Kalinovsky, A.I.; Popov, R.S.; Ermakova, S.P.; Tran, T.T.T.; Doan, L.P. Three New Steroid Biglycosides, Plancisides A, B, and C, from the Starfish *Acanthaster planci*. *Nat Prod Commun* **2014**, *9*, 1269–1274.
1059. Ha, D.T.; Kicha, A.A.; Kalinovsky, A.I.; Malyarenko, T.V.; Popov, R.S.; Malyarenko, O.S.; Ermakova, S.P.; Thuy, T.T.T.; Long, P.Q.; Ivanchina, N.V. Asterosaponins from the Tropical Starfish *Acanthaster planci* and Their Cytotoxic and Anticancer Activities in Vitro. *Natural Product Research* **2021**, *35*, 548–555, doi:10.1080/14786419.2019.1585845.
1060. Lee, C.-C.; Hsieh, H.J.; Hsieh, C.-H.; Hwang, D.-F. Plancitoxin I from the Venom of Crown-of-Thorns Starfish (*Acanthaster planci*) Induces Oxidative and Endoplasmic Reticulum Stress Associated Cytotoxicity in A375.S2 Cells. *Exp Mol Pathol* **2015**, *99*, 7–15, doi:10.1016/j.yexmp.2015.05.001.
1061. Lee, C.-C.; Hsieh, H.J.; Hwang, D.-F. Cytotoxic and Apoptotic Activities of the Plancitoxin I from the Venom of Crown-of-Thorns Starfish (*Acanthaster planci*) on A375.S2 Cells. *J Appl Toxicol* **2015**, *35*, 407–417, doi:10.1002/jat.3034.
1062. Malyarenko, T.V.; Kicha, A.A.; Ivanchina, N.V.; Kalinovsky, A.I.; Popov, R.S.; Vishchuk, O.S.; Stonik, V.A. Asterosaponins from the Far Eastern Starfish *Leptasterias ochotensis* and Their Anticancer Activity. *Steroids* **2014**, *87*, 119–127, doi:10.1016/j.steroids.2014.05.027.
1063. Popov, R.S.; Ivanchina, N.V.; Kalinovsky, A.I.; Kharchenko, S.D.; Kicha, A.A.; Malyarenko, T.V.; Ermakova, S.P.; Dmitrenok, P.S. Aphelasteroside F, a New Asterosaponin from the Far Eastern Starfish *Aphelasterias japonica*. *Natural Product Communications* **2016**, *11*, 1934578X1601100913, doi:10.1177/1934578X1601100913.
1064. Kicha, A.A.; Ivanchina, N.V.; Huong, T.T.T.; Kalinovsky, A.I.; Dmitrenok, P.S.; Fedorov, S.N.; Dyshlovoy, S.A.; Long, P.Q.; Stonik, V.A. Two New Asterosaponins, Archasterosides A and B, from the Vietnamese Starfish *Archaster typicus* and Their Anticancer Properties. *Bioorganic & Medicinal Chemistry Letters* **2010**, *20*, 3826–3830, doi:10.1016/j.bmcl.2010.04.005.
1065. Kicha, A.A.; Malyarenko, T.V.; Kalinovsky, A.I.; Popov, R.S.; Malyarenko, O.S.; Ermakova, S.P.; Ivanchina, N.V. Polar Steroid Compounds from the Arctic Starfish Asterias Microdiscus and Their Cytotoxic Properties against Normal and Tumor Cells in Vitro. *Natural Product Research* **2020**, *0*, 1–8, doi:10.1080/14786419.2020.1834551.

1066. Malyarenko, O.S.; Malyarenko, T.V.; Kicha, A.A.; Ivanchina, N.V.; Ermakova, S.P. Effects of Polar Steroids from the Starfish *Patiria* (=*Asterina*) *pectinifera* in Combination with X-Ray Radiation on Colony Formation and Apoptosis Induction of Human Colorectal Carcinoma Cells. *Molecules* **2019**, *24*, 3154, doi:10.3390/molecules24173154.
1067. Malyarenko, T.V.; Kicha, A.A.; Ivanchina, N.V.; Kalinovskii, A.I.; Dmitrenok, P.S.; Ermakova, S.P.; Minkh, Ch.V. Asteropsiside A and Other Asterosaponins from the Starfish *Asteropsis carinifera*. *Russ Chem Bull* **2012**, *61*, 1986–1991, doi:10.1007/s11172-012-0275-3.
1068. Malyarenko, O.S.; Malyarenko, T.V.; Usoltseva, R.V.; Silchenko, A.S.; Kicha, A.A.; Ivanchina, N.V.; Ermakova, S.P. Fucoidan from Brown Algae Fucus Evanescens Potentiates the Anti-Proliferative Efficacy of Asterosaponins from Starfish *Asteropsis carinifera* in 2D and 3D Models of Melanoma Cells. *International Journal of Biological Macromolecules* **2021**, *185*, 31–39, doi:10.1016/j.ijbiomac.2021.06.080.
1069. Thao, N.P.; Luyen, B.T.T.; Kim, E.-J.; Kang, H.-K.; Kim, S.; Cuong, N.X.; Nam, N.H.; Kiem, P.V.; Minh, C.V.; Kim, Y.H. Asterosaponins from the Starfish *Astropecten monacanthus* Suppress Growth and Induce Apoptosis in HL-60, PC-3, and SNU-C5 Human Cancer Cell Lines. *Biol Pharm Bull* **2014**, *37*, 315–321, doi:10.1248/bpb.b13-00705.
1070. Thao, N.P.; Cuong, N.X.; Luyen, B.T.T.; Thanh, N.V.; Nhiem, N.X.; Koh, Y.-S.; Ly, B.M.; Nam, N.H.; Kiem, P.V.; Minh, C.V.; et al. Anti-Inflammatory Asterosaponins from the Starfish *Astropecten monacanthus*. *J. Nat. Prod.* **2013**, *76*, 1764–1770, doi:10.1021/np400492a.
1071. Zhou, J.; Cheng, G.; Cheng, G.; Tang, H.-F.; Zhang, X. Novaeguinoside II Inhibits Cell Proliferation and Induces Apoptosis of Human Brain Glioblastoma U87MG Cells through the Mitochondrial Pathway. *Brain Res* **2011**, *1372*, 22–28, doi:10.1016/j.brainres.2010.11.093.
1072. Xue, Y.-Y.; Lu, Y.-Y.; Sun, G.-Q.; Fang, F.; Ji, Y.-Q.; Tang, H.-F.; Qiu, P.-C.; Cheng, G. CN-3 Induces Mitochondrial Apoptosis in Glioma via Ros-Mediated PI3K/AKT Pathway. *Pharmazie* **2021**, *76*, 208–214, doi:10.1691/ph.2021.1363.
1073. Ivanchina, N.V.; Malyarenko, T.V.; Kicha, A.A.; Kalinovsky, A.I.; Dmitrenok, P.S.; Ermakova, S.P. Structures and Cytotoxic Activities of Two New Asterosaponins from the Antarctic Starfish *Diplasterias brucei*. *Russ J Bioorg Chem* **2011**, *37*, 499, doi:10.1134/S1068162011030083.
1074. Ivanchina, N.V.; Kalinovsky, A.I.; Kicha, A.A.; Malyarenko, T.V.; Dmitrenok, P.S.; Ermakova, S.P.; Stonik, V.A. Two New Asterosaponins from the Far Eastern Starfish *Lethasterias fusca*. *Natural Product Communications* **2012**, *7*, 1934578X1200700711, doi:10.1177/1934578X1200700711.
1075. Kicha, A.A.; Kalinovsky, A.I.; Ivanchina, N.V.; Malyarenko, T.V.; Dmitrenok, P.S.; Kuzmich, A.S.; Sokolova, E.V.; Stonik, V.A. Furostane Series Asterosaponins and Other Unusual Steroid Oligoglycosides from the Tropical Starfish *Pentaceraster regulus*. *J. Nat. Prod.* **2017**, *80*, 2761–2770, doi:10.1021/acs.jnatprod.7b00574.

1076. Quang, T.H.; Lee, D.-S.; Han, S.J.; Kim, I.C.; Yim, J.H.; Kim, Y.-C.; Oh, H. Steroids from the Cold Water Starfish Ctenodiscus Crispatus with Cytotoxic and Apoptotic Effects on Human Hepatocellular Carcinoma and Glioblastoma Cells. *Bulletin of the Korean Chemical Society* **2014**, *35*, 2335–2341, doi:10.5012/bkcs.2014.35.8.2335.
1077. Palyanova, N.V.; Pankova, T.M.; Starostina, M.V.; Kicha, A.A.; Ivanchina, N.V.; Stonik, V.A. Neuritogenic and Neuroprotective Effects of Polar Steroids from the Far East Starfishes *Patiria pectinifera* and *Distolasterias nipon*. *Mar Drugs* **2013**, *11*, 1440–1455, doi:10.3390/md11051440.
1078. Thao, N.P.; Cuong, N.X.; Luyen, B.T.T.; Nam, N.H.; Cuong, P.V.; Thanh, N.V.; Nhiem, N.X.; Hanh, T.T.H.; Kim, E.-J.; Kang, H.-K.; et al. Steroidal Constituents from the Starfish *Astropecten polyacanthus* and Their Anticancer Effects. *Chemical and Pharmaceutical Bulletin* **2013**, *61*, 1044–1051, doi:10.1248/cpb.c13-00490.
1079. Kicha, A.A.; Kalinovsky, A.I.; Ivanchina, N.V.; Malyarenko, T.V.; Dmitrenok, P.S.; Ermakova, S.P.; Stonik, V.A. Four New Asterosaponins, Hippasteriosides A – D, from the Far Eastern Starfish *Hippasteria kurilensis*. *Chemistry & Biodiversity* **2011**, *8*, 166–175, doi:10.1002/cbdv.200900402.
1080. Malyarenko, T.V.; Malyarenko Vishchuk, O.S.; Ivanchina, N.V.; Kalinovsky, A.I.; Popov, R.S.; Kicha, A.A. Four New Sulfated Polar Steroids from the Far Eastern Starfish *Leptasterias ochotensis*: Structures and Activities. *Mar Drugs* **2015**, *13*, 4418–4435, doi:10.3390/md13074418.
1081. Malyarenko, T.V.; Kicha, A.A.; Kalinovsky, A.I.; Dmitrenok, P.S.; Malyarenko, O.S.; Kuzmich, A.S.; Stonik, V.A.; Ivanchina, N.V. New Triterpene Glycosides from the Far Eastern Starfish *Solaster pacificus* and Their Biological Activity. *Biomolecules* **2021**, *11*, 427, doi:10.3390/biom11030427.
1082. Wätjen, W.; Ebada, S.S.; Bergermann, A.; Chovolou, Y.; Totzke, F.; Kubbutat, M.H.G.; Lin, W.; Proksch, P. Cytotoxic Effects of the Anthraquinone Derivatives 1′-Deoxyrhodoptilometrin and (S)-(-)-Rhodoptilometrin Isolated from the Marine Echinoderm *Comanthus* sp. *Arch Toxicol* **2017**, *91*, 1485–1495, doi:10.1007/s00204-016-1787-7.
1083. Hasan, I.; Gerdol, M.; Fujii, Y.; Ozeki, Y. Functional Characterization of OXYL, A SghC1qDC LacNAc-Specific Lectin from The Crinoid Feather Star *Anneissia japonica*. *Mar Drugs* **2019**, *17*, E136, doi:10.3390/md17020136.
1084. Cirino, P.; Brunet, C.; Ciaravolo, M.; Galasso, C.; Musco, L.; Vega Fernández, T.; Sansone, C.; Toscano, A. The Sea Urchin *Arbacia lixula*: A Novel Natural Source of Astaxanthin. *Mar Drugs* **2017**, *15*, doi:10.3390/md15060187.
1085. Hatakeyama, T.; Ichise, A.; Yonekura, T.; Unno, H.; Goda, S.; Nakagawa, H. CDNA Cloning and Characterization of a Rhamnose-Binding Lectin SUL-I from the Toxopneustid Sea Urchin *Toxopneustes pileolus* Venom. *Toxicon* **2015**, *94*, 8–15, doi:10.1016/j.toxicon.2014.11.236.
1086. Wu, L.; Yang, X.; Duan, X.; Cui, L.; Li, G. Exogenous Expression of Marine Lectins DlFBL and SpRBL Induces Cancer Cell Apoptosis Possibly through PRMT5-E2F-1 Pathway. *Sci Rep* **2014**, *4*, 4505, doi:10.1038/srep04505.
1087. Li, C.; Haug, T.; Moe, M.K.; Styrvold, O.B.; Stensvåg, K. Centrocins: Isolation and Characterization of Novel Dimeric Antimicrobial Peptides from the Green Sea Urchin, *Strongylocentrotus droebachiensis*. *Developmental & Comparative Immunology* **2010**, *34*, 959–968, doi:10.1016/j.dci.2010.04.004.

1088. Li, C.; Blencke, H.-M.; Haug, T.; Jørgensen, Ø.; Stensvåg, K. Expression of Antimicrobial Peptides in Coelomocytes and Embryos of the Green Sea Urchin (*Strongylocentrotus droebachiensis*). *Dev. Comp. Immunol.* **2014**, *43*, 106–113, doi:10.1016/j.dci.2013.10.013.
1089. Schillaci, D.; Vitale, M.; Cusimano, M.G.; Arizza, V. Fragments of β-Thymosin from the Sea Urchin *Paracentrotus lividus* as Potential Antimicrobial Peptides against *Staphylococcal* Biofilms. *Ann. N. Y. Acad. Sci.* **2012**, *1270*, 79–85, doi:10.1111/j.1749-6632.2012.06652.x.
1090. Schillaci, D.; Cusimano, M.G.; Spinello, A.; Barone, G.; Russo, D.; Vitale, M.; Parrinello, D.; Arizza, V. Paracentrin 1, a Synthetic Antimicrobial Peptide from the Sea-Urchin *Paracentrotus Lividus*, Interferes with Staphylococcal and *Pseudomonas aeruginosa* Biofilm Formation. *AMB Express* **2014**, *4*, 78, doi:10.1186/s13568-014-0078-z.
1091. Solstad, R.G.; Li, C.; Isaksson, J.; Johansen, J.; Svenson, J.; Stensvåg, K.; Haug, T. Novel Antimicrobial Peptides EeCentrocins 1, 2 and EeStrongylocin 2 from the Edible Sea Urchin *Echinus esculentus* Have 6-Br-Trp Post-Translational Modifications. *PLoS ONE* **2016**, *11*, e0151820, doi:10.1371/journal.pone.0151820.
1092. Spinello, A.; Cusimano, M.G.; Schillaci, D.; Inguglia, L.; Barone, G.; Arizza, V. Antimicrobial and Antibiofilm Activity of a Recombinant Fragment of β-Thymosin of Sea Urchin *Paracentrotus lividus*. *Mar Drugs* **2018**, *16*, doi:10.3390/md16100366.
1093. Brasseur, L.; Hennebert, E.; Fievez, L.; Caulier, G.; Bureau, F.; Tafforeau, L.; Flammang, P.; Gerbaux, P.; Eeckhaut, I. The Roles of Spinochromes in Four Shallow Water Tropical Sea Urchins and Their Potential as Bioactive Pharmacological Agents. *Mar Drugs* **2017**, *15*, doi:10.3390/md15060179.
1094. Hu, Z.; Ye, L.; Xing, Y.; Hu, J.; Xi, T. Combined SEP and Anti-PD-L1 Antibody Produces a Synergistic Antitumor Effect in B16-F10 Melanoma-Bearing Mice. *Sci Rep* **2018**, *8*, 217, doi:10.1038/s41598-017-18641-y.
1095. Ke, M.; Wang, H.; Zhou, Y.; Li, J.; Liu, Y.; Zhang, M.; Dou, J.; Xi, T.; Shen, B.; Zhou, C. SEP Enhanced the Antitumor Activity of 5-Fluorouracil by up-Regulating NKG2D/MICA and Reversed Immune Suppression via Inhibiting ROS and Caspase-3 in Mice. *Oncotarget* **2016**, *7*, 49509–49526, doi:10.18632/oncotarget.10375.
1096. Xie, X.; Zhou, Y.; Wang, X.; Guo, J.; Li, J.; Fan, H.; Dou, J.; Shen, B.; Zhou, C. Enhanced Antitumor Activity of Gemcitabine by Polysaccharide-Induced NK Cell Activation and Immune Cytotoxicity Reduction in Vitro/Vivo. *Carbohydr Polym* **2017**, *173*, 360–371, doi:10.1016/j.carbpol.2017.06.024.
1097. Teixeira, F.C.O.B.; Kozlowski, E.O.; Micheli, K.V. de A.; Vilela-Silva, A.C.E.S.; Borsig, L.; Pavão, M.S.G. Sulfated Fucans and a Sulfated Galactan from Sea Urchins as Potent Inhibitors of Selectin-Dependent Hematogenous Metastasis. *Glycobiology* **2018**, *28*, 427–434, doi:10.1093/glycob/cwy020.
1098. Vasconcelos, A.A.; Sucupira, I.D.; Guedes, A.L.; Queiroz, I.N.; Frattani, F.S.; Fonseca, R.J.; Pomin, V.H. Anticoagulant and Antithrombotic Properties of Three Structurally Correlated Sea Urchin Sulfated Glycans and Their Low-Molecular-Weight Derivatives. *Mar Drugs* **2018**, *16*, doi:10.3390/md16090304.
1099. Castellano, I.; Migliaccio, O.; D'Aniello, S.; Merlino, A.; Napolitano, A.; Palumbo, A. Shedding Light on Ovothiol Biosynthesis in Marine Metazoans. *Sci Rep* **2016**, *6*, 21506, doi:10.1038/srep21506.

1100. Kim, H.K.; Youm, J.B.; Jeong, S.H.; Lee, S.R.; Song, I.-S.; Ko, T.H.; Pronto, J.R.; Ko, K.S.; Rhee, B.D.; Kim, N.; et al. Echinochrome A Regulates Phosphorylation of Phospholamban Ser16 and Thr17 Suppressing Cardiac SERCA2A Ca^{2+} Reuptake. *Pflugers Arch.* **2015**, *467*, 2151–2163, doi:10.1007/s00424-014-1648-2.

1101. Kim, H.K.; Cho, S.W.; Heo, H.J.; Jeong, S.H.; Kim, M.; Ko, K.S.; Rhee, B.D.; Mishchenko, N.P.; Vasileva, E.A.; Fedoreyev, S.A.; et al. A Novel Atypical PKC-Iota Inhibitor, Echinochrome A, Enhances Cardiomyocyte Differentiation from Mouse Embryonic Stem Cells. *Mar Drugs* **2018**, *16*, doi:10.3390/md16060192.

1102. Luo, L.; Wu, M.; Xu, L.; Lian, W.; Xiang, J.; Lu, F.; Gao, N.; Xiao, C.; Wang, S.; Zhao, J. Comparison of Physicochemical Characteristics and Anticoagulant Activities of Polysaccharides from Three Sea Cucumbers. *Mar Drugs* **2013**, *11*, 399–417, doi:10.3390/md11020399.

1103. La, M.-P.; Li, C.; Li, L.; Sun, P.; Tang, H.; Liu, B.-S.; Gong, W.; Han, H.; Yi, Y.-H.; Zhang, W. New Bioactive Sulfated Alkenes from the Sea Cucumber *Apostichopus japonicus*. *Chemistry & Biodiversity* **2012**, *9*, 1166–1171, doi:10.1002/cbdv.201100324.

1104. Omran, N.E.; Khedr, A.M. Structure Elucidation, Protein Profile and the Antitumor Effect of the Biological Active Substance Extracted from Sea Cucumber *Holothuria polii*. *Toxicol Ind Health* **2015**, *31*, 1–8, doi:10.1177/0748233712466135.

1105. Balabanova, L.; Golotin, V.; Kovalchuk, S.; Bulgakov, A.; Likhatskaya, G.; Son, O.; Rasskazov, V. A Novel Bifunctional Hybrid with Marine Bacterium Alkaline Phosphatase and Far Eastern Holothurian Mannan-Binding Lectin Activities. *PLoS ONE* **2014**, *9*, e112729, doi:10.1371/journal.pone.0112729.

1106. Han, L.-L.; Yuan, Z.; Dahms, H.-U.; Li, Q.-Y.; Zhang, Q.-Z.; Wu, R.-J.; Tan, J.; Zou, X.-Y.; Hou, L. Molecular Cloning, Characterization and Expression Analysis of a C-Type Lectin (AJCTL) from the Sea Cucumber *Apostichopus japonicus*. *Immunol. Lett.* **2012**, *143*, 137–145, doi:10.1016/j.imlet.2011.12.004.

1107. Moura, R. da M.; Aragão, K.S.; de Melo, A.A.; Carneiro, R.F.; Osório, C.B.H.; Luz, P.B.; de Queiroz, A.F.S.; Dos Santos, E.A.; de Alencar, N.M.N.; Cavada, B.S. *Holothuria grisea* Agglutinin (HGA): The First Invertebrate Lectin with Anti-Inflammatory Effects. *Fundam Clin Pharmacol* **2013**, *27*, 656–668, doi:10.1111/j.1472-8206.2012.01073.x.

1108. Moura, R.M.; Melo, A.A.; Carneiro, R.F.; Rodrigues, C.R.F.; Delatorre, P.; Nascimento, K.S.; Saker-Sampaio, S.; Nagano, C.S.; Cavada, B.S.; Sampaio, A.H. Hemagglutinating/Hemolytic Activities in Extracts of Marine Invertebrates from the Brazilian Coast and Isolation of Two Lectins from the Marine Sponge *Cliona varians* and the Sea Cucumber *Holothuria grisea*. *An. Acad. Bras. Cienc.* **2015**, *87*, 973–984, doi:10.1590/0001-3765201520140399.

1109. Shimizu, Y.; Yamazaki, H.; Yoshida, S.; Yonekura, M.; Kouzuma, Y. Molecular Cloning, Functional Expression, and Characterization of Isolectin Genes of Hemolytic Lectin CEL-III from the Marine Invertebrate *Cucumaria echinata*. *Biosci. Biotechnol. Biochem.* **2012**, *76*, 276–282, doi:10.1271/bbb.110635.

1110. Wang, Y.; Xue, Z.; Yi, Q.; Wang, H.; Wang, L.; Lu, G.; Liu, Y.; Qu, C.; Li, Y.; Song, L. A Novel Fucolectin from *Apostichopus japonicus* with Broad PAMP Recognition Pattern. *Fish Shellfish Immunol.* **2018**, *77*, 402–409, doi:10.1016/j.fsi.2018.04.013.

1111. Schillaci, D.; Cusimano, M.G.; Cunsolo, V.; Saletti, R.; Russo, D.; Vazzana, M.; Vitale, M.; Arizza, V. Immune Mediators of Sea-Cucumber *Holothuria tubulosa* (Echinodermata) as Source of Novel Antimicrobial and Anti-Staphylococcal Biofilm Agents. *AMB Express* **2013**, *3*, 35, doi:10.1186/2191-0855-3-35.

1112. Cai, Y.; Yang, W.; Yin, R.; Zhou, L.; Li, Z.; Wu, M.; Zhao, J. An Anticoagulant Fucan Sulfate with Hexasaccharide Repeating Units from the Sea Cucumber *Holothuria albiventer*. *Carbohydr. Res.* **2018**, *464*, 12–18, doi:10.1016/j.carres.2018.05.007.
1113. Cao, R.-A.; Surayot, U.; You, S. Structural Characterization of Immunostimulating Protein-Sulfated Fucan Complex Extracted from the Body Wall of a Sea Cucumber, *Stichopus japonicus*. *Int. J. Biol. Macromol.* **2017**, *99*, 539–548, doi:10.1016/j.ijbiomac.2017.03.026.
1114. Chen, S.; Hu, Y.; Ye, X.; Li, G.; Yu, G.; Xue, C.; Chai, W. Sequence Determination and Anticoagulant and Antithrombotic Activities of a Novel Sulfated Fucan Isolated from the Sea Cucumber *Isostichopus badionotus*. *Biochim. Biophys. Acta* **2012**, *1820*, 989–1000, doi:10.1016/j.bbagen.2012.03.002.
1115. Kariya, Y.; Mulloy, B.; Imai, K.; Tominaga, A.; Kaneko, T.; Asari, A.; Suzuki, K.; Masuda, H.; Kyogashima, M.; Ishii, T. Isolation and Partial Characterization of Fucan Sulfates from the Body Wall of Sea Cucumber *Stichopus japonicus* and Their Ability to Inhibit Osteoclastogenesis. *Carbohydr. Res.* **2004**, *339*, 1339–1346, doi:10.1016/j.carres.2004.02.025.
1116. Li, S.; Li, J.; Zhi, Z.; Wei, C.; Wang, W.; Ding, T.; Ye, X.; Hu, Y.; Linhardt, R.J.; Chen, S. Macromolecular Properties and Hypolipidemic Effects of Four Sulfated Polysaccharides from Sea Cucumbers. *Carbohydr Polym* **2017**, *173*, 330–337, doi:10.1016/j.carbpol.2017.05.063.
1117. Li, Q.; Cai, C.; Chang, Y.; Zhang, F.; Linhardt, R.J.; Xue, C.; Li, G.; Yu, G. A Novel Structural Fucosylated Chondroitin Sulfate from *Holothuria mexicana* and Its Effects on Growth Factors Binding and Anticoagulation. *Carbohydr Polym* **2018**, *181*, 1160–1168, doi:10.1016/j.carbpol.2017.10.100.
1118. Liu, X.; Hao, J.; Shan, X.; Zhang, X.; Zhao, X.; Li, Q.; Wang, X.; Cai, C.; Li, G.; Yu, G. Antithrombotic Activities of Fucosylated Chondroitin Sulfates and Their Depolymerized Fragments from Two Sea Cucumbers. *Carbohydr Polym* **2016**, *152*, 343–350, doi:10.1016/j.carbpol.2016.06.106.
1119. Panagos, C.G.; Thomson, D.S.; Moss, C.; Hughes, A.D.; Kelly, M.S.; Liu, Y.; Chai, W.; Venkatasamy, R.; Spina, D.; Page, C.P.; et al. Fucosylated Chondroitin Sulfates from the Body Wall of the Sea Cucumber *Holothuria forskali*: Conformation, Selectin Binding, and Biological Activity. *J. Biol. Chem.* **2014**, *289*, 28284–28298, doi:10.1074/jbc.M114.572297.
1120. Shang, F.; Mou, R.; Zhang, Z.; Gao, N.; Lin, L.; Li, Z.; Wu, M.; Zhao, J. Structural Analysis and Anticoagulant Activities of Three Highly Regular Fucan Sulfates as Novel Intrinsic Factor Xase Inhibitors. *Carbohydr Polym* **2018**, *195*, 257–266, doi:10.1016/j.carbpol.2018.04.117.
1121. Surayot, U.; Lee, S.; You, S. Effects of Sulfated Fucan from the Sea Cucumber *Stichopus japonicus* on Natural Killer Cell Activation and Cytotoxicity. *Int. J. Biol. Macromol.* **2018**, *108*, 177–184, doi:10.1016/j.ijbiomac.2017.11.102.
1122. Thinh, P.D.; Ly, B.M.; Usoltseva, R.V.; Shevchenko, N.M.; Rasin, A.B.; Anastyuk, S.D.; Malyarenko, O.S.; Zvyagintseva, T.N.; San, P.T.; Ermakova, S.P. A Novel Sulfated Fucan from Vietnamese Sea Cucumber *Stichopus variegatus*: Isolation, Structure and Anticancer Activity in Vitro. *Int. J. Biol. Macromol.* **2018**, *117*, 1101–1109, doi:10.1016/j.ijbiomac.2018.06.017.

1123. Ustyuzhanina, N.E.; Bilan, M.I.; Dmitrenok, A.S.; Shashkov, A.S.; Nifantiev, N.E.; Usov, A.I. Two Structurally Similar Fucosylated Chondroitin Sulfates from the Holothurian Species *Stichopus chloronotus* and *Stichopus horrens*. *Carbohydr Polym* **2018**, *189*, 10–14, doi:10.1016/j.carbpol.2018.02.008.
1124. Ustyuzhanina, N.E.; Bilan, M.I.; Panina, E.G.; Sanamyan, N.P.; Dmitrenok, A.S.; Tsvetkova, E.A.; Ushakova, N.A.; Shashkov, A.S.; Nifantiev, N.E.; Usov, A.I. Structure and Anti-Inflammatory Activity of a New Unusual Fucosylated *Chondroitin sulfate* from *Cucumaria djakonovi*. *Mar Drugs* **2018**, *16*, doi:10.3390/md16100389.
1125. Wang, H.; Yang, S.; Wang, Y.; Jiang, T.; Li, S.; Lv, Z. Immunoenhancement Effects of Glycosaminoglycan from *Apostichopus japonicus*: In Vitro and In Cyclophosphamide-Induced Immunosuppressed Mice Studies. *Mar Drugs* **2017**, *15*, E347, doi:10.3390/md15110347.
1126. Wu, M.; Xu, L.; Zhao, L.; Xiao, C.; Gao, N.; Luo, L.; Yang, L.; Li, Z.; Chen, L.; Zhao, J. Structural Analysis and Anticoagulant Activities of the Novel Sulfated Fucan Possessing a Regular Well-Defined Repeating Unit from Sea Cucumber. *Marine Drugs* **2015**, *13*, 2063–2084, doi:10.3390/md13042063.
1127. Yang, L.; Wang, Y.; Yang, S.; Lv, Z. Separation, Purification, Structures and Anticoagulant Activities of Fucosylated Chondroitin Sulfates from *Holothuria scabra*. *Int. J. Biol. Macromol.* **2018**, *108*, 710–718, doi:10.1016/j.ijbiomac.2017.11.058.
1128. Yang, W.; Cai, Y.; Yin, R.; Lin, L.; Li, Z.; Wu, M.; Zhao, J. Structural Analysis and Anticoagulant Activities of Two Sulfated Polysaccharides from the Sea Cucumber *Holothuria coluber*. *Int. J. Biol. Macromol.* **2018**, *115*, 1055–1062, doi:10.1016/j.ijbiomac.2018.04.175.
1129. Aminin, D.L.; Chaykina, E.L.; Agafonova, I.G.; Avilov, S.A.; Kalinin, V.I.; Stonik, V.A. Antitumor Activity of the Immunomodulatory Lead Cumaside. *Int. Immunopharmacol.* **2010**, *10*, 648–654, doi:10.1016/j.intimp.2010.03.003.
1130. Bahrami, Y.; Zhang, W.; M M Franco, C. Distribution of Saponins in the Sea Cucumber *Holothuria lessoni*; the Body Wall Versus the Viscera, and Their Biological Activities. *Mar Drugs* **2018**, *16*, E423, doi:10.3390/md16110423.
1131. Cuong, N.X.; Vien, L.T.; Hoang, L.; Hanh, T.T.H.; Thao, D.T.; Thanh, N.V.; Nam, N.H.; Thung, D.C.; Kiem, P.V.; Minh, C.V. Cytotoxic Triterpene Diglycosides from the Sea Cucumber *Stichopus horrens*. *Bioorg. Med. Chem. Lett.* **2017**, *27*, 2939–2942, doi:10.1016/j.bmcl.2017.05.003.
1132. Menchinskaya, E.S.; Pislyagin, E.A.; Kovalchyk, S.N.; Davydova, V.N.; Silchenko, A.S.; Avilov, S.A.; Kalinin, V.I.; Aminin, D.L. Antitumor Activity of Cucumarioside A2-2. *Chemotherapy* **2013**, *59*, 181–191, doi:10.1159/000354156.
1133. Sajwani, F.H.; Collin, P.; Adrian, T.E. Frondoside A Potentiates the Effects of Conventional Therapeutic Agents in Acute Leukemia. *Leuk. Res.* **2017**, *63*, 98–108, doi:10.1016/j.leukres.2017.11.002.
1134. Vien, L.T.; Hoang, L.; Hanh, T.T.H.; Thanh, N.V.; Cuong, N.X.; Nam, N.H.; Thung, D.C.; Kiem, P.V.; Minh, C.V. Triterpene Tetraglycosides from the Sea Cucumber *Stichopus horrens*. *Nat. Prod. Res.* **2018**, *32*, 1039–1043, doi:10.1080/14786419.2017.1378206.

1135. Yu, S.; Ye, X.; Huang, H.; Peng, R.; Su, Z.; Lian, X.-Y.; Zhang, Z. Bioactive Sulfated Saponins from Sea Cucumber *Holothuria moebii*. *Planta Med.* **2015**, *81*, 152–159, doi:10.1055/s-0034-1383404.
1136. Yun, S.-H.; Shin, S.-W.; Stonik, V.A.; Park, J.-I. Ceramide as a Target of Marine Triterpene Glycosides for Treatment of Human Myeloid Leukemia. *Mar Drugs* **2016**, *14*, doi:10.3390/md14110205.
1137. Yue, Z.; Wang, A.; Zhu, Z.; Tao, L.; Li, Y.; Zhou, L.; Chen, W.; Lu, Y. Holothurian Glycosaminoglycan Inhibits Metastasis via Inhibition of P-Selectin in B16F10 Melanoma Cells. *Mol. Cell. Biochem.* **2015**, *410*, 143–154, doi:10.1007/s11010-015-2546-4.
1138. Torii, M.; Hitora, Y.; Kato, H.; Koyanagi, Y.; Kawahara, T.; Losung, F.; Mangindaan, R.E.P.; Tsukamoto, S. Siladenoserinols M–P, Sulfonated Serinol Derivatives from a Tunicate. *Tetrahedron* **2018**, *74*, 7516–7521, doi:10.1016/j.tet.2018.11.031.
1139. Dou, X.; Dong, B. Origins and Bioactivities of Natural Compounds Derived from Marine Ascidians and Their Symbionts. *Marine Drugs* **2019**, *17*, 670, doi:10.3390/md17120670.
1140. Lyakhova, I.A.; Bryukhovetsky, I.S.; Kudryavtsev, I.V.; Khotimchenko, Y.S.; Zhidkov, M.E.; Kantemirov, A.V. Antitumor Activity of Fascaplysin Derivatives on Glioblastoma Model In Vitro. *Bull Exp Biol Med* **2018**, *164*, 666–672, doi:10.1007/s10517-018-4055-4.
1141. Bracegirdle, J.; Robertson, L.P.; Hume, P.A.; Page, M.J.; Sharrock, A.V.; Ackerley, D.F.; Carroll, A.R.; Keyzers, R.A. Lamellarin Sulfates from the Pacific Tunicate *Didemnum ternerratum*. *J. Nat. Prod.* **2019**, *82*, 2000–2008, doi:10.1021/acs.jnatprod.9b00493.
1142. Suzuki, T.; Kubota, T.; Kobayashi, J. Eudistomidins H-K, New β-Carboline Alkaloids from the Okinawan Marine Tunicate *Eudistoma glaucus*. *Bioorg Med Chem Lett* **2011**, *21*, 4220–4223, doi:10.1016/j.bmcl.2011.05.072.
1143. Chan, S.T.S.; Pearce, A.N.; Page, M.J.; Kaiser, M.; Copp, B.R. Antimalarial β-Carbolines from the New Zealand Ascidian *Pseudodistoma opacum*. *J Nat Prod* **2011**, *74*, 1972–1979, doi:10.1021/np200509g.
1144. Murcia, C.; Coello, L.; Fernández, R.; Martín, M.J.; Reyes, F.; Francesch, A.; Munt, S.; Cuevas, C. Tanjungides A and B: New Antitumoral Bromoindole Derived Compounds from Diazona Cf Formosa. Isolation and Total Synthesis. *Marine Drugs* **2014**, *12*, 1116–1130, doi:10.3390/md12021116.
1145. Finlayson, R.; Pearce, A.N.; Page, M.J.; Kaiser, M.; Bourguet-Kondracki, M.-L.; Harper, J.L.; Webb, V.L.; Copp, B.R. Didemnidines A and B, Indole Spermidine Alkaloids from the New Zealand Ascidian *Didemnum* sp. *J. Nat. Prod.* **2011**, *74*, 888–892, doi:10.1021/np1008619.
1146. Núñez-Pons, L.; Nieto, R.M.; Avila, C.; Jiménez, C.; Rodríguez, J. Mass Spectrometry Detection of Minor New Meridianins from the Antarctic Colonial Ascidians *Aplidium falklandicum* and *Aplidium meridianum*. *Journal of Mass Spectrometry* **2015**, *50*, 103–111, doi:10.1002/jms.3502.
1147. Núñez-Pons, L.; Carbone, M.; Vázquez, J.; Rodríguez, J.; Nieto, R.M.; Varela, M.M.; Gavagnin, M.; Avila, C. Natural Products from Antarctic Colonial Ascidians of the Genera *Aplidium* and *Synoicum*: Variability and Defensive Role. *Marine Drugs* **2012**, *10*, 1741–1764, doi:10.3390/md10081741.

1148. Núñez-Pons, L.; Forestieri, R.; Nieto, R.M.; Varela, M.; Nappo, M.; Rodríguez, J.; Jiménez, C.; Castelluccio, F.; Carbone, M.; Ramos-Espla, A.; et al. Chemical Defenses of Tunicates of the Genus *Aplidium* from the Weddell Sea (Antarctica). *Polar Biol* **2010**, *33*, 1319–1329, doi:10.1007/s00300-010-0819-7.
1149. Noguez, J.H.; Diyabalanage, T.K.K.; Miyata, Y.; Xie, X.-S.; Valeriote, F.A.; Amsler, C.D.; McClintock, J.B.; Baker, B.J. Palmerolide Macrolides from the Antarctic Tunicate *Synoicum adareanum*. *Bioorg Med Chem* **2011**, *19*, 6608–6614, doi:10.1016/j.bmc.2011.06.004.
1150. Sikorska, J.; Hau, A.M.; Anklin, C.; Parker-Nance, S.; Davies-Coleman, M.T.; Ishmael, J.E.; McPhail, K.L. Mandelalides A–D, Cytotoxic Macrolides from a New Lissoclinum Species of South African Tunicate. *J. Org. Chem.* **2012**, *77*, 6066–6075, doi:10.1021/jo3008622.
1151. Dyshlovoy, S.A.; Fedorov, S.N.; Kalinovsky, A.I.; Shubina, L.K.; Bokemeyer, C.; Stonik, V.A.; Honecker, F. Mycalamide A Shows Cytotoxic Properties and Prevents EGF-Induced Neoplastic Transformation through Inhibition of Nuclear Factors. *Marine Drugs* **2012**, *10*, 1212–1224, doi:10.3390/md10061212.
1152. Imperatore, C.; Luciano, P.; Aiello, A.; Vitalone, R.; Irace, C.; Santamaria, R.; Li, J.; Guo, Y.-W.; Menna, M. Structure and Configuration of Phosphoeleganin, a Protein Tyrosine Phosphatase 1B Inhibitor from the Mediterranean Ascidian *Sidnyum elegans*. *J. Nat. Prod.* **2016**, *79*, 1144–1148, doi:10.1021/acs.jnatprod.6b00063.
1153. Smitha, D.; Kumar, M.M.K.; Ramana, H.; Rao, D.V. Rubrolide R: A New Furanone Metabolite from the Ascidian *Synoicum* of the Indian Ocean. *Nat Prod Res* **2014**, *28*, 12–17, doi:10.1080/14786419.2013.827194.
1154. Di Bella, M.D.; Fedders, H.; Leippe, M.; De Leo, G. A Preliminary Study on Antimicrobial Peptides in the Naturally Damaged Tunic of *Ciona intestinalis* (Tunicata). In *Science against microbial pathogens: communicating current research and technological advances*; Méndez-Vilas, A: Formatex Research Center, Badajoz, 2011; pp. 1003–1007.
1155. Di Bella, M.D.; Fedders, H.; Leo, G.D.; Leippe, M. Localization of Antimicrobial Peptides in the Tunic of *Ciona intestinalis* (Ascidiacea, Tunicata) and Their Involvement in Local Inflammatory-like Reactions. *Results in immunology* **2011**, doi:10.1016/j.rinim.2011.09.001.
1156. Fedders, H.; Podschun, R.; Leippe, M. The Antimicrobial Peptide Ci-MAM-A24 Is Highly Active against Multidrug-Resistant and Anaerobic Bacteria Pathogenic for Humans. *Int J Antimicrob Agents* **2010**, *36*, 264–266, doi:10.1016/j.ijantimicag.2010.04.008.
1157. Jena, P.; Mishra, B.; Leippe, M.; Hasilik, A.; Griffiths, G.; Sonawane, A. Membrane-Active Antimicrobial Peptides and Human Placental Lysosomal Extracts Are Highly Active against Mycobacteria. *Peptides* **2011**, *32*, 881–887, doi:10.1016/j.peptides.2011.03.002.
1158. Zhao, J.; Wei, J.; Liu, M.; Xiao, L.; Wu, N.; Liu, G.; Huang, H.; Zhang, Y.; Zheng, L.; Lin, X. Cloning, Characterization and Expression of a CDNA Encoding a Granulin-like Polypeptide in *Ciona savignyi*. *Biochimie* **2013**, *95*, 1611–1619, doi:10.1016/j.biochi.2013.05.001.
1159. Asolkar, R.N.; Kirkland, T.N.; Jensen, P.R.; Fenical, W. Arenimycin, an Antibiotic Effective against Rifampin- and Methicillin-Resistant *Staphylococcus aureus* from the Marine Actinomycete *Salinispora arenicola*. *J Antibiot (Tokyo)* **2010**, *63*, 37–39, doi:10.1038/ja.2009.114.

1160. Dou, X.; Li, X.; Yu, H.; Dong, B. Dual Roles of Ascidian Chondromodulin-1: Promoting Cell Proliferation Whilst Suppressing the Growth of Tumor Cells. *Mar Drugs* **2018**, *16*, E59, doi:10.3390/md16020059.
1161. Li, J.L.; Han, S.C.; Yoo, E.S.; Shin, S.; Hong, J.; Cui, Z.; Li, H.; Jung, J.H. Anti-Inflammatory Amino Acid Derivatives. *J Nat Prod* **2011**, *74*, 1792–1797, doi:10.1021/np200397g.
